# A Molecular Machine Directs the Synthesis of a Rotaxane

**DOI:** 10.1002/anie.202520085

**Published:** 2025-11-10

**Authors:** Robert Kluifhooft, Tommy Wachsmuth, Bob Barthel, Mira Müller, Ann‐Kathrin Rückert, Michael Kathan

**Affiliations:** ^1^ Humboldt Universität zu Berlin, Institut für Chemie Brook Taylor Str. 2 12489 Berlin Germany

**Keywords:** Molecular machines, Photochemistry, Rotaxanes, Synthetic methods

## Abstract

Preorganization by assembly or templating strategies is frequently used in the synthesis of rotaxanes. While these approaches have led to complex interlocked molecules, they are often limited by strict compositional requirements of templates and starting materials. Here, we use a molecular machine to direct the synthesis of a rotaxane by active shaping of starting materials through mechanical winding. Light induced rotation of a molecular motor actively winds a molecular strand around an axle, forming discrete, thermodynamically disfavored crossings between these two parts. Covalent capture preserves the kinetically stable entanglements, transforming the strand into a macrocycle that is subsequently released and mechanically trapped on the axle, yielding a rotaxane. Our machine‐directed strategy pioneers a new way of synthesizing rotaxanes by active mechanical shaping of molecular building blocks, enabling access to interlocked architectures beyond the reach of traditional assembly and templating approaches.

## Graphical abstract

A light‐driven molecular machine winds a flexible strand around a rigid axle to synthesize a rotaxane. This machine‐directed approach enables access to molecular shapes previously unattainable by assembly or templating, demonstrating a generalizable strategy for expanding the chemical space of mechanically bonded molecules.

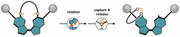

## Introduction

Achieving macroscopic‐level mechanical precision at the molecular scale could enable the construction of complex interlocked structures, such as molecular fabrics,^[^ ^1^, ^2^, ^3^ ^]^ chains,^[^ ^4^, ^5^, ^6^ ^]^ and other intricate materials. This ambitious task may appear daunting as the molecular world is governed by random thermal motion. However, its successful implementation would open up uncharted chemical space, by enabling the mechanical shaping of molecules.^[^ ^7^, ^8^ ^]^ Its importance can be illustrated by Nature's development of molecular machines capable of guiding the shaping process of biomacromolecules that is vital for their correct functioning. For example, proteins are folded or even knotted by chaperones^[^ ^9^, ^10^, ^11^ ^]^ and DNA is twisted or unknotted/unlinked by topoisomerase to enable proper readout by the ribosome.^[^ ^12^, ^13^, ^14^ ^]^ Similar to Nature's ability to shape molecules, synthetic strategies have been developed to construct mechanically interlocked molecules (MIMs)—knots, links and rotaxanes—which have sparked an immense interest for scientists in the past and current century.^[^ ^15^, ^16^, ^17^, ^18^ ^]^ Among these MIMs, rotaxanes are characterized by a linear dumbbell‐shaped axle that threads one (or more) macrocycle(s) between its two bulky stopper ends. This type of MIM was first postulated by Frisch and Wasserman in 1961.^[^ ^19^ ^]^ However, the first synthesis was reported by Harrison and Harrison in 1967, who employed a purely statistical approach.^[^ ^20^ ^]^ Simultaneously, Schill and Zollenkopf used a directed approach by an intramolecular ring closing reaction around an axle with subsequent liberation of the macrocycle.^[^ ^21^ ^]^

Nowadays, template approaches—a method that has been introduced by the groups of Sauvage and Stoddart^[^ ^22^, ^23^, ^24^ ^]^—dominate the synthesis of rotaxanes.^[^ ^25^, ^26^, ^27^, ^28^ ^]^ The ability to form rotaxanes of increasing complexity has, for instance, initiated the development of shuttling and pumping systems where macrocycles are trapped on an axle by redox processes,^[^ ^29^, ^30^, ^31^ ^]^ chemical stimuli,^[^ ^32^, ^33^ ^]^ or light.^[^ ^34^, ^35^ ^]^ A common feature of these examples is that supramolecular assembly is necessary to facilitate the key mechanical prerequisite to synthesize a rotaxane: establishing at least two crossings between axle and ring to form the mechanical bond between the two parts. In contrast, unidirectional molecular motors offer a fundamentally different strategy: rather than relying on noncovalent organization, they actively enforce mechanical entanglement through directed motion. These motors have been shown to translate stimulus‐driven rotation into various forms of mechanical work, including the build‐up of mechanical strain,^[^ ^36^ ^]^ contraction of gels,^[^ ^37^, ^38^, ^39^ ^]^ shifting of chemical equilibria,^[^ ^40^ ^]^ coupled motion,^[^ ^41^ ^]^ and mechanical threading.^[^ ^42^ ^]^ In a recent study, our group demonstrated that the unidirectional motion of rotary molecular motors can be harnessed to direct the synthesis of catenanes.^[^ ^43^ ^]^ In this machine‐directed approach, light and heat were used to precisely entangle a bismacrocyclic structure, selectively forming thermodynamically unfavorable but kinetically stable entanglements with a defined number of crossings. Covalent capture and subsequent release of the entangled intermediates produced catenanes in high yields. With this machine‐directed technique, preorganization is not required for mechanical bond formation and instead interlocking is accomplished by enforcing a double‐helix like shape through the directed motion of the molecular machine.

**Figure 1:**
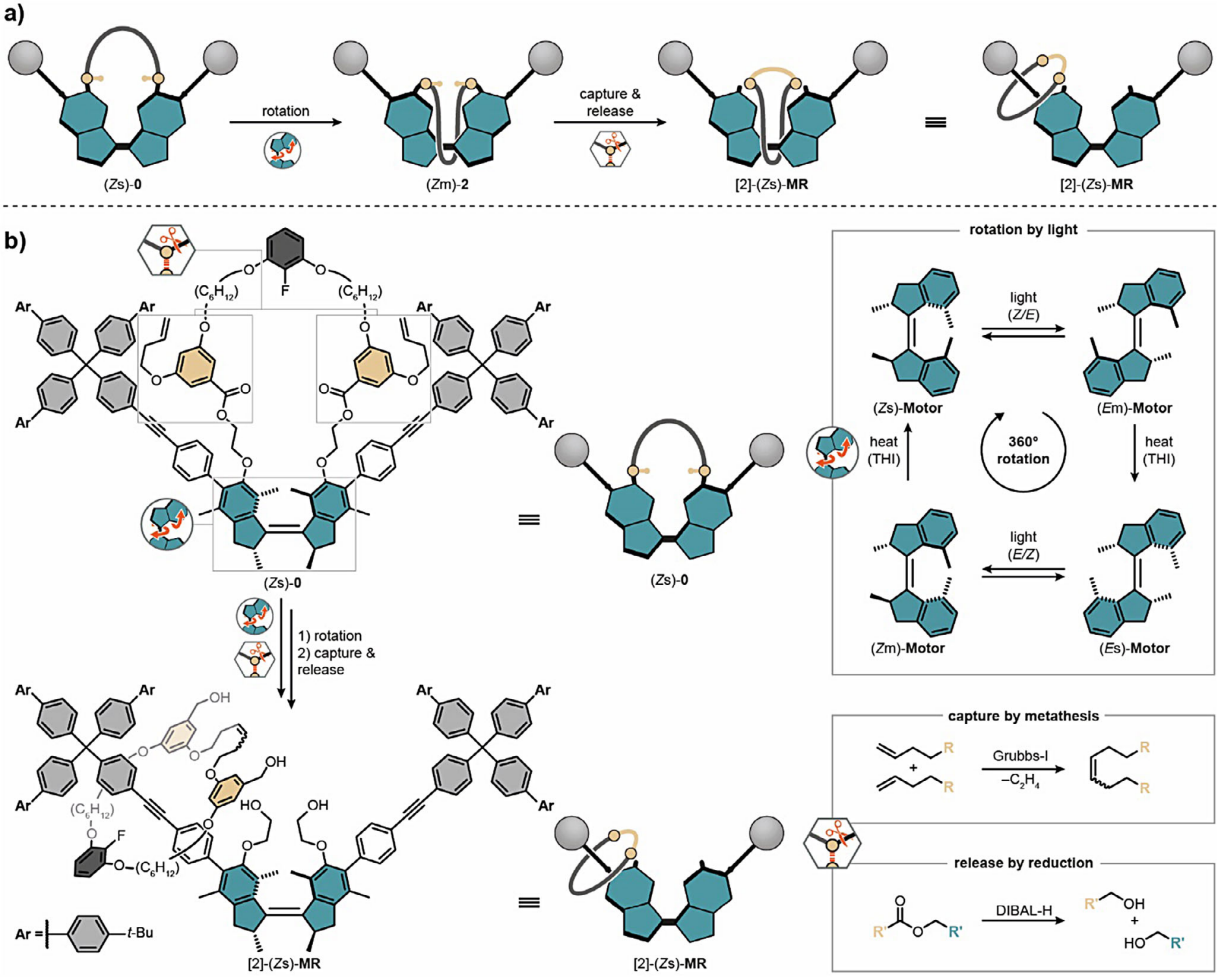
Conceptional display of the machine‐directed synthesis of a rotaxane. a) Hypothesized and schematic mechanism of the machine‐directed synthesis of rotaxane [2]‐(*Z*s)‐**MR**. Photochemical double bond isomerizations and THI lead to formation of wound machine (*Z*m)‐**2** with two crossings between the strand and axle. The wound structure can be chemically preserved, released and relaxed to form rotaxane [2]‐(*Z*s)‐**MR**. b) Molecular structure of machine (*Z*s)‐**0** and motor rotaxane [2]‐(*Z*s)‐**MR**. Machine (*Z*s)‐**0** is operated by unidirectional rotation around the central motor core, introducing a new crossing after each photochemical double bond isomerization. Chemical preservation of (*Z*m)‐**2** is realized by ring closing olefin metathesis of the terminal double bonds with a Grubbs‐I catalyst. DIBAL‐H releases the newly formed macrocycle onto the axle forming rotaxane [2]‐(*Z*s)‐**MR** after thermal relaxation of the motor core. Nomenclature of machine isomers: “(*X*y)‐**n**”, where *X* denotes the configurational isomer being either *Z* (cis) or *E* (trans), y illustrates the helicity of motor core being s for stable or m for metastable and where **n** indicates the number of crossings in the system. Only the (*R*,*R*)‐enantiomer (where s and m indicate (*P*,*P*) and (*M*,*M*) helicities, respectively) is shown for clarity although the motor unit was used as a racemic mixture.

Here, we expand the machine‐directed concept to the synthesis of rotaxanes, a distinct class of MIMs that presents a fundamentally different synthetic challenge: the prevention of macrocycle dethreading from the axle. In our new machine design, a molecular strand is attached to a unidirectional motor that is embedded within a dumbbell‐shaped axle. The strand is wound around the axle upon rotation of the central motor core, introducing the thermodynamically disfavored crossings between the two components. Covalent capture then forms a new macrocycle that is subsequently released from the motor core and trapped between the stoppers, yielding a rotaxane (Figure 1a). Unlike the catenane synthesis, where two macrocycles were intertwined, this new design benefits from a monomacrocyclic architecture that enables the use of rigid building blocks, expanding the structural scope of the machine‐directed synthesis strategy.

## Results and Discussion

Our rotaxane synthesizer (*Z*s)‐**0** (Figure 1b) is constructed from four main parts: i) a unidirectional and light‐driven molecular motor (teal) decorated with ii) two tolan‐bridged bulky stopper units (light grey) enabling winding of iii) a molecular strand (dark grey) attached via cleavable ester groups which contains iv) two terminal alkene groups (orange) that allow for covalent capture of the entangled species. A synthetic scheme of the synthesis of machine (*Z*s)‐**0** is described in Figures S1 and S2. Photochemical molecular motors, pioneered by Feringa and co‐workers,^[^ ^44^, ^45^, ^46^, ^47^ ^]^ are light‐ and heat‐responsive overcrowded alkenes. Operation of a generic, unconstrained motor starts from isomer (*Z*s)‐**Motor** by a light‐induced *Z/E* isomerization forming metastable isomer (*E*m)‐**Motor**. A thermal helix inversion (THI) then acts as a ratcheting step, resulting in the formation of isomer (*E*s)‐**Motor**, thereby completing a 180° rotation. A subsequent photochemical *E/Z* isomerization forms isomer (*Z*m)‐**Motor** which then undergoes a second THI forming initial isomer (*Z*s)‐**Motor** and completing a 360° rotation around the central double bond. Continuous irradiation with light at sufficiently high temperatures results in a progressive and unidirectional rotation.

In machine (*Z*s)‐**0**, the contra‐rotating halves of the central motor unit are mechanically constrained by the ester macrocycle and the rigid stopper units that prevent the macrocycle from skipping over the machine upon rotation. Thus, light‐driven unidirectional rotation actively winds the molecular strand around the machine, forming one crossing between axle and strand for each photochemical *E/Z* double bond isomerization performed by the central motor unit. A minimum number of two crossings—, i.e., two photochemical *E/Z* double bond isomerizations and one THI—are necessary to interlink a macrocycle with an axle and thus establish a mechanical bond. This means that our machine needs to be able to reach at least machine isomer (*Z*m)‐**2** to enable productive capture of the doubly wound ester macrocycle. Preservation of the entangled structure is achieved by ring‐closing olefin metathesis of the two terminal alkenes with a first‐generation Grubbs catalyst (Grubbs‐I). Finally, the anchoring ester groups are cleaved by reduction with diisobutylaluminium hydride (DIBAL‐H) resulting in liberation of the newly formed olefin macrocycle that is then mechanically trapped on the motor axle, yielding motor rotaxane [2]‐(*Z*s)‐**MR**.

## Mechanistic Analysis of the Winding Process

**Figure 2:**
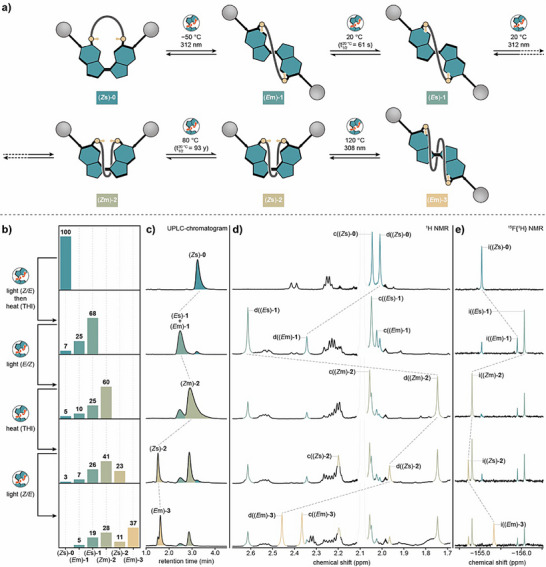
Investigation of the winding mechanism of machine (*Z*s)‐**0**. a) Schematic representation of the stepwise winding sequence of machine isomer (*Z*s)‐**0**, see Figure S6 for the molecular structures of each wound machine isomer. Half‐lives (t_1/2_) are given at 20 °C. b) Mole fraction of winding states determined by ^1^H NMR spectroscopy. The NMR error is estimated as ± 2%. c) Normalized, qualitative UPLC chromatograms of the winding sequence of (*Z*s)‐**0**. Motor structure was confirmed by coupled UV–vis spectroscopy and HRMS spectrometry (Figure S7). d) Partial ^1^H NMR (600 MHz, −10 °C, 1.5 mM, toluene‐*d*_8_) of the winding states. Xylene methyl groups c and d are color coded and were assigned using ^1^H COSY and ^1^H ROESY NMR (Figures S10–S17). Residual solvent peak is greyed out for clarity. e) ^19^F{^1^H} NMR (472 MHz, 20 °C, 1.5 mM, toluene‐*d*_8_) of the winding states.

We first investigated the winding mechanism of machine isomer (*Z*s)‐**0**, which proceeds via an alternating sequence of photochemical double bond isomerizations and THI steps (Figure 2a,b). Each step was monitored with ultra‐performance liquid chromatography coupled to high‐resolution mass spectroscopy (UPLC‐HRMS, Figures 2c and S7), ^1^H/^19^F{^1^H} nuclear magnetic resonance (NMR) (Figure 2d,e) and ultraviolet–visible (UV–vis) spectroscopy (Figures S7, S27, S31, and S32). In the ^1^H NMR spectra, the configuration of bis‐indanylidene molecular motors can be clearly followed by the chemical shift of the methyl peak in the fjord‐region (assigned as d). Generally, in (*Z*)‐configurations, this methyl group appears between 1.6–1.9 ppm while for (*E*)‐configurations the methyl group is significantly downfield‐shifted from 2.3 ppm. Additionally, UV–vis spectroscopy allows for clear distinction between stable and metastable states. The latter are characterized by their red‐shifted absorption spectra relative to the corresponding stable states.^[^ ^36^, ^43^, ^48^, ^49^ ^]^

Winding of machine isomer (*Z*s)‐**0** was initiated by photochemical *Z*/*E* double bond isomerization using UV light (*λ*_irr_  = 312 nm) at −50 °C. This resulted in the formation of isomer (*E*m)‐**1** in 93% NMR yield and introduced the first crossing between the axle and the macrocycle. Subsequent THI at 20 °C resulted in the formation of isomer (*E*s)‐**1** (Δ*G* ^‡, 20 °C ^((*E*m)‐**1 → **(*E*s)‐**1**) = 19.8 ± 0.1 kcal mol^−1^, Figure S31). Since the system is mechanically constrained, the ester macrocycle introduces mechanical resistance to the motor rotation, facilitating the reverse THI and establishing a thermal equilibrium between the isomers (*E*s)‐**1** and (*E*m)‐**1** in a ratio of 73:27.

The second crossing was introduced by a subsequent photochemical *E*/*Z* double bond isomerization using UV light (*λ*_irr_ = 312 nm) at 20 °C, affording machine isomer (*Z*m)‐**2** in 60% NMR yield. Thermal relaxation by THI at 80 °C resulted in formation of isomer (*Z*s)‐**2** (Δ*G* ^‡, 20 °C^ ((*Z*m)‐**2 **→ (*Z*s)‐**2**) = 30.0 ± 0.1 kcal mol^−1^, Figure S32) in a ratio of 35:65 ((*Z*s)‐**2**:(*Z*m)‐**2**) at thermal equilibrium, which is expected for such a mechanically constrained system. Most importantly, completing a full 360° rotation of the motor core does not result in the reformation of (*Z*s)‐**0**, further confirming active winding of the machine.^[^ ^43^ ^]^

To investigate the full winding capacity of machine (*Z*s)‐**0**, irradiation with UV light (*λ*_irr_ = 308 nm) at 120 °C was carried out. This induced the formation of a third crossing in the system yielding isomer (*E*m)‐**3** in 37% NMR yield, as confirmed by UPLC‐HRMS and NMR spectroscopy. Under these conditions, no THI toward isomer (*E*s)‐**3** was observed, indicating that the winding limit of the machine had been reached. On the contrary, continued heating at 80 °C in the absence of UV light resulted in thermal double bond back isomerization towards machine isomer (*Z*s)‐**2**. Similarly, irradiation of a mixture enriched with isomer (*E*m)‐**3** with violet light (*λ*_irr_ = 405 nm, −50 °C) resulted in photochemical back isomerization also yielding machine isomer (*Z*s)‐**2**. Subsequent heating at 80 °C then restored the thermal equilibrium between (*Z*m)‐**2** and (*Z*s)‐**2** (Figure S19).

**Figure 3:**
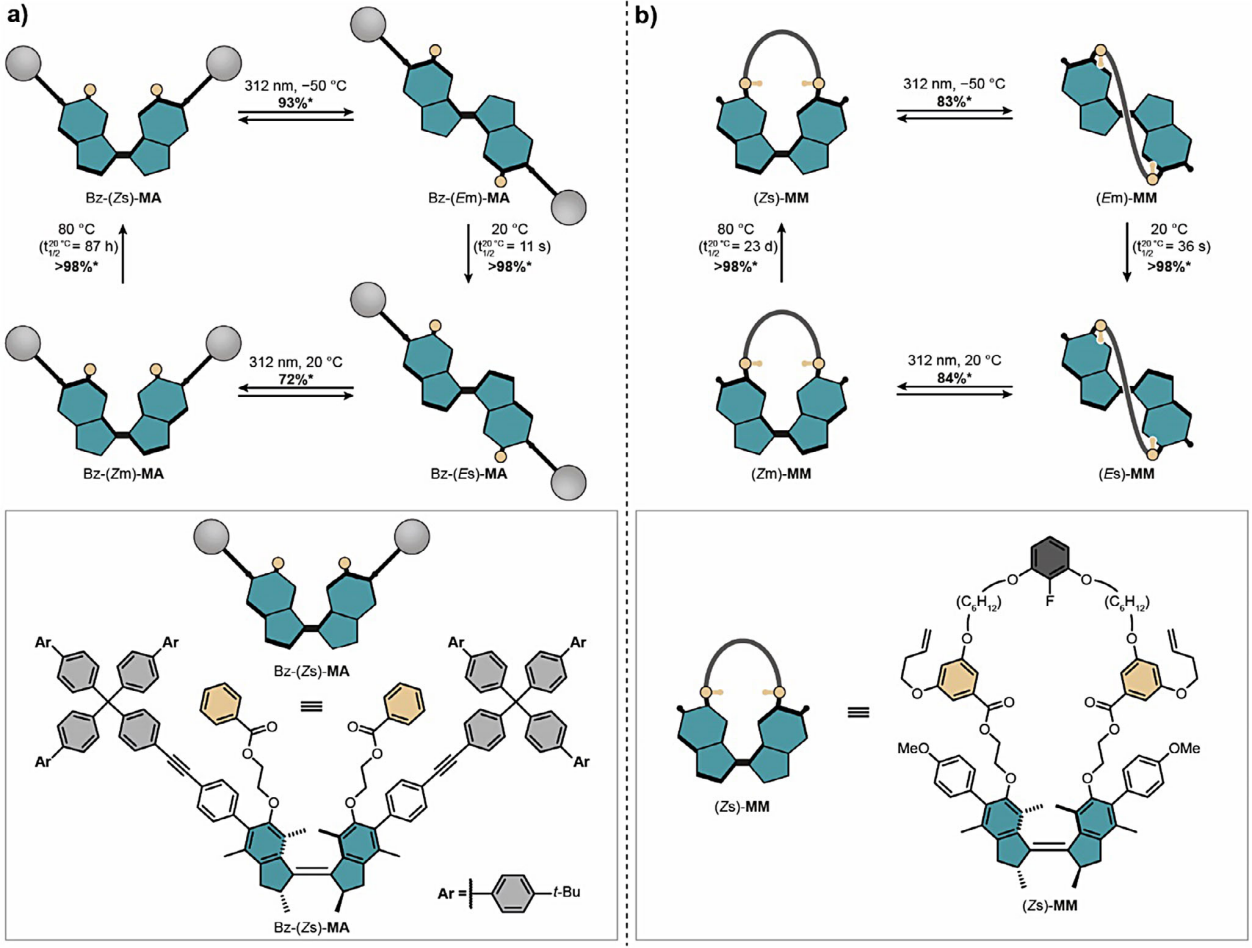
Rotational cycles of axle Bz‐(*Z*s)‐**MA** and macrocycle (*Z*s)‐**MM**. a) Schematic representation of the rotational cycle and molecular structure of axle Bz‐(*Z*s)‐**MA**. b) Schematic representation of the rotational cycle and molecular structure of macrocycle (*Z*s)‐**MM**. Both compounds perform a unidirectional rotation starting from the (*Z*s) isomer via a photochemical *Z*/*E* double bond isomerization with UV light (*λ*_irr_ = 312 nm) forming the respective (*E*m) isomer that undergoes subsequent THI to the (*E*s) isomer at 20 °C. Another photochemical *E*/*Z* double bond isomerization with UV light (*λ*_irr_ = 312 nm) forms the (*Z*m) isomer that undergoes THI at 80 °C restoring the initial (*Z*s) isomer. Half‐lives (t_1/2_) are given at 20 °C. Only the (*R*,*R*)‐enantiomer is shown for clarity although the motor unit was used as a racemic mixture. *Yields determined by NMR spectroscopy.

To further support these observations, two mechanically unconstrained control compounds were synthesized: non‐macrocyclized, stoppered motor axle Bz‐(*Z*s)‐**MA** (Figure 3a) and non‐stoppered motor macrocycle (*Z*s)‐**MM** (Figure 3b), whose synthesis is described in Figures S4 and S5. The rotational behavior of these control systems was also investigated with the help of UPLC‐HRMS (Figures S21 and S24), ^1^H/^19^F{^1^H} NMR (Figures S22, S25, and S26) and UV–vis spectroscopy (Figures S28, S29, and S33–S36). The key findings were: i) two subsequent irradiation and heating steps restored the initial *Z*s states, ii) the activation barriers for THIs were lower compared to the mechanically constrained machine (Table S9), and iii) THIs proceeded quantitatively and metastable states were not observed after thermal equilibration. This is in stark contrast to mechanically constrained machine isomer (*Z*s)‐**0** and further indicates that our machine actively winds a macrocycle around a stoppered axle by performing photochemical *E*/*Z* double bond isomerizations and THIs in a sequential manner.

## Covalent Capture of the Wound Isomers

**Figure 4:**
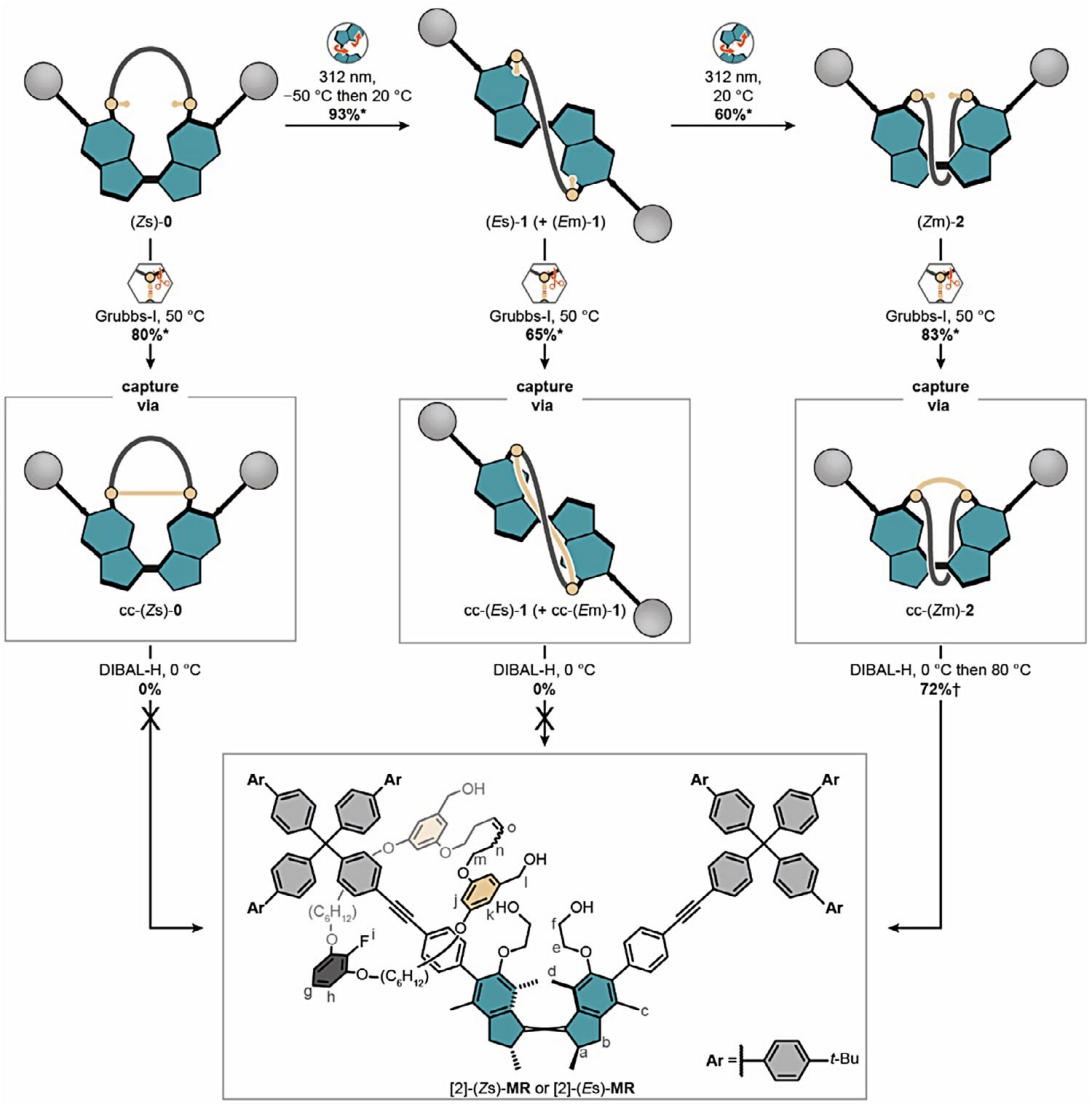
Covalent capture and reductive release of wound isomers. In all cases, covalent capture was performed with a Grubbs‐I catalyst in toluene at 50 °C and subsequent reductive release was realized with DIBAL‐H in toluene at 0 °C. Covalent capture of (*Z*s)‐**0** results in the formation of covalently captured cc‐(*Z*s)‐**0** in 80 ± 2% NMR yield. Reductive release does not result in the formation of a rotaxane. Covalent capture of (*E*s)‐**1** and (*E*m)‐**1** result in the formation of covalently captured cc‐(*E*s)‐**1** and cc‐(*E*m)‐**1** in 65 ± 2% NMR yield (only the (*E*s) configurational isomers are shown). Reductive release does not result in the formation of a rotaxane. Covalent capture of (*Z*m)‐**2** results in the formation of covalently captured cc‐(*Z*m)‐**2** in 83 ± 2% NMR yield. Reductive release forms rotaxane [2]‐(*Z*s)‐**MR** after thermal relaxation in 72% isolated yield. *Yields determined by NMR spectroscopy. †Isolated yield of rotaxane [2]‐(*Z*s)‐**MR** from (*Z*m)‐**2**.

After establishing the sequence specific winding mechanism of our machine, we investigated the outcome of the covalent capture of machine isomers (*Z*s)‐**0**, (*E*s)‐**1**/(*E*m)‐**1,** and (*Z*m)‐**2** (Figure 4). Covalent capture was performed by using a first‐generation Grubbs catalyst at 50 °C and the progress of the reaction was monitored by ^1^H/^19^F{^1^H} NMR spectroscopy and UPLC‐HRMS. Alkene metathesis from machine isomer (*Z*s)‐**0** resulted in the formation of covalently captured cc‐(*Z*s)‐**0** in 80% NMR yield (Figures S41 and S42; cc indicates covalently captured). Similarly, covalent capture of (*E*s)‐**1**/(*E*m)‐**1** resulted in the formation of cc‐(*E*s)‐**1** and cc‐(*E*m)‐**1** in 65% NMR yield (Figures S45 and S46). Finally, covalent capture of (*Z*m)‐**2** resulted in the formation of cc‐(*Z*m)‐**2** in 83% NMR yield (Figures S49 and S50).

## Release of the Captured Wound Isomers

Next, release of the newly formed olefin macrocycle **OM** was performed by treatment of each covalently captured species with DIBAL‐H followed with analysis by UPLC‐HRMS. DIBAL‐H has shown to be an excellent releasing agent as it is highly selective toward the carbonyl group and fully reduces the esters into two separate alcohols at 0 °C and above. These factors combined allow for fast and controllable reduction of the esters with no side product formation. After quantitative reductive release of cc‐(*Z*s)‐**0**, UPLC‐HRMS analysis indicated that the mixture almost exclusively consists of motor axle (*Z*s)‐**MA** along with formation of macrocycle **OM** (Supporting Figure S43). More importantly, formation of rotaxane [2]‐(*Z*s)‐**MR** was not observed due to absence of the required entanglements. After release of cc‐(*E*s)‐**1** and cc‐(*E*m)‐**1**, UPLC‐HRMS again indicated the absence of a rotaxane and showed that the mixture mainly consists of motor axle (*E*s)‐**MA** along with formation of macrocycle **OM**, further indicating that one crossing does not result in the formation of a mechanical bond. As expected, removal of the strand leads to rapid THI of motor axle (*E*m)‐**MA** into (*E*s)‐**MA** (Figure S47). Starting from machine isomer (*Z*m)‐**2**, we established the minimum number of crossings between macrocycle and axle that is required for the formation of a mechanical bond. So, reductive release of cc‐(*Z*m)‐**2** with DIBAL‐H resulted in the formation of motor rotaxane [2]‐(*Z*m)‐**MR** according to UPLC‐HRMS (Figure S51). Rotaxane [2]‐(*Z*m)‐**MR** was then thermally relaxed into [2]‐(*Z*s)‐**MR** and isolated in 72% yield from machine isomer (*Z*m)‐**2** (Figure 4).

## Structural Confirmation

**Figure 5:**
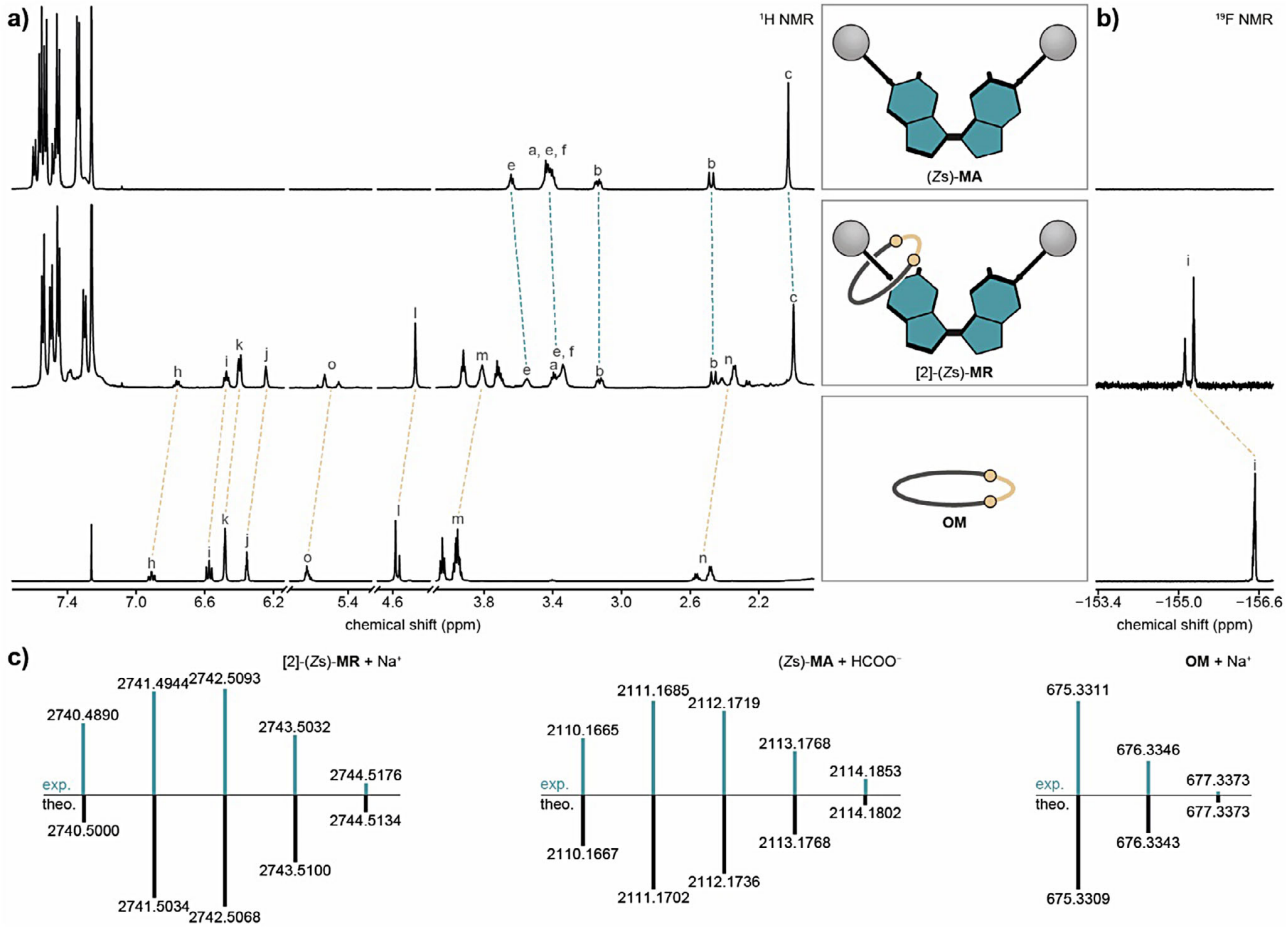
Characterization of rotaxane [2]‐(*Z*s)‐**MR**. a) Comparison of partial ^1^H NMR spectra (CDCl_3_, 25 °C, 500 or 600 MHz) of motor axle (*Z*s)‐**MA** (top), rotaxane [2]‐(*Z*s)‐**MR** (middle) and olefin macrocycle **OM** (bottom). Teal dotted lines compare signals from motor axle and orange dotted lines compare signals from olefin macrocycle. Signals were assigned using ^1^H COSY and ^1^H ROESY NMR (Figures S55 and S56). b) Comparison of ^19^F NMR spectra (CDCl_3_, 25 °C, 471 or 476 MHz) of motor axle (*Z*s)‐**MA** (top), rotaxane [2]‐(*Z*s)‐**MR** (middle) and olefin macrocycle **OM** (bottom). c) Comparison of experimental (teal) and simulated (black) isotope patterns of rotaxane [2]‐(*Z*s)‐**MR** (left), motor axle (*Z*s)‐**MA** (middle) and olefin macrocycle **OM** (right).

To investigate and confirm the structure of our interlocked molecule, we compared the ^1^H, ^19^F and ^13^C{^1^H} NMR spectra of motor rotaxane [2]‐(*Z*s)‐**MR** with that of motor axle (*Z*s)‐**MA** and olefin macrocycle **OM** (Figure 5a). In ^1^H NMR spectra, a significant shift upfield of the aromatic, benzylic and ether protons in the macrocycle of [2]‐(*Z*s)‐**MR** was observed compared to noninterlocked macrocycle **OM**, arising from the shielding effect that the two individual components exert onto each other. In ^19^F NMR spectroscopy, a significant shift downfield was observed along with a better peak separation between the *E* and *Z* isomer of the olefin macrocycle (Figure 5b). Additionally, ^1^H and ^19^F NMR spectra of a 1:1 molar mixture of motor axle (*Z*s)‐**MA** and olefin macrocycle **OM** was compared to that of rotaxane [2]‐(*Z*s)‐**MR** (Figure S52), which confirmed that the differences in chemical shifts do not arise from interactions between the two compounds in the same solution. In contrast to this, ^13^C{^1^H} NMR showed very small shifts between the rotaxane and the individual components and enabled complete assignment of all carbon signals of rotaxane [2]‐(*Z*s)‐**MR** (Table S11). Diffusion‐ordered spectroscopy (DOSY) NMR further showed that the diffusion coefficient of rotaxane [2]‐(*Z*s)‐**MR** is significantly smaller than that of olefin macrocycle **OM**. Additionally, correlations originating from both axle and macrocycle have the same diffusion coefficient in the DOSY NMR of the rotaxane, further supporting the interlocked nature of the rotaxane (Figures S57 and S58). HRMS of rotaxane [2]‐(*Z*s)‐**MR** showed excellent agreement with that of the simulated isotopic pattern along with that of the individual components (Figure 5c). Lastly, UPLC chromatography provided further evidence for the interlocked structure by showing a difference in retention time between rotaxane [2]‐(*Z*s)‐**MR**, motor axle (*Z*s)‐**MA,** and olefin macrocycle **OM** (Figure S59).

## Rotational Behavior of the Rotaxane

**Figure 6:**
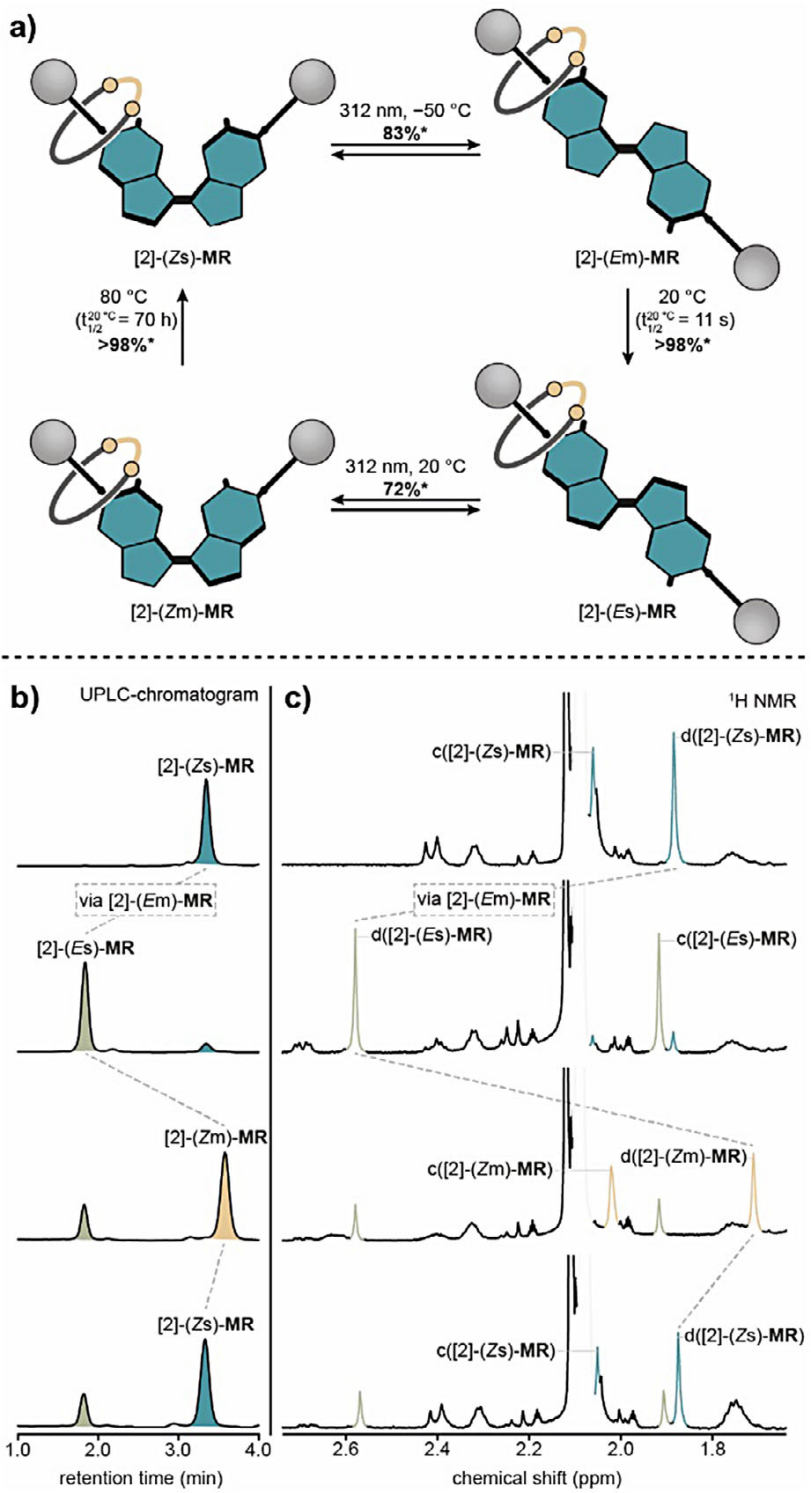
Rotational behavior of rotaxane [2]‐(*Z*s)‐**MR**. a) Schematic representation of the stepwise rotational cycle of rotaxane [2]‐(*Z*s)‐**MR**, see Figure S60 for molecular structures of each isomer. Half‐lives (t_1/2_) are given at 20 °C. b) UPLC chromatograms of the rotational cycle of rotaxane [2]‐(*Z*s)‐**MR**. Motor configurations were confirmed by UV–vis spectroscopy (Figure S61). c) Partial ^1^H NMR (600 MHz, 20 °C, 0.75 mM, toluene‐*d*_8_) of the rotation. Xylene methyl groups c and d are color coded and were assigned using ^1^H COSY and ^1^H ROESY NMR. *Yields determined by NMR spectroscopy.

Finally, we were interested if the threaded macrocycle would interfere with the rotational properties of the central motor unit in the axle of our rotaxane. Therefore, a full 360° rotation of rotaxane [2]‐(*Z*s)‐**MR** was performed by alternating photochemical double bond isomerizations and THI steps (Figure 6a). Each step was monitored with UPLC‐HRMS (Figure 6b), ^1^H/^19^F{^1^H} NMR (Figures 6c and S63) and UV–vis spectroscopy (Figures S30, S37, S38, and S61). Rotation of rotaxane [2]‐(*Z*s)‐**MR** was initiated by photochemical *Z*/*E* double bond isomerization using UV light (*λ*_irr_ = 312 nm) at −50 °C. This gave rotaxane [2]‐(*E*m)‐**MR** in 83% NMR yield. Subsequent, quantitative, THI at 20 °C resulted in the formation of rotaxane [2]‐(*E*s)‐**MR** (Δ*G* ^‡, 20 °C ^([2]‐(*E*m)‐**MR → **[2]‐(*E*s)‐**MR** = 18.4 ± 0.1 kcal mol^−1^, Figure S37). Then, another photochemical *E*/*Z* double bond isomerization was performed using UV light (*λ*_irr_ = 312 nm) at 20 °C, affording rotaxane [2]‐(*Z*m)‐**MR** in 72% NMR yield. Thermal relaxation at 80 °C restored the initial rotaxane isomer (Δ*G* ^‡, 20 °C^ ([2]‐(*Z*m)‐**MR **→ [2]‐(*Z*s)‐**MR** = 24.6 ± 0.2 kcal mol^−1^, Figure S38). This is further supported by minute changes in the chemical shifts in ^19^F{^1^H} NMR (Table S16), which is in stark contrast to machine (*Z*s)‐**0**, where the change in chemical shift is more pronounced due to the active winding of the molecular strand. This indicates that photochemical double bond isomerizations and activation barriers for each THI step are similar to those of non‐constrained motor Bz‐(*Z*s)‐**MA** (Figure 3a) and implies that the threaded macrocycle has no significant effect on the rotational performance of the central motor unit in the axle. With respect to the location of the macrocycle, it is plausible to assume, based on the above‐mentioned experimental data, that it mainly resides on the sterically least demanding tolan units, while shuttling back and forth between the two, as indicated by the fully symmetric ^1^H NMR spectra. This finding suggests that mechanical bonds could serve as robust attachment points for cargo in molecular motors and machines without interfering with their performance.

## Conclusion

Building on our machine‐directed synthesis of catenanes,^[^ ^43^ ^]^ we now demonstrate that this strategy can be generalized to produce rotaxanes. In this work, we expand the machine‐directed approach through a new molecular machine design that actively winds a molecular strand around a rigid axle core, achieving up to three crossings through successive photochemical double‐bond isomerizations and THI steps. Covalent capture and release of the doubly wound isomer exclusively yielded a rotaxane, underscoring that directed motor motion is essential to interlock two otherwise separate structures. Remarkably, the interlocked nature of the rotaxane does not affect the rotational properties of the motor core, suggesting that mechanical bonds could serve as attachment points for further functional elements without impairing motor performance. Together, these results show that the machine‐directed approach provides a versatile platform for the synthesis of diverse MIMs.

## Supporting Information

The authors have cited additional references within the Supporting Information.^[^ ^50^, ^51^, ^52^, ^53^, ^54^, ^55^, ^56^, ^57^, ^58^, ^59^ ^]^

## Conflict of Interests

The authors declare no conflict of interest.

## Supporting information



Supporting information

Supporting information

## Data Availability

The data that support the findings of this study are available in the Supporting Information of this article.
