# Supplementary material for: A Molecular Machine Directs the Synthesis of a Rotaxane
Source: Angew Chem Int Ed Engl. 2025 Nov 10;65(1):e20085. doi: 10.1002/anie.202520085 (PMC12759215; doi:10.1002/anie.202520085)
Supplement: Supplementary file 1 — Supporting information [file ANIE-65-e20085-s001.pdf]

Supporting Information

A Molecular Machine Directs the Synthesis of a Rotaxane

Robert Kluifhooft, Tommy Wachsmuth, Bob Barthel, Mira Müller, Ann-Kathrin Rückert, Michael Kathan*

Humboldt-Universität zu Berlin, Institut für Chemie

Brook-Taylor Strasse 2, 12489 Berlin

*e-mail: Michael.peter.kathan@hu-berlin.de

Contents

1 General Remarks....................4

2 Synthesis....................6

2.1 Synthesis of Motor Axle Core (*Z*s)-MA....................6

2.2 Synthesis of the Tether and Macrocyclization towards Machine (*Z*s)-0....................21

2.3 Synthesis of Rotaxane [2]-(*Z*s)-MR....................30

2.4 Synthesis of Bz-(*Z*s)-MA, OM and (*Z*s)-MM....................33

3 Winding and Rotation Experiments....................44

3.1 Winding of Mechanism of Machine Isomer (*Z*s)-0....................45

3.2 Rotational Cycle of Control Bz-(*Z*s)-MA....................55

3.3 Rotational Cycle of (*Z*s)-MM....................58

4 Switching Behavior....................62

4.1 Switching Behavior of Machine Isomer (*Z*s)-0....................62

4.2 Switching Behavior of Bz-(*Z*s)-MA....................62

4.3 Switching Behavior of (*Z*s)-MM....................63

4.4 Switching Behavior of rotaxane [2]-(*Z*s)-MR....................63

5 Eyring Analysis....................64

5.1 Thermal Helix Inversion of Machine Isomer (*E*m)-1 to (*E*s)-1....................66

5.2 Thermal Helix Inversion of Machine Isomer (*Z*m)-2 to (*Z*s)-2....................67

5.3 Thermal Helix Inversion of Bz-(*E*m)-MA to Bz-(*E*s)-MA....................68

5.4 Thermal Helix Inversion of Bz-(Zm)-MA to Bz-(*Z*s)-MA....................69

5.5 Thermal Helix Inversion of Control (*E*m)-MM to (*E*s)-MM....................70

5.6 Thermal Helix Inversion of Control (*Z*m)-MM to (*Z*s)-MM....................71

5.7 Thermal Helix Inversion of Rotaxane [2]-(*E*m)-MR to [2]-(*E*s)-MR....................72

5.8 Thermal Helix Inversion of Rotaxane [2]-(*Z*m)-MR to [2]-(*Z*s)-MR....................73

6 Covalent Capture and Release Experiments....................74

6.1 Covalent Capture and Release from Machine Isomer (*Z*s)-0....................75

6.2 Covalent Capture and Release from Machine Isomers (*E*m)-1 and (*E*s)-1 78

6.3 Covalent Capture and Release from Machine Isomer (*Z*m)-2 81

7 Characterization of Rotaxane [2]-(*Z*s)-MR 84

7.1 NMR Spectroscopy Comparisons 84

7.2 DOSY Experiments of Rotaxane [2]-(*Z*s)-MR and Olefin Macrocycle OM 88

7.3 UPLC-HRMS 90

7.4 Rotation of rotaxane [2]-(*Z*s)-MR 91

8. Single Crystal X-Ray Crystallography 95

8.1 Crystal Structure of S7 95

8.2 Crystal Structure of S10 96

9 References 97

10 Spectra Appendix 97

1 General Remarks

Reagents were purchased as reagent grade and used without further purification, unless otherwise specified. All non-aq. reactions were performed in oven-dried glassware and under Ar atmosphere. Automated Medium Pressure Column Chromatography (MPLC) was performed on a Teledyne ISCO CombiFlashRf 300 system with 200 mL/min max flow, 200 psi, equipped with integrated ELSD and 200–800 nm UV-vis variable wavelength detector. Thin layer chromatography (TLC) was conducted on aluminium sheets coated with SiO_2-60 F_{254} obtained from Merck; visualization with a UV lamp (254 or 365 nm). Nuclear magnetic resonance (NMR) spectra were recorded using a Bruker Avance II 500 (500 MHz for ^{1}H, 126 MHz for ^{13}C and 471 MHz for ^{19}F) at 25 °C and are reported as follows: chemical shift (δ) in ppm (multiplicity, coupling constant J in Hz, number of protons; assignment). The residual deuterated solvent was used as the internal reference. For ^{1}H NMR: $CDCl_3$ δ_H = 7.26 ppm, toluene-d_8 δ_{CH3} = 2.09 ppm, CD_2Cl_2 δ_H = 5.32 ppm, DMSO-d_6 δ_H = 2.5 ppm and for ^{13}C NMR $CDCl_3$ δ_C = 77.16 ppm, toluene-d_8 δ_C = 137.48, 128.87, 127.96, 125.13, 20.43 ppm, CD_2Cl_2 δ_C = 53.84 ppm, DMSO-d_6 δ_C = 39.52 ppm. In ^{19}F NMR, fluorobenzene (δ_F = –113.15 ppm) and 4-fluoroanisole (δ_F = –124.73 ppm) were used as internal references. The resonance multiplicity is described as s (singlet), d (doublet), t (triplet), q (quartet), p (pentet), m (multiplet), and br (broad). Ultra-performance liquid chromatography (UPLC) was performed on an Acquity H-class UPLC equipped with an Acquity QDa detector in combination with a PDA eλ diode array detector using reversed phase columns (Acquity UPLC BEH phenyl cyclohexyl (1.7 μm 100 × 2.10 mm) or Acquity UPLC BEH C18 (1.7 μm 50 × 2.10 mm)). High resolution mass spectrometry (HRMS) was performed on a Xevo G3 QTof MS, measuring in either positive or negative mode. Single-crystal X-ray data were measured with a BRUKER D8 VENTURE area detector with Mo-Kα radiation (λ = 0.71073 Å). Multi-scan absorption corrections implemented in SADABS[1] were applied to the data. The structures were solved by intrinsic phasing method (SHELXT-2013)[2] and refined by full matrix least square procedures based on F2 with all measured reflections (SHELXL-2014)[3] in the graphical user interface (SHELXLe)[4] with anisotropic temperature factors for all non-hydrogen atoms. All hydrogen atoms were added geometrically and refined by using a riding reference. Ultraviolet-Visible (UV-vis) absorbance spectroscopy was performed on Agilent Cary 60 instruments connected to a cryostat from Unisoku Scientific Instruments (temperature accuracy ± 0.1 K) in 10 × 10 mm quartz cuvettes with 3 mL volume. Weighing of small quantities was performed on a Sartorius ME5 analytical microbalance.

Recycling gel permeation chromatography size exclusion chromatography (*r*GPC-SEC) was performed on a Shimadzu instrument using trichloromethane with 0.6% ethanol as eluent. The *r*GPC was equipped with two JAIGEL 2HR 40 and one JAIGEL 3HR 40 columns. The following compounds were prepared according to literature **S1**,[5] **S3**[6] and **S5**.[7] All ^{1}H, ^{13}C and ^{19}F NMR along with ^{1}H, ^{13}C HSQC, ^{1}H, ^{13}C HMBC, ^{1}H COSY and ^{1}H ROESY NMR spectra of novel synthesized compounds can be found in the spectra appendix.

2 Synthesis

2.1 Synthesis of Motor Axle Core (*Z*s)-MA

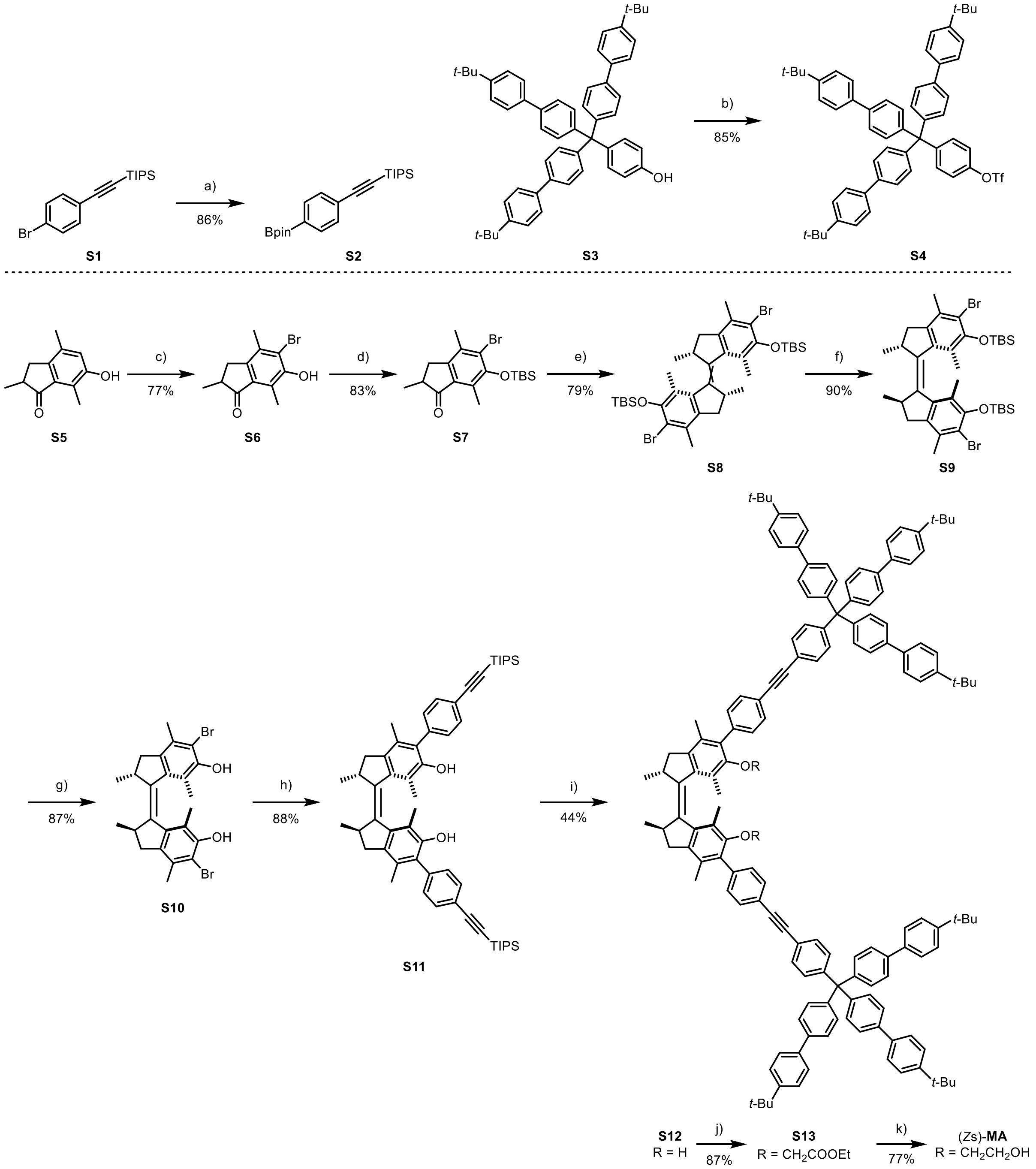

Supporting Figure S1 | ** Synthesis of motor axle (*Z*s)-MA**. a) B_2pin_2, KOAc, $Pd(dppf)Cl_2$·DCM, 1,4-dioxane, 80 °C, 18 h, 86%; b) Tf_2O, pyridine, CH_2Cl_2, 25 °C, 1.5 h, 85%; c) NBS, NaI, $MeNO_2$, 25 °C, 1 h, 77%; d) TBSCl, imidazole, DMF, 25 °C, 18 h, 83%; e) $TiCl_4$, Zn, 1,4-dioxane, 66 °C, 4 d, 79%; f) nonane, 150 °C, 3 d, 90%; g) TBAF, THF, 25 °C, 10 min, 87%; h) **S2**, $Pd(OAc)_2$, SPhos, K_2CO_3, 1,4-dioxane/H_2O, 100 °C, 18 h, 88%; i) 1: TBAF, THF. 2: **S4**, $Pd(dppf)Cl_2$·DCM, CuI, 1,4-dioxane/DBU, 100 °C, 18 h, 44%; j) ethyl 2-bromoacetate, K_2CO_3, DMF, 80 °C, 18 h, 87%; k) DIBAL-H, THF, 25 °C 1 h, 77%. Only the (*R*,*R*)-enantiomer is shown for clarity.

Triisopropyl((4-(4,4,5,5-tetramethyl-1,3,2-dioxaborolan-2-yl)phenyl)ethynyl)silane (S2)

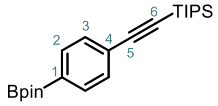

S2
$C_{23}H_{37}BO_2Si$
Mw = 384.44 g/mol

This synthesis was based on an adapted procedure from literature.[8] A suspension of **S1** (1.18 g, 3.50 mmol, 1.0 equiv.), Pd(dppf)Cl_2·DCM (313 mg, 0.383 mmol, 11 mol%), B_2pin_2 (977 mg, 3.85 mmol, 1.1 equiv.) and anhydrous KOAc (710 mg, 10,4 mmol, 3.0 equiv.) in 1,4-dioxane (15 mL) was stirred at 80 °C for 18 h. The reaction was subsequently cooled to room temperature and filtered over silica using EtOAc, concentrated under reduced pressure and purified by MPLC (SiO_2, cHex/EtOAc gradient 100:0 ⟶ 80:20), affording the title compound **S2** (1.15 g, 86%) as an orange solid. An analytically pure sample could be obtained by MPLC (SiO_2, PE/Et_2O gradient 100:0 ⟶ 88:12), obtaining **S2** as white crystals.

^{1}H NMR (500 MHz, $CDCl_3$, 25 °C) δ = 7.74 (d, J = 8.1 Hz, 2H, H−C^2), 7.47 (d, J = 8.2 Hz, 2H, H−C^3), 1.35 (s, 12H, CH_3−pin), 1.14 (s, 21H, *i*-Pr−Si) ppm.

^{13}C{^{1}H} NMR (126 MHz, $CDCl_3$, 25 °C) δ = 134.6 (C^2), 131.3 (C^3), 129.0 (C^1), 126.4 (C^4), 107.3 (C^5), 92.2 (C^6), 84.1 (C−pin), 25.0 (CH_3−pin), 18.8 (CH_3−*i*-Pr), 11.5 (CH−*i*-Pr) ppm. Note: low intensity C^1 signal missing due to quadrupolar relaxation induced by the boron nuclei. The signal could be assigned using ^{1}H, ^{13}C HMBC.

HR-ESI-TOF-MS (ESI+) *m/z* calculated for $C_{23}H_{38}BO_2Si^+$ ($[M+H]^+$) 385.2729, found 385.2715. Mass error: 3.6 ppm.

4-(Tris(4'-(*tert*-butyl)-[1,1'-biphenyl]-4-yl)methyl)phenyl trifluoromethanesulfonate (S4)

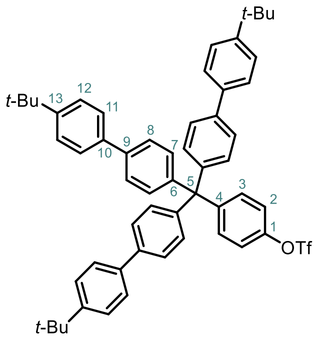

S4

$C_{56}H_{55}F_3O_3S$

Mw = 865.09 g/mol

This synthesis was based on an adapted procedure from literature.[9] Compound **S3** (2.1 g, 2.9 mmol, 1.0 equiv.) was suspended in anhydrous CH_2Cl_2 (20 mL). Anhydrous pyridine (0.46 mL, 0.45 g, 5.7 mmol, 2.0 equiv.) was added, turning the suspension into a solution, which was then cooled to 0 °C using an ice bath. Triflic anhydride (0.72 mL, 1.2 g, 4.3 mmol, 1.5 equiv.) was added dropwise to the solution, and stirred at 25 °C for 2 h. The reaction was quenched by dropwise addition of water and extracted with CH_2Cl_2. The combined organic phases were dried over anhydrous $MgSO_4$, concentrated under reduced pressure and purified by MPLC (SiO_2, CH_2Cl_2/cHex gradient 100:0 ⟶ 95:5), affording the title compound **S4** (2.1 g, 85%) as a white solid.

^{1}H NMR (500 MHz, CD_2Cl_2, 25 °C) δ = 7.59 (d, J = 8.5 Hz, 12H, H−C^{8+11}), 7.52 (d, J = 9.0 Hz, 2H, H−C^3), 7.50 (d, J = 8.5 Hz, 6H, H−C^{12}), 7.40 (d, J = 8.5 Hz, 6H, H−C^7), 7.26 (d, J = 9.0 Hz, 2H, H−C^2), 1.38 (s, 27H, CH_3−*t*-Bu) ppm.

^{13}C{^{1}H} NMR (126 MHz, CD_2Cl_2, 25 °C) δ = 151.0 (C^{13}), 148.1 (C^1), 148.0 (C^4), 145.4 (C^6), 139.2 (C^9), 137.8 (C^{10}), 133.3 (C^3), 131.7 (C^7), 126.9 (C^{11}), 126.7 (C^8), 126.2 (C^{12}), 120.8 (C^2), 119.2 (q, J_{C-F} = 320.7 Hz, CF_3), 64.5 (C^5), 34.9 (C−*t*-Bu), 31.5 (CH_3−*t*-Bu) ppm.

^{19}F NMR (471 MHz, CD_2Cl_2, 25 °C) δ = −72.7 (s, 3F, CF_3−S) ppm.

HR-ESI-TOF-MS (ESI+) *m/z* calculated for $C_{56}H_{55}F_3KO_3S^+$ ($[M+K]^+$) 903.3456, found 903.3474. Mass error: 2.0 ppm.

5-Bromo-6-hydroxy-2,4,7-trimethyl-2,3-dihydro-1H-inden-1-one (S6)

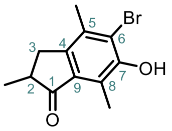

S6

$C_{12}H_{13}BrO_2$

Mw = 269.13 g/mol

This synthesis was based on an adapted procedure from literature.[7] Under ambient conditions and exclusion of light, **S5** (8.59 g, 45.1 mmol, 1.0 equiv.) and NBS (20.1 g, 113 mmol, 2.5 equiv.) were dissolved in $MeNO_2$ (80 mL). The reaction mixture was stirred at 25 °C for 30 min, after which NaI (27.1 g, 181 mmol, 4.0 equiv.) was added. The resulting mixture was stirred at 25 °C for 1 h and subsequently quenched with a sat. aq. $Na_2S_2O_3$ solution. The product was extracted with CH_2Cl_2, combined organic layers were washed with sat. aq. $NaHCO_3$, brine, subsequently dried over anhydrous $MgSO_4$ and concentrated under reduced pressure. The crude product was purified by column chromatography (SiO_2, PE/EtOAc 80:20), affording the title compound **S6** (10.9 g, 77%) as a white solid. Note: alfa bromination occurs as a side reaction, which is then cleaved by NaI. However, prolonged stirring after addition of NaI results in the debromination of the aromatic bromide as well.

^{1}H NMR (500 MHz, $CDCl_3$, 25 °C) δ = 5.71 (s, 1H, HO−C^{7}), 3.22 (dd, J = 16.6, 8.0 Hz, 1H, H−C^{3}), 2.71 – 2.63 (m, 1H, H−C^{2}), 2.59 (s, 3H, CH_3−C^{8}), 2.52 (dd, J = 16.6, 4.0 Hz, 1H, H−C^{3}), 2.36 (s, 3H, CH_3−C^{5}), 1.29 (d, J = 7.4 Hz, 3H, CH_3−C^{2}) ppm .

^{13}C{^{1}H} NMR (126 MHz, $CDCl_3$, 25 °C) δ = 210.3 (C^{1}), 149.8 (C^{7}), 144.8 (C^{4}), 133.3 (C^{9}), 132.4 (C^{6}), 122.1 (C^{8}), 120.7 (C^{5}), 42.7 (C^{2}), 33.6 (C^{3}), 18.7 (CH_3−C^{5}), 16.7 (CH_3−C^{2}), 10.8 (CH_3−C^{8}) ppm.

HR-ESI-TOF-MS (ESI+) *m/z* calculated for $C_{12}H_{14}BrO_2^+$ ($[M+H]^+$) 269.0172, found 269.0177. Mass error: 1.9 ppm.

5-Bromo-6-((*tert*-butyldimethylsilyl)oxy)-2,4,7-trimethyl-2,3-dihydro-1H-inden-1-one (S7)

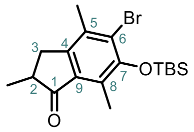

S7
$C_{18}H_{27}BrO_2Si$
Mw = 383.40 g/mol

A solution of **S6** (927 mg, 3.44 mmol, 1.0 equiv.), TBSCl (781 mg, 5.18 mmol, 1.5 equiv.), and imidazole (285 mg, 4.19 mmol, 1.2 equiv.) was stirred in DMF (10 mL) at 25 °C for 18 h, then poured into water and extracted with Et_2O. Combined phases were washed with aq. 5 m/m% LiCl, brine, dried over anhydrous $MgSO_4$ and concentrated under reduced pressure. The crude product was purified by MPLC (SiO_2, cHex/EtOAc gradient 100:0 ⟶ 90:10), affording the title compound **S7** (1.10 g, 83%) as a colorless oil that crystalized into a white solid. Unambiguous structural proof was provided by single-crystal X-ray diffraction (Supplementary Section 8.1).

^{1}H NMR (500 MHz, $CDCl_3$, 25 °C) δ = 3.23 (dd, J = 16.8, 8.0 Hz, 1H, H−C^{3}), 2.71 – 2.61 (m, 1H, H−C^{2}), 2.53 (dd, J = 16.7, 4.0 Hz 1H, H−C^{3}), 2.52 (s, 3H, CH_3−C^{8}), 2.36 (s, 3H, CH_3−C^{5}), 1.29 (d, J = 7.4 Hz, 3H, CH_3−C^{2}), 1.05 (s, 9H, CH_3−*t*-Bu), 0.25 (s, 6H, CH_3−Si) ppm.

^{13}C{^{1}H} NMR (126 MHz, $CDCl_3$, 25 °C) δ = 210.3 (C^{1}), 150.6 (C^{7}), 146.5 (C^{4}), 133.7 (C^{6}), 133.3 (C^{9}), 126.9 (C^{8}), 126.6 (C^{5}), 42.7 (C^{2}), 33.8 (C^{3}), 26.4 (CH_3−*t*-Bu), 19.1 (CH_3−C^{5}), 19.0 (C−*t*-Bu), 16.7 (CH_3−C^{2}), 13.0 (CH_3−C^{8}), −2.3 (CH_3−Si) ppm.

HR-ESI-TOF-MS (ESI+) *m/z* calculated for $C_{18}H_{28}BrO_2Si^+$ ($[M+H]^+$) 383.1036, found 383.1040. Mass error: 1.0 ppm.

(*E*/*Z*)-((5,5'-Dibromo-2,2',4,4',7,7'-hexamethyl-2,2',3,3'-tetrahydro-[1,1'-biindenylidene]-6,6'-diyl)bis(oxy))bis(*tert*-butyldimethylsilane) (S8)

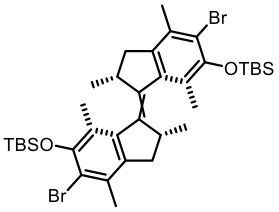

S8
$C_{36}H_{54}Br_2O_2Si_2$
Mw = 734.79 g/mol

This synthesis was based on an adapted procedure from literature.[10] A suspension of zinc powder (3.42 g, 52.3 mmol, 4.0 equiv.) in 1,4-dioxane (60 mL) was stirred while $TiCl_4$ (3.0 mL, 5.19 g, 27.4 mmol, 2.1 equiv.) was added dropwise. The mixture was heated at reflux for 2 h and subsequently cooled to 66 °C, after which indanone **S7** (5.00 g, 13.0 mmol, 1.0 equiv.) was added. The reaction mixture was then continued to be stirred at 66 °C for 4 d. The reaction was quenched by addition of sat. aq. NH_4Cl and extracted with EtOAc. Combined organic phases were washed with sat. aq. $NaHCO_3$, brine, dried over anhydrous $MgSO_4$ and concentrated under reduced pressure. The crude product was purified by MPLC (SiO_2, cHex), affording the title compound **S8** (*E*/Z mixture, 3.77 g, 79%) as a white solid. The product was used for the next step without further purification. Note: the McMurry reaction towards **S8** is highly diastereoselective and results in a mixture of the (*S*,*S*)- and (*R*,*R*)-enantiomer. Enantiomeric resolution was not performed, however the (*R*,*R*)-enantiomer will be depicted as a standard to avoid confusion with a theoretically possible (*R*,*S*)-meso compound.[7]

HR-ESI-TOF-MS (ESI+) *m/z* calculated for $C_{36}H_{54}Br_2O_2Si_2^{\cdot+}$ ($[M]^{\cdot+}$) 732.2024, found. 732.2021. Mass error: 0.4 ppm.

(*Z*)-((5,5'-Dibromo-2,2',4,4',7,7'-hexamethyl-2,2',3,3'-tetrahydro-[1,1'-biindenylidene]-6,6'-diyl)bis(oxy))bis(*tert*-butyldimethylsilane) (S9)

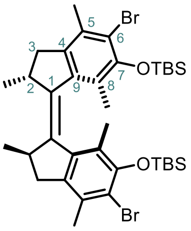

S9

$C_{36}H_{54}Br_2O_2Si_2$

Mw = 734.79 g/mol

This synthesis was based on an adapted literature procedure.[10] Motor mixture **S8** (1.63 g, 2.22 mmol) was suspended in nonane (20 mL), degassed using freeze pump thaw (5 cycles) and subsequently heated at 150 °C for 3 d. The reaction was concentrated under reduced pressure and purified by column chromatography (SiO_2, PE), affording the title compound **S9** (1.47 g, 90%) as a white solid.

^{1}H NMR (500 MHz, $CDCl_3$, 25 °C) δ = 3.30 (p, J = 6.6 Hz, 2H, H−C^2), 3.10 (dd, J = 14.6, 6.2 Hz, 2H, H−C^3), 2.44 (d, J = 14.6 Hz, 2H, H−C^3), 2.33 (s, 6H, CH_3−C^5), 1.44 (s, 6H, CH_3−C^8), 1.06 (d, J = 6.7 Hz, 6H, CH_3−C^2), 1.01 (s, 18H, CH_3−*t*-Bu), 0.23 (s, 6H, CH_3−Si), 0.22 (s, 6H, CH_3−Si) ppm.

^{13}C{^{1}H} NMR (126 MHz, $CDCl_3$, 25 °C) δ = 149.5 (C^7), 141.2 (C^9), 141.0 (C^1), 138.0 (C^5), 131.7 (C^4), 125.4 (C^8), 117.4 (C^6), 42.0 (C^2), 39.7 (C^3), 26.6 (CH_3−*t*-Bu), 20.4 (CH_3−C^2), 20.1 (CH_3−C^8), 19.1 (C−*t*-Bu), 16.8 (CH_3−C^5), −1.4 (CH_3−Si), −2.5 (CH_3−Si) ppm.

HR-ESI-TOF-MS (ESI+) *m/z* calculated for $C_{36}H_{55}Br_2O_2Si_2^{\cdot+}$ ([M]$^{\cdot+}$) 732.2024, found. 732.2024. Mass error: 0.0 ppm.

(*Z*)-5,5'-Dibromo-2,2',4,4',7,7'-hexamethyl-2,2',3,3'-tetrahydro-[1,1'-biindenylidene]-6,6'-diol (S10)

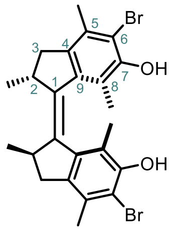

S10
$C_{24}H_{26}Br_2O_2$
Mw = 506.27 g/mol

A solution of **S9** (1.47 g, 2.00 mmol, 1.0 equiv.) in THF (20 mL) was cooled to 0 °C, after which TBAF (1 M in THF, 6.00 mL, 6.00 mmol, 3.0 equiv.) was added. The reaction was heated to 25 °C and left to stir for 10 min, subsequently quenched by addition of sat. aq. NH_4Cl and extracted with EtOAc. Combined organic phases were washed with brine, dried over anhydrous $MgSO_4$ and concentrated under reduced pressure. The crude product was purified by column chromatography (SiO_2, PE/EtOAc 10:1), affording the title compound **S10** (844 mg, 87%) as an off white solid. Unambiguous structural proof was provided by single-crystal X-ray diffraction (Supplementary Section 8.2).

^{1}H NMR (500 MHz, $CDCl_3$, 25 °C) δ = 5.50 (s, 2H, HO−C^7), 3.32 (p, J = 6.7 Hz, 2H, H−C^2), 3.11 (dd, J = 14.5, 6.3 Hz, 2H, H−C^3), 2.43 (d, J = 14.6 Hz, 2H, H−C^3), 2.33 (s, 6H, CH_3−C^5), 1.47 (d, J = 0.7 Hz, 6H, CH_3−C^8), 1.07 (d, J = 6.7 Hz, 6H, CH_3−C^2) ppm.

^{13}C{^{1}H} NMR (126 MHz, $CDCl_3$,25 °C) δ = 148.8 (C^7), 141.0 (C^9), 140.8 (C^1), 136.7 (C^4), 130.3 (C^5), 120.6 (C^8), 112.2 (C^6), 41.8 (C^2), 39.3 (C^3), 20.5 (CH_3−C^2), 19.5 (CH_3−C^5), 15.3 (CH_3−C^8) ppm.

HR-ESI-TOF-MS (ESI−) *m/z* calculated for $C_{24}H_{25}Br_2O_2^-$ ([M−H]$^-$) 503.0227, found 503.0222. Mass error: 1.0 ppm.

(*Z*)-2,2',4,4',7,7'-Hexamethyl-5,5'-bis(4-((tri*iso*propylsilyl)ethynyl)phenyl)-2,2',3,3'-tetrahydro-[1,1'-biindenylidene]-6,6'-diol (S11)

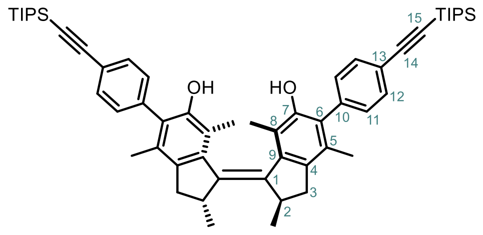

S11

$C_{58}H_{76}O_2Si_2$

Mw = 861.39 g/mol

A suspension of **S10** (200 mg, 0.395 mmol, 1.0 equiv.), **S2** (402 mg, 1.05 mmol, 2.6 equiv.), $Pd(OAc)_2$ (13 mg, 0.058 mmol, 15 mol%), SPhos (108 mg, 0.263 mmol, 65 mol%) and K_2CO_3 (218 mg, 1.58 mmol, 4.0 equiv.) in degassed 1,4-dioxane/water mixture (5:1, 4 mL) was heated at 100 °C for 18 h. The reaction was quenched by addition of sat. aq. NH_4Cl and extracted with EtOAc. The combined organic phases were washed with brine, dried over anhydrous $MgSO_4$ and concentrated under reduced pressure. The crude product was purified by MPLC (SiO_2, PE/Et_2O gradient 100:0 ⟶ 85:15), affording the title compound **S11** (298 mg, 88%) as a white solid.

^{1}H NMR (500 MHz, $CDCl_3$, 25 °C) δ = 7.62 (d, J = 8.6 Hz, 4H, H−C^{12}), 7.27 (b, 4H, H−C^{11}), 4.53 (s, 2H, HO−C^{7}), 3.38 (p, J = 6.7 Hz, 2H, H−C^{2}), 3.11 (dd, J = 14.6, 6.2 Hz, 2H, H−C^{3}), 2.43 (d, J = 14.5 Hz, 2H, H−C^{3}), 1.97 (s, 6H, CH_3−C^{5}), 1.53 (s, 6H, CH_3−C^{8}), 1.16 (s, 42H, *i*-Pr−Si), 1.14 (d, J = 3.6 Hz, 6H, CH_3−C^{2}) ppm.

^{13}C{^{1}H} NMR (126 MHz, $CDCl_3$, 25 °C) δ = 149.2 (C^{7}), 141.7 (C^{9}), 141.2 (C^{1}), 137.0 (C^{10}), 136.2 (C^{4}), 133.0 (C^{12}), 130.8 (C^{11}), 130.7 (C$^{11'}$), 128.8 (C^{5}), 125.7 (C^{6}), 123.1 (C^{13}), 119.6 (C^{8}), 106.8 (C^{14}), 91.5 (C^{15}), 41.7 (C^{2}), 39.0 (C^{3}), 20.7 (CH_3−C^{2}), 18.8 (CH_3−*i*-Pr), 16.8 (CH_3−C^{5}), 14.7 (CH_3−C^{8}), 11.5 (CH−*i*-Pr) ppm. Note: C^{11} and C^{12} are split into two low intensity and/or broad signal(s) due to diastereotopicity and conformational isomerism.

HR-ESI-TOF-MS (ESI+) *m/z* calculated for $C_{58}H_{77}O_2Si_2^+$ ($[M+H]^+$) 861.5457, found 861.5441. Mass error: 1.9 ppm.

(*Z*)-2,2',4,4',7,7'-Hexamethyl-5,5'-bis(4-((4-(tris(4'-(*tert*-butyl)-[1,1'-biphenyl]-4-yl) methyl)phenyl)ethynyl)phenyl)-2,2',3,3'-tetrahydro-[1,1'-biindenylidene]-6,6'-diol (S12)

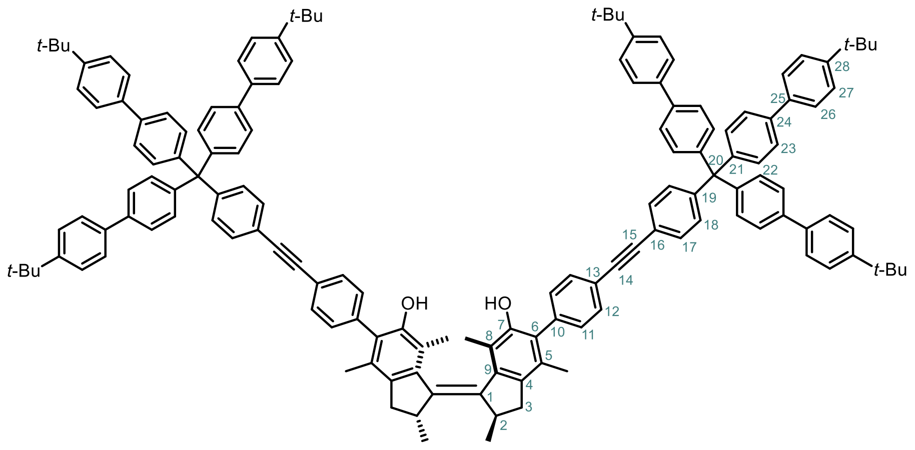

S12
$C_{150}H_{144}O_2$
Mw = 1978.75 g/mol

A solution of **S11** (400 mg, 0.465 mmol, 1.0 equiv.) in THF (4 mL) was reacted with TBAF (1 M in THF) until full conversion to the deprotected motor was observed by UPLC-MS or TLC. The resulting solution was then concentrated, after which **S4** (630 mg, 0.729 mmol, 2.5 equiv.), Pd(dppf)Cl_2·DCM (48 mg, 0.058 mmol, 20 mol%) and CuI (6 mg, 0.030 mmol, 10 mol%) were added to the vessel and suspended in a degassed 1,4-dioxane/DBU mixture (10:1, 8 mL). The mixture was heated at 100 °C for 18 h, after which it was quenched with aq. 2 M HCl was and extracted with EtOAc. Combined phases were washed with brine, dried over anhydrous $MgSO_4$ and concentrated under reduced pressure. The crude product was purified by MPLC (SiO_2, PE/CH_2Cl_2 gradient 100:0 ⟶ 70:30), affording the title compound **S12** (262 mg, 44%) as an orange solid. Note: fluoride is able to quench the triflate resulting in a failed reaction when too much TBAF is added.

^{1}H NMR (500 MHz, $CDCl_3$, 25 °C) δ = 7.67 (d, J = 8.7 Hz, 4H, H–C^{12}), 7.57 (d, J = 8.5 Hz, 12H, H–C^{26}), 7.54 (d, J = 8.6 Hz, 12H, H–C^{23}), 7.50 (d, J = 8.5 Hz, 4H, H–C^{17}), 7.47 (d, J = 8.4 Hz, 12H, H–C^{27}), 7.35 (d, J = 8.5 Hz, 16H, H–C^{18+22}), 7.33 – 7.29 (b, 4H, H−C^{11}), 4.58 (s, 2H, HO–C^{7}), 3.39 (p, J = 6.6 Hz, 2H, H−C^{2}), 3.12 (dd, J = 14.5, 6.2 Hz, 2H, H−C^{3}), 2.45 (d, J = 14.5 Hz, 2H, H−C^{3}), 2.00 (s, 6H, CH_3−C^{5}), 1.55 (s, 6H, CH_3−C^{8}), 1.37 (s, 54H, CH_3−*t*-Bu), 1.16 (d, J = 6.6 Hz, 6H, CH_3−C^{2}) ppm.

^{13}C{^{1}H} NMR (126 MHz, $CDCl_3$, 25 °C) δ = 150.4 (C^{28}), 149.3 (C^{7}), 147.5 (C^{19}), 145.3 (C^{21}), 141.7 (C^{9}), 141.2 (C^{1}), 138.8 (C^{24}), 137.8 (C^{25}), 136.9 (C^{10}), 136.2 (C^{4}), 132.5 (C^{12}), 131.6 (C^{22}), 131.4 (C^{18}), 131.0 (C^{17}), 130.8 (C^{11}), 128.8 (C^{5}), 126.8 (C^{26}), 126.2 (C^{23}), 125.9 (C^{27}),

125.7 (C^{6}), 122.9 (C^{13}), 120.9 (C^{16}), 119.6 (C^{8}), 90.2 (C^{15}), 89.3 (C^{14}), 64.5 (C^{20}), 41.6 (C^{2}), 39.0 (C^{3}), 34.7 (C−*t*-Bu), 31.5 (CH_3−*t*-Bu), 20.8 (CH_3−C^{2}), 16.8 (CH_3−C^{5}), 14.7 (CH_3−C^{8}) ppm. Note: C^{11} and C^{12} are split into two low intensity and/or broad signal(s) due to diastereotopicity and conformational isomerism.

HR-ESI-TOF-MS (ESI−) *m/z* calculated for $C_{151}H_{145}O_4^-$ ([M+HCOO]$^-$) 2022.1148, found 2022.1078. Mass error: 3.5 ppm.

(*Z*)-Diethyl 2,2'-((2,2',4,4',7,7'-hexamethyl-5,5'-bis(4-((4-(tris(4'-(*tert*-butyl)-[1,1'-biphenyl]-4-yl)methyl)phenyl)ethynyl)phenyl)-2,2',3,3'-tetrahydro-[1,1'-biindenylidene]-6,6'-diyl)bis(oxy))diacetate (S13)

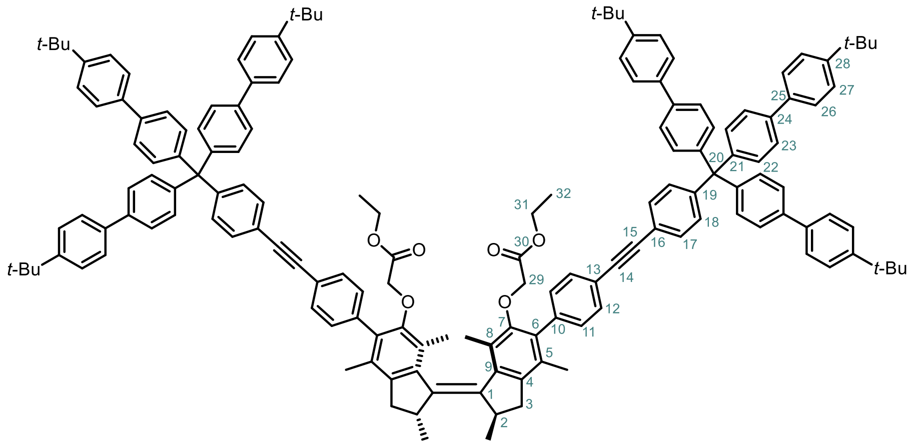

S13

$C_{158}H_{156}O_6$

Mw = 2150.93 g/mol

A suspension of **S12** (100 mg, 0.0505 mmol, 1.0 equiv.) and K_2CO_3 (37 mg, 0.268 mmol, 5.3 equiv.) in DMF (4 mL) was stirred at 80 °C, while a solution of ethyl 2-bromoacetate (0.11 mL, 170 mg, 1.0 mmol, 20 equiv.) in DMF (4 mL) was added dropwise over the course of 18 h. The reaction was quenched by addition of sat. aq. NH_4Cl (25 mL) and the product was extracted using CH_2Cl_2. Combined organic phases were dried over anhydrous $MgSO_4$ and concentrated under reduced pressure. The crude product was purified by MPLC (SiO_2, PE/CH_2Cl_2 gradient 100:0⟶ 50:50), affording the title compound **S13** (94 mg, 87%) as a yellow solid.

^{1}H NMR (500 MHz, $CDCl_3$, 25 °C) δ = 7.61 (d, J = 8.7 Hz, 4H, H–C^{12}), 7.58 (d, J = 8.6 Hz, 12H, H–C^{26}), 7.56 (d, J = 8.6 Hz, 12H, H–C^{23}), 7.51 (d, J = 8.6 Hz, 4H, H–C^{17}), 7.48 (d, J = 8.6 Hz, 12H, H–C^{27}), 7.38 (d, J = 8.6 Hz, 16H, H–C^{18+22}), 7.42 − 7.28 (b, 4H, H–C^{11}), 4.13 – 4.05 (m, 4H, H–C^{31}), 4.05 (d, J = 15.6 Hz, 2H, H–C^{29}), 3.92 (d, J = 15.4 Hz, 2H, H–C^{29}), 3.42 (p, J = 6.6 Hz, 2H, H–C^{2}), 3.15 (dd, J = 14.8, 6.1 Hz, 2H, H–C^{3}), 2.49 (d, J = 14.8 Hz, 2H, H–C^{3}), 2.05 (s, 6H, CH_3–C^{5}), 1.64 (s, 6H, CH_3–C^{8}), 1.39 (s, 54H, CH_3–*t*-Bu), 1.20 (t, J = 7.1 Hz, 6H, H–C^{32}), 1.15 (d, J = 6.6 Hz, 6H, CH_3–C^{2}) ppm.

^{13}C{^{1}H} NMR(500 MHz, $CDCl_3$, 25 °C) δ = 168.9 (C^{30}), 153.1 (C^{7}), 150.4 (C^{28}), 147.3 (C^{19}), 145.3 (C^{21}), 141.8 (C^{4}), 141.4 (C^{1}), 141.0 (C^{9}), 138.7 (C^{24}), 138.2 (C^{10}), 137.8 (C^{25}), 133.1 (C^{6}), 131.6 (C^{22}), 131.5 (C^{12}), 131.3 (C^{18}), 131.0 (C^{17}), 130.7 (C^{11}), 130.5 (C$^{11'}$), 130.0 (C^{5}), 126.8 (C^{26}), 126.4 (C^{8}), 126.2 (C^{23}), 125.8 (C^{27}), 121.8 (C^{13}), 121.1 (C^{16}), 89.7 (C^{14}), 89.6 (C^{15}), 69.5 (C^{29}), 64.5 (C^{20}), 61.0 (C^{31}), 42.1 (C^{2}), 39.3 (C^{3}), 34.7 (C–*t*-Bu), 31.5 (CH_3–*t*-Bu), 20.7

(CH_3–C^2), 16.8 (CH_3–C^5), 15.0 (CH_3–C^8), 14.2 (C^{32}) ppm. Note: C^{11} and C^{12} are split into two low intensity and/or broad signal(s) due to diastereotopicity and conformational isomerism.

HR-ESI-TOF-MS (ESI+) *m/z* calculated for $C_{158}H_{156}NaO_6^+$ ($[M+Na]^+$) 2172.1794, found 2172.1743. Mass error: 2.3 ppm.

Motor Axle (*Zs*)-MA

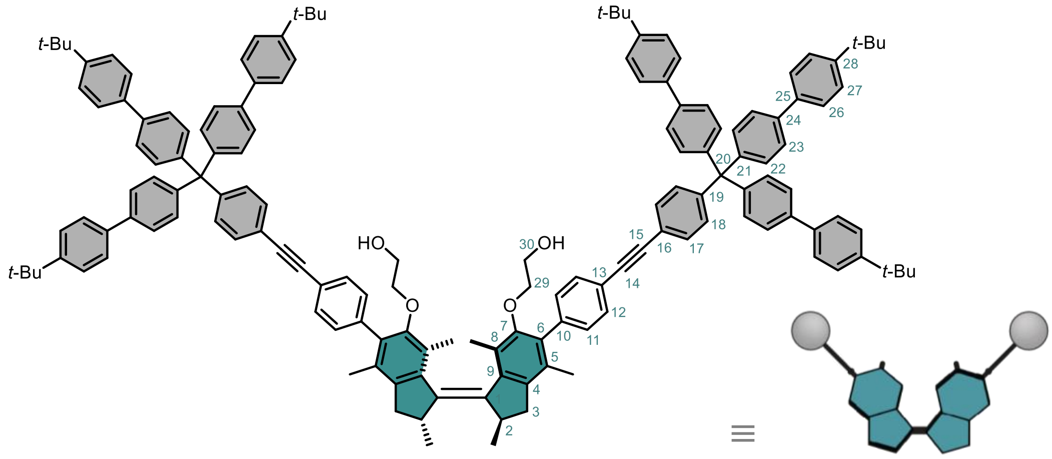

(*Zs*)-**MA**
$C_{154}H_{152}O_4$
Mw = 2066.85 g/mol

A solution of **S13** (136 mg, 63 μmol, 1.0 equiv.) in THF (2 mL) was reacted with DIBAL-H (1.2 M in toluene, 0.53 mL, 0.63 mmol, 10 equiv.) at 0 °C. The mixture was subsequently stirred at 25 °C for 1 h and carefully quenched by addition of water and aq. 2 M HCl and extracted using CH_2Cl_2. The combined phases were washed with brine, dried over $MgSO_4$ and concentrated under reduced pressure. The crude product was purified by MPLC (SiO_2, PE/EtOAc gradient 100:0 ⟶ 70:30), affording motor axle (*Zs*)-**MA** (100 mg, 77%) as an off white solid.

^{1}H NMR (500 MHz, $CDCl_3$, 25 °C) δ = 7.61 (d, *J* = 8.7 Hz, 4H, H–C^{12}), 7.57 (d, *J* = 8.6 Hz, 12H, H–C^{26}), 7.54 (d, *J* = 8.6 Hz, 12H, H–C^{23}), 7.50 (d, *J* = 8.6 Hz, 4H, H–C^{17}), 7.47 (d, *J* = 8.5 Hz, 12H, C^{27}), 7.39 – 7.29 (b, 4H, H–C^{11}), 7.36 (d, *J* = 8.6 Hz, 16H, H–C^{18+22}), 3.70 – 3.62 (m, 2H, H–C^{29}), 3.49 – 3.37 (m, 8H, H–C$^{2+29+30}$), 3.15 (dd, *J* = 14.9, 6.2 Hz, 2H, H–C^{3}), 2.49 (d, *J* = 14.8 Hz, 2H, H–C^{3}), 2.04 (s, 6H, CH_3–C^{5}), 1.60 (s, 6H, CH_3–C^{8}), 1.37 (s, 54H, CH_3–*t*-Bu), 1.16 (d, *J* = 6.6 Hz, 6H, CH_3–C^{2}) ppm.

^{13}C{^{1}H} NMR (500 MHz, $CDCl_3$, 25 °C) δ = 153.0 (C^{7}), 150.4 (C^{28}), 147.3 (C^{19}), 145.3 (C^{21}), 142.0 (C^{9}), 141.4 (C^{1}), 140.6 (C^{4}), 138.7 (C^{24}), 138.6 (C^{10}), 137.8 (C^{25}), 133.4 (C^{6}), 131.6 (C^{22}), 131.4 (C^{18}), 131.3 (C^{12}), 131.0 (C^{17}), 130.8 (C^{11}), 130.4 (C$^{11'}$), 130.0 (C^{5}), 126.8 (C^{26}), 126.2 (C^{23}), 126.1 (C^{8}), 125.8 (C^{27}), 121.9 (C^{13}), 121.0 (C^{16}), 89.7 (C^{15}), 89.6 (C^{14}), 73.5 (C^{29}), 64.5 (C^{20}), 62.0 (C^{30}), 41.9 (C^{2}), 39.2 (C^{3}), 34.7 (C–*t*-Bu), 31.5 (CH_3–*t*-Bu), 20.8 (CH_3–C^{2}), 16.9 (CH_3–C^{8}), 15.2 (CH_3–C^{5}) ppm. Note: C^{11} and C$^{11'}$ are split into two low intensity and/or broad signal(s) due to diastereotopicity and conformational isomerism.

HR-ESI-TOF-MS (ESI+) *m/z* calculated for $C_{154}H_{153}O_4^+$ ($[M+H]^+$), 2066.1763 found 2066.1799. Mass error: 1.7 ppm.

2.2 Synthesis of the Tether and Macrocyclization towards Machine (*Z*s)-0

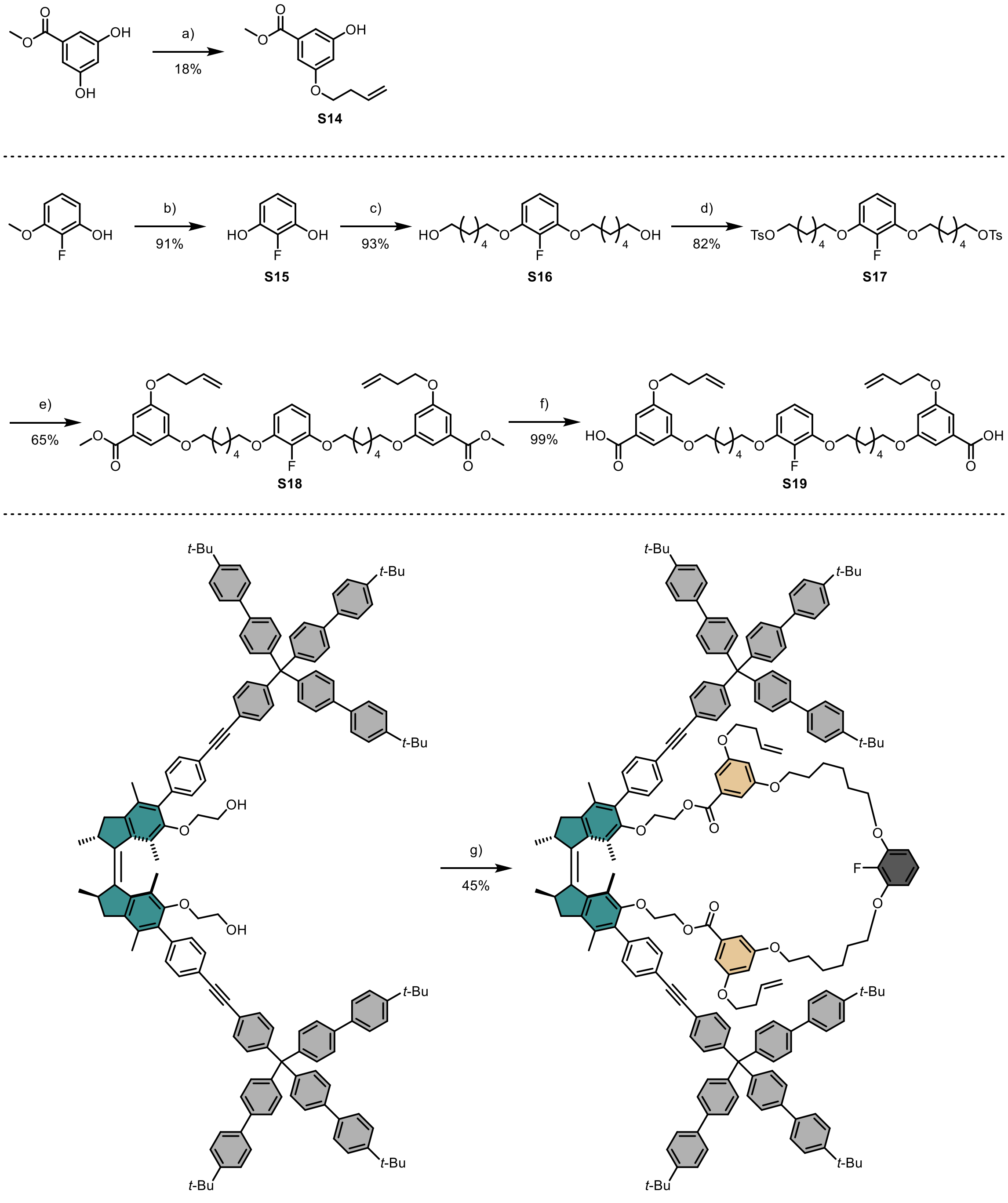

(*Z*s)-**MA** (*Z*s)-**0**

Supporting Figure S2 | Synthesis of the linker **S14** (top), tether **S19** (middle) and machine (*Z*s)-**0** (bottom). a) 4-Bromo-1-butene, K_2CO_3, DMF, 70 °C, 18 h, 18%; b) $AlCl_3$, toluene, 100 °C, 1.5 h, 91%; c) $BrCH_2(CH_2)_4CH_2OH$, K_2CO_3, DMF, 80 °C, 18 h, 93%; d) TsCl, Et_3N, DMAP, CH_2Cl_2, 25 °C, 3 h, 82%; e) **S14**, K_2CO_3, DMF, 80 °C, 18 h, 65%; f) LiOH, H_2O, THF, 60 °C, 18 h, 99%; g) **S19**, EDAC·HCl, DMAP, CH_2Cl_2, 25 °C, 3 d, 45%. Only the (*R*,*R*)-enantiomer is shown for clarity.

Methyl 3-(but-3-en-1-yloxy)-5-hydroxybenzoate (S14)

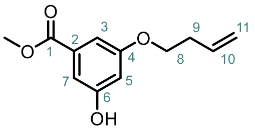

S14

$C_{12}H_{14}O_4$

Mw = 222.24 g/mol

This synthesis was based on an adapted procedure from literature.[11] A suspension of 4-bromo-1-butene (7.6 mL, 10 g, 75 mmol, 1.0 equiv.), methyl 3,5-dihydroxybenzoate (13.3 g, 79.3 mmol, 1.05 equiv.) and K_2CO_3 (22.5 g, 162 mmol, 2.2 equiv.) in DMF (150 mL) was heated at 80 °C for 18 h. The reaction was quenched by addition of sat. aq. NH_4Cl and extracted with EtOAc. Combined organic phases were washed with aq. 5 m/m% LiCl, brine, dried over anhydrous $MgSO_4$ and concentrated under reduced pressure. The crude product was purified by column chromatography (SiO_2, PE/EtOAc 70:30), affording the title compound **S14** (3.0 g, 18%) as a colorless oil that crystalized into a white solid.

^{1}H NMR (500 MHz, $CDCl_3$, 25 °C) δ = 7.20 (dd, J = 2.3, 1.3 Hz, 1H, H−C^7), 7.13 (dd, J = 2.3, 1.3 Hz, 1H, H−C^3), 6.64 (t, J = 2.3 Hz, 1H, H−C^5), 6.49 – 6.39 (b, 1H, HO−C^6), 5.88 (ddt, J = 17.0, 10.3, 6.7 Hz, 1H, H−C^{10}), 5.16 (dq, J = 17.2, 1.6 Hz, 1H, H−C^{11}), 5.10 (dq, J = 10.3, 1.4 Hz, 1H, H−C^{11}), 4.00 (t, J = 6.7 Hz, 2H, H−C^8), 3.90 (s, 3H, MeO−C^1), 2.52 (qt, J = 6.7, 1.4 Hz, 2H, H−C^9) ppm.

^{13}C{^{1}H} NMR (126 MHz, $CDCl_3$, 25 °C) δ = 167.5 (C^1), 160.3 (C^4), 157.1 (C^6), 134.3 (C^{10}), 131.9 (C^2), 117.3 (C^{11}), 109.5 (C^7), 107.8 (C^3), 107.4 (C^5), 67.6 (C^8), 52.6 (MeO−C^1), 33.6 (C^9) ppm.

HR-ESI-TOF-MS (ESI+) *m/z* calculated for $C_{12}H_{15}O_4^+$ ($[M+H]^+$) 223.0965, found 223.0972. Mass error: 3.1 ppm.

2-Fluorobenzene-1,3-diol (S15)

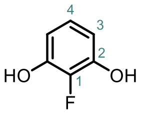

S15
$C_6H_5FO_2$
Mw = 128.10 g/mol

This synthesis was previously reported in literature.[12] A solution of 2-fluoro-3-methoxyphenol (2.08 g, 14.6 mmol, 1.0 equiv.) in anhydrous toluene (20 mL) was reacted with $AlCl_3$ (5.85 g, 43.9 mmol, 3.0 equiv.). The resulting suspension was heated at 100 °C for 1.5 h, subsequently cooled to room temperature and carefully quenched by addition of water. The suspension was transferred to a separation funnel containing aq. 2 M HCl and the product was extracted with EtOAc. Combined organic phases were washed with brine, dried over anhydrous $MgSO_4$ and concentrated under reduced pressure. The crude product was purified by column chromatography (SiO_2, cHex/EtOAc gradient 100:0 ⟶ 60:40), affording the title compound **S15** (1.70 g, 91%) as a beige solid.

^{1}H NMR (500 MHz, DMSO-d_6, 25 °C) δ = 9.58 (s, 2H, HO−C^2), 6.71 (td, J = 8.2, 1.9 Hz, 1H, H−C^4), 6.37 (t, J = 7.9 Hz, 2H, H−C^3) ppm.

^{13}C{^{1}H} NMR (126 MHz, DMSO-d_6, 25 °C) δ = 146.1 (d, J_{C-F} = 9.8 Hz, C^2), 141.0 (d, J_{C-F} = 236.1 Hz, C^1), 123.4 (d, J_{C-F} = 4.7 Hz, C^4), 108.1 (C^3) ppm.

^{19}F NMR (471 MHz, DMSO-d_6, 25 °C) δ = −161.0 (td, J = 7.7, 2.0 Hz, 1F, F−C^1) ppm.

HR-ESI-TOF-MS (ESI−) *m/z* calculated for $C_6H_4FO_2^-$ ($[M-H]^-$) 127.0201, found 127.0197. Mass error: 3.1 ppm.

6,6'-((2-Fluoro-1,3-phenylene)bis(oxy))bis(hexan-1-ol) (S16)

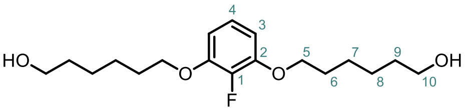

S16
$C_{18}H_{29}FO_4$
Mw = 328.42 g/mol

A suspension of **S15** (425 mg, 3.32 mmol, 1.0 equiv.), K_2CO_3 (4.60 g, 33.2 mmol, 6.0 equiv.) and 6-bromohexan-1-ol (1.1 mL, 1.5 g, 2.4 equiv.) in MeCN was heated at 80 °C for 18 h. The reaction was quenched with water and extracted with EtOAc. Combined organic phases were washed with brine, dried over anhydrous $MgSO_4$ and concentrated under reduced pressure. The crude product was purified by MPLC (SiO_2, CH_2Cl_2/MeOH gradient 100:0 ⟶ 90:10), affording the title compound **S16** (1.01 g, 93%) as a colorless oil, that crystalized into a white solid.

^{1}H NMR (500 MHz, DMSO-d_6, 25 °C) δ = 6.98 (td, J = 8.5, 2.1 Hz, 1H, H−C^4), 6.72 (dd, J = 8.5, 7.4 Hz, 2H, H−C^3), 4.35 (t, J = 5.2 Hz, 2H, HO−C^{10}), 3.99 (t, J = 6.5 Hz, 4H, H−C^5), 3.39 (td, J = 6.4, 5.2 Hz, 4H, H−C^{10}), 1.69 (dt, J = 8.2, 6.6 Hz, 4H, H−C^6), 1.47 – 1.28 (m, 12H, H−C^{7+8+9}) ppm.

^{13}C{^{1}H} NMR (126 MHz, DMSO-d_6, 25 °C) δ = 147.5 (d, $J_{C–F}$ = 7.9 Hz, C^2), 142.0 (d, $J_{C–F}$ = 241.8 Hz, C^1), 123.5 (d, $J_{C–F}$ = 5.1 Hz, C^4), 107.0 (C^3), 68.8 (C^5), 60.6 (C^{10}), 32.5 (C^9), 28.7 (C^6), 25.3 (C^7), 25.2 (C^8) ppm.

^{19}F NMR (471 MHz, DMSO-d_6, 25 °C) δ = −158.2 (td, J = 7.4, 2.3 Hz, 1F, F−C^1) ppm.

HR-ESI-TOF-MS (ESI+) m/z calculated for $C_{18}H_{30}FO_4^+$ ([M+H]$^+$) 329.2123, found 329.2130. Mass error: 2.1 ppm.

((2-Fluoro-1,3-phenylene)bis(oxy))bis(hexane-6,1-diyl) bis(4-methylbenzenesulfonate) (S17)

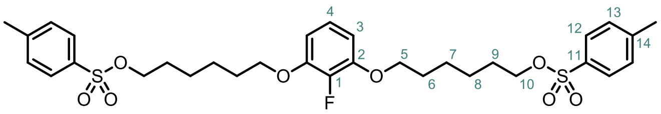

S17
$C_{32}H_{41}FO_8S_2$
Mw = 636.79 g/mol

A solution of **S16** (776 mg, 2.37 mmol, 1.0 equiv.), *p*-toluenesulfonyl chloride (1.13 g, 5.92 mmol, 2.5 equiv.), Et_3N (2.6 mL, 1.9 g, 18 mmol, 8.0 equiv.) and DMAP (87 mg, 0.71 mmol, 30 mol%) in CH_2Cl_2 (15 mL) was stirred at 25 °C for 2 h. The reaction was quenched with water and extracted with CH_2Cl_2. The combined organic phases were dried over anhydrous $MgSO_4$ and concentrated under reduced pressure. The crude product was purified by MPLC (SiO_2, cHex/EtOAc gradient 100:0 ⟶ 70:30), affording the title compound **S17** (1.24 g, 82%) as a colorless oil that crystalized into a white solid.

^{1}H NMR (500 MHz, $CDCl_3$, 25 °C) δ = 7.78 (d, J = 8.3 Hz, 4H, H−C^{12}), 7.33 (d, J = 8.2 Hz, 4H, H−C^{13}), 6.91 (td, J = 8.4, 2.1 Hz, 1H, H−C^{4}), 6.55 (dd, J = 8.4, 7.2 Hz, 2H, H−C^{3}), 4.02 (t, J = 6.4 Hz, 4H, H−C^{10}), 3.96 (t, J = 6.4 Hz, 4H, H−C^{5}), 2.43 (s, 6H, CH_3−C^{14}) 1.74 (p, J = 6.5 Hz, 4H, H−C^{6}), 1.66 (p, J = 6.5 Hz, 4H, H−C^{9}), 1.46 – 1.32 (m, 8H, H−C^{7+8}) ppm.

^{13}C{^{1}H} NMR (126 MHz, $CDCl_3$, 25 °C) δ = 148.1 (d, J_{C-F} = 8.4 Hz, C^{2}), 144.8 (C^{14}), 143.4 (d, J_{C-F} = 244.1 Hz, C^{1}), 133.2 (C^{11}), 129.9 (C^{13}), 128.0 (C^{12}), 123.1 (d, J_{C-F} = 5.3 Hz, C^{4}), 107.5 (C^{3}), 70.6 (C^{10}), 69.4 (C^{5}), 29.1 (C^{6}), 28.8 (C^{9}), 25.4 (C^{7}), 25.2 (C^{8}), 21.7 (CH_3−C^{14}) ppm.

^{19}F NMR (471 MHz, $CDCl_3$, 25 °C) δ = −157.2 (td, J = 7.2, 2.3 Hz, F−C^{1}) ppm.

HR-ESI-TOF-MS (ESI+) *m/z* calculated for $C_{32}H_{42}FO_8S_2^+$ ($[M+H]^+$) 637.2300, found 637.2303. Mass error: 0.5 ppm.

Dimethyl 5,5'-((((2-fluoro-1,3-phenylene)bis(oxy))bis(hexane-6,1-diyl))bis(oxy))bis(3-(but-3-en-1-yloxy)benzoate) (S18)

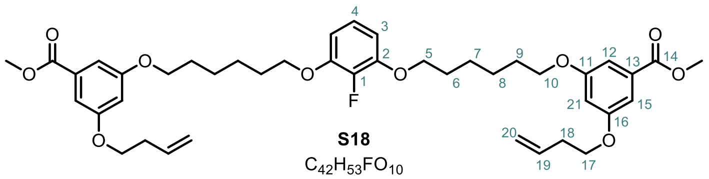

S18
$C_{42}H_{53}FO_{10}$
Mw = 736.86 g/mol

A suspension of **S17** (510 mg, 0.801 mmol, 1.0 equiv.), **S14** (460 mg, 2.07 mmol, 2.6 equiv.) and K_2CO_3 (1.17 g, 5.08 mmol, 6.3 equiv.) in DMF (10 mL) was heated at 100 °C for 18 h. The reaction was quenched with water and sat. aq. NH_4Cl and extracted with Et_2O. Combined organic phases were washed with aq. 5 m/m% LiCl, brine, dried over anhydrous $MgSO_4$ and concentrated under reduced pressure. The crude product was purified by MPLC (SiO_2, PE/Et_2O gradient 100:0 ⟶ 70:30), affording the title compound **S18** (384 mg, 65%) as a colorless oil.

^{1}H NMR (500 MHz, $CDCl_3$, 25 °C) δ 7.16 (d, J = 2.3 Hz, 4H, H−$C^{12,15}$), 6.92 (td, J = 8.4, 2.2 Hz, 1H, H−C^{4}), 6.64 (t, J = 2.4 Hz, 2H, H−C^{21}), 6.58 (dd, J = 8.4, 7.1 Hz, 2H, H−C^{3}), 5.89 (ddt, J = 17.0, 10.2, 6.7 Hz, 2H, H−C^{19}), 5.17 (dq, J = 17.2, 1.6 Hz, 2H, H−C^{20}), 5.11 (dq, J = 10.2, 1.3 Hz, 2H, H−C^{20}), 4.03 (t, J = 6.6 Hz, 8H, H−$C^{5,17}$), 3.98 (t, J = 6.4 Hz, 4H, H−C^{10}), 3.89 (s, 6H, MeO−C^{14}), 2.54 (qt, J = 6.7, 1.4 Hz, 4H, H−C^{18}), 1.88 – 1.77 (m, 8H, H−C^{6+9}), 1.57 – 1.50 (m, 8H, H−$C^{7,8}$) ppm.

^{13}C{^{1}H} NMR (126 MHz, $CDCl_3$, 25 °C) δ 167.1 (C^{14}), 160.2 (C^{11}), 160.1 (C^{16}), 148.3 (d, $J_{C\text{–}F}$ = 8.4 Hz, C^{2}), 143.6 (d, $J_{C\text{–}F}$ = 244.4 Hz, C^{1}), 134.4 (C^{19}), 132.0 (C^{13}), 123.1 (d, $J_{C\text{–}F}$ = 5.3 Hz, C^{4}), 117.3 (C^{20}), 108.0, 107.8, 107.6 (C^{3}), 106.8 (C^{21}), 69.7 (C^{5}), 68.3 (C^{10}), 67.6 (C^{17}), 52.3 (MeO−C^{14}), 33.7 (C^{18}), 29.3, 29.2, 25.9, 25.9 ppm. Some carbon atoms could not be assigned due to signal overlap.

^{19}F NMR (471 MHz, $CDCl_3$, 25 °C) δ = –157.0 (td, J = 7.1, 2.2 Hz, F−C^{1}) ppm.

HR-ESI-TOF-MS (ESI+) *m/z* calculated for $C_{42}H_{54}FO_{10}^+$ ($[M+H]^+$) 737.3696, found 737.3689. Mass error: 0.9 ppm.

5,5'-((((2-Fluoro-1,3-phenylene)bis(oxy))bis(hexane-6,1-diyl))bis(oxy))bis(3-(but-3-en-1-yloxy)benzoic acid) (S19)

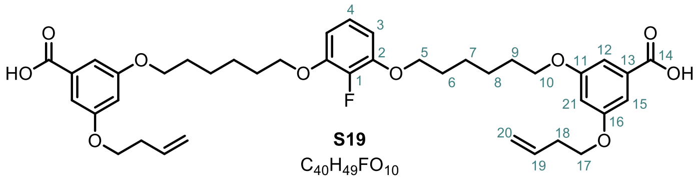

S19

$C_{40}H_{49}FO_{10}$

Mw = 708.81 g/mol

Under ambient conditions, **S18** (366 mg, 0.497 mmol, 1.0 equiv.) was dissolved in THF (5 mL). Then, aq. 3 M LiOH (5 mL) was added and the mixture was vigorously stirred at 60 °C for 18 h. The reaction was quenched by addition of aq. 2 M HCl and the product was extracted with EtOAc. The combined organic phases were washed with brine, dried over anhydrous $MgSO_4$ and concentrated under reduced pressure. The crude product was purified by MPLC (SiO_2, CH_2Cl_2/MeOH gradient 100:0 ⟶ 95:5), affording the title compound **S19** as an off-white solid after lyophilization form benzene (350 mg, 99%).

^{1}H NMR (500 MHz, DMSO-d_6, 25 °C) δ = 12.98 (s, 2H, HO−C^{14}), 7.03 (t, J = 2.5 Hz, 4H, H−$C^{12,15}$), 6.97 (td, J = 8.4, 2.0 Hz, 1H, H−C^{4}), 6.73 – 6.68 (m, 4H, H−$C^{3,21}$), 5.87 (ddt, J = 17.0, 10.3, 6.6 Hz, 2H, H−C^{19}), 5.15 (dq, J = 17.2, 1.7 Hz, 2H, H−C^{20}), 5.07 (dd, J = 10.3, 2.0 Hz, 2H, H−C^{20}), 4.03 (t, J = 6.6 Hz, 4H, H−C^{17}), 4.00 (t, J = 6.3 Hz, 4H, H−C^{5}), 3.97 (t, J = 6.5 Hz, 4H, H−C^{10}), 2.49 – 2.43 (m, 4H, H−C^{18}), 1.71 (dq, J = 12.1, 6.3 Hz, 8H, H−C^{6+9}), 1.45 (p, J = 3.4 Hz, 8H, H−C^{7+8}) ppm.

^{13}C{^{1}H} NMR (126 MHz, DMSO-d_6, 25 °C) = δ 167.0 (C^{14}), 159.8 (C^{11}), 159.6 (C^{16}), 147.5 (d, J_{C-F} = 8.2 Hz, C^{2}), 142.0 (d, J_{C-F} = 242.0 Hz, C^{1}), 134.8 (C^{19}), 132.8 (C^{13}), 123.5 (d, J_{C-F} = 5.3 Hz, C^{4}), 117.0 (C^{20}), 107.4, 107.4, 107.0 (C^{3}), 105.7 (C^{21}), 68.7 (C^{17}), 67.7 (C^{20}), 67.0 (C^{5}), 33.0 (C^{18}), 28.6, 28.5, 25.2, 25.2 ppm. Note: Certain carbon atoms could not be assigned due to signal overlap.

^{19}F NMR (471 MHz, DMSO-d$_6$, 25 °C) δ = –158.1 (td, J = 7.4, 2.2 Hz, F−C^{1}) ppm.

HR-ESI-TOF-MS (ESI–) m/z calculated for $C_{40}H_{48}FO_{10}^-$ ([M–H]$^-$) 707.3237, found 707.3238. Mass error: 0.1 ppm.

Machine (*Zs*)-0

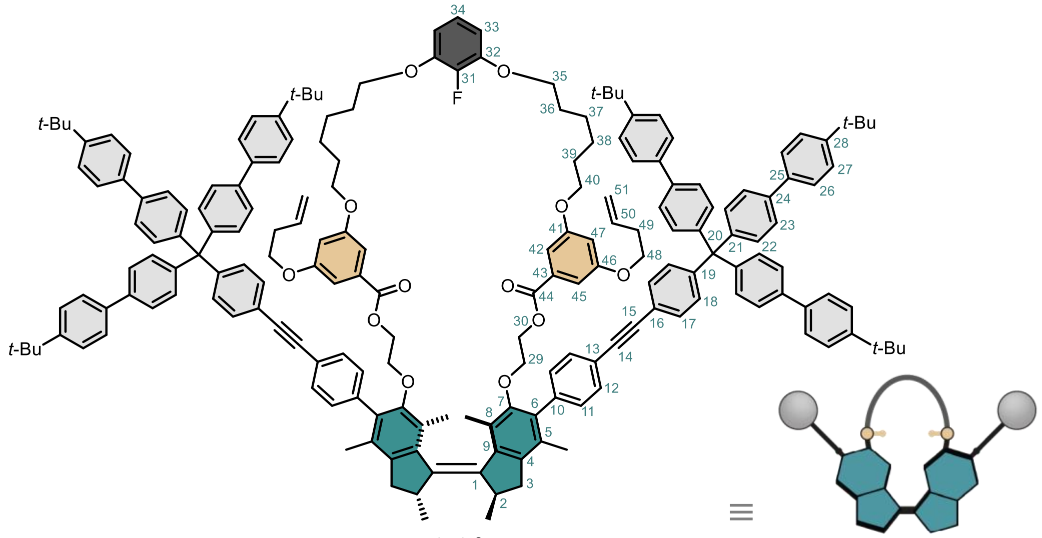

(*Zs*)-**0**
$C_{194}H_{197}FO_{12}$
Mw = 2739.63 g/mol

A solution of (*Zs*)-**MA** (15 mg, 7.3 µmol, 1.0 equiv.), **S19** (6 mg, 8 µmol, 1.1 equiv.), EDAC·HCl (22 mg, 116 µmol, 16 equiv.) and DMAP (22 mg, 182 µmol, 25 equiv.) in CH_2Cl_2 (7 mL) was stirred at 20 °C for 3 d. The reaction was concentrated and subsequently filtered over a silica plug using CH_2Cl_2. The filtrate was purified by *r*GPC chromatography ($CHCl_3$) affording machine (*Zs*)-**0** (11 mg, 55%) as a white solid. A UPLC pure sample was obtained by performing MPLC (SiO_2, PE/Et_2O gradient 100:0 ⟶ 80:20) with subsequent *r*GPC chromatography ($CHCl_3$).

^{1}H NMR (500 MHz, $CDCl_3$, 25 °C) δ = 7.57 (d, *J* = 8.5 Hz, 12H, H–C^{26}), 7.55 (d, *J* = 8.5 Hz, 12H, H–C^{23}), 7.52 (d, *J* = 8.6 Hz, 4H, H–C^{17}), 7.47 (d, *J* = 8.5 Hz, 16H, H–C^{12+27}), 7.40 – 7.33 (b, 2H, H–C^{11}), 7.36 (d, *J* = 8.5 Hz, 16H, H–C^{18+22}), 7.30 – 7.22 (b, 2H, H–C$^{11'}$), 7.04 (dd, *J* = 2.4, 1.3 Hz, 2H, H–C^{42}), 7.01 (dd, *J* = 2.4, 1.3 Hz, 2H, H–C^{45}), 6.83 (td, *J* = 8.4, 2.0 Hz, 1H, H–C^{34}), 6.57 (t, *J* = 2.3 Hz, 2H, H–C^{47}), 6.51 (dd, *J* = 8.4, 7.1 Hz, 2H, H–C^{33}), 5.82 (ddt, *J* = 17.0, 10.2, 6.6 Hz, 2H, H–C^{50}), 5.11 (dq, *J* = 17.2, 1.6 Hz, 2H, H–C^{51}), 5.06 (dq, *J* = 10.2, 1.3 Hz, 2H, H–C^{51}), 4.16 (ddd, *J* = 11.9, 5.5, 3.6 Hz, 2H, H–C^{30}), 4.09 (ddd, *J* = 12.0, 6.6, 3.6 Hz, 2H, H–C^{30}), 3.97 (t, *J* = 6.0 Hz, 4H, H–C^{35}), 3.95 – 3.83 (m, 8H, H–C^{40+48}), 3.72 (td, *J* = 7.4, 6.5, 4.0 Hz, 2H, H–C^{29}), 3.51 (ddt, *J* = 7.2, 5.2, 3.3 Hz, 2H, H–C^{29}), 3.41 (p, *J* = 6.8 Hz, 2H, H–C^{2}), 3.14 (dd, *J* = 14.8, 6.2 Hz, 2H, H–C^{3}), 2.52 – 2.42 (m, 6H, H–C^{3+49}), 2.03 (s, 6H,

CH_3–C^{5}), 1.77 – 1.67 (m, 8H, H–C^{36+39}), 1.63 (s, 6H, CH_3–C^{8}), 1.52 – 1.42 (m, 8H, H–C^{37+38}), 1.37 (s, 54H, CH_3–*t*-Bu), 1.15 (d, *J* = 6.6 Hz, 6H, CH_3–C^{2}) ppm.

$^{13}C\{^{1}H\}$ NMR (126 MHz, $CDCl_3$, 25 °C) δ = 166.2 (C^{44}), 160.1 (C^{41}), 159.8 (C^{46}), 152.9 (C^{7}), 150.4 (C^{28}), 148.1 (d, $J_{C–F}$ = 8.5 Hz, C^{32}), 147.2 (C^{19}), 145.4 (C^{21}), 144.1 (d, $J_{C–F}$ = 244.7 Hz, C^{31}), 141.8 (C^{9}), 141.4 (C^{1}), 140.7 (C^{2}), 138.7 (C^{24}), 138.4 (C^{10}), 137.8 (C^{25}), 134.4 (C^{50}), 133.6 (C^{6}), 131.8 (C^{43}), 131.6 (C^{22}), 131.3 (C^{17}), 131.0 (C^{18}), 130.8 – 130.2 (b, C^{11+12}) , 130.0 (C^{5}), 126.8 (C^{26}), 126.2 (C^{8+23}), 125.9 (C^{27}), 123.0 (d, $J_{C–F}$ = 4.8 Hz, C^{34}), 121.6 (C^{13}), 121.2 (C^{16}), 117.2 (C^{51}), 108.2 (C^{33}), 108.1 (C^{42}), 107.7 (C^{45}), 106.9 (C^{47}), 89.9 (C^{15}), 89.4 (C^{14}), 69.9 (C^{29}), 69.8 (C^{35}), 68.1 (C^{40}), 67.4 (C^{48}), 64.5 (C^{20}), 64.2 (C^{30}), 42.0 (C^{4}), 39.2 (C^{3}), 34.7 (C–*t*-Bu), 33.7 (C^{49}), 31.5 (CH_3–*t*-Bu), 29.0 (C^{39}), 29.0 (C^{36}), 25.8 (C^{37}), 25.6 (C^{38}), 20.8 (CH_3–C^{2}), 16.9 (CH_3–C^{5}), 15.1 (CH_3–C^{8}) ppm. Note: C^{11} and C^{12} are split into two low intensity and/or broad signal(s) due to diastereotopicity and conformational isomerism.

^{19}F NMR (471 MHz, $CDCl_3$, 25 °C) δ = –155.9 (t, *J* = 7.3 Hz, F–C^{31}) ppm.

HR-ESI-TOF-MS (ESI+) *m/z* calculated for $C_{194}H_{197}FO_{12}Na^{+}$ ($[M+Na]^{+}$) 2760.4681, found 2760.4756. Mass error: 2.7 ppm.

2.3 Synthesis of Rotaxane [2]-(*Z*s)-MR

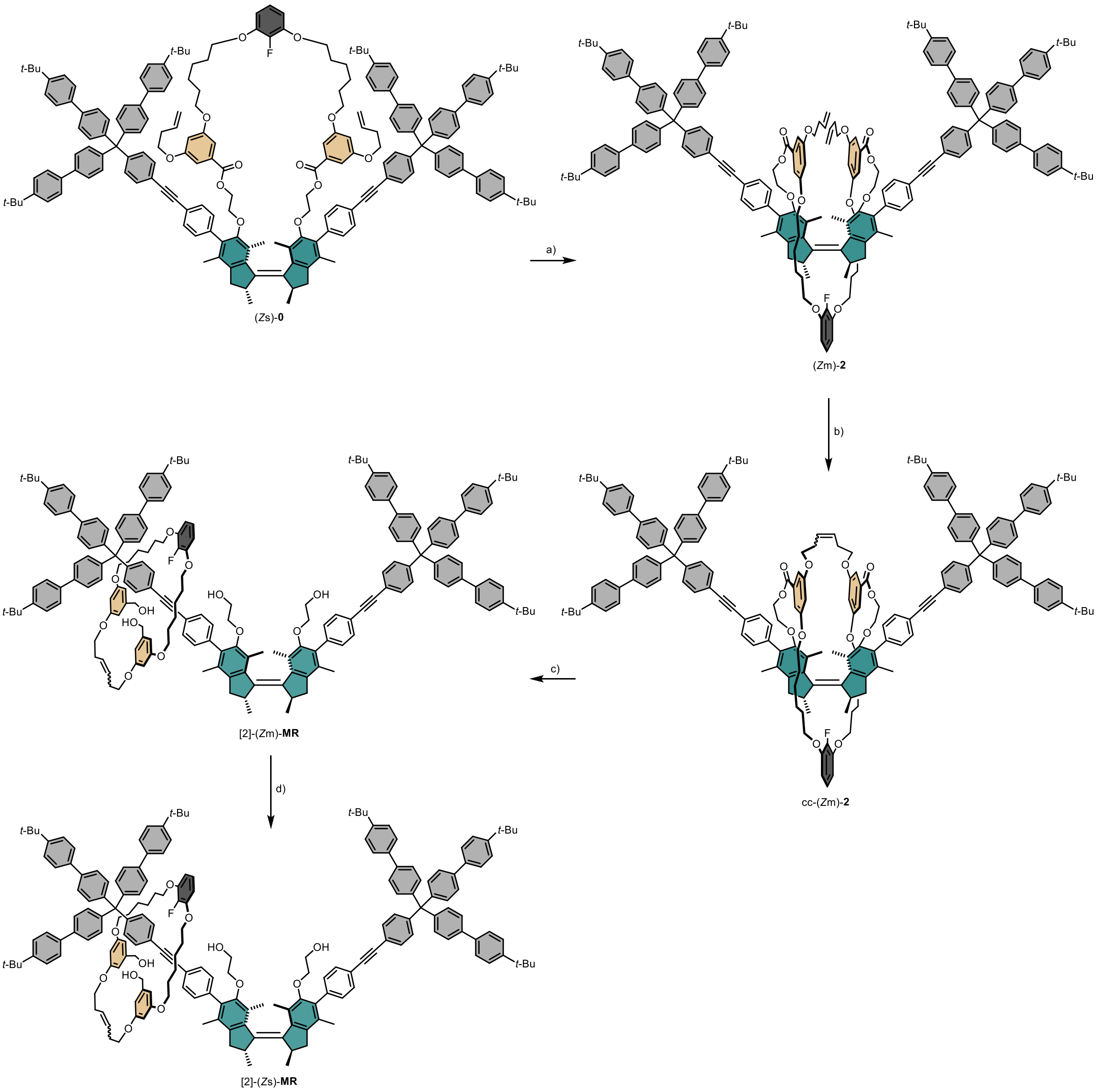

Supporting Figure S3 | Synthesis of motor rotaxane [2]-(*Z*s)-**MR**. a) Toluene, 312 nm, 20 °C, 2 h, 60% PSS; b) Grubbs catalyst M110, toluene, 20 °C, 2 d. c) DIBAL-H, toluene, 0 °C, 15 min; d) toluene/H_2O, $KNaC_4H_4O_6$, 80 °C, 1 h, 44% over 4 steps. Isolated yield of 72% when calculated from the total amount of available (*Z*m)-**2**. Only the (*R*,*R*)-enantiomer is shown for clarity.

Rotaxane [2]-(*Zs*)-MR

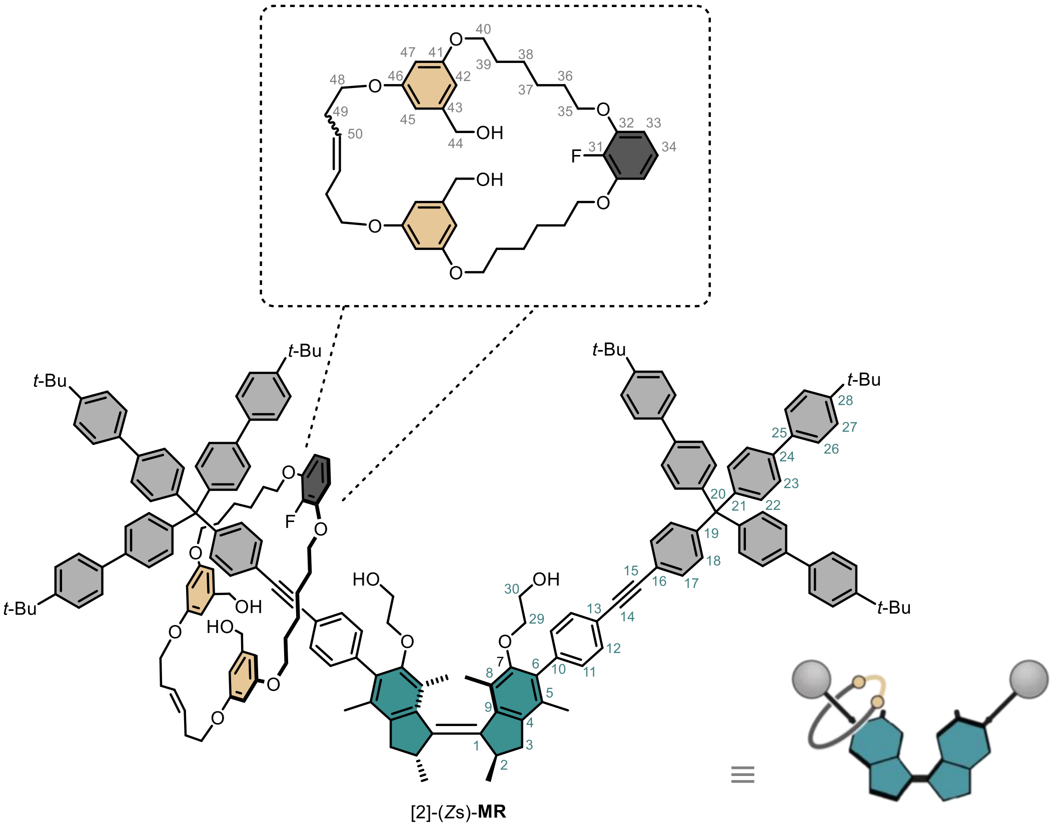

[2]-(*Zs*)-**MR**
$C_{192}H_{201}FO_{12}$
Mw = 2719.64

A solution of (*Zs*)-**0** (5 mg, 2.0 μmol, 1.0 equiv.) in anhydrous degassed toluene (5 mL) was irradiated with 312 nm UV light (Vilber Lourmat, 12 W) for 2 h. Grubbs catalyst M110 (5 mM in toluene, 35 μL, 10 mol%) was added and the reaction mixture was heated at 50 °C for 2 d in the glovebox with an open cap. UPLC-HRMS showed full conversion to covalently captured cc-(*Zm*)-**2**. **HRMS** (ESI+) *m/z* calculated for $C_{192}H_{193}FO_{12}Na$ $[M+Na]^+$ 2734.4436, found 2734.4434. Mass error 0.1 ppm. Note: after one day, another portion of Grubbs catalyst M110 was added (5 mM in toluene, 17 μL, 5 mol%).

DIBAL-H (1.2 M in toluene, 15 μL, 20 equiv.) was added to the reaction at 0 °C under a flow of argon. After 10 min, sat. aq. Rochelle's salt solution was added and the biphasic mixture was vigorously stirred at 80 °C for 1 h, followed by 1 h at room temperature. The phases were allowed to separate and the aqueous layer was extracted with EtOAc. The combined organic layers were washed with brine, dried over Na_2SO_4 and concentrated under reduced pressure, Note: drying over $MgSO_4$ is discouraged as the product adsorption to the magnesium surface might result in lower yields.

The crude product was first purified by *r*GPC-SEC ($CHCl_3$, 8 cycles), then dry-loaded on Celite and subsequently purified by MPLC (SiO_2, CH_2Cl_2/MeOH gradient 100:0 ⟶ 85:15) affording motor rotaxane [2]-(*Z*s)-**MR** (2.2 mg, 44%) as a white solid after lyophilization from benzene. ^{1}H and ^{19}F NMR spectroscopy revealed that the product had a 1:2.6 ratio of *E/Z* isomer of the macrocycle. Note: using silica during dry loading is discouraged to prevent possible degradation.

^{1}H NMR (500 MHz, $CDCl_3$, 25 °C) δ = 7.54 (d, *J* = 8.1 Hz, 16H, H–C^{12+26}), 7.50 (d, *J* = 8.2 Hz, 12H, H–C^{23}), 7.45 (d, *J* = 8.2 Hz, 12H, H–C^{27}), 7.39 (d, *J* = 8.0 Hz, 4H, H–C^{17}), 7.34 (d, *J* = 8.6 Hz, 4H, H–C^{18}), 7.30 (d, *J* = 8.2 Hz, 12H, H–C^{22}), 7.32 – 7.18 (b, 4H, H–C^{11}), 6.76 (q, *J* = 7.8 Hz, 1H, H–C^{34}), 6.47 (t, *J* = 7.7 Hz, 2H, H–C^{33}), 6.40 (d, *J* = 6.7 Hz, 4H, H–C^{42+45}), 6.24 (s, 2H, H–C^{47}), 5.54 (t, *J* = 3.2 Hz, H–C^{50Z}), 5.45 (t, *J* = 4.5 Hz, H–C^{50E}), 4.47 (s, 4H, H–C^{44}), 3.92 (t, *J* = 6.3 Hz, 4H, H–C^{35}), 3.85 – 3.77 (m, 4H, H–C^{48}), 3.72 (t, *J* = 6.3 Hz, 4H, H–C^{40}), 3.55 (qd, *J* = 8.5, 7.2, 4.6 Hz, 2H, H–C^{29}), 3.40 (p, *J* = 6.8 Hz, 2H, H–C^{2}), 3.39 – 3.30 (m, 6H, H–C^{29+30}), 3.13 (dd, *J* = 14.9, 6.1 Hz, 2H, H–C^{3}), 2.46 (d, *J* = 14.8 Hz, 2H, H–C^{3}), 2.45 – 2.37 (m, H–C^{49E}), 2.37 – 2.32 (m, H–C^{49Z}), 2.00 (s, 6H, CH_3–C^{5}), 1.69 – 1.59 (m, 8H, H–C^{36+39}), 1.56 (s, 6H, CH_3–C^{8}), 1.41 – 1.30 (m, 8H, H–C^{37+38}), 1.36 (s, 54H, CH_3–*t*-Bu), 1.14 (d, *J* = 6.6 Hz, 6H, CH_3–C^{2}) ppm.

^{13}C{^{1}H} NMR (151 MHz, $CDCl_3$, 25 °C) δ = 160.5 (C^{41Z}),160.5 (C^{41E}), 160.3 (C^{46Z}), 160.3 (C^{46E}), 153.0 (C^{7}), 150.4 (C^{28}), 148.1 (d, *J* = 8.2 Hz, C^{32}), 147.2 (C^{19}), 145.3 (C^{21}), 144.2 (d, *J* = 252.1 Hz, C^{31}), 143.3 (C^{43}), 141.9 (C^{9}), 141.4 (C^{1}), 140.6 (C^{4}), 138.7 (C^{24}), 138.4 (C^{10}), 137.8 (C^{25}), 133.3 (C^{6}), 131.6 (C^{22}),131.3 (C^{18}), 131.3 (C^{12}), 131.0 (C^{17}), 130.7 (C^{11}), 130.4 ($C^{11'}$), 130.0 (C^{5}), 128.5 (C^{50Z}), 127.9 (C^{50E}), 126.7 (C^{26}), 126.2 (C^{23}), 126.0 (C^{8}), 125.9 (C^{27}), 123.1 (d, *J* = 5.6 Hz, (C^{34}), 121.8 (C^{13}), 121.1 (C^{16}), 108.5 (C^{33E}), 108.4 (C^{33Z}), 105.7 (C^{45E}), 105.6 (C^{45Z}), 105.3 (C^{42Z}), 104.9 (C^{42E}), 100.6 (C^{47E}), 100.3 (C^{47Z}), 89.8 (C^{15}), 89.7 (C^{14}), 73.4 (C^{29}), 69.8 (C^{35}), 67.7 (C^{40}), 67.5 (C^{48Z}), 67.4 (C^{48E}), 65.4 (C^{44}), 64.4 (C^{20}), 61.8 (C^{30}), 41.8 (C^{2}), 39.2 (C^{3}),34.7 (C–*t*-Bu), 32.6 (C^{49Z}), 31.5 (CH_3–*t*-Bu), 29.2 (C^{39Z}), 29.1 (C^{36E}), 29.1 (C^{36Z}), 27.6 (C^{49E}),25.7 (C^{37}), 25.63 (C^{38}), 20.8 (CH_3–C^{2}), 16.9 (CH_3–C^{5}), 15.2 (CH_3–C^{8}) ppm. Note: C^{11} and $C^{11'}$ are split into two low intensity and/or broad signal(s) due to diastereotopicity and conformational isomerism.

^{19}F NMR (471 MHz, $CDCl_3$, 25 °C) δ = –155.1 (t, *J* = 6.5 Hz, F–C^{31E}), –155.3 (t, *J* = 6.4 Hz, F–C^{31Z}) ppm.

HR-ESI-TOF-MS (ESI+) *m/z* calculated for $C_{192}H_{202}FO_{12}^+$ ($[M+H]^+$) 2718.5176, found 2718.5286. Mass error: 4.0 ppm.

2.4 Synthesis of Bz-(*Zs*)-MA, OM and (*Zs*)-MM

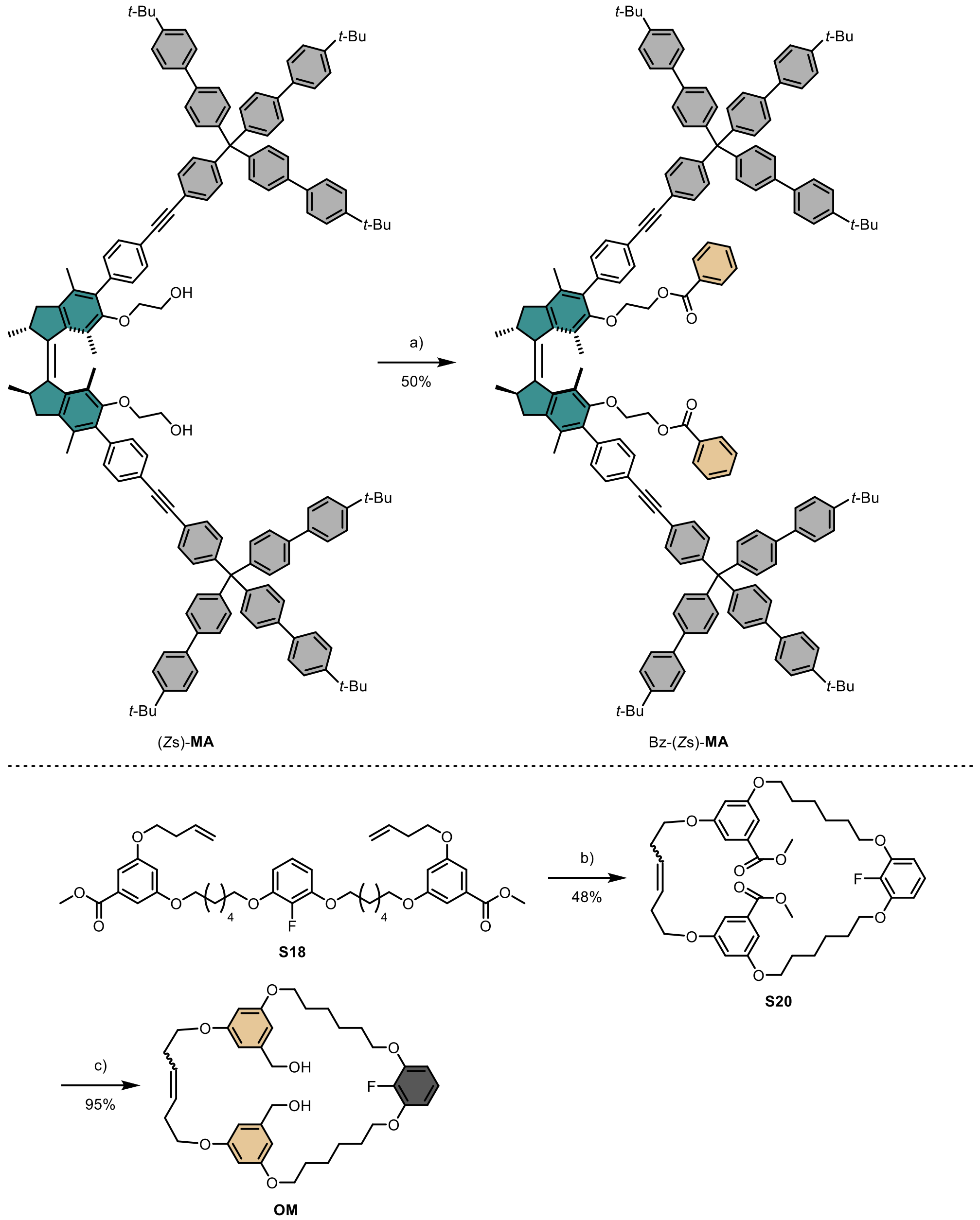

Supporting Figure S4 | Synthesis of Bz-(*Z*s)-**MA** (top) and **OM** (bottom). a) Bz_2O, DMAP, CH_2Cl_2, 40 °C, 3 h, 50%; b) Grubbs catalyst M110 toluene, 25 °C, 1 d, 48%; c) DIBAL-H, THF, RT, 1 h, 95%. Only the (*R*,*R*)-enantiomer is shown for clarity.

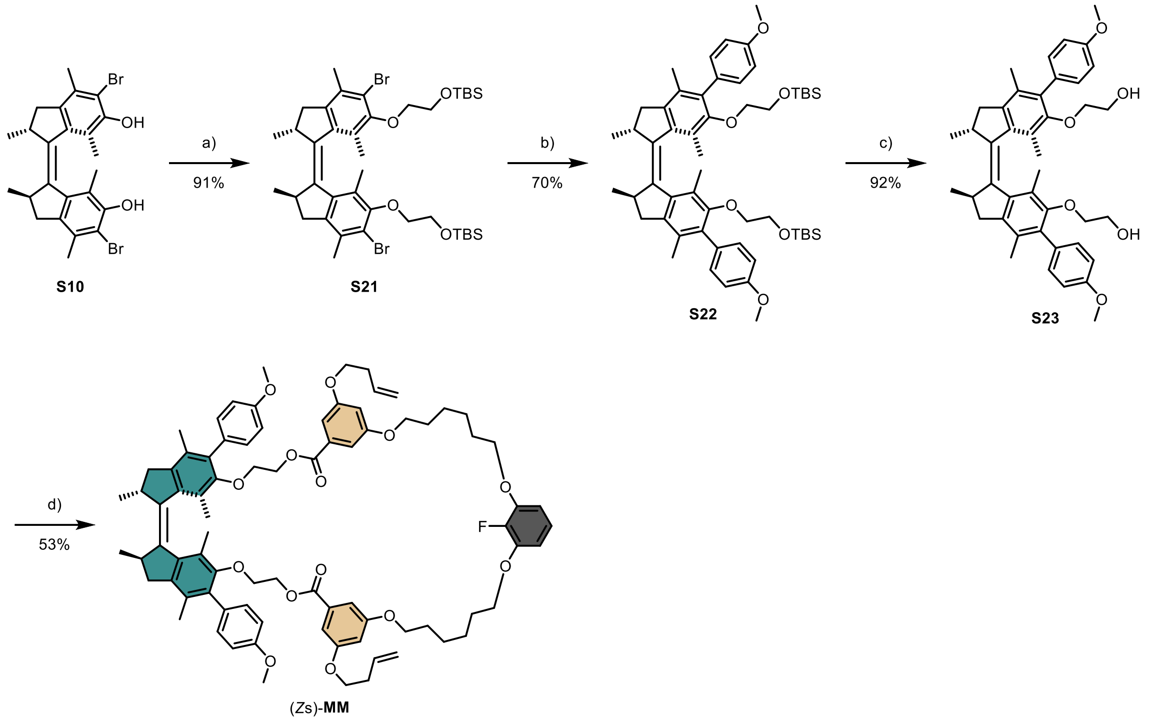

Supporting Figure S5 | Synthesis of motor macrocycle (*Z*s)-**MM**. a) $TBSOCH_2CH_2Br$, K_2CO_3, DMF, 70 °C, 18 h, 91%; b) 4-hydroxy phenyl boronic acid, Pd(dppf)Cl_2·DCM, K_2CO_3, 1,4-dioxane/H_2O, 80 °C, 5 h, 70%; c) TBAF, THF, 0 °C, 40 min, 92%; d) **S19**, EDAC·HCl, DMAP, CH_2Cl_2, 20 °C, 5 d, 53%. Only the (*R*,*R*)-enantiomer is shown for clarity.

Benzoylated Motor Axle Bz-(*Zs*)-MA

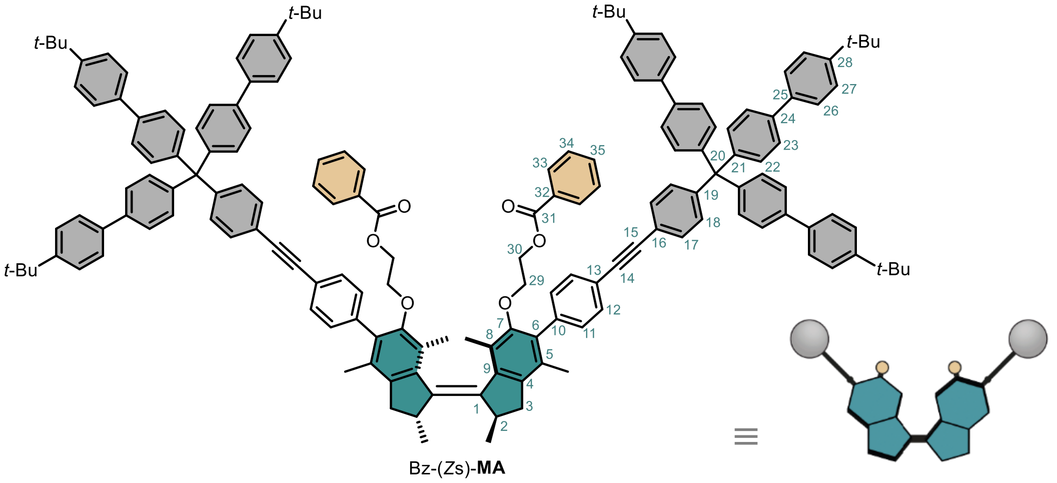

Bz-(*Zs*)-**MA**
$C_{168}H_{160}O_6$
Mw = 2275.06

A solution of (*Zs*)-**MA** (18 mg, 8.7 μmol, 1.0 equiv.), benzoyl anhydride (16 mg, 70 μmol, 8.0 equiv.) and DMAP (8.0 mg, 70 μmol, 8.0 equiv.) in CH_2Cl_2 was stirred at 40 °C for 3 h. The reaction was concentrated and immediately purified by MPLC (SiO_2, cHex/CH_2Cl_2 gradient 100:0 ⟶ 25:75). Second purification by MPLC (SiO_2, PE/Et_2O gradient 100:0 ⟶ 80:20), affording Bz-(*Zs*)-**MA** (10 mg, 50%) as a white powder after lyophilization from benzene.

^{1}H NMR (500 MHz, $CDCl_3$, 25 °C) δ = 7.87 (d, *J* = 7.6 Hz, 4H, H–C^{33}), 7.57 (d, *J* = 7.9 Hz, 12H, H–C^{26}), 7.54 (d, *J* = 7.9 Hz, 16H, H–C^{12+23}), 7.49 (d, *J* = 7.8 Hz, 6H, H–C^{17+35}), 7.46 (d, *J* = 7.5 Hz, 12H, H–C^{27}), 7.40 (t, *J* = 7.4 Hz, 4H, H–C^{34}), 7.36 (d, *J* = 7.7 Hz, 18H, H–C$^{18+22+11}$), 7.29 – 7.16 (b, 2H, H–C$^{11'}$), 4.23 (dd, *J* = 10.6, 4.9 Hz, 2H, H–C^{30}), 4.14 (dd, *J* = 11.7, 6.3 Hz, 2H, H–C^{30}), 3.73 (t, *J* = 8.3 Hz, 2H, H–C^{29}), 3.56 (t, *J* = 8.2 Hz, 2H, H–C^{29}), 3.40 (p, *J* = 7.0 Hz, 2H, H–C^{2}), 3.14 (dd, *J* = 15.1, 6.2 Hz, 2H, H–C^{3}), 2.47 (d, *J* = 14.7 Hz, 6H, H–C^{3}), 2.02 (s, 6H, CH_3–C^{5}), 1.60 (s, 6H, CH_3–C^{8}), 1.36 (s, 54H, CH_3–*t*-Bu), 1.13 (d, *J* = 6.6 Hz, 6H, CH_3–C^{2}) ppm. H–C^{11} is split into two low intensity and/or broad signal(s) due to diastereotopicity and conformational isomerism.

^{13}C{^{1}H} NMR (126 MHz, $CDCl_3$, 25 °C) δ = 165.4 (C^{31}), 151.8 (C^{7}), 149.4 (C^{28}), 146.3 (C^{19}), 144.3 (C^{21}), 140.9 (C^{9}), 140.3 (C^{1}), 139.7 (C^{4}), 137.7 (C^{24}), 137.5 (C^{32}), 136.7 (C^{25}), 132.5 (C^{6}), 132.0 (C^{35}), 130.6 (C^{18+22}), 130.3 (C^{12}), 130.2 (C^{11}), 129.9 (C^{17}), 129.4 (C$^{11'}$), 129.0 (C^{5}), 128.8 (C^{33}), 127.4 (C^{34}), 125.7 (C^{26}), 125.2 (C^{8+23}), 124.8 (C^{27}), 120.7 (C^{13}), 120.1 (C^{16}), 88.9 (C^{14}), 88.6 (C^{15}), 68.8 (C^{29}), 63.5 (C^{20}), 63.2 (C^{30}), 40.9 (C^{2}), 38.2 (C^{3}), 33.7 (C–*t*-Bu), 30.5 (CH_3–*t*-Bu), 19.7 (CH_3–C^{2}), 15.8 (CH_3–C^{5}), 13.9 (CH_3–C^{8}) ppm. C^{11} and C^{12} are split into two

low intensity and/or broad signal(s) due to diastereotopicity and conformational isomerism. The signal of C^8 is overlapping with C^{23} and could be assigned using ^1H ^{13}C HMBC.

HR-ESI-TOF-MS (ESI+) *m/z* calculated for $C_{168}H_{160}O_6Na^+$ ($[M+Na]^+$) 2296.2107, found 2296.1995. Mass error: 4.9 ppm.

Macrocycle S20

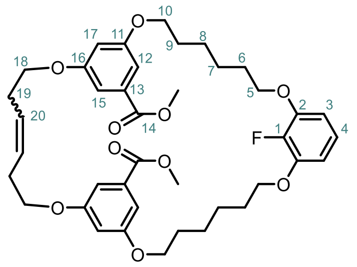

S20
$C_{40}H_{49}FO_{10}$
Mw = 708.81 g/mol

A solution of **S18** (164 mg, 0.258 mmol, 1.0 equiv.) and Grubbs catalyst M110 (8.0 mg, 0.010 mmol, 4 mol%) in degassed toluene (30 mL) was stirred at 20 °C for 24 h with an open vessel in the glovebox. The solution was concentrated under reduced pressure and purified by MPLC (SiO_2, PE/Et_2O gradient 100:0 ⟶ 40:60), affording the title compound **S20** (81 mg, 48%), *E/Z* ratio (1:2), as an off-white solid.

^{1}H NMR (500 MHz, $CDCl_3$, 25 °C) δ = 7.18 – 7.12 (m, 4H, H–$C^{12,15}$), 6.91 (td, J = 8.4, 2.1 Hz, 1H, H–C^{4}), 6.62 (dt, J = 3.9, 2.4 Hz, 2H, H–C^{17}), 6.58 (ddd, J = 8.4, 7.1, 1.0 Hz, 2H, H–C^{3}), 5.68 – 5.58 (m, 2H, H–$C^{20Z,E}$), 4.05 (t, J = 6.1 Hz, 4H, H–C^{5}), 4.04 – 3.93 (m, 8H, H–$C^{10,18}$), 3.88 (s, 4H, MeO–C^{14Z}), 3.88 (s, 2H, MeO–C^{14E}), 2.58 (q, J = 6.2 Hz, 1H, H–C^{19E}), 2.50 (q, J = 5.2 Hz, 3H, H–C^{19Z}), 1.79 (p, J z= 6.1 Hz, 8H, H–$C^{6,9}$), 1.57 – 1.48 (m, 8H, H–$C^{7,8}$) ppm.

^{13}C{^{1}H} NMR (126 MHz, $CDCl_3$, 25 °C) δ = 167.1 (C^{14Z}), 167.1 (C^{14E}), 160.2, 160.1, 160.0, 148.2 (d, $J_{C–F}$ = 8.4 Hz, C^{2}), 144.0 (d, $J_{C–F}$ = 244.2 Hz, C^{1E}), 144.0 (d, $J_{C–F}$ = 244.3 Hz, C^{1Z}), 132.0, 132.0, 128.6 (C^{20Z}), 127.9 (C^{20E}), 123.1 (d, $J_{C–F}$ = 5.2 Hz, C^{4}), 108.6, 108.2, 108.2, 108.1, 107.7, 107.7, 106.8 (C^{17E}), 106.6 (C^{17Z}), 69.9 (C^{5E}), 69.8 (C^{5Z}), 68.1 (C^{10}), 68.0 (C^{18Z}), 67.7 (C^{18E}), 52.3 (MeO–C^{14}), 32.5 (C^{19Z}), 29.2, 29.1, 29.1, 29.1, 27.6 (C^{19E}), 25.8, 25.7 (C^{7+8Z}), 25.7 (C^{7+8E}) ppm. Some carbon atoms could not be assigned due to signal overlap.

^{19}F NMR (471 MHz, $CDCl_3$, 25 °C) δ = –156.4 (td, J = 7.1, 2.2 Hz, F–C^{1E}), –156.5 (td, J = 7.1, 2.2 Hz, F–C^{1Z}) ppm.

HR-ESI-TOF-MS (ESI+) *m/z* calculated for $C_{40}H_{50}FO_{10}^+$ ($[M+H]^+$) 709.3383, found 709.3381. Mass error: 0.3 ppm.

Olefin Macrocycle OM

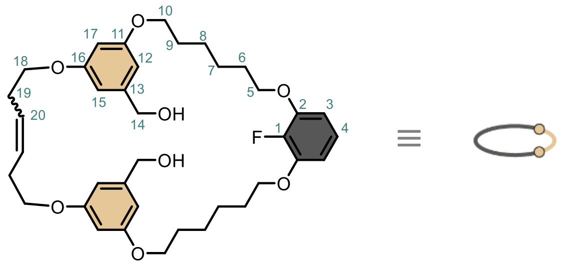

OM
$C_{38}H_{49}FO_8$
Mw = 652.79 g/mol

A solution of **S20** (48 mg, 0.068 mmol, 1.0 equiv.) in THF (2 mL) was reacted with DIBAL-H (1.2 M in toluene, 0.56 mL, 0.68 mmol, 10 equiv.) at 25 °C for 1 h. The reaction was carefully quenched by addition of water, then aq. 1 M HCl, after which the product was extracted using EtOAc. Combined phases were washed with brine, dried over $MgSO_4$ and concentrated under reduced pressure. The crude product was purified by MPLC (SiO_2, CH_2Cl_2/MeOH gradient 100:0 ⟶ 85:15), affording olefin macrocycle **OM** (42 mg, 95%), *E/Z* ratio (1:2), as a white solid.

^{1}H NMR (500 MHz, $CDCl_3$, 25 °C) δ = 6.91 (td, *J* = 8.4, 2.1 Hz, 1H, H–C^{4}), 6.57 (ddd, *J* = 8.4, 7.2, 1.2 Hz, 2H, H–C^{3}), 6.48 (t, *J* = 1.9 Hz, 4H, H–C^{12+15}), 6.35 (q, *J* = 2.4 Hz, 2H, H–C^{17}), 5.64 (m, 2H, H–C^{20}), 4.59 (s, 3H, H–C^{14Z}), 4.56 (s, 1H, H–C^{14E}), 4.04 (t, *J* = 6.0, Hz, 4H, H–C^{5}), 3.99 – 3.92 (m, 8H, H–C^{10+18}), 2.59 – 2.54 (m, 1H, H–C^{19E}), 2.51 – 2.46 (m, 3H, H–C^{19Z}), 1.84 – 1.73 (m, 8H, H–C^{6+9}), 1.59 – 1.48 (m, 8H, H–C^{7+8}) ppm.

Z-**S24**: **^{13}C{^{1}H} NMR** (126 MHz, $CDCl_3$, 25 °C) δ = 160.5, 160.4, 148.2 (d, J_{C-F} = 8.4 Hz, C^{2}), 143.9 (d, J_{C-F} = 244.4 Hz, C^{1}), 143.4 (C^{13}), 128.6 (C^{20}), 123.1 (d, J_{C-F} = 5.1 Hz, C^{4}), 108.0 (C^{3}), 106.0, 105.0, 100.6 C^{17}), 69.8 (C^{5}), 67.9 (C^{10}), 67.7 (C^{18}), 65.5 (C^{14}), 32.6 (C^{19}), 29.1, 29.1, 29.1, 25.8, 25.7 ppm. Some carbon atoms could not be assigned due to signal overlap.

E-**S24**: **^{13}C{^{1}H} NMR** (126 MHz, $CDCl_3$, 25 °C) δ = 160.5, 160.3, 148.2 (d, J_{C-F} = 8.4 Hz, C^{2}), 143.9 (d, J_{C-F} = 244.3 Hz, C^{1}), 143.5 (C^{13}), 128.0 (C^{20}), 123.1 (d, J_{C-F} = 5.1 Hz, C^{4}), 108.0 (C^{3}), 105.4, 105.3, 100.8 (C^{17}), 69.8 (C^{5}), 67.9 (C^{10}), 67.5 (C^{18}), 65.4 (C^{14}), 29.1, 29.0, 27.7 (C^{19}), 25.8, 25.6 ppm. Some carbon atoms could not be assigned due to signal overlap.

^{19}F NMR (471 MHz, $CDCl_3$, 25 °C) δ = –156.5 (td, *J* = 7.2, 2.2 Hz, F–C^{1E}), –156.5 (td, *J* = 7.0, 2.3 Hz, F–C^{1Z}) ppm.

HR-ESI-TOF-MS (ESI+) *m/z* calculated for $C_{38}H_{49}FO_8^{+}$ ($[M+H]^{+}$) 653.3484, found 653.3486. Mass error: 0.3 ppm.

(*Z*)-((((5,5'-Dibromo-2,2',4,4',7,7'-hexamethyl-2,2',3,3'-tetrahydro-[1,1'-biindenylidene]-6,6'-diyl)bis(oxy))bis(ethane-2,1-diyl))bis(oxy))bis(*tert*-butyldimethylsilane) (S21)

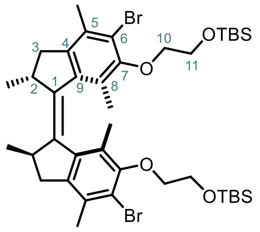

S21

$C_{40}H_{62}Br_2O_4Si_2$

Mw = 822.90

A suspension of **S10** (511 mg, 1.00 mmol, 1.0 equiv.), (2-bromethoxy)-*tert*-butyldimethylsilane (2.2 mL, 2.45 g, 10.3 mmol, 10 equiv.) and K_2CO_3 (1.4 g, 10.1 mmol, 10 equiv.) in DMF (10 mL) was heated at 70 °C for 18 h. The reaction was quenched with sat. aq. NH_4Cl and extracted with Et_2O. Combined organic phases were washed with aq. 5 m/m% LiCl, brine, dried over anhydrous $MgSO_4$ and concentrated under reduced pressure. The product was purified by MPLC (SiO_2, PE/Et_2O gradient 100:0 ⟶ 90:10) affording the title compound **S21** (757 mg, 91%) as a yellow oil.

^{1}H NMR (500 MHz, $CDCl_3$, 25 °C) δ = 4.02 – 3.91 (m, 4H, H−C^{11}), 3.91 – 3.85 (m, 4H, H−C^{10}), 3.32 (p, J = 6.7 Hz, 2H, H−C^{2}), 3.11 (dd, J = 14.8, 6.3 Hz, 2H, H−C^{3}), 2.45 (d, J = 14.7 Hz, 2H, H−C^{3}), 2.34 (s, 6H, CH_3−C^{5}), 1.51 (s, 6H, CH_3−C^{8}), 1.06 (d, J = 6.8 Hz, 6H, CH_3−C^{2}), 0.89 (s, 18H, CH_3−*t*-Bu), 0.08 (s, 6H, CH_3−Si), 0.08 (s, 6H, CH_3−Si) ppm.

^{13}C{^{1}H} NMR (126 MHz, $CDCl_3$, 25 °C) δ = 152.8 (C^{7}), 141.2 (C^{9}), 140.9 (C^{1+3}), 131.9 (C^{4}), 127.8, 119.4 (C^{6}), 73.3 (C^{10}), 62.7 (C^{11}), 42.0 (C^{2}), 39.7 (C^{3}), 26.1 (CH_3−*t*-Bu), 20.5 (CH_3−C^{2}), 19.5 (CH_3−C^{5}), 18.5 (C_q−*t*-Bu), 15.3 (CH_3−C^{8}), –5.1 (CH_3−Si), –5.1 (CH_3−Si) ppm.

HR-ESI-TOF-MS (ESI+) *m/z* calculated for $C_{40}H_{63}O_4Si_2^+$ ($[M+H]^+$) 821.2626, found 821.2610. Mass error: 1.9 ppm.

(*Z*)-((((5,5'-Bis(4-methoxyphenyl)-2,2',4,4',7,7'-hexamethyl-2,2',3,3'-tetrahydro-[1,1'-biindenylidene]-6,6'-diyl)bis(oxy))bis(ethane-2,1-diyl))bis(oxy))bis(*tert*-butyldimethyl silane) (S22)

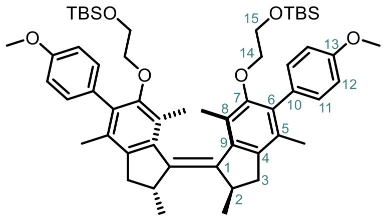

S22

$C_{54}H_{76}O_6Si_2$

Mw = 877.35

Under nitrogen atmosphere, a suspension of **S21** (201 mg, 0.273 mmol, 1.0 equiv.), (4-methoxyphenyl)boronic acid (167 mg, 1.10 mmol, 4.0 equiv.) and K_2CO_3 (377 mg, 2.70 mmol, 10.0 equiv.) in 1,4-dioxane/water 5:1 mixture (3.3 mL) was degassed using freeze-pump-thaw. Pd(dppf)Cl_2·DCM (30 mg, 41 μmol, 15 mol%) was added to the mixture which was subsequently heated at 80 °C for 5 h. The reaction was quenched with sat. aq. NH_4Cl solution and extracted with EtOAc. The combined organic phases were concentrated under reduced pressure and the crude product was purified by MPLC (SiO_2, cHex/EtOAc gradient 100:0 ⟶ 90:10), affording the title compound **S22** (182 mg, 76%) as an off-white solid.

^{1}H NMR (500 MHz, CD_2Cl_2, 25 °C) δ = 7.28 – 7.13 (b, 4H, H–C^{11}), 6.94 (d, *J* = 8.9 Hz, 4H, H–C^{12}), 3.84 (s, 6H, MeO–C^{13}), 3.50 (ddd, *J* = 9.1, 6.3, 4.8 Hz, 4H, H–C^{14}), 3.45 – 3.37 (m, 6H, H–C^{2+14}), 3.33 – 3.27 (m, 2H, H–C^{14}), 3.13 (dd, *J* = 14.8, 6.3 Hz, 2H, H–C^{3}), 2.47 (d, *J* = 14.8 Hz, 2H, H–C^{3}), 2.02 (s, 6H, CH_3–C^{5}), 1.56 (s, 6H, CH_3–C^{8}), 1.13 (d, *J* = 6.7 Hz, 6H, CH_3–C^{2}), 0.80 (s, 18H, CH_3–*t*-Bu), –0.07 (s, 6H, CH_3–Si), –0.07 (s, 6H, CH_3–Si) ppm. H–C^{11} is split into two low intensity and/or broad signal(s) due to diastereotopicity and conformational isomerism.

^{13}C{^{1}H} NMR (126 MHz, CD_2Cl_2, 25 °C) δ = 158.9 (C^{13}), 154.2 (C^{7}), 141.8 (C^{9}), 141.6 (C^{1}), 140.5 (C^{4}), 134.2 (C^{6}), 132.0 (C^{11}), 131.2 (C^{10}), 130.6 (C^{5}), 126.8 (C^{8}), 113.7 (C^{12}), 73.6 (C^{14}), 62.9 (C^{15}), 55.7 (MeO–C^{13}), 42.3 (C^{2}), 39.7 (C^{3}), 26.2 (CH_3–*t*-Bu), 20.9 (CH_3–C^{2}), 18.7 (C–*t*-Bu), 17.0 (CH_3–C^{5}), 15.1 (CH_3–C^{8}), –5.1 (CH_3–Si), –5.1 (CH_3–Si) ppm. C^{11} is split into two low intensity and/or broad signal(s) due to diastereotopicity and conformational isomerism.

HR-ESI-TOF-MS (ESI+) *m/z* calculated for $C_{54}H_{77}O_6Si_2^+$ ($[M+H]^+$) 877.5259, found 877.5245. Mass error: 1.6 ppm.

(*Z*)-2,2'-((5,5'-Bis(4-methoxyphenyl)-2,2',4,4',7,7'-hexamethyl-2,2',3,3'-tetrahydro-[1,1'-biindenylidene]-6,6'-diyl)bis(oxy))diethanol (S23)

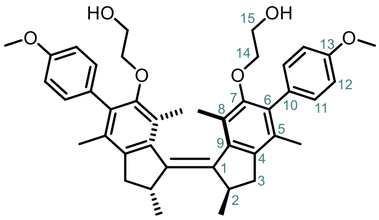

S23

$C_{42}H_{48}O_6$

Mw = 648.83

Under nitrogen atmosphere, a solution of **S22** (100 mg, 0.114 mmol, 1.0 equiv.) in anhydrous THF (3.8 mL) was reacted with TBAF (1 M in THF, 0.57 mL, 0.57 mmol, 5.0 equiv.) at 0 °C for 40 min. The reaction was quenched by addition of sat. aq. NH_4Cl and extracted with EtOAc. The combined organic phases were washed with brine solution and concentrated under reduced pressure and the crude product was purified by MPLC (SiO_2, CH_2Cl_2/MeOH gradient 100:0 ⟶ 95:5), affording the title compound **S23** (68 mg, 92%) as a white powder.

^{1}H NMR (500 MHz, CD_2Cl_2, 25 °C) δ = 7.22 (d, *J* = 5.6 Hz, 4H, H–C^{11}), 6.97 (d, *J* = 8.9 Hz, 4H, H–C^{12}), 3.84 (s, 6H, MeO–C^{13}), 3.66 – 3.60 (m, 2H, H–C^{14}), 3.44 – 3.37 (m, 6H, H–C$^{2+14+15}$), 3.37 – 3.31 (m, 2H, H–C^{15}), 3.14 (dd, *J* = 14.9, 6.3 Hz, 2H, H–C^{3}), 2.49 (d, *J* = 14.8 Hz, 2H, H–C^{3}), 2.02 (s, 6H, CH_3–C^{5}), 1.57 (s, 6H, CH_3–C^{8}), 1.14 (d, *J* = 6.7 Hz, 6H, CH_3–C^{2}) ppm. H–C^{11} is split into two low intensity and/or broad signal(s) due to diastereotopicity and conformational isomerism.

^{13}C{^{1}H} NMR (126 MHz, CD_2Cl_2, 25 °C) δ = 159.1 (C^{13}), 153.8 (C^{7}), 142.0 (C^{9}), 141.8 (C^{1}), 140.9 (C^{4}), 134.1 (C^{5}), 132.1 (C^{11}), 131.9 (C$^{11'}$), 131.0 (C^{6}), 130.9 (C^{10}), 126.4 (C^{8}), 113.9 (C^{12}), 73.9 (C^{15}), 62.3 (C^{14}), 55.8 (MeO–C^{13}), 42.3 (C^{2}), 39.7 (C^{3}), 20.8 (CH_3–C^{2}), 17.0 (CH_3–C^{5}), 15.4 (CH_3–C^{8}) ppm. C^{11} is split into two low intensity and/or broad signal(s) due to diastereotopicity and conformational isomerism.

HR-ESI-TOF-MS (ESI–) *m/z* calculated for $C_{52}H_{48}O_6Cl^-$ ($[M+Cl]^-$) 683.3139, found 683.3137. Mass error: 0.4 ppm.

Motor Macrocycle (*Zs*)-MM

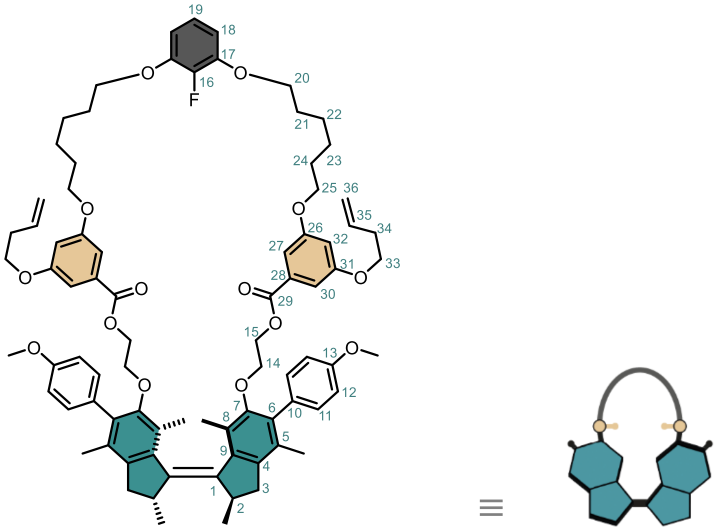

(*Zs*)-**MM**
$C_{82}H_{93}FO_{14}$
Mw = 1321.61

A solution of **S19** (22 mg, 33 μmol, 1.0 equiv.), **S23** (24 mg, 34 μmol, 1.0 equiv.), DMAP (81 mg, 0.66 mmol, 20 equiv.) and EDAC·HCl (162 mg, 846 μmol, 26 equiv.) were dissolved in anhydrous CH_2Cl_2 (33 mL). After 5 days, the mixture was diluted with CH_2Cl_2 (30 mL) and aq. 1 M HCl solution was added. The phases were separated and the aq. phase was extracted with EtOAc. The combined phases were washed with brine, dried over $MgSO_4$ and concentrated under reduced pressure. The crude product was purified by MPLC (SiO_2, cHex/EtOAc gradient 100:0 ⟶ 85:15), affording motor macrocycle (*Zs*)-**MM** (23 mg, 53%) as an off-white solid.

^{1}H NMR (500 MHz, $CDCl_3$, 25 °C) δ = 7.29 – 7.07 (b, 4H, H–C^{11}), 7.02 (dd, *J* = 2.4, 1.3 Hz, 2H, H–C^{27}), 7.01 (dd, *J* = 2.4, 1.3 Hz, 2H, H–C^{30}), 6.89 (td, *J* = 8.6, 2.2 Hz, 1H, H–C^{19}), 6.87 – 6.85 (m, 4H, H–C^{12}), 6.60 – 6.55 (m, 4H, H–C^{18+32}), 5.85 (ddt, *J* = 17.0, 10.2, 6.7 Hz, 2H, H–C^{35}), 5.14 (dq, *J* = 17.2, 1.6 Hz, 2H, H–C^{36}), 5.08 (dq, *J* = 10.3, 1.2 Hz, 2H, H–C^{36}), 4.14 (ddd, *J* = 11.8, 5.8, 3.1 Hz, 2H, H–C^{15}), 4.04 (ddd, *J* = 11.8, 5.8, 3.1 Hz, 2H, H–C^{15}), 4.05 (t, *J* = 6.1 Hz, 4H, H–C^{20}), 3.98 – 3.85 (m, 8H, H–C^{25+33}), 3.73 (s, 6H, MeO–C^{13}), 3.67 (ddd, *J* = 10.2, 6.7, 3.2 Hz, 2H, H–C^{14}), 3.50 (ddd, *J* = 10.8, 5.8, 3.2 Hz, 2H, H–C^{14}), 3.38 (p, *J* = 6.6 Hz, 2H, H–C^{2}), 3.11 (dd, *J* = 14.8, 6.3 Hz, 2H, H–C^{3}), 2.51 – 2.45 (m, 4H, H–C^{34}), 2.38 (d, *J* = 14.7 Hz, 2H, H–C^{3}), 2.02 (s, 6H, CH_3–C^{5}), 1.83 – 1.72 (m, 8H, H–C^{21+24}), 1.61 (s, 6H, CH_3–C^{8}), 1.59 – 1.45 (m, 8H, H–C^{22+23}), 1.13 (d, *J* = 6.7 Hz, 6H, CH_3–C^{2}) ppm. H–C^{11} is split into two low intensity and/or broad signal(s) due to diastereotopicity and conformational isomerism.

^{13}C{^{1}H} NMR (126 MHz, $CDCl_3$, 25 °C) δ = 166.2 (C^{29}), 160.1 (C^{26}), 159.8 (C^{31}), 158.4 (C^{13}), 153.3 (C^{7}), 148.2 (d, $J_{C–F}$ = 8.4 Hz, C^{17}), 144.1 (d, $J_{C–F}$ = 244.7 Hz, C^{16}), 141.3 (C^{9}), 141.2 (C^{1}), 140.5 (C^{4}), 134.5 (C^{35}), 133.8 (C^{8}), 131.9 (C^{28}), 131.5 (C^{11}), 131.4 ($C^{11`}$), 130.3 (C^{5}), 130.3 (C^{10}), 126.1 (C^{8}), 123.1 (d, $J_{C–F}$ = 5.2 Hz, C^{20}), 117.2 (C^{36}), 113.4 (C^{12}), 108.3 (C^{27}), 108.2 (C^{19}), 107.6 (C^{30}), 106.8 (C^{32}), 69.9 (C^{21}), 69.7 (C^{14}), 68.1 (C^{25}), 67.5 (C^{33}), 64.4 (C^{15}), 55.2 (MeO–C^{13}), 41.9 (C^{2}), 39.3 (C^{3}), 33.7 (C^{34}), 29.1 (C^{21+24}), 25.9 (C^{22}), 25.6 (C^{23}), 20.8 (CH_3–C^{2}), 16.9 (CH_3–C^{5}), 15.1 (CH_3–C^{8}) ppm. C^{11} is split into two low intensity and/or broad signal(s) due to diastereotopicity and conformational isomerism.

^{19}F NMR (471 MHz, $CDCl_3$, 25 °C) δ = –155.9 (t, J = 7.2 Hz, F–C^{16}) ppm.

HR-ESI-TOF-MS (ESI+) *m/z* calculated for $C_{82}H_{94}FO_{14}^{+}$ ($[M+H]^{+}$) 1321.6622, found 1321.6605. Mass error: 1.3 ppm.

3 Winding and Rotation Experiments

General irradiation conditions

Samples of (*Z*s)-**0**, (*Z*s)-**MM**, Bz-(*Z*s)-**MA** or [2]-(*Z*s)-**MR** were irradiated in anhydrous toluene (0.1–0.3 mM) under argon atmosphere. UPLC samples were prepared in a MeCN/IPA (8:2) mixture before being analyzed by UPLC-HRMS. UV-vis spectra were extracted for each isomer and used for characterization.

^{1}H and ^{19}F NMR spectroscopy was performed under argon atmosphere in a J. Young NMR tube containing a 1–2 mM solution of (*Z*s)-**0**, (*Z*s)-**MM**, Bz-(*Z*s)-**MA** or [2]-(*Z*s)-**MR** in toluene-d_8 (0.5 mL). For forward rotations, a Vilber Lourmat (312 nm, 12 W) or a Thorlabs M310L1 (308 nm, 38.5 mW) mounted LED was used. A ThorLabs M405L2 (405 nm, 410 mW) mounted LED was used for irradiations of the metastable states. Low temperature irradiations were performed in an ethanol/dry ice bath (–50 °C) with a distance of 5–10 cm from the light source. Room temperature irradiations were performed at a distance of 1 cm from the light source. Numbering of the atoms was kept consistent with that of the experimental section. Analysis was performed on a Bruker Avance II 600 (600 MHz for ^{1}H) spectrometer at –10 °C and a Bruker Avance II 500 (471 MHz for ^{19}F) spectrometer at 20 °C. In ^{19}F NMR, enriched *E*m states could not be measured due to cooling limitations. Therefore, residual signals after the helix inversion of these states was used for the assignments.

From the stepwise winding sequence of (*Z*s)-**0**, the ^{1}H NMR spectra of newly evolving species offer additional indications for the configuration of the molecular motor double bond. For bis-indanylidene molecular motors, the methyl group in the fjord-region is an excellent diagnostic signal to assign the double bond configuration. In (*Z*)-configurations, this methyl group typically appears between 1.6–1.9 ppm while for (*E*)-configurations the methyl group is significantly upfield-shifted above 2.3 ppm.[13–15]

3.1 Winding of Mechanism of Machine Isomer (*Zs*)-0

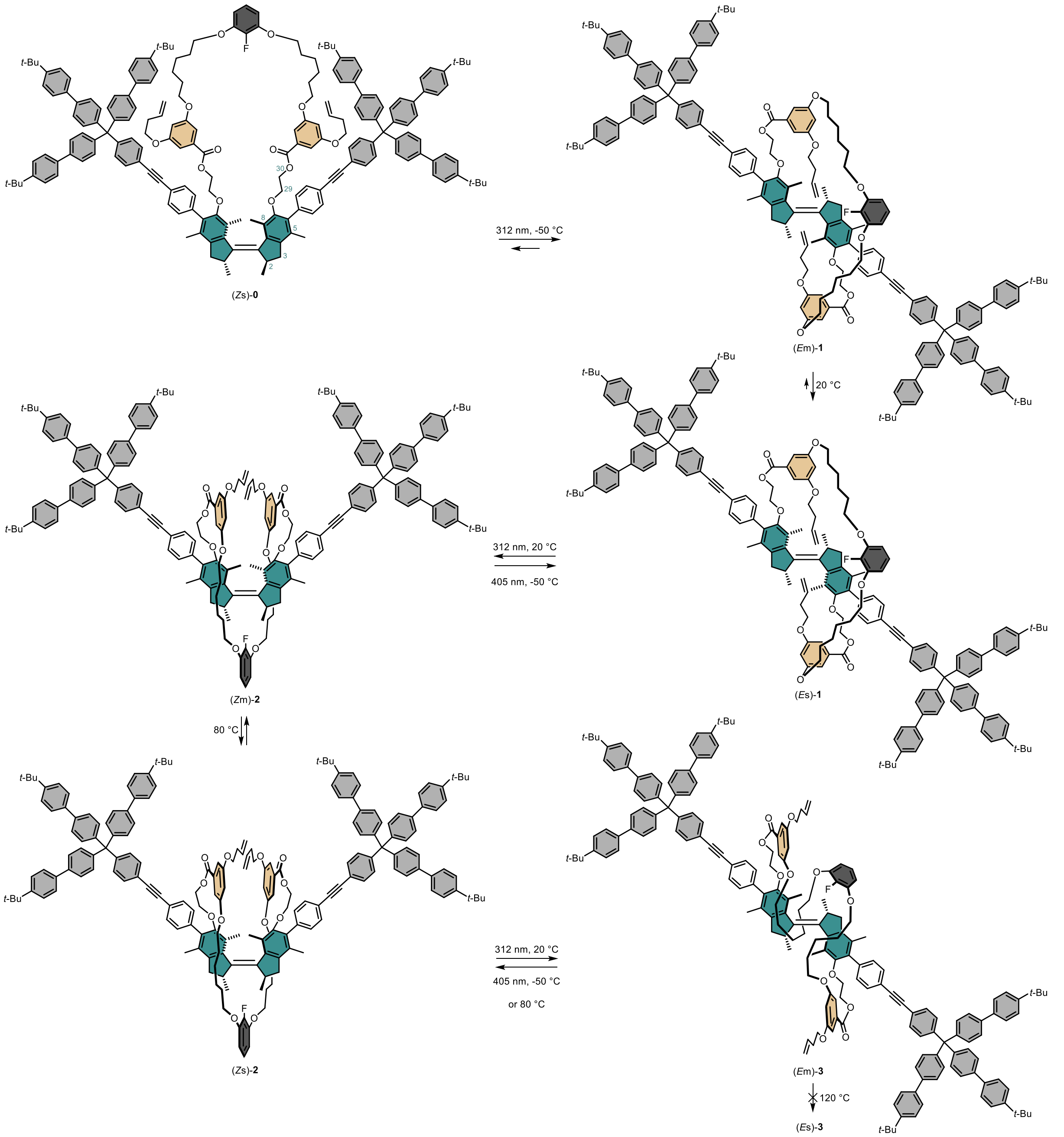

Supporting Figure S6 | Structural depiction of the winding mechanism of machine isomer (*Z*s)-**0**.

UPLC-HRMS Analysis of the Winding Mechanism of Machine Isomer (*Z*s)-0

Supporting Table S1 | Winding sequence of machine isomer (*Z*s)-**0** followed by UPLC-HRMS analysis (BEH phenyl cyclohexyl column, eluted: 98% MeCN/IPA 9:1, 2% H_2O, 0.1% FA, 40 °C). Retention times shown in min and absorption maxima shown in nm.

	(*Z*s)-**0**	(*E*s)-**1**	(*Z*m)-**2**	(*Z*s)-**2**	(*E*m)-**3**
**Retention time [min]**	3.24	2.48	2.92	1.52	2.62
**A_{max} [nm]**	346	332	366	350	362

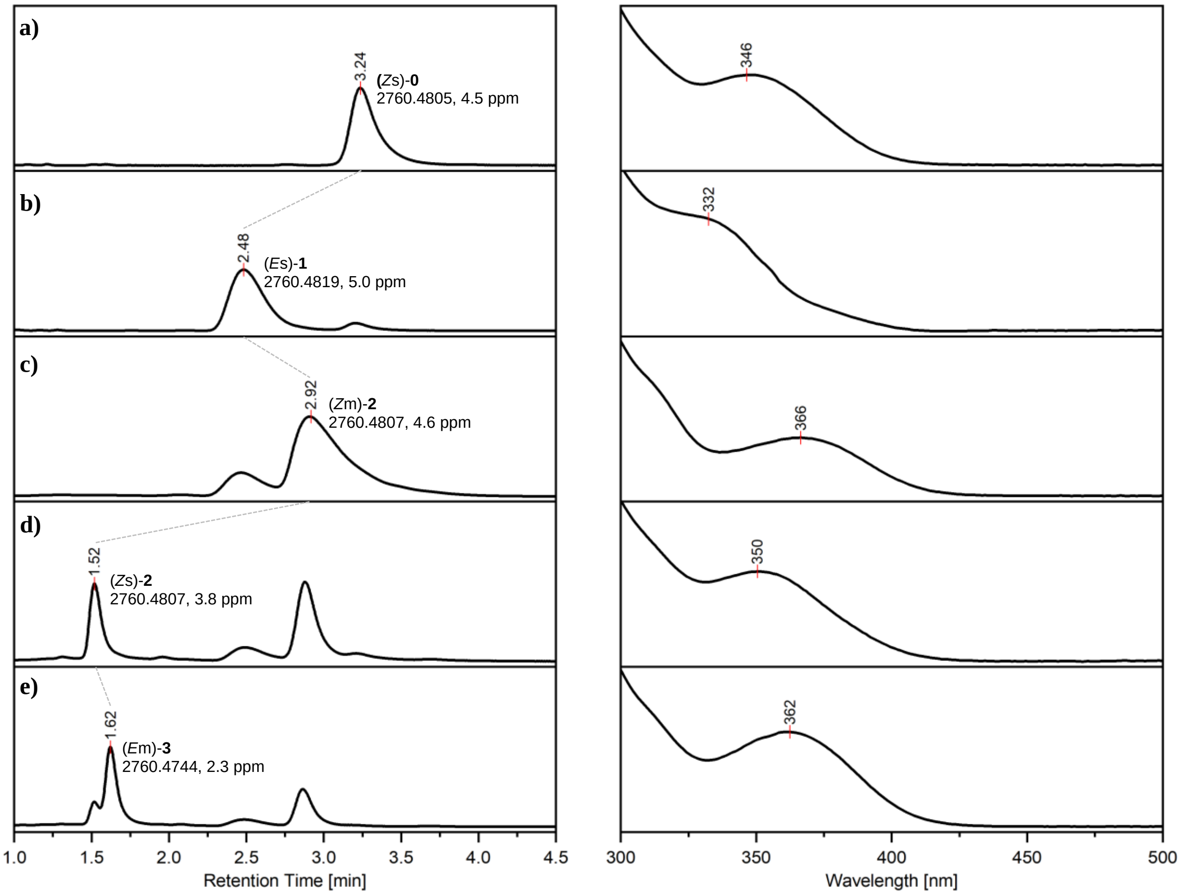

Supporting Figure S7 | UPLC-HRMS analysis of the winding mechanism of (*Z*s)-**0** (BEH phenyl cyclohexyl column, eluent: 98% MeCN/IPA 9:1, 2% H_2O, 0.1% FA, 40 °C). Left: UPLC chromatograms (absorption at 270 nm), highlighted peak indicates the newly formed species after each step. Masses are given as the sodium adduct ($[M+Na]^+$). Right: normalized UV-vis spectrum from the respective highlighted peak on the left. a) Initial (*Z*s)-**0** chromatogram, b) after irradiation with 312 nm at –50 °C and subsequent thermal relaxation at 20 °C, c) after irradiation with 312 nm at 20 °C, d) after thermal relaxation at 80 °C, e) after irradiation with 308 nm at 120 °C.

^{1}H NMR Analysis of the Winding Mechanism of Machine Isomer (*Z*s)-0

Supporting Table S2 | Chemical shifts (ppm) of characteristic proton signals during the winding sequence of machine isomer (*Z*s)-**0** (600 MHz, toluene-*d*$_8$, *c* = 1.5 mM, –10 °C). Zoomed-in spectra of the characteristic signals are shown on page 48. ^{1}H COSY and ^{1}H ROESY NMR was used for the assignment of the protons, see page 49 to 52.

	(*Z*s)-**0**	(*E*m)-**1**	(*E*s)-**1**	(*Z*m)-**2**	(*Z*s)-**2**	(*E*m)-**3**
**CH_3–C^2**	1.18	1.01	overlap	1.39	1.20	overlap
**CH_3–C^5**	2.04	2.02	2.05	2.06	2.20	2.37
**CH_3–C^8**	2.01	2.34	2.61	1.75	1.97	2.46
**H–C^2**	3.36	3.12	3.00	3.42	overlap	overlap
**H–C^3**	2.40, 3.10	2.56, 2.94	2.53, overlap	2.54, 2.98	overlap	overlap

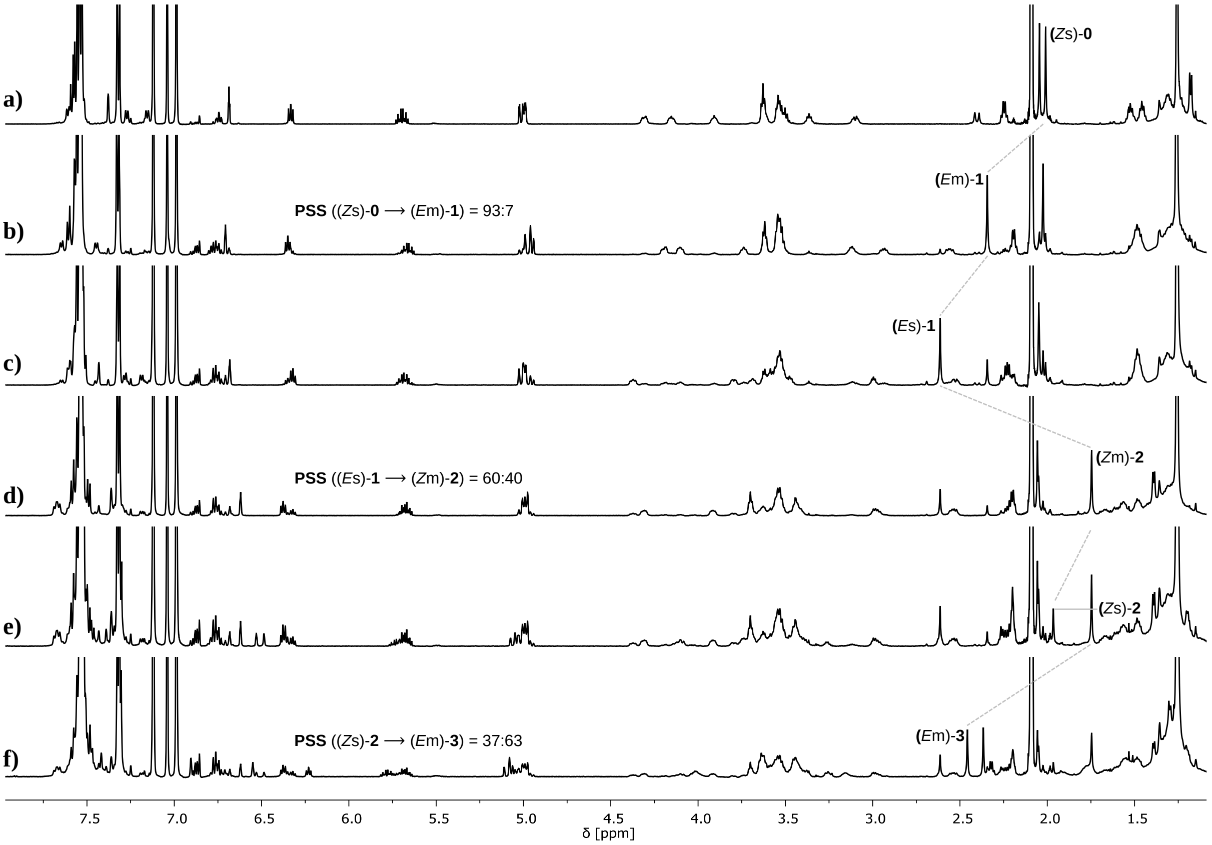

Supporting Figure S8 | ^{1}H NMR spectra (600 MHz, toluene-*d*$_8$, *c* = 1.5 mM, –10 °C) of the winding sequence of machine isomer (*Z*s)-**0**, sequence from top to bottom. a) Initial (*Z*s)-**0** spectrum, b) after irradiation with 312 nm at –50 °C, c) after thermal relaxation at 20 °C, d) after irradiation with 312 nm at 20 °C, e) after thermal relaxation at 80 °C, f) after irradiation with 308 nm at 120 °C.

Zoomed in ^{1}H NMR Spectra of the Winding Sequence

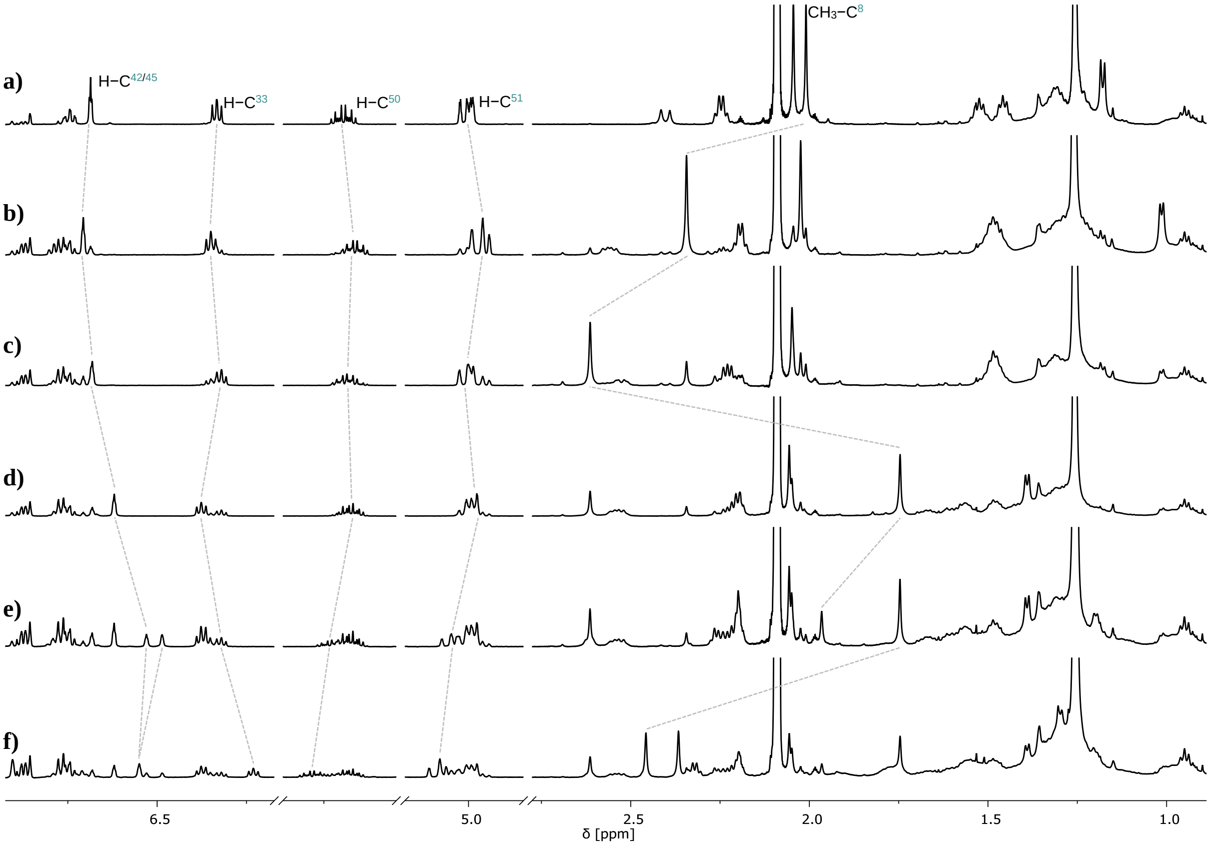

Supporting Figure S9 | Partial ^{1}H NMR spectra (600 MHz, toluene-d_8, c = 1.5 mM, –10 °C) of the winding sequence of machine isomer (*Z*s)-**0**, sequence from top to bottom. a) Initial (*Z*s)-**0** spectrum, b) after irradiation with 312 nm at –50 °C, c) after thermal relaxation at 20 °C, d) after irradiation with 312 nm at 20 °C, e) after thermal relaxation at 80 °C, f) after irradiation with 308 nm at 120 °C.

^{1}H COSY and ^{1}H ROESY NMR Analysis of the Wound Isomers of (*Z*s)-0

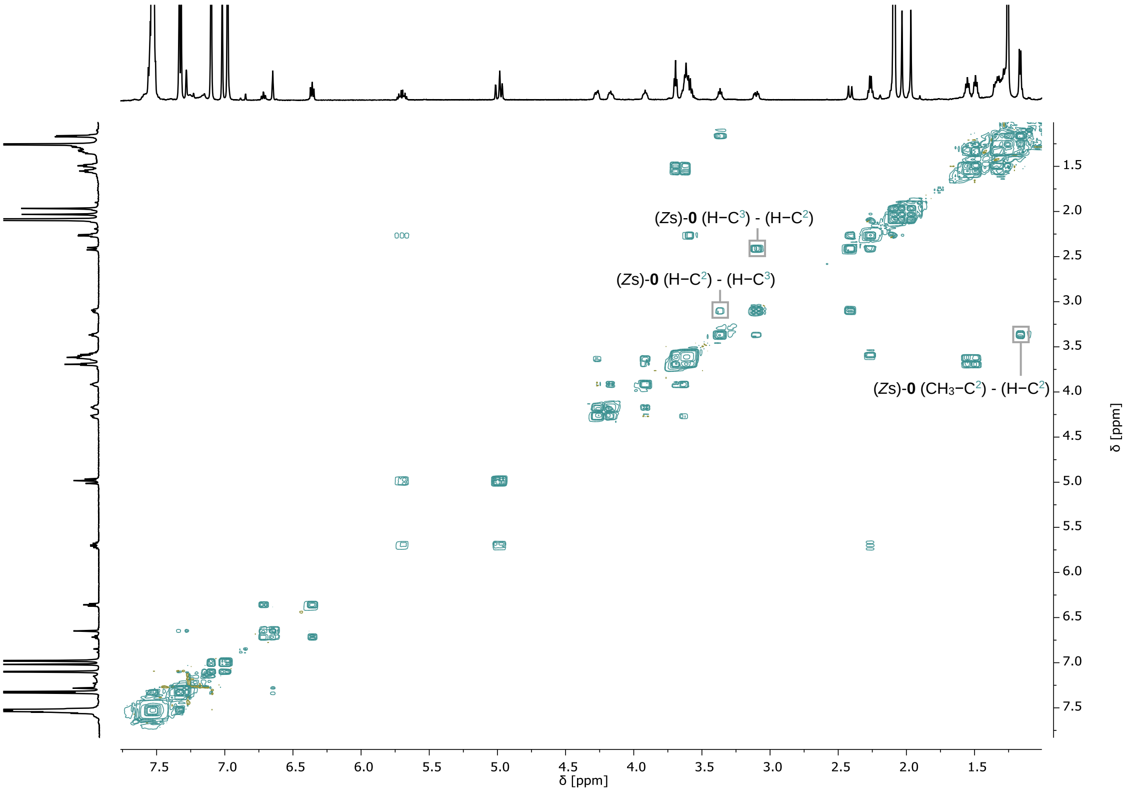

Supporting Figure S10 | ^{1}H COSY NMR of machine isomer (*Z*s)-**0** (600 MHz, toluene-d_8, c = 1.5 mM, 25 °C). Cross-peaks were used to assign protons CH_3–C[2], H–C[2] and H–C[3].

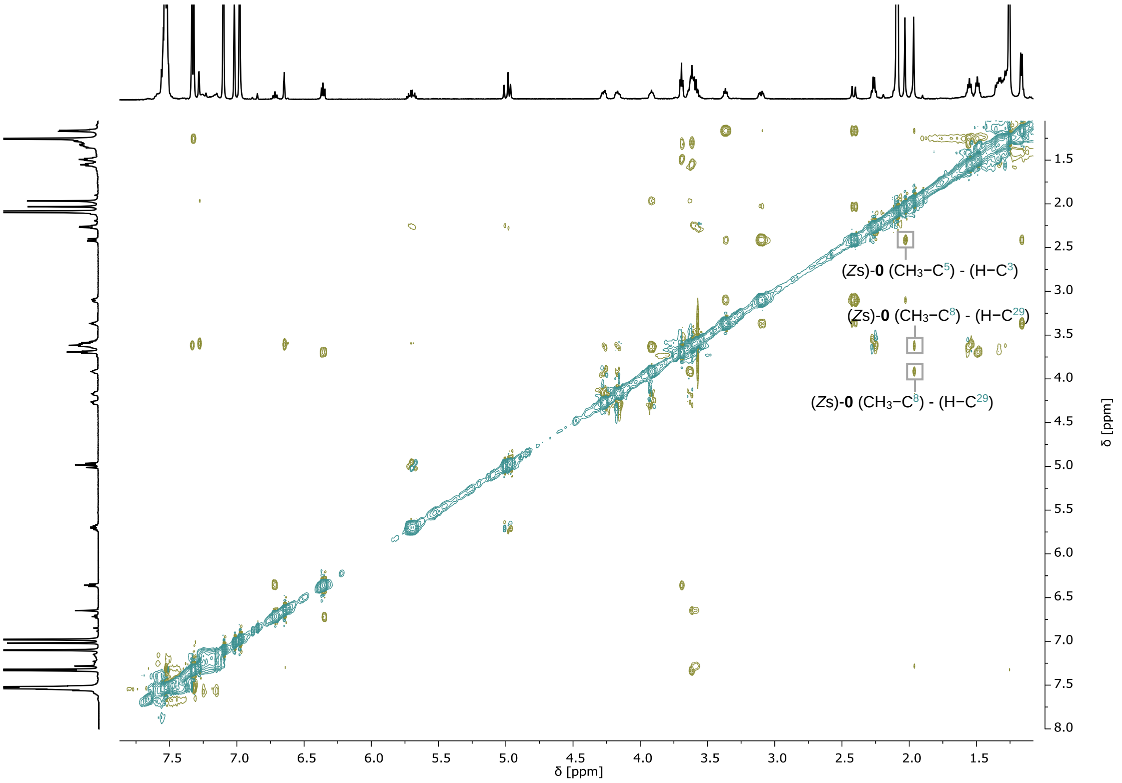

Supporting Figure S11 | ^{1}H ROESY NMR of machine isomer (*Z*s)-**0** (600 MHz, toluene-d_8, c = 1.5 mM, 25 °C). Cross-peaks were used to assign protons CH_3–C[5], CH_3–C[8] and H–C[29].

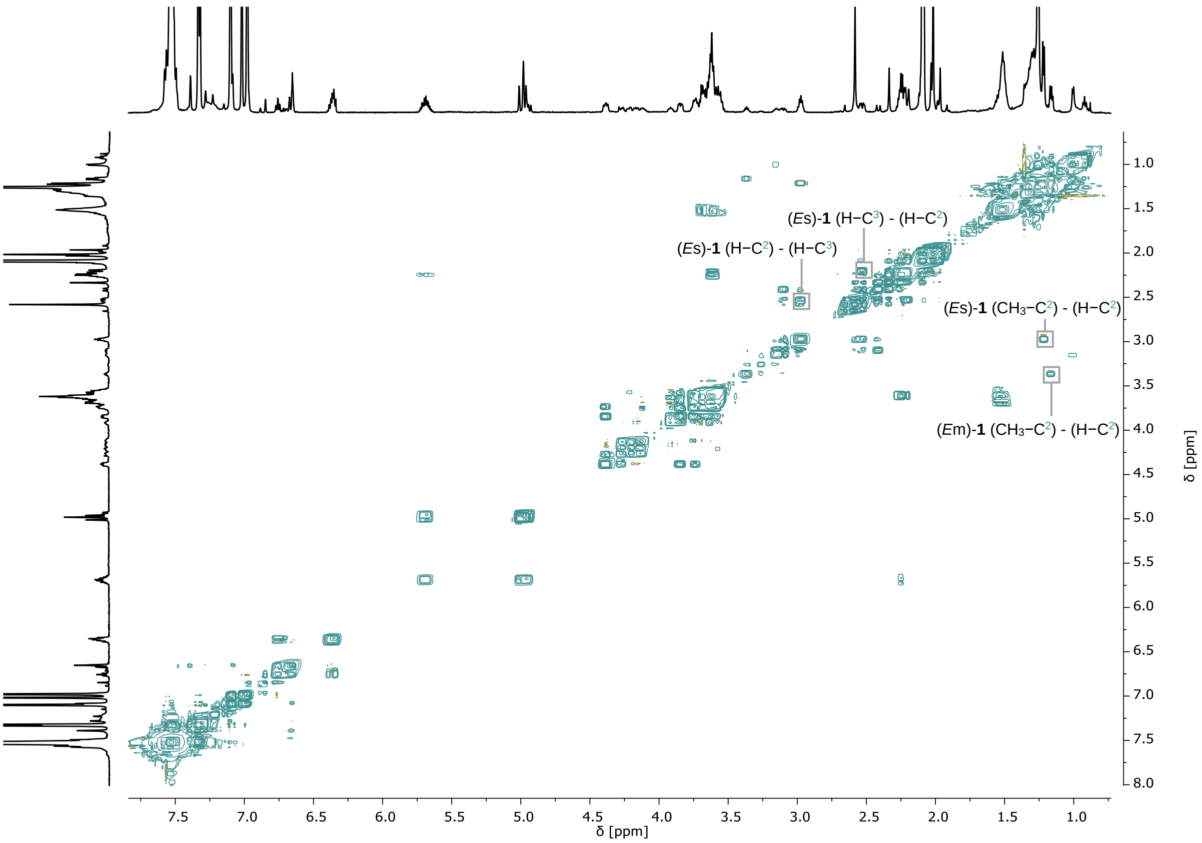

Supporting Figure S12 | ^{1}H COSY NMR of machine isomers (*E*m)-**1**/(*E*s)-**1** (600 MHz, toluene-*d*$_8$, *c* = 1.5 mM, 25 °C). Cross-peaks were used to assign protons CH_3−C^2, H−C^2 and H−C^3.

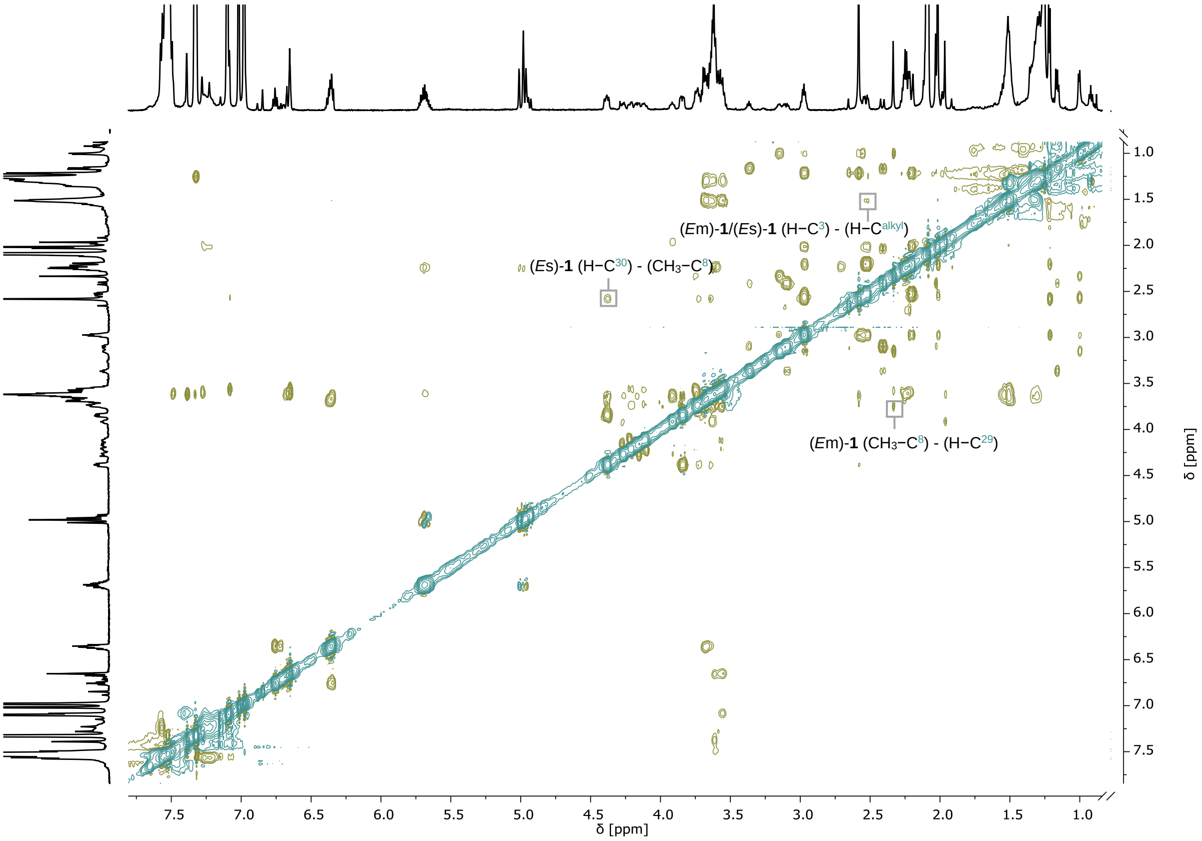

Supporting Figure S13 | ^{1}H ROESY NMR of machine isomers (*E*m)-**1**/(*E*s)-**1** (600 MHz, toluene-*d*$_8$, *c* = 1.5 mM, 25 °C). Cross-peaks were used to assign protons CH_3−C^5, CH_3−C^8, H−C^{29} and H−C^{30}. Additionally, new cross-peaks formed between H−C^3 and the alkyl protons in the tether were observed not seen in machine isomer (*Z*s)-**0**.

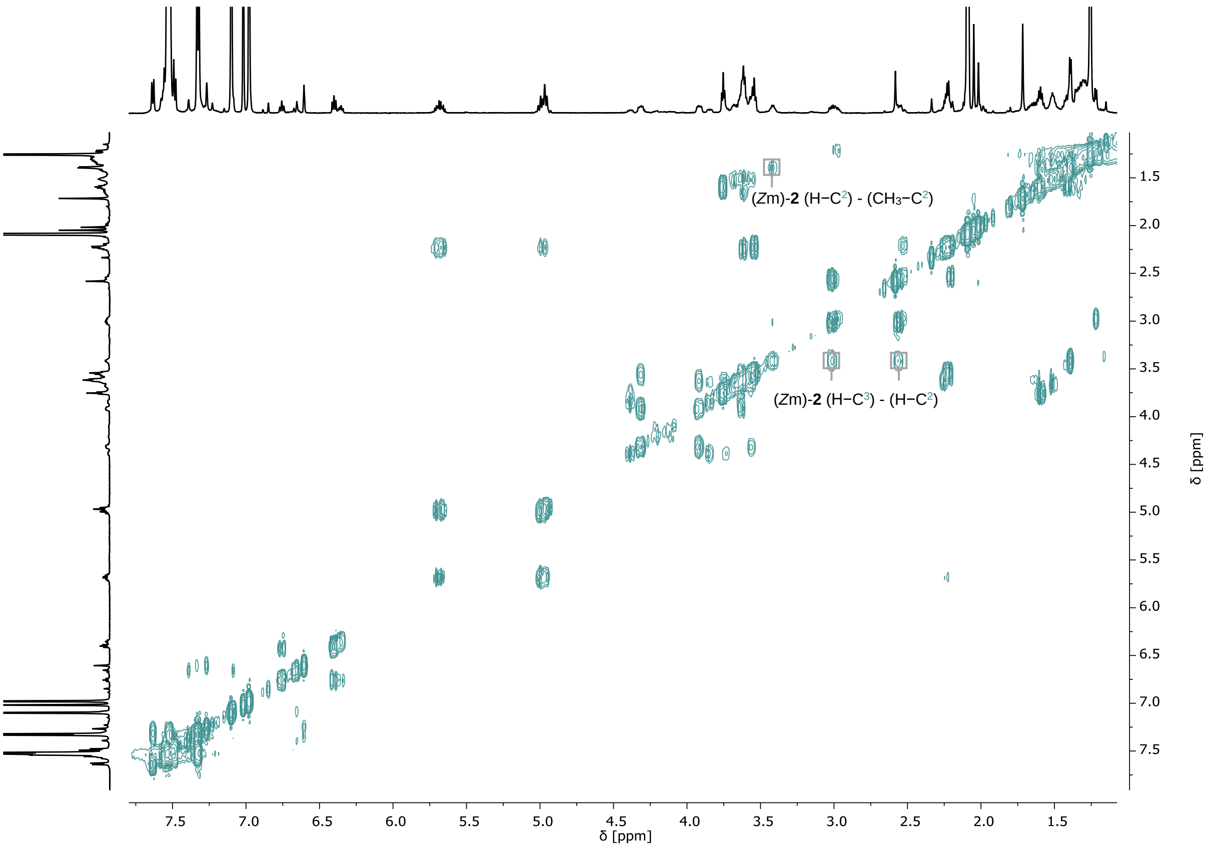

Supporting Figure S14 | ^{1}H COSY NMR of machine isomer (Zm)-**2** (600 MHz, toluene-d_8, c = 1.5 mM, 25 °C). Cross-peaks were used to assign protons CH_3−C^2, H−C^2 and H−C^3.

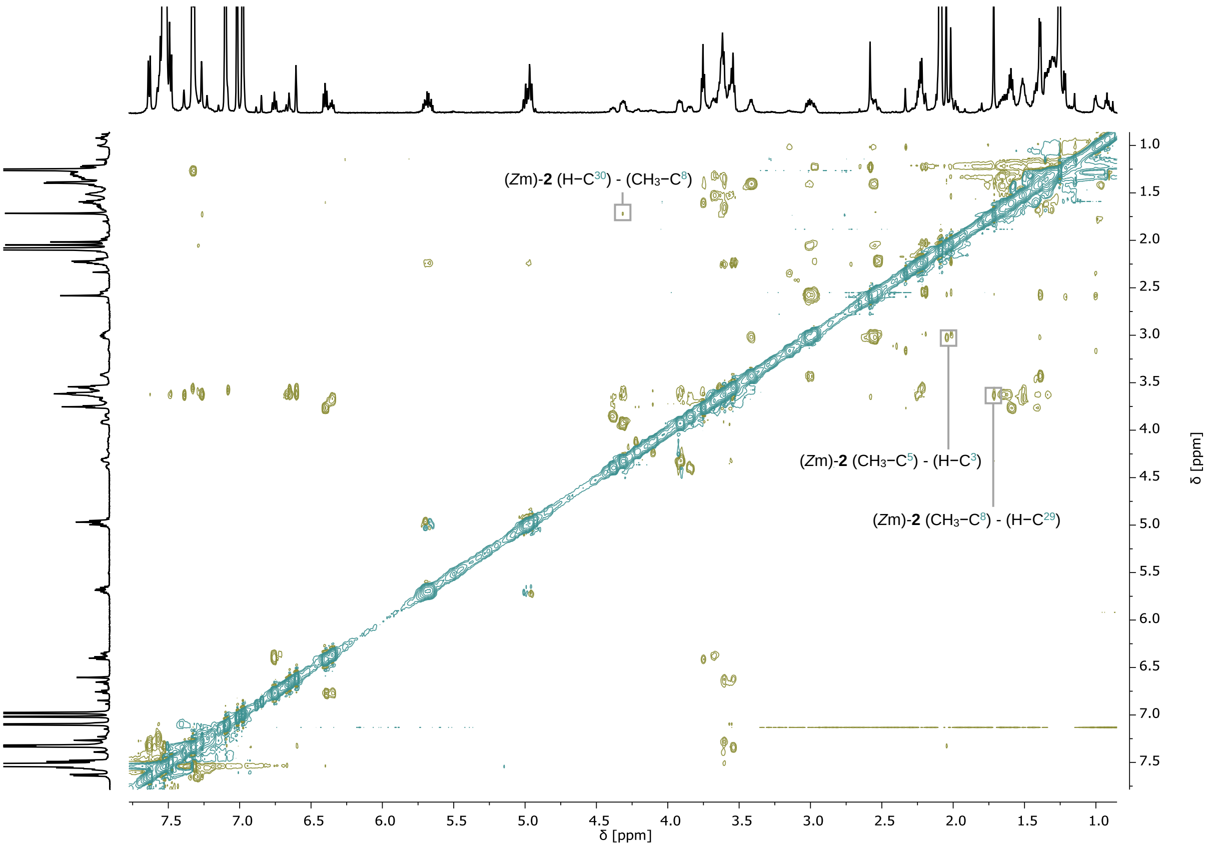

Supporting Figure S15 | ^{1}H ROESY NMR of machine isomer (Zm)-**2** (600 MHz, toluene-d_8, c = 1.5 mM, 25 °C). Cross-peaks were used to assign protons CH_3−C^5, CH_3−C^8, H−C^{29} and H−C^{30}.

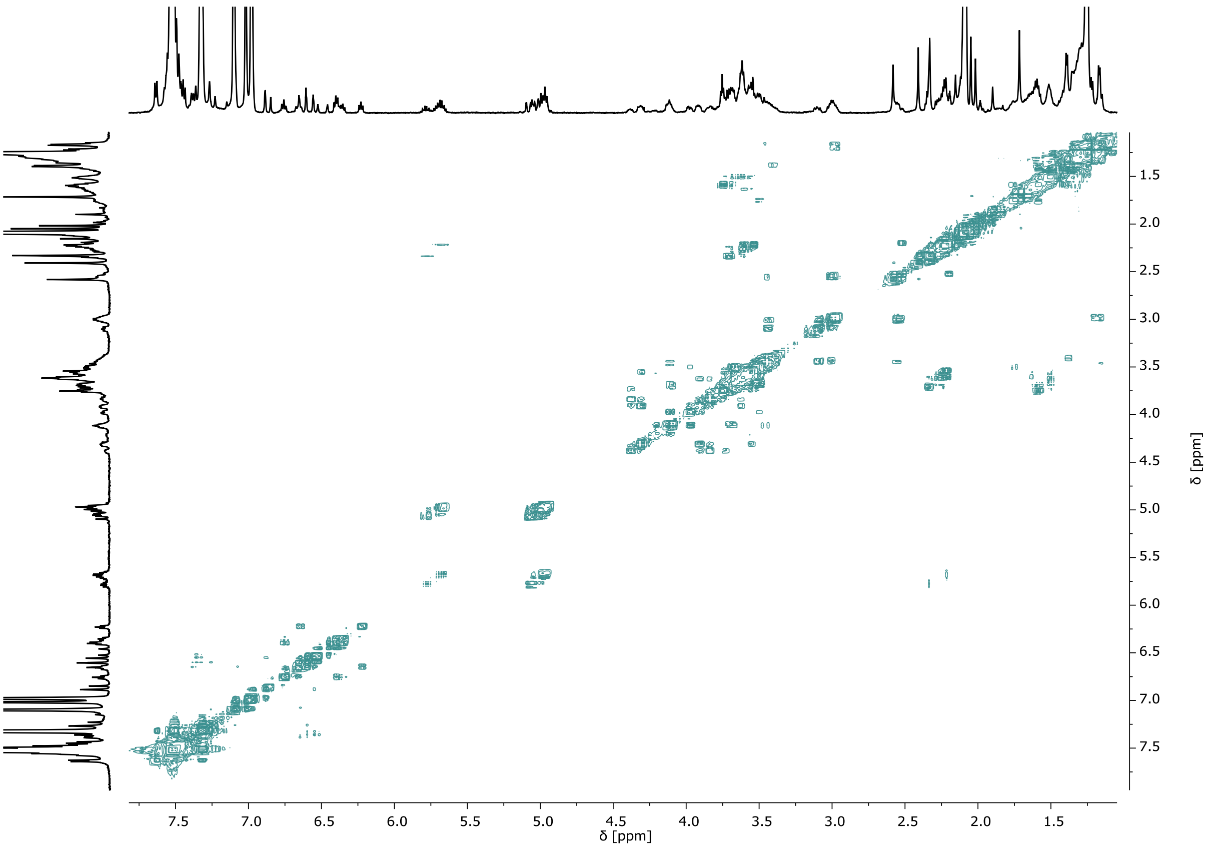

Supporting Figure S16 | ^{1}H COSY NMR of machine isomers (*Z*s)-**2** and (*E*m)-**3** (600 MHz, toluene-d_8, c = 1.5 mM, 25 °C).

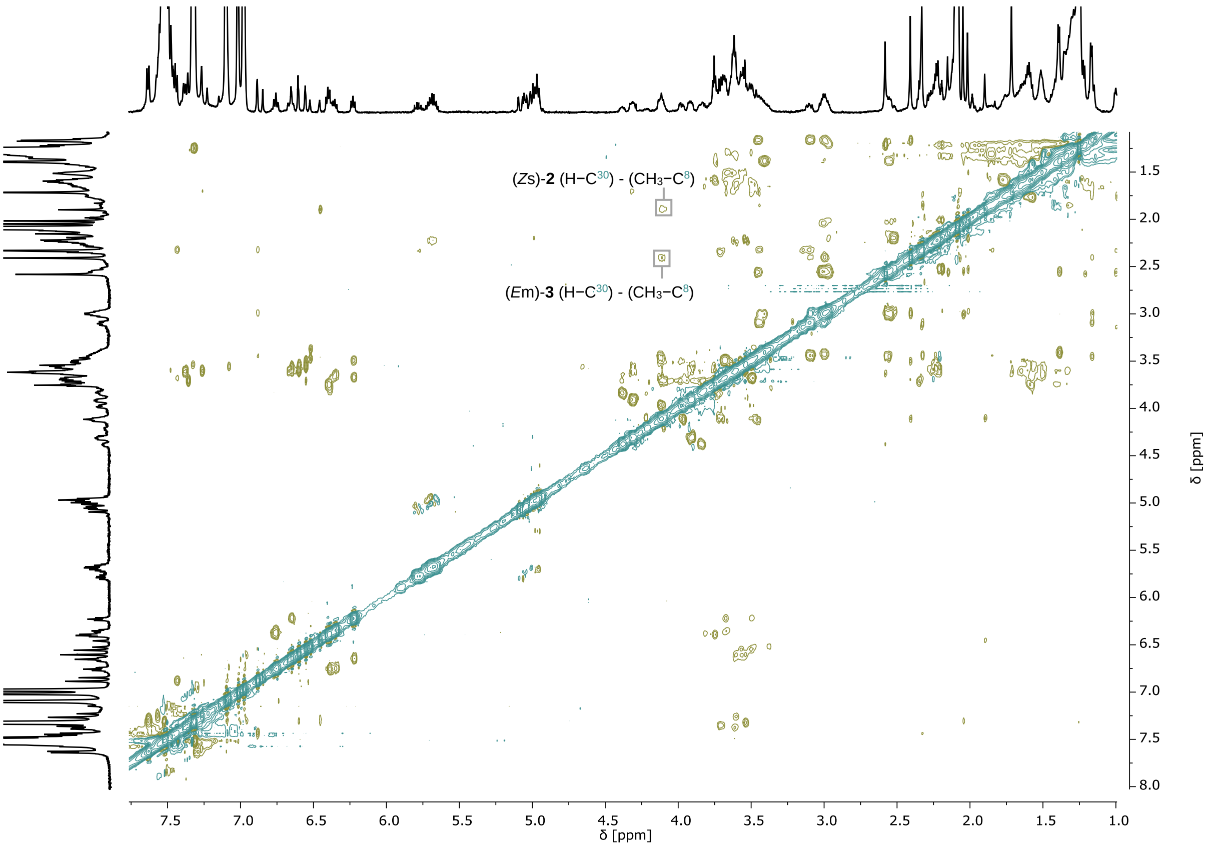

Supporting Figure S17 | ^{1}H ROESY NMR of machine isomers (*Z*s)-**2** and (*E*m)-**3** (600 MHz, toluene-d_8, c = 1.5 mM, 25 °C). Cross-peaks were used to assign protons $CH_3{-}C^5$, $CH_3{-}C^8$ and $H{-}C^{30}$.

^{19}F NMR Analysis of the Winding Mechanism of Machine Isomer (*Z*s)-0

Supporting Table S3 | Chemical shifts (ppm) of the fluorine probe during the winding sequence of machine isomer (*Z*s)-**0** (471 MHz, toluene-d_8, c = 1.5 mM, 25 °C).

	(*Z*s)-**0**	(*E*m)-**1**	(*E*s)-**1**	(*Z*m)-**2**	(*Z*s)-**2**	(*E*m)-**3**
**F–C**[31]	–155.04	–155.90	–156.07	–154.80	–154.72	–155.34

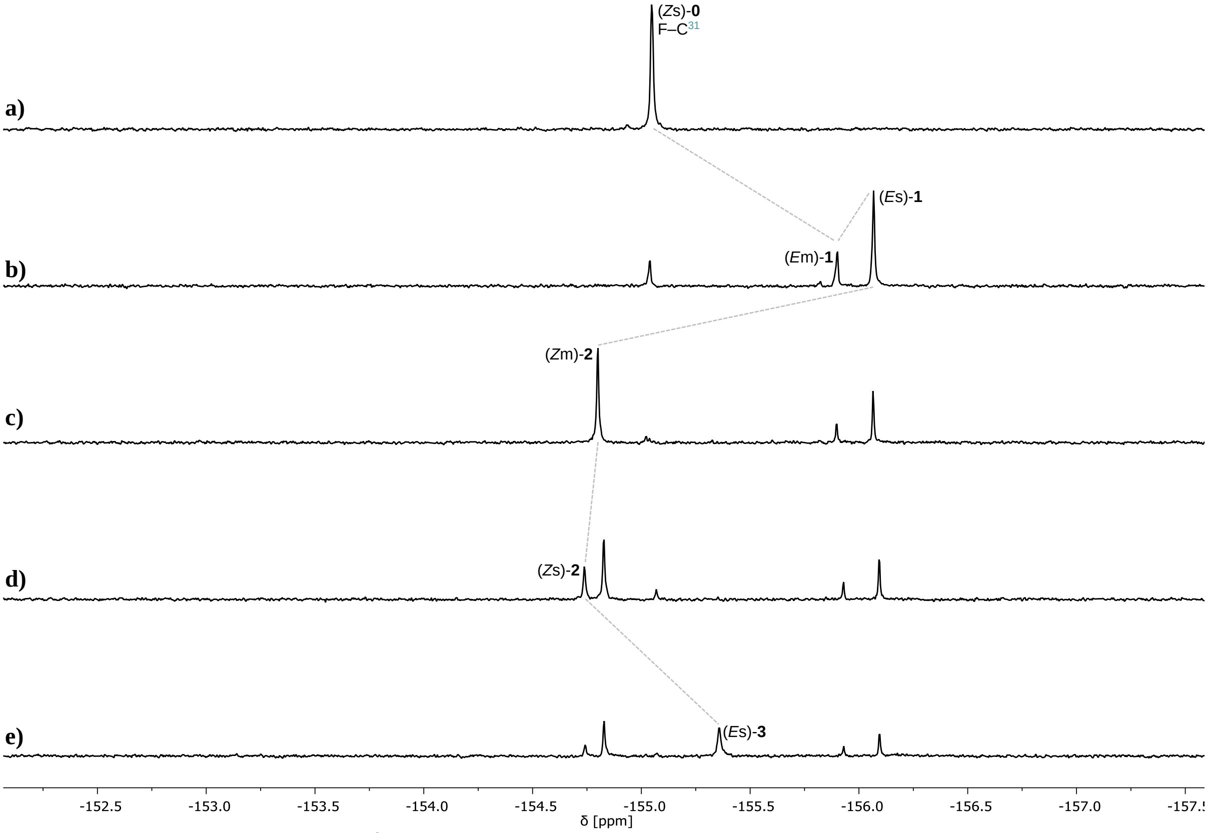

Supporting Figure S18 | Partial ^{19}F{^{1}H} NMR spectra (471 MHz, toluene-d_8, c = 1.5 mM, 25 °C) of the winding sequence of machine isomer (*Z*s)-**0**, sequence from top to bottom. a) Initial (*Z*s)-**0** spectrum, b) after irradiation with 312 nm at –50 °C and subsequent thermal relaxation at 20 °C, c) after irradiation with 312 nm at 20 °C, d) after thermal relaxation at 80 °C, e) after irradiation with 308 nm at 120 °C.

Thermal- and Photochemical Back Isomerization of Machine Isomer (*E*m)-3

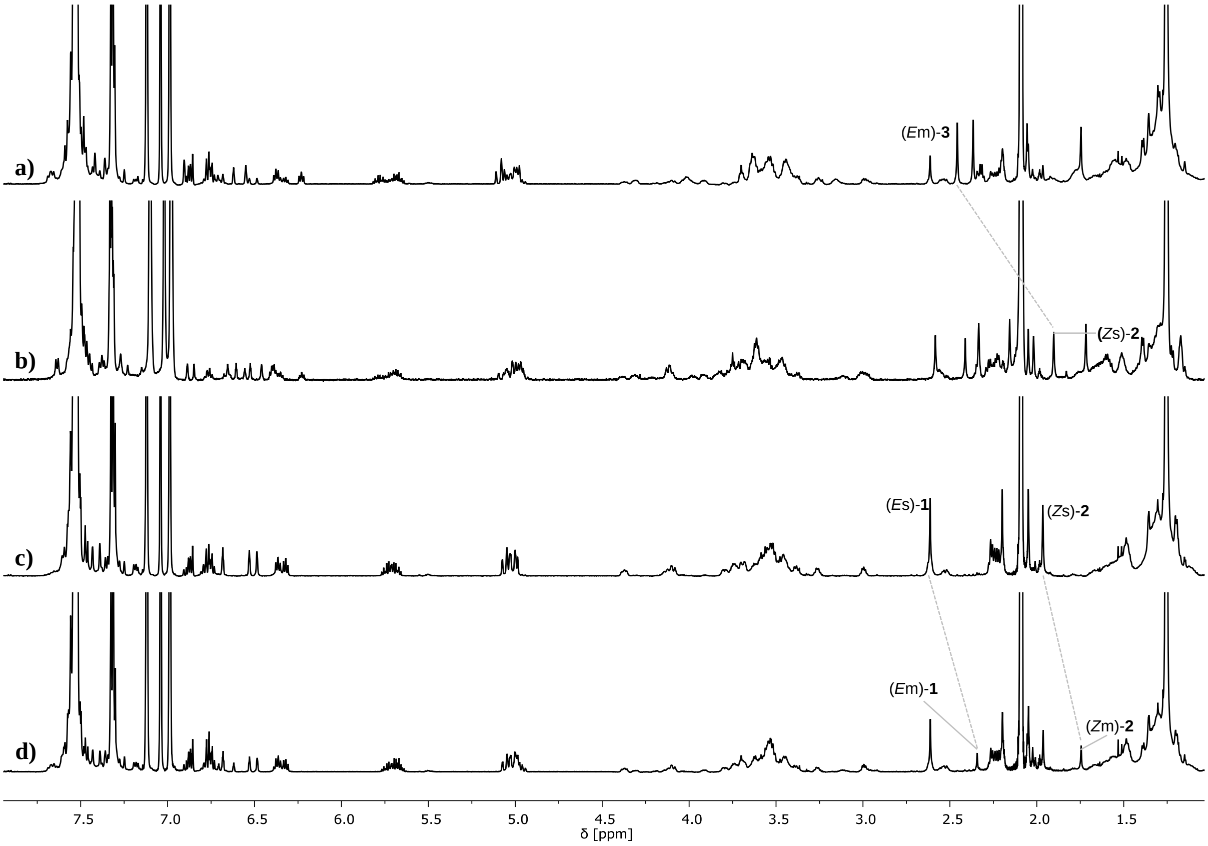

Supporting Figure S19 | ^{1}H NMR spectra (600 MHz, toluene-d_8, c = 1.5 mM, –10 °C) of the back isomerization of machine isomer (*E*m)-**3**. a) Initial machine isomer (*E*m)-**3**. b) After thermal back isomerization of the (*E*m)-**3** at 80 °C. Forming the equilibrium between machine isomer (*Z*m)-**2** and (*Z*s)-**2** (measured at 25 °C). c) After back irradiation of machine isomer (*E*m)-**3** with 405 nm light at –50 °C. Note that machine isomer (*Z*m)-**2** also isomerizes back to (*E*s)-**1**, as well as machine isomer (*E*m)-**1** that isomerizes back to (*Z*s)-**0** creating a mixture of only stable isomers. d) After thermal equilibration at 80 °C of machine isomer (*Z*s)-**2** forming partially machine isomer (*Z*m)-**2**. Additionally, the equilibrium between machine isomers (*E*m)-**1** and (*E*s)-**1** is restored as well.

3.2 Rotational Cycle of Control Bz-(*Z*s)-MA

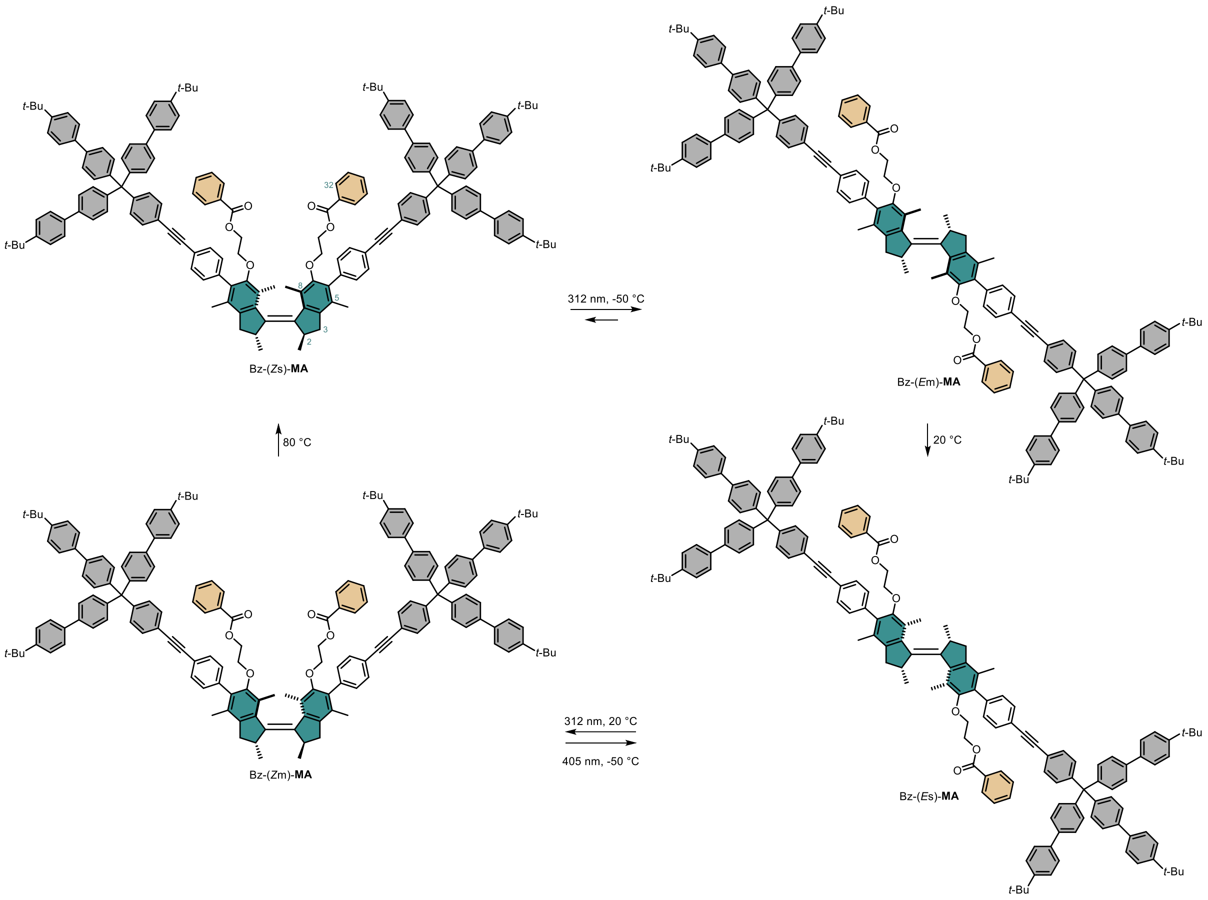

Supporting Figure S20 | Structural depiction of the rotational cycle of control Bz-(*Z*s)-**MA**.

UPLC-HRMS Analysis of the Rotation of Bz-(*Z*s)-MA

Supporting Table S4 | Rotational cycle of Bz-(*Z*s)-**MA** followed by UPLC-HRMS (BEH phenyl cyclohexyl column, eluted: 98% MeCN/IPA 9:1, 2% H_2O, 0.1% FA, 40 °C). Retention times shown in min and absorption maxima shown in nm.

	Bz-(*Z*s)-**MA**	Bz-(*E*s)-**MA**	Bz-(*Z*m)-**MA**
**Retention time [min]**	2.10	1.58	2.29
**A_{max} [nm]**	346	323	364

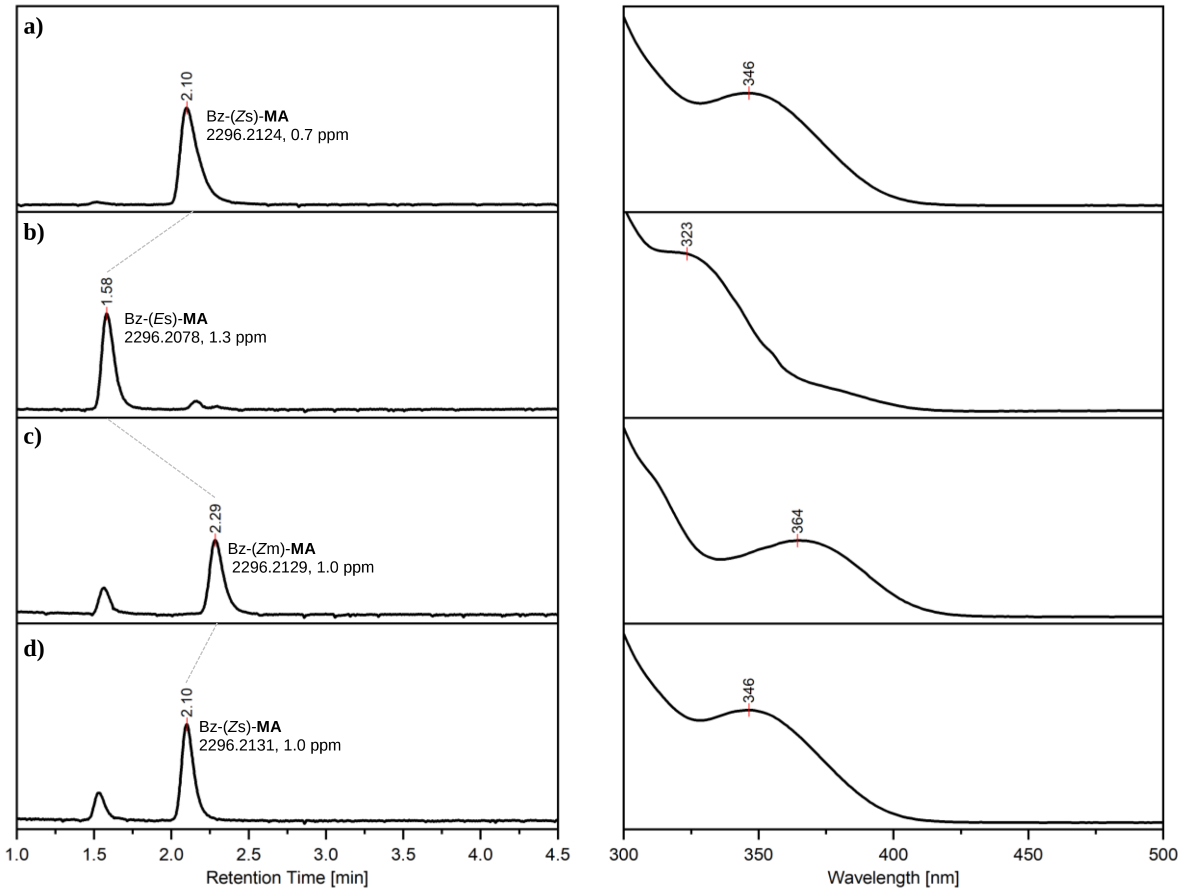

Supporting Figure S21 | UPLC-HRMS analysis of the rotational cycle of Bz-(*Z*s)-**MA** (BEH phenyl cyclohexyl column, eluent: 98% MeCN/IPA 9:1, 2% H_2O, 0.1% FA, 40 °C). Left: UPLC chromatograms (diode array), highlighted peak indicates the newly formed species after each step. Masses are given as the sodium adduct ($[M+Na]^+$). Right: extracted UV-vis spectrum from the respective highlighted peak of the left chromatogram. a) Initial Bz-(*Z*s)-**MA** chromatogram, b) after irradiation with 312 nm at –50 °C and subsequent thermal relaxation at 20 °C, c) after irradiation with 312 nm at 20 °C, d) after thermal relaxation at 80 °C.

^{1}H NMR Analysis of the Rotation of benzoylated (*Z*s)-MA

Supporting Table S5 | Chemical shifts (ppm) of characteristic proton signals during the rotation of Bz-(*Z*s)-**MA** (600 MHz, toluene-d_8, c = 2 mM, –10 °C).

	Bz-(*Z*s)-**MA**	Bz-(*E*m)-**MA**	Bz-(*E*s)-**MA**	Bz-(*Z*m)-**MA**
**CH_3–C^2**	1.17	0.99	1.24 (overlap)	1.40
**CH_3–C^5**	2.03	2.03	1.92	2.00
**CH_3–C^8**	1.95	2.31	2.62	1.78
**H–C^2**	3.35	3.12	3.13	3.42
**H–C^3**	2.38, 3.05	2.55, 2.92	2.24, 2.70	2.56, 2.94

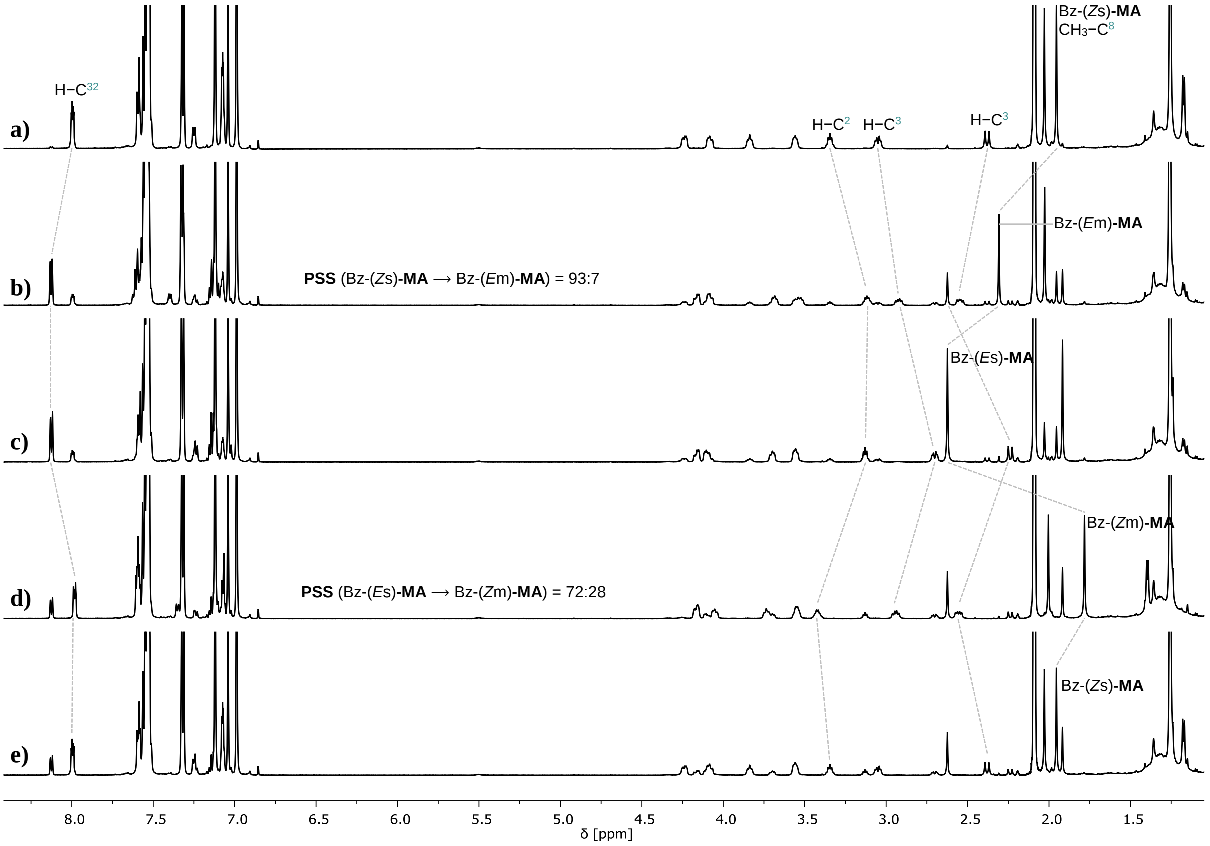

Supporting Figure S22 | ^{1}H NMR spectra (600 MHz, toluene-d_8, c = 2 mM, –10 °C) of the rotational cycle of Bz-(*Z*s)-**MA**, sequence from top to bottom. a) Initial Bz-(*Z*s)-**MA** spectrum, b) after irradiation with 312 nm at –50 °C, c) after thermal relaxation at 20 °C, d) after irradiation with 312 nm at 20 °C, e) after thermal relaxation at 80 °C.

3.3 Rotational Cycle of (*Z*s)-MM

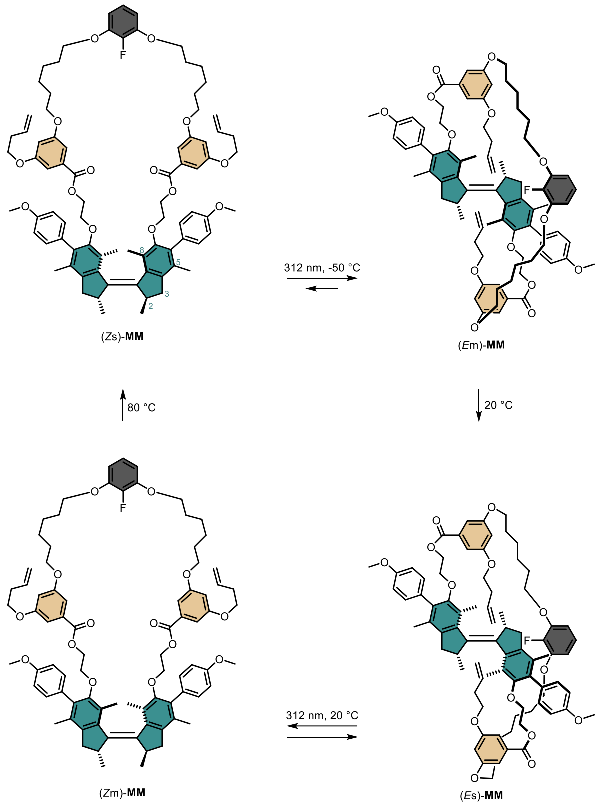

Supporting Figure S23 | Structural depiction of the rotational cycle of control (*Z*s)-**MM**. The tether skips over the motor core after the first thermal helix inversion cancelling out the formed pseudo-crossing.

UPLC-HRMS Analysis of the rotation of Control (*Z*s)-MM

Supporting Table S6 | Rotational cycle of (*Z*s)-**MM** followed by UPLC-HRMS (BEH phenyl cyclohexyl column, eluted: 90% MeCN/IPA 9:1, 10% H_2O, 0.1% FA, 40 °C). Retention times shown in min and absorption maxima shown in nm.

	(*Z*s)-**MM**	(*E*s)-**MM**	(*Z*m)-**MM**
**Retention time [min]**	2.01	1.67	2.13
**A_{max} [nm]**	327	317	361

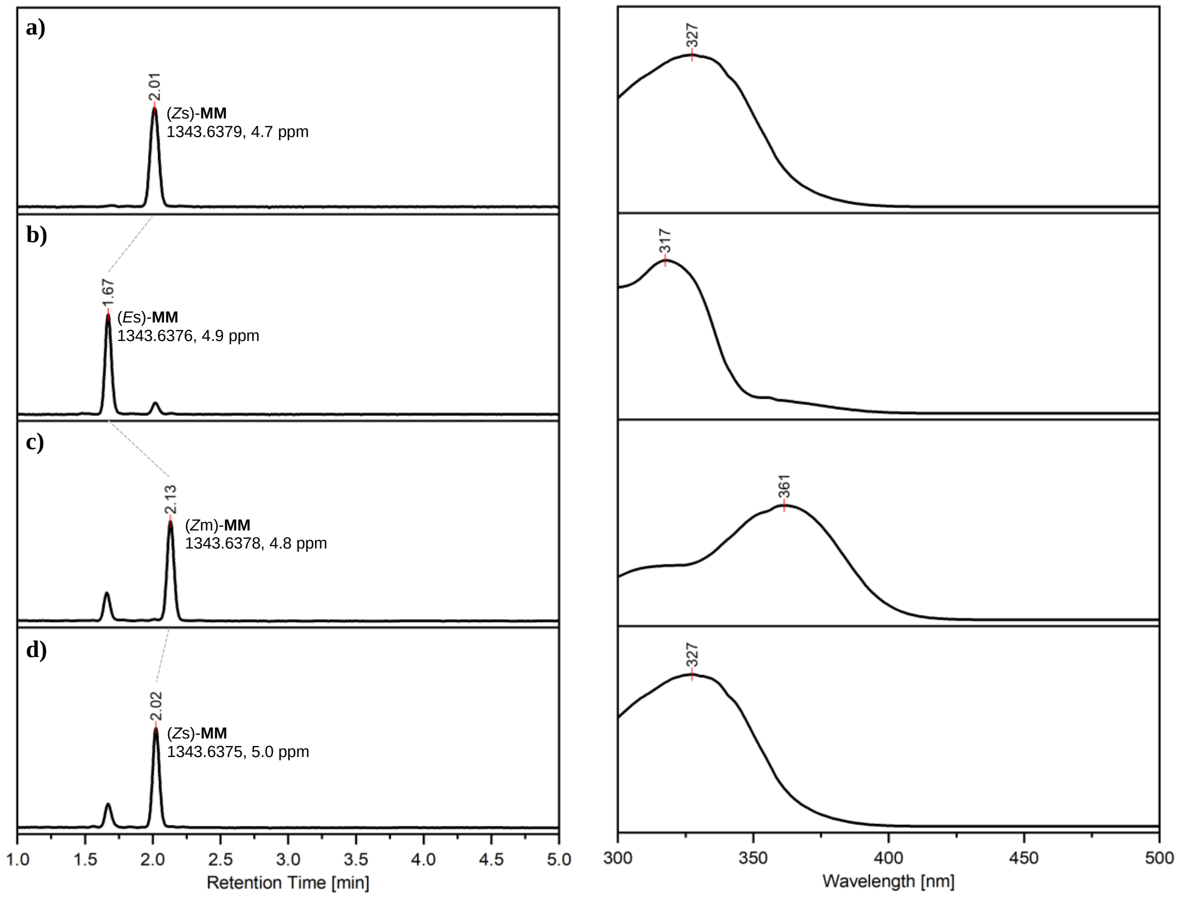

Supporting Figure S24 | UPLC-HRMS analysis of the rotational cycle of control (*Z*s)-**MM** (BEH phenyl cyclohexyl column, eluent: 90% MeCN/IPA 9:1, 10% H_2O, 0.1% FA, 40 °C). Left: UPLC chromatograms (diode array), highlighted peak indicates the newly formed species after each step. Masses are given as the sodium adduct ($[M+Na]^+$). Right: extracted UV-vis spectrum from the respective highlighted peak of the left chromatogram. a) Initial (*Z*s)-**MM** chromatogram, b) after irradiation with 312 nm at –50 °C and subsequent thermal relaxation at 20 °C, c) after irradiation with 312 nm at 20 °C, d) after thermal relaxation at 80 °C.

^{1}H NMR Analysis of the Rotation of (*Z*s)-MM

Supporting Table S7 | Chemical shifts (ppm) of characteristic proton signal during the rotation of control (*Z*s)-**MM** (600 MHz, toluene-d_8, c = 2 mM, –10 °C).

	(*Z*s)-**MM**	(*E*m)-**MM**	(*E*s)-**MM**	(*Z*m)-**MM**
**CH_3–C^2**	1.19	1.04	1.26	1.44
**CH_3–C^5**	2.16	2.13	2.04	2.13
**CH_3–C^8**	2.04	2.39	2.72	1.85
**H–C^2**	3.38	3.16	3.16	3.46
**H–C^3**	2.44, 3.14	2.61, 2.98	2.28, 2.74	2.64, 3.01
**MeO–C^{13}**	3.34	3.34	3.32	3.36

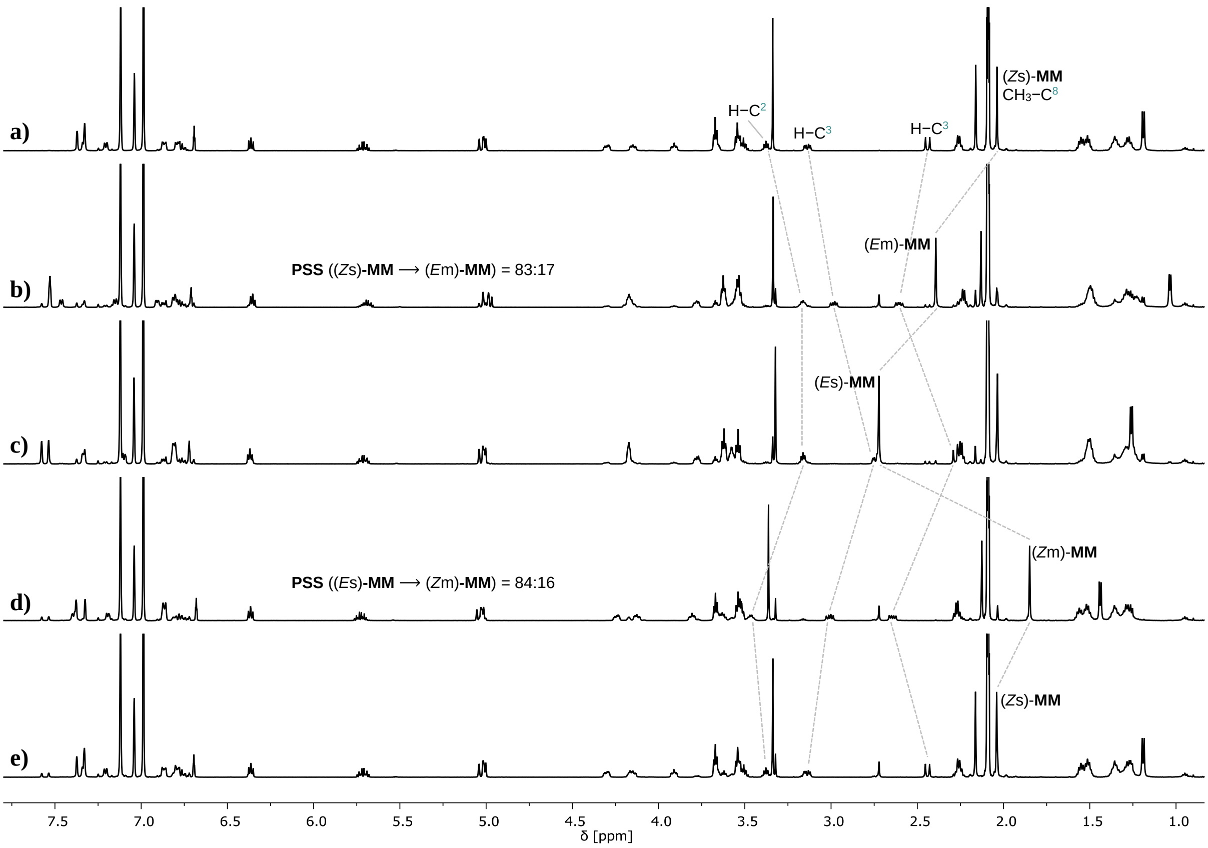

Supporting Figure S25 | ^{1}H NMR spectra (600 MHz, toluene-d_8, c = 2 mM, –10 °C) of the rotational cycle of (*Z*s)-**MM**. Sequence from top to bottom. a) Initial (*Z*s)-**MM** spectrum, b) after irradiation with 312 nm at –50 °C, c) after thermal relaxation at 20 °C, d) after irradiation with 312 nm at 20 °C, e) after thermal relaxation at 80 °C.

^{19}F NMR Analysis of the Rotation of Control (*Z*s)-MM

Supporting Table S8 | Chemical shifts (ppm) of the fluorine probe during the rotation of control (*Z*s)-**MM** (471 MHz, toluene-d_8, c = 2 mM, 25 °C).

	(*Z*s)-**MM**	(*E*m)-**MM**	(*E*s)-**MM**	(*Z*m)-**MM**
**F–C**[16]	–155.26	–155.88	–155.83	–155.31

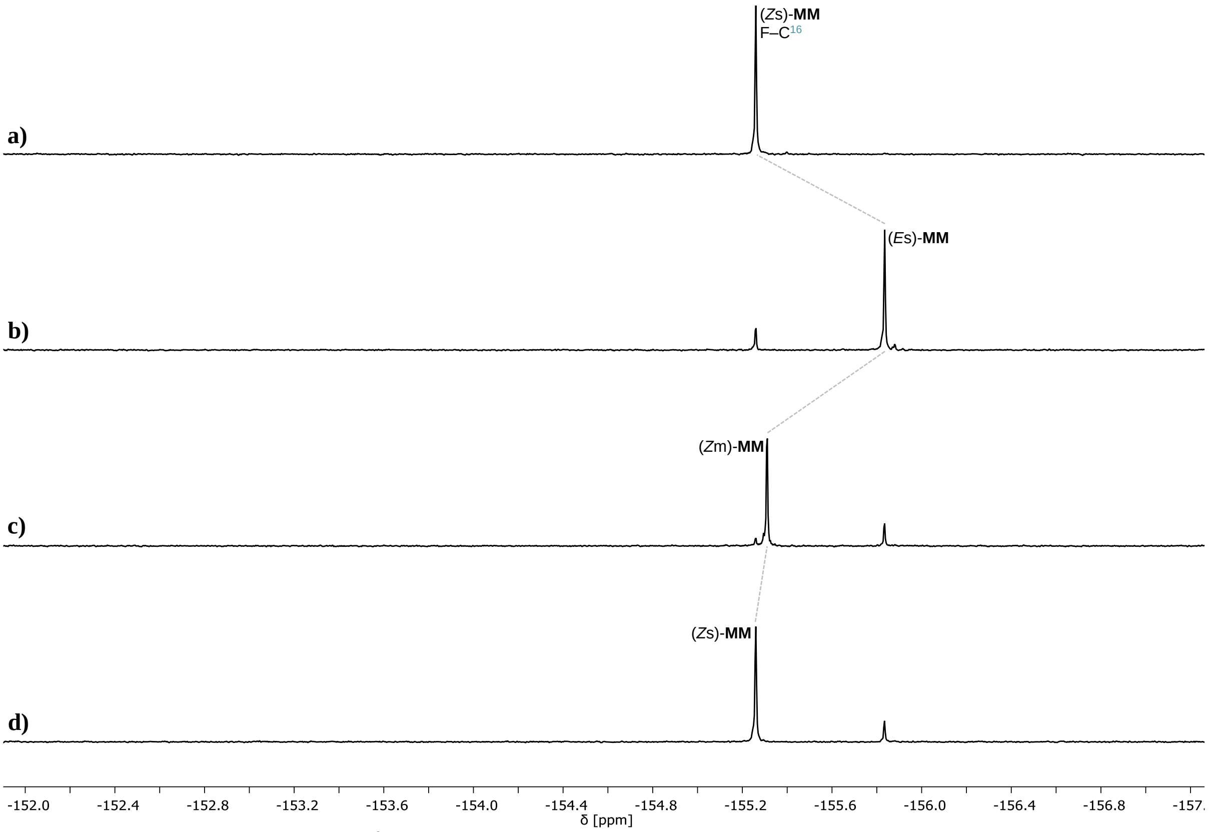

Supporting Figure S26 | Partial ^{19}F{^{1}H} NMR spectra (471 MHz, toluene-d_8, c = 2 mM, 25 °C) of the rotational cycle of control (*Z*s)-**MM**, sequence from top to bottom. Residual (*E*m)-**MM** was found in the spectrum of (*E*s)-**MM** after thermal relaxation at 20 °C. a) Initial (*Z*s)-**MM** spectrum, b) after irradiation with 312 nm at –50 °C and subsequent thermal relaxation at 20 °C, c) after irradiation with 312 nm at 20 °C, d) after thermal relaxation at 80 °C.

4 Switching Behavior

All compounds were measured in toluene. The *E*s isomers were obtained thermally from *E*m/*Z*s PSS mixtures. Samples were irradiated with Thorlabs M310L1 (308 nm, 38.5 mW) mounted LED.

4.1 Switching Behavior of Machine Isomer (*Z*s)-0

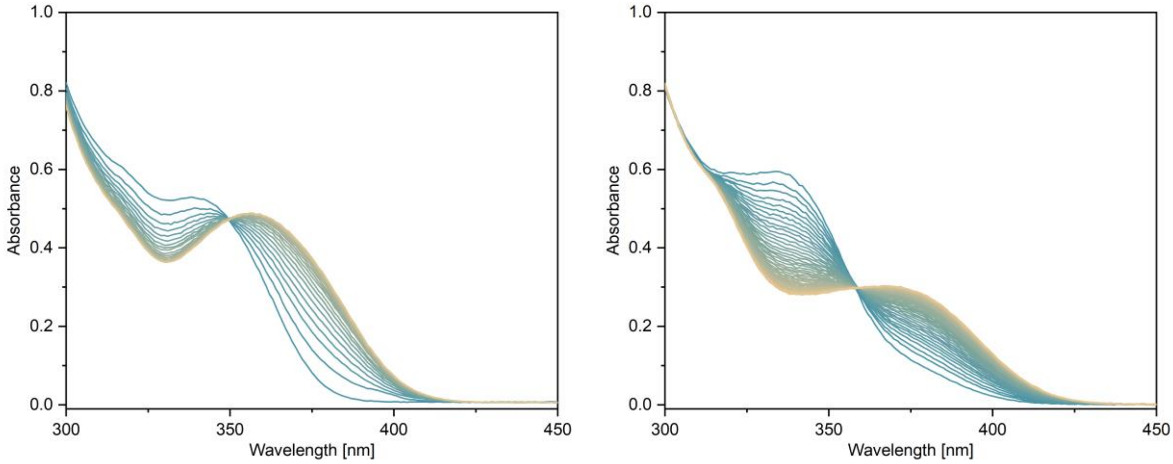

Supporting Figure S27 | Switching behavior of machine (*Z*s)-**0** in toluene, *c* = ~8 μM, from teal to orange. Left: Photo-isomerization of (*Z*s)-**0** to (*E*m)-**1**, –30 °C, isosbestic point at 350 nm. Right: Photo-isomerization of (*E*s)-**1** to (*Z*m)-**2**, 20 °C, isosbestic point at 359 nm.

4.2 Switching Behavior of Bz-(*Z*s)-MA

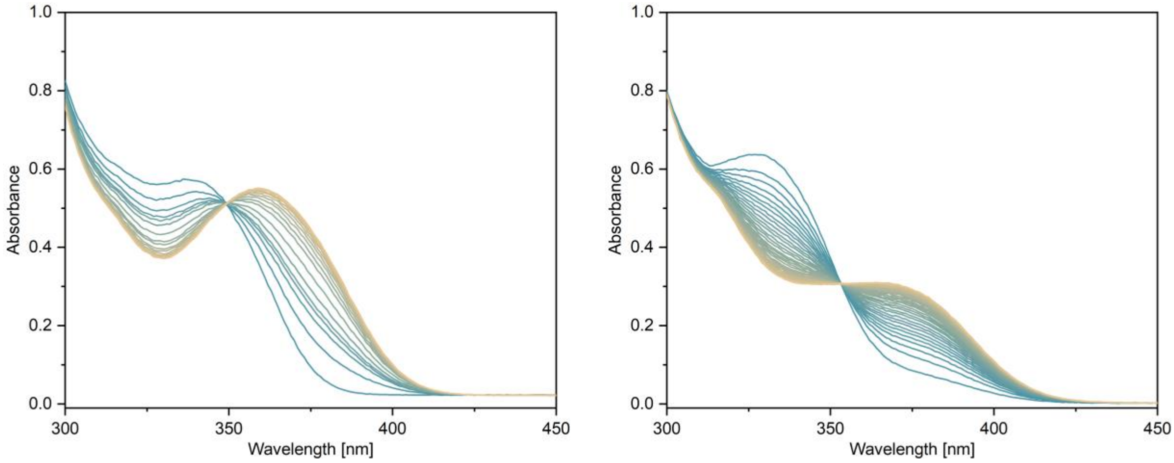

Supporting Figure S28 | Switching behavior of control Bz-(*Z*s)-**MA** in toluene, *c* = ~10 μM, from teal to orange. Left: Photo-isomerization of Bz-(*Z*s)-**MA** to Bz-(*E*m)-**MA**, –30 °C, isosbestic point at 349 nm. Right: Photo-isomerization of Bz-(*E*s)-**MA** to Bz-(*Z*m)-**MA**, 20 °C, isosbestic point at 354 nm.

4.3 Switching Behavior of (*Z*s)-MM

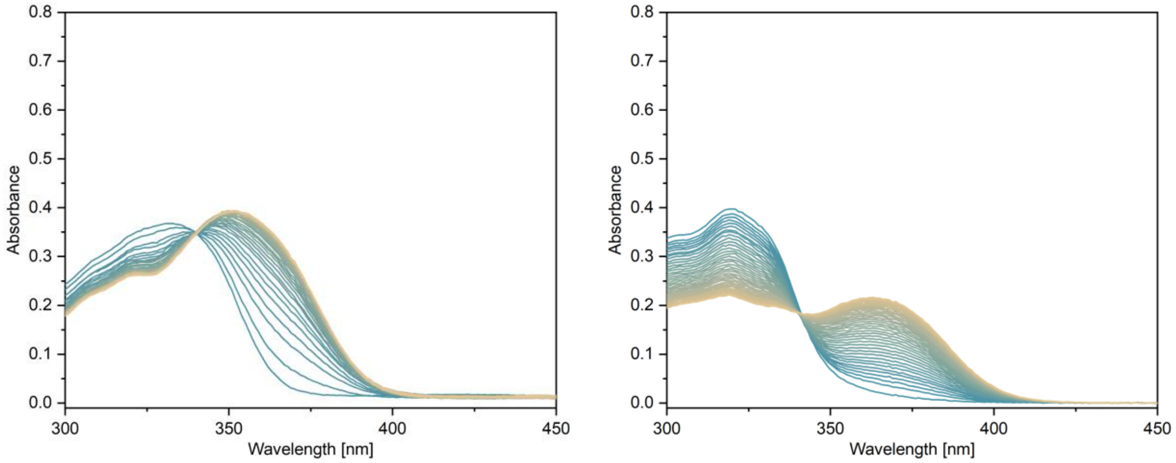

Supporting Figure S29 | Switching behavior of control (*Z*s)-**MM** in toluene, *c* = ~10 μM, from teal to orange. Left: Photo-isomerization of (*Z*s)-**MM** to (*E*m)-**MM**, –30 °C, isosbestic point at 341 nm. Right: Photo-isomerization of (*E*s)-**MM** to (*Z*m)-**MM**, 20 °C, isosbestic point at 343 nm.

4.4 Switching Behavior of rotaxane [2]-(*Z*s)-MR

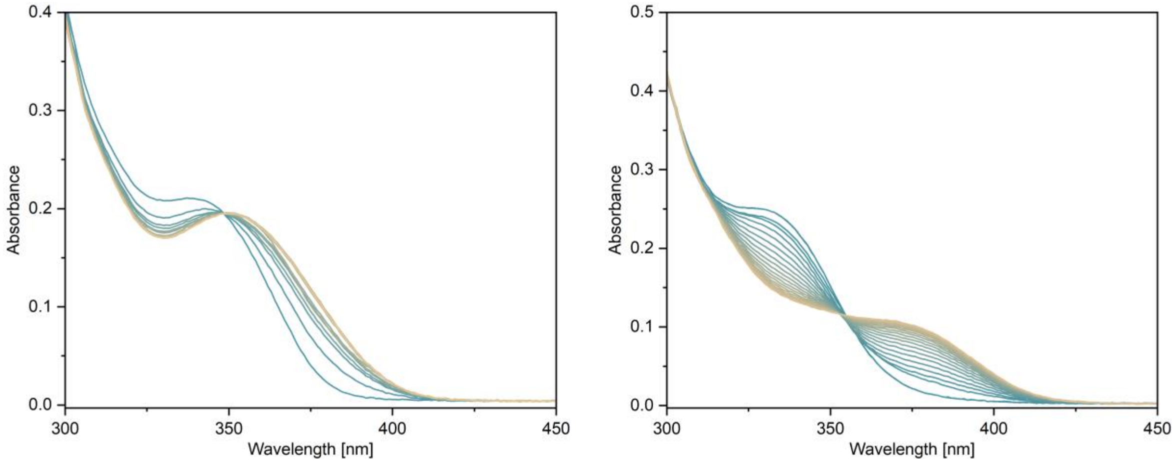

Supporting Figure S30 | Switching behavior of rotaxane [2]-(*Z*s)-**MR** in toluene, *c* = ~5 μM by irradiation with 313 nm, from teal to orange. Left: Photo-isomerization of [2]-(*Z*s)-**MR** to [2]-(*E*m)-**MR**, –23 °C, isosbestic point at 349 nm. Right: Photo-isomerization of [2]-(*E*s)-**MR** to [2]-(*Z*m)-**MR**, 20 °C, isosbestic point at 354 nm.

5 Eyring Analysis

Samples were prepared in toluene (8 – 10 μM) and irradiated to PSS with a Thorlabs M310L1 (308 nm, 38.5 mW) mounted LED. Generally, irradiations to obtain the *E*m isomers were performed at –30 °C, whereas the *Z*m isomers were obtained by irradiation at 20 °C. After irradiation, samples were heated to the desired temperature and the decay of the absorbance (at 375 nm for (*Z*s)-**0**, Bz-(*Z*s)-**MA** and [2]-(*Z*s)-**MR** and 360 nm for (*Z*s)-**MM**) was plotted against time to extract the respective rate constants. In order to determine the activation energy $\Delta G^‡$, a linear least square analysis was performed on the Eyring.

Supporting Table S9 | Activation barriers for the thermal helix inversions calculated for 20 °C. $\Delta G^‡$ and $\Delta H^‡$ are given in kcal/mol, ΔS is given in cal/(K·mol).

Compound	$\Delta G^‡$ (*E*m ⟶ *E*s)<br>[$\Delta H^‡$, $\Delta S^‡$, $t_{1/2}$]	$\Delta G^‡$ (*Z*m ⟶ *Z*s)<br>[$\Delta H^‡$, $\Delta S^‡$, $t_{1/2}$]
(*Z*s)-**0**	19.8 ± 0.1<br>[16.6, –10.7, 61 s]	30.0 ± 0.1<br>[36.6, 22.3, 93 y]
(*Z*s)-**MM**	19.5 ± 0.1<br>[14.6, –16.6, 36 s]	25.8 ± 0.3<br>[26.6, 2.78, 23 d]
Bz-(*Z*s)-**MA**	18.8 ± 0.2<br>[17.5, –4.20, 11 s]	24.7 ± 0.4<br>[21.3, –11.7, 87 h]
[2]-(*Z*s)-**MR**	18.4 ± 0.1<br>[17.3, –3.38, 11 s]	24.6 ± 0.2<br>[21.7, –9.93, 70 h]

First-Order Kinetics Least Linear Square

The thermal helix inversion follows first-order kinetics which can be expressed with the following rate equation:

$$[\mathrm{A}]_t = [\mathrm{A}]_0 \cdot e^{-kt} \qquad (4.1)$$

where [A] is the concentration at time t, $[\mathrm{A}]_0$ is the initial concentration of A and k is the first-order rate constant (time^{-1}).

The [A] is directly proportional with the UV-vis absorbance due to Lambert-Beer's law:

$$A = c \cdot \varepsilon \cdot b \qquad (4.2)$$

where A is the absorbance, c is the concentration in mol L^{-1}, ε is the molar extinction coefficient in mol^{-1} cm^{-1} L and b is the path length in cm.

Linearization of the rate equation 6.1 can be achieved by taking the logarithm on both sides of equation, giving:

$$\ln[\mathrm{A}]_t = \ln[\mathrm{A}]_{\mathrm{Initial}} - kt \tag{4.3}$$

However, due to residual meta stable isomers and peak tailing, a nonzero baseline value A_∞ at infinite time will be obtained while measuring the absorption at the respective wavelength. Correction for this gives the following equation:

$$[\mathrm{A}]_t - [\mathrm{A}]_\infty = ([\mathrm{A}]_{\mathrm{Initial}} - [\mathrm{A}]_\infty) \cdot e^{-kt} \tag{4.4}$$

Which can be linearized by taking the logarithm:

$$\ln([\mathrm{A}]_t - [\mathrm{A}]_\infty) = \ln([\mathrm{A}]_{\mathrm{Initial}} - [\mathrm{A}]_\infty) - kt \tag{4.5}$$

and be simplified into:

$$\ln \frac{[\mathrm{A}]_t - [\mathrm{A}]_\infty}{[\mathrm{A}]_{\mathrm{Initial}} - [\mathrm{A}]_\infty} = -kt \tag{4.6}$$

Where $[\mathrm{A}]_\infty$ can be obtained by plotting the exponential decay curve and the rate constant is obtained from the slope of the linear fit. From this the thermal half-life can be calculated according to:

$$\mathrm{t}_{1/2} = \frac{\ln(2)}{k} \tag{4.7}$$

Finally, the activation Gibbs free energy of activation, activation enthalpy and activation entropy can be determined by plotting the Eyring of *k* as a function of T^{-1}:

$$\ln \frac{k \cdot h}{k_B \cdot T} = -\frac{\Delta H^\ddagger}{RT} + \frac{\Delta S^\ddagger}{R} = -\frac{\Delta G^\ddagger}{RT} \tag{4.8}$$

where *h* is Planck's constant in m^2 kg s^{-1}, k_B is Boltzmann's constant in m^2 kg s^{-2} K^{-1}, T is temperature in K, R is the ideal gas constant in J mol^{-1} K^{-1}, $\Delta H^\ddagger$ is the activation enthalpy in J mol^{-1} and $\Delta S^\ddagger$ is the entropy of activation in J K^{-1} mol^{-1} and $\Delta G^\ddagger$ is Gibbs free energy of activation in J mol^{-1}.

5.1 Thermal Helix Inversion of Machine Isomer (*E*m)-1 to (*E*s)-1

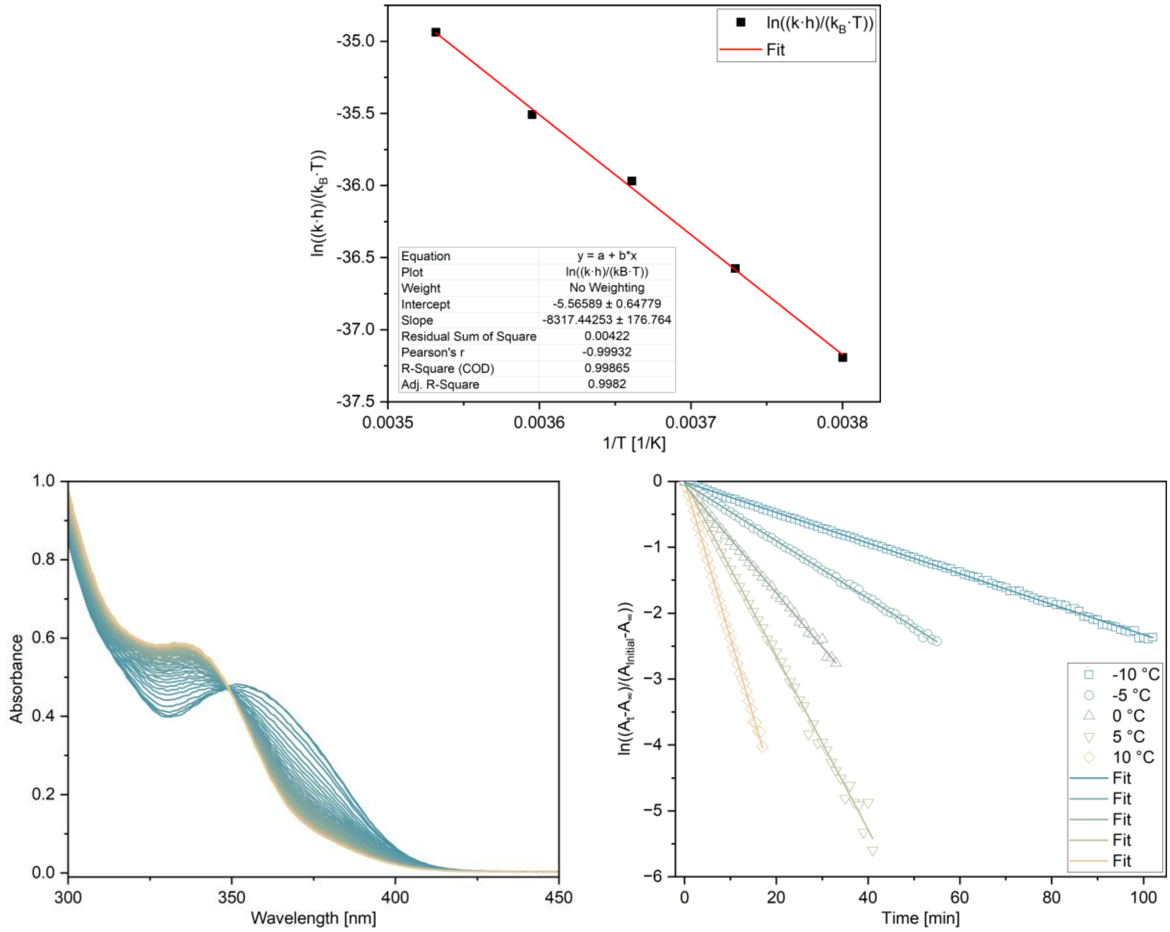

Supporting Figure S31 | Eyring analysis of thermal helix inversion of machine isomer (*E*m)-**1** to (*E*s)-**1**. Rate constants were determined by UV-vis spectroscopy in toluene (~8 μM) at –10, –5, 0, 5 and 10 °C. Top: Eyring plot. Bottom left: Representative example for thermal isomerization followed by UV-vis spectroscopy at –10 °C. From teal to orange, isosbestic point at 349 nm. Bottom right: Linearized decay curves at 375 nm.

5.2 Thermal Helix Inversion of Machine Isomer (*Z*m)-2 to (*Z*s)-2

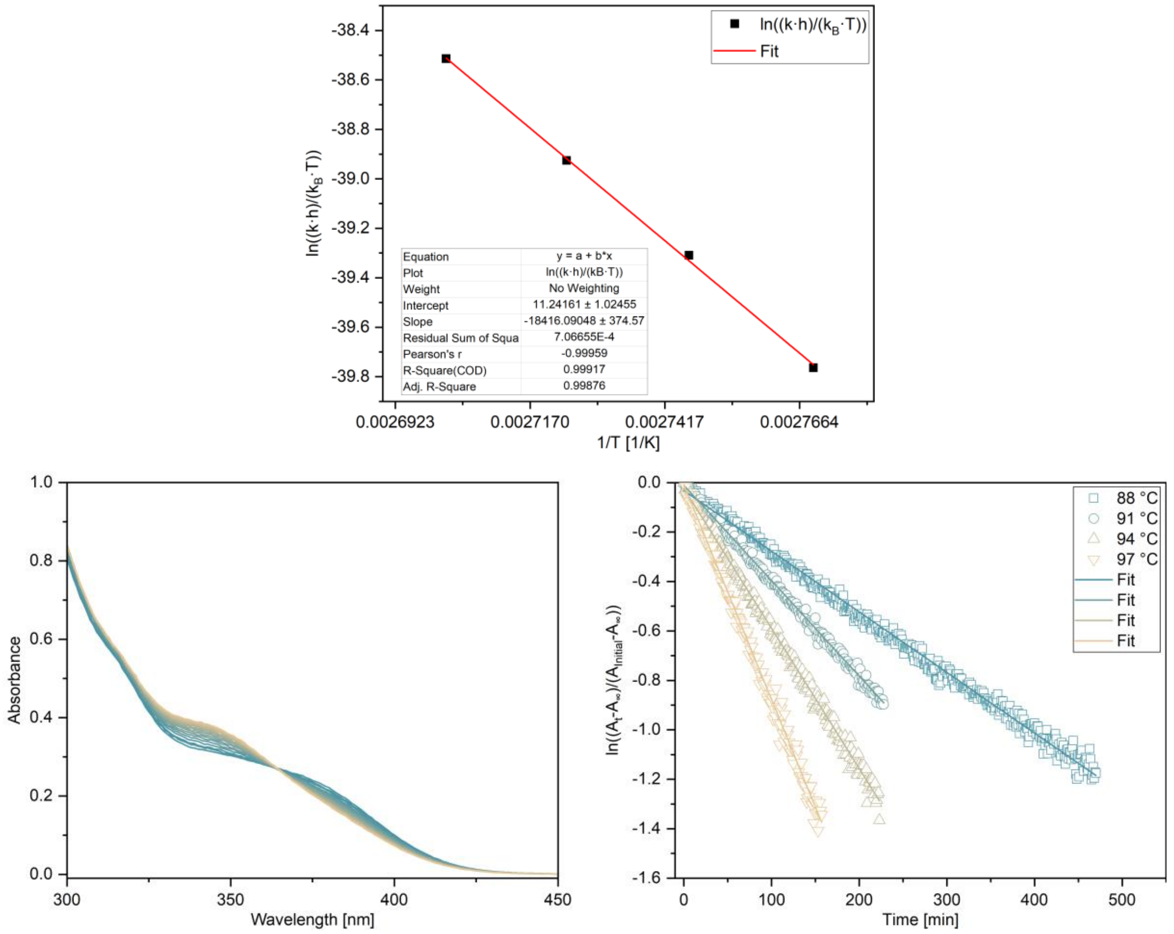

Supporting Figure S32 | Eyring analysis of thermal helix inversion of machine isomer (*Z*m)-**2** to (*Z*s)-**2**. Rate constants were determined by UV-vis spectroscopy in toluene (~8 μM) at 88, 91, 94 and 97 °C. Top: Eyring plot. Bottom left: Representative example for thermal isomerization followed by UV-vis spectroscopy at 97 °C. From teal to orange, isosbestic point at 364 nm. Bottom right: Linearized decay curves at 375 nm.

5.3 Thermal Helix Inversion of Bz-(*E*m)-MA to Bz-(*E*s)-MA

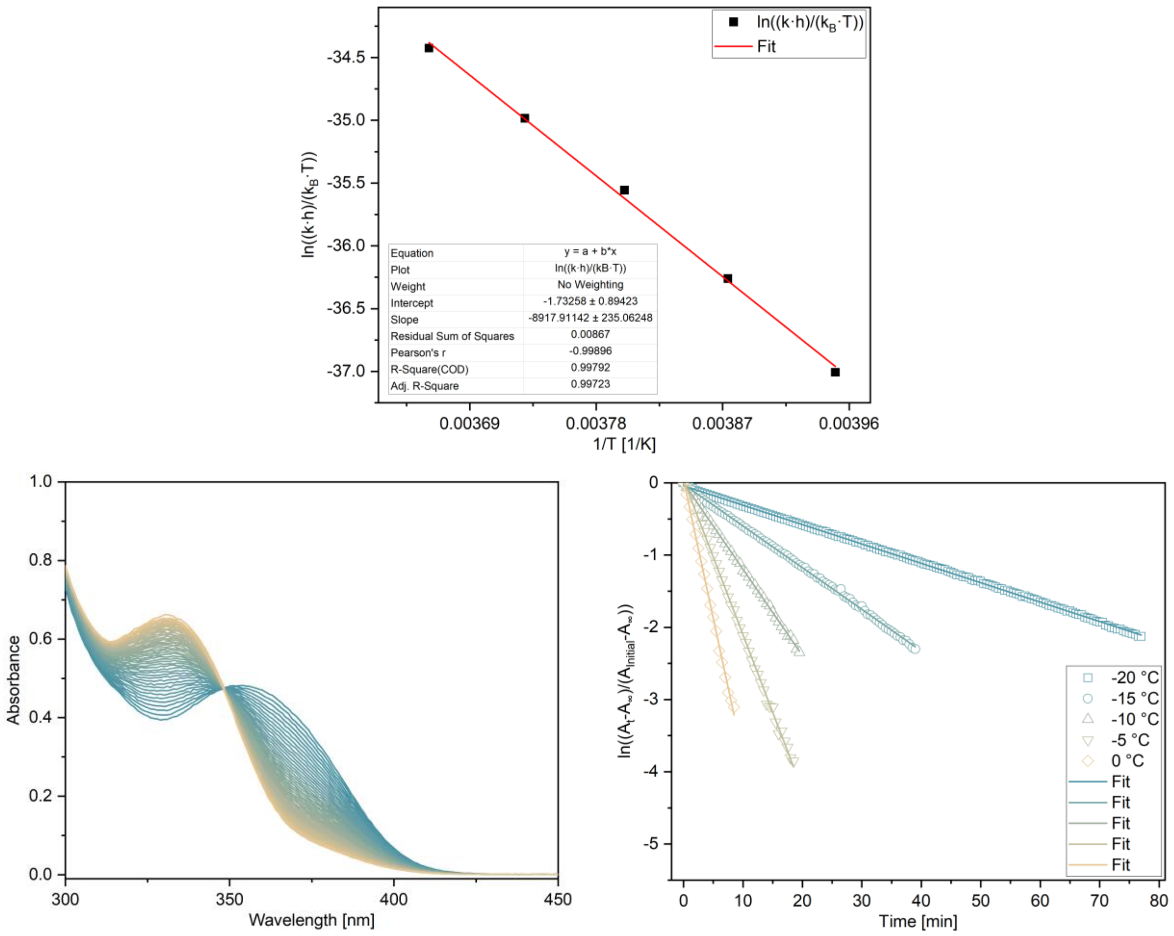

Supporting Figure S33 | Eyring analysis of thermal helix inversion of control Bz-(*E*m)-**MA** to Bz-(*E*s)-**MA**. Rate constants were determined by UV-vis spectroscopy in toluene (~10 μM) at –20, –15, –10, –5 and 0 °C. Top: Eyring plot. Bottom left: Representative example for thermal isomerization followed by UV-vis spectroscopy at –20 °C. From teal to orange, isosbestic point at 348 nm. Bottom right: Linearized decay curves at 375 nm.

5.4 Thermal Helix Inversion of Bz-(*Z*m)-MA to Bz-(*Z*s)-MA

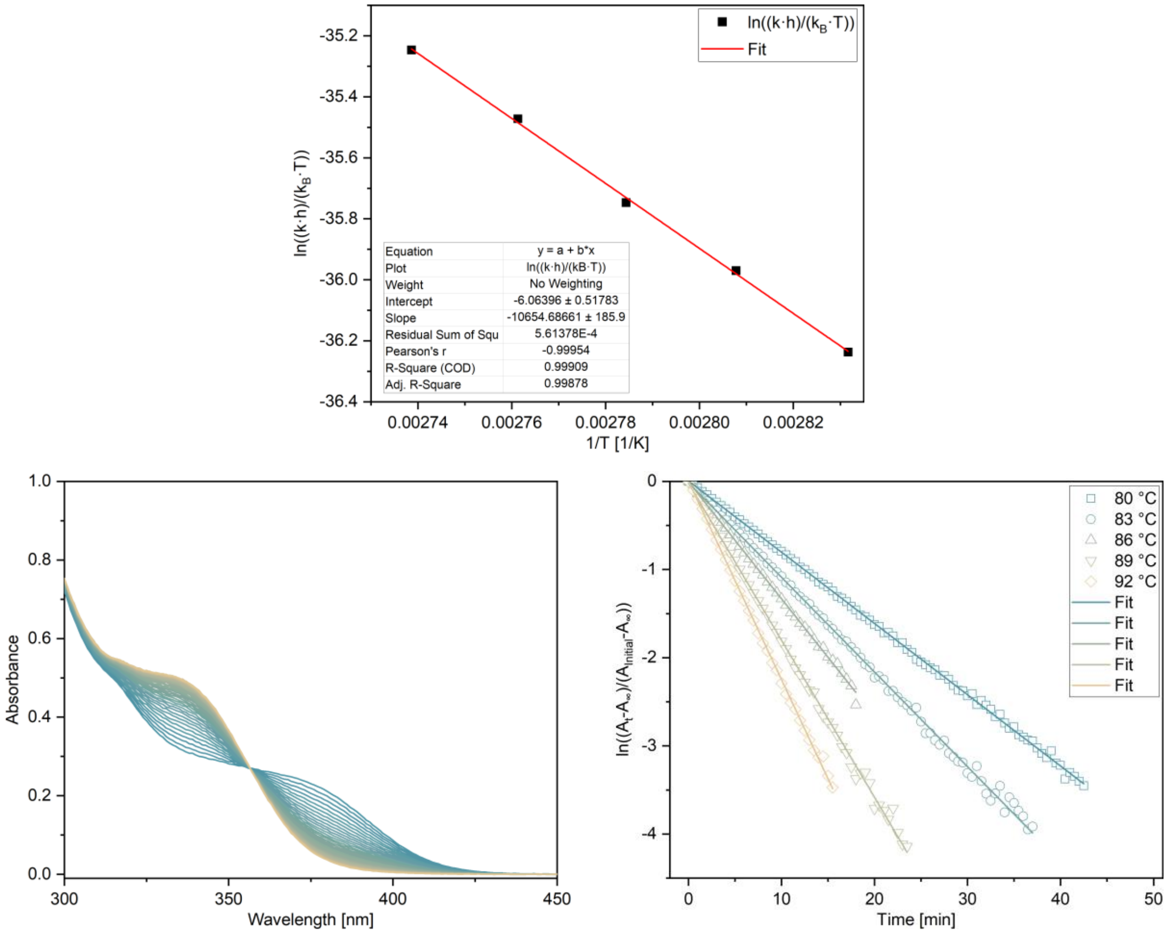

Supporting Figure S34 | Eyring analysis of thermal helix inversion of control Bz-(*Z*m)-**MA** to Bz-(*Z*s)-**MA**. Rate constants were determined by UV-vis spectroscopy in toluene (~10 μM) at 80, 83, 86, 89 and 92 °C. Top: Eyring plot. Bottom left: Representative example for thermal isomerization followed by UV-vis spectroscopy at 80 °C. From teal to orange, isosbestic point at 357 nm. Bottom right: Linearized decay curves at 375 nm.

5.5 Thermal Helix Inversion of Control (*E*m)-MM to (*E*s)-MM

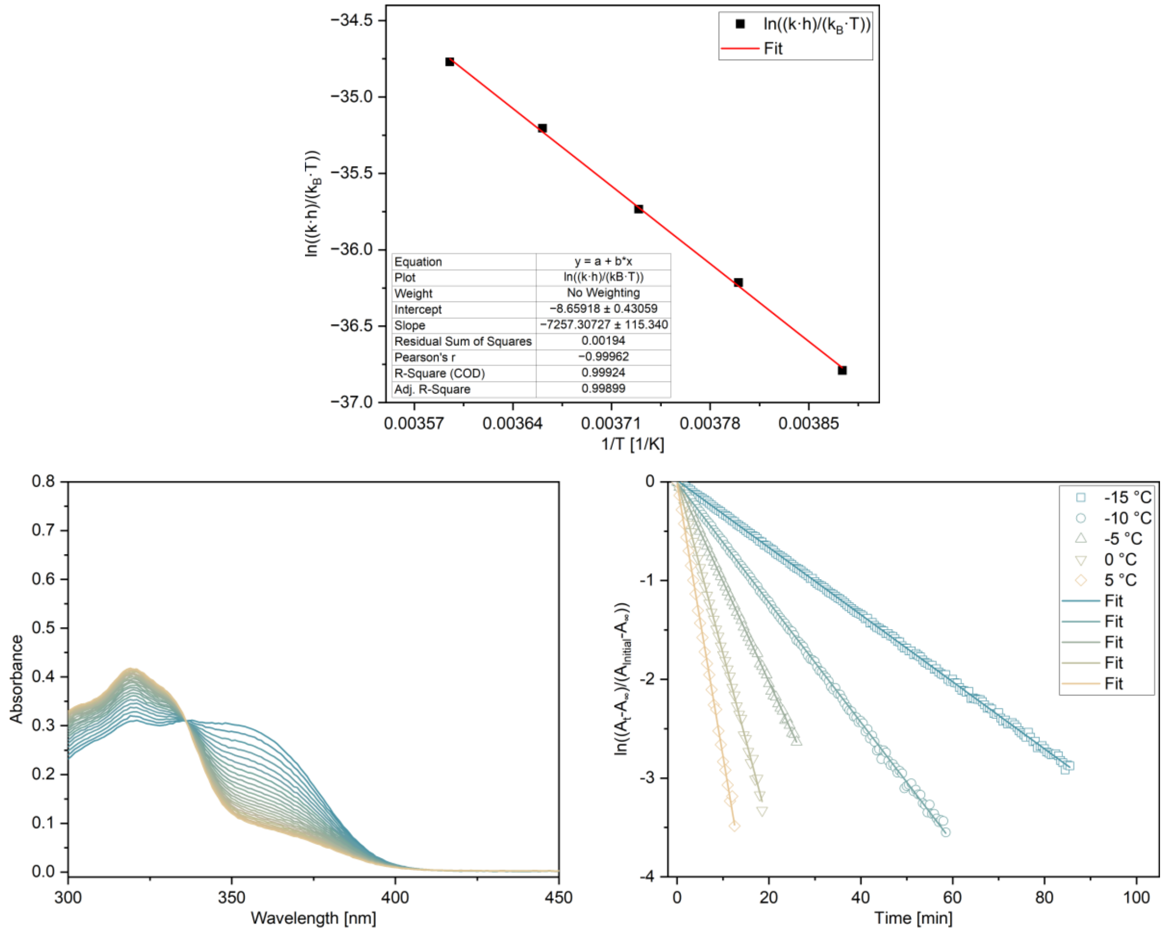

Supporting Figure S35 | Eyring analysis of thermal helix inversion of control (*E*m)-**MM** to (*E*s)-**MM**. Rate constants were determined by UV-vis spectroscopy in toluene (~10 μM) at –15, –10, –5, 0 and 5 °C. Top: Eyring plot. Bottom left: Representative example for thermal isomerization followed by UV-vis spectroscopy at –10 °C. From teal to orange, isosbestic point at 336 nm. Bottom right: Linearized decay curves at 360 nm.

5.6 Thermal Helix Inversion of Control (*Z*m)-MM to (*Z*s)-MM

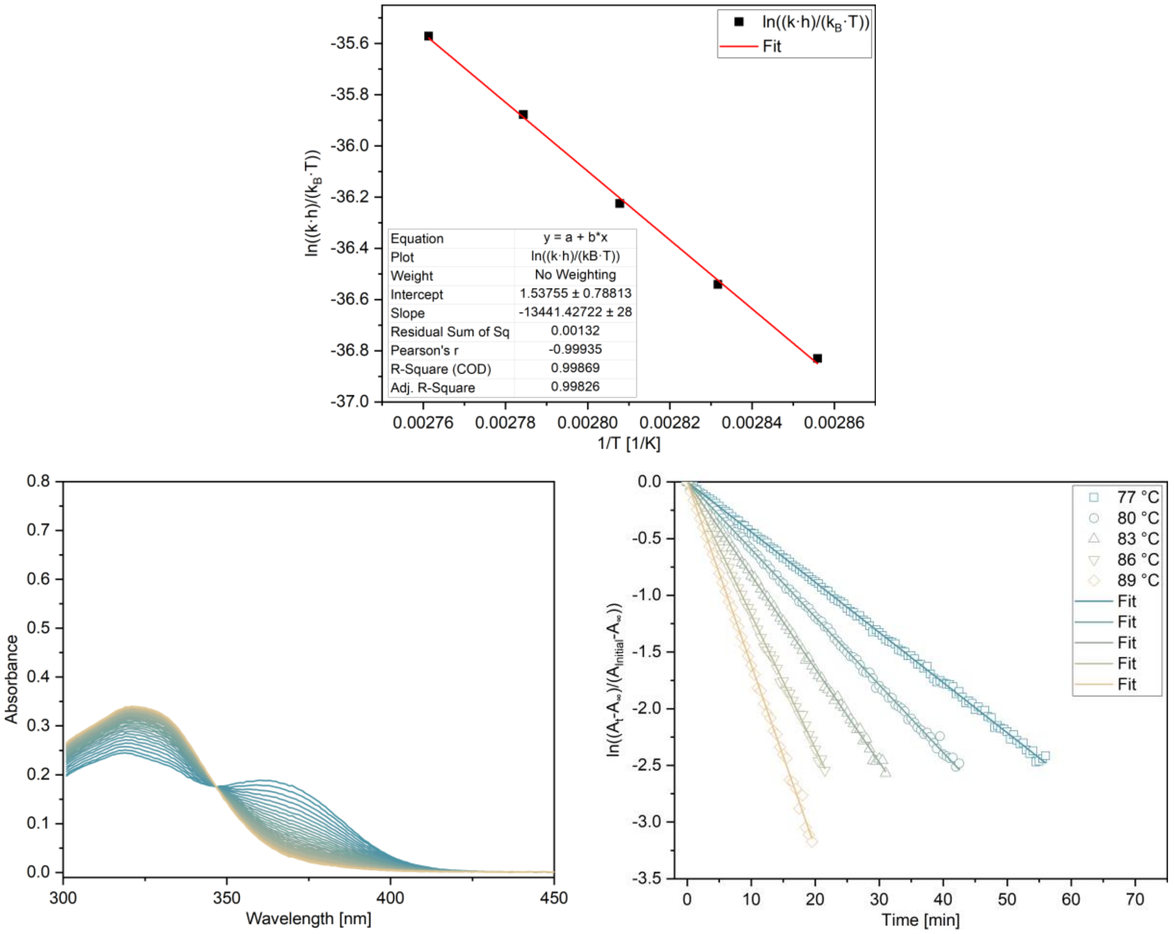

Supporting Figure S36 | Eyring analysis of thermal helix inversion of control (*Z*m)-**MM** to (*Z*s)-**MM**. Rate constants were determined by UV-vis spectroscopy in toluene (~10 μM) at 77, 80, 83, 86 and 89 °C. Top: Eyring plot. Bottom left: Representative example for thermal isomerization followed by UV-vis spectroscopy at 80 °C. From teal to orange, isosbestic point at 347 nm. Bottom right: Linearized decay curves at 360 nm.

5.7 Thermal Helix Inversion of Rotaxane [2]-(*E*m)-MR to [2]-(*E*s)-MR

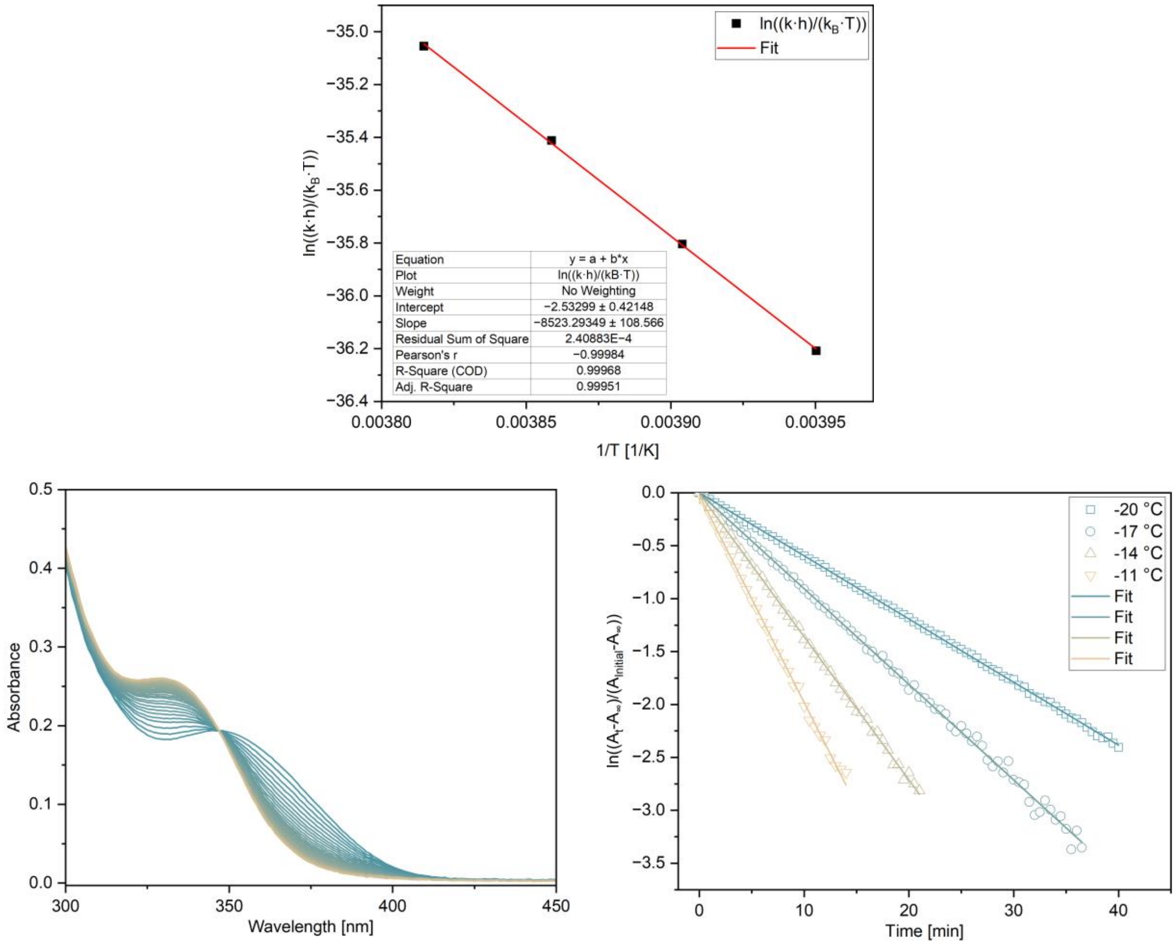

Supporting Figure S37 | Eyring analysis of thermal helix inversion of rotaxane [2]-(*E*m)-**MR** to [2]-(*E*s)-**MR**. Rate constants were determined by UV-vis spectroscopy in toluene (~5 μM) at –20, –17, –14 and –11 °C. Top: Eyring plot. Bottom left: Representative example for thermal isomerization followed by UV-vis spectroscopy at –20 °C. From teal to orange, isosbestic point at 348 nm. Bottom right: Linearized decay curves at 375 nm.

5.8 Thermal Helix Inversion of Rotaxane [2]-(*Z*m)-MR to [2]-(*Z*s)-MR

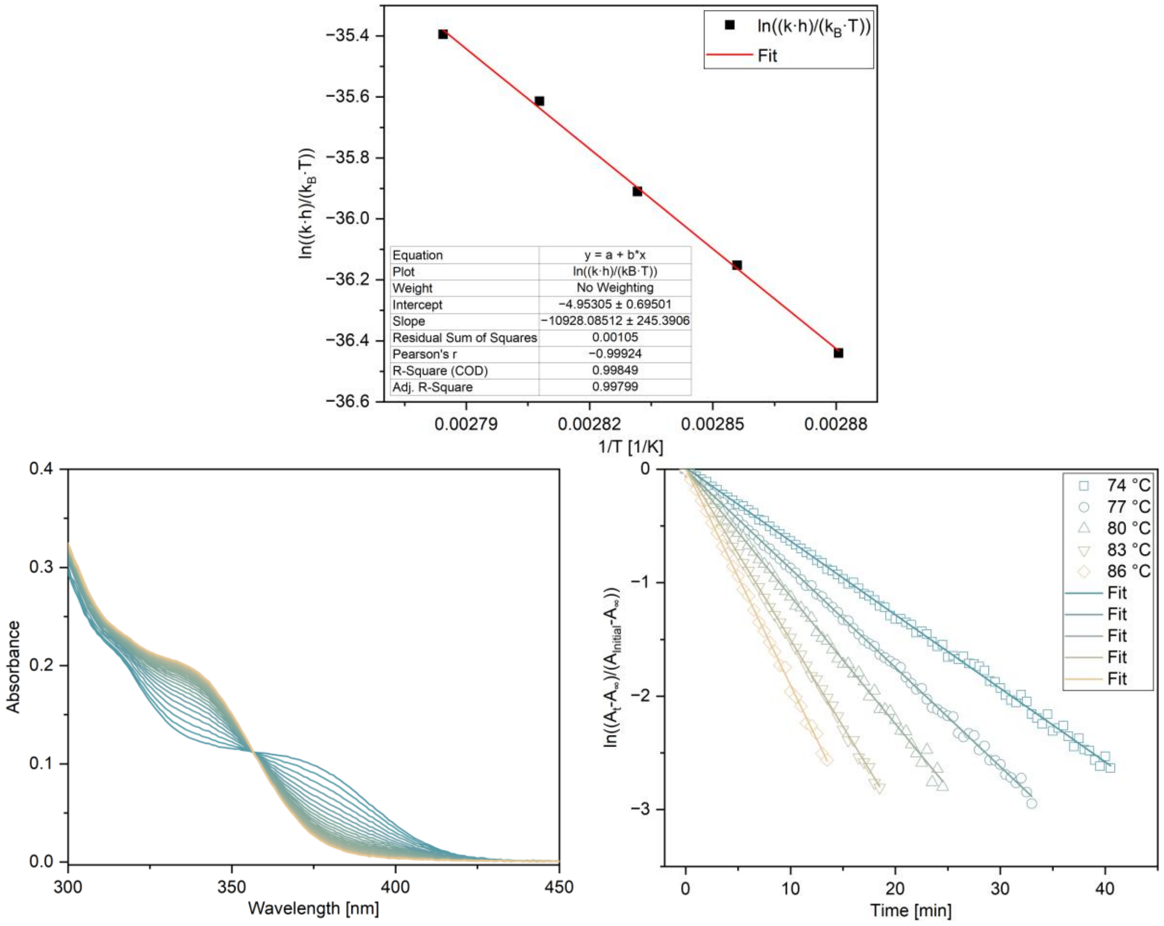

Supporting Figure S38 | Eyring analysis of thermal helix inversion of rotaxane [2]-(*Z*m)-**MR** to [2]-(*Z*s)-**MR**. Rate constants were determined by UV-vis spectroscopy in toluene (~5 μM) at 74, 77, 80, 83 and 86 °C. Top: Eyring plot. Bottom left: Representative example for thermal isomerization followed by UV-vis spectroscopy at 80 °C. From teal to orange, isosbestic point at 357 nm. Bottom right: Linearized decay curves at 375 nm.

6 Covalent Capture and Release Experiments

General Procedure Grubbs Alkene Metathesis (Covalent Capture)

To a J. Young tube was added a stock solution of (*Z*s)-**0** (0.5 mL, 0.37 mM, 0.19 μmol, in toluene-d_8) with 4-fluoroanisole (~0.35 mM in toluene-d_8). Each NMR tube was measured before irradiation. Tubes were then used immediately for (*Z*s)-**0** or irradiated with 312 nm for 2 h at either –50 °C ((*E*m)-**1**/(*E*s)-**1**) or 20 °C ((*Z*m)-**2**). These samples were then analyzed by ^{1}H and ^{19}F NMR spectroscopy and UPLC-HRMS (BEH phenyl cyclohexyl column, eluent: 98% MeCN/IPA 9:1, 2% water, 0.1% FA, 40 °C). Grubbs catalyst M110 (5 μL, 5 mM, 14 mol% in toluene-d_8) was added and the reaction was heated at 50 °C with an open vessel in the glovebox for 2 d. The samples were again analyzed by ^{1}H and ^{19}F NMR and UPLC-HRMS (BEH phenyl cyclohexyl column, eluent: 98% MeCN/IPA 9:1, 2% H_2O, 0.1% FA 40 °C) before subsequent reductive release.

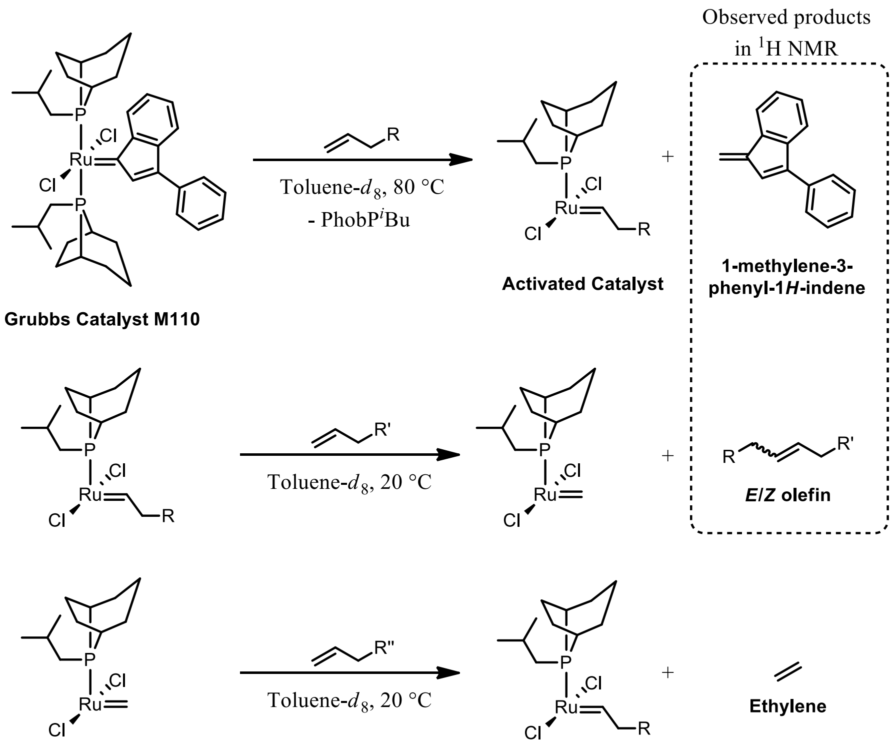

Supporting Figure S39 | Proposed Grubbs olefin metathesis reaction sequence. Top: first initiation of the pre-catalyst with a terminal alkene forms the activated catalyst and 1-methylene-3-phenyl-1*H*-indene. Middle: reaction of the activated catalyst with another terminal alkene initiates the metathesis forming the *E*/*Z* olefin and a new metal-carbene. Bottom: finally, the catalyst undergoes reaction with another terminal alkene reforming the activated catalyst and ethylene gas.

General Procedure DIBAL-H Reduction (Reductive Release)

The respective sample was transferred to a glass vial and subsequently reacted with DIBAL-H (1.2 M in toluene, 3 drops) at 0 °C, shaken and left to react for 5 min at 0 °C. The sample was subsequently quenched with saturated Rochelle's salt solution and extracted with EtOAc. The organic phase was analyzed by UPLC-HRMS (BEH phenyl cyclohexyl column, eluent: 98% MeCN/IPA 9:1, 2% water, 0.1% FA, 40 °C). Peaks were assigned by their UV-vis spectrum and high-resolution mass signals.

6.1 Covalent Capture and Release from Machine Isomer (*Z*s)-0

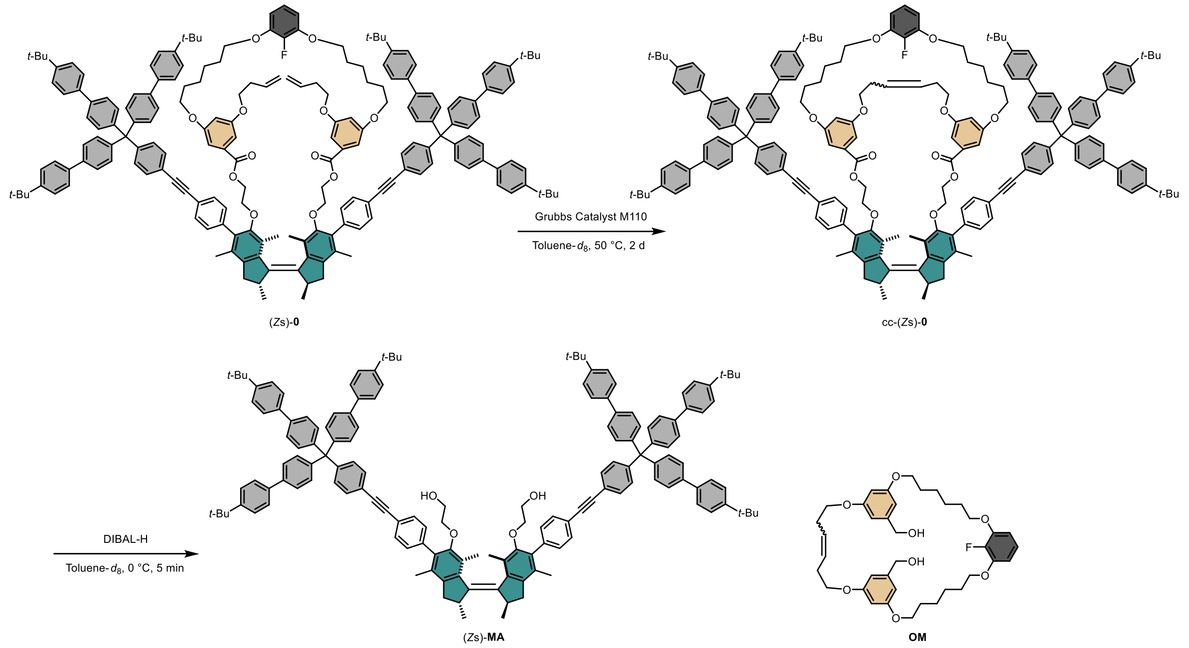

Supporting Figure S40 | Reaction scheme for the covalent capture and release from machine isomer (*Z*s)-**0**.

Machine isomer (*Z*s)-**0** reacts with Grubbs M110 at 50 °C in toluene-d_8 for 2 d forming covalently captured cc-(*Z*s)-**0** in 80 ± 2% NMR conversion along with the formation of dimer in 13 ± 5% NMR conversion. Additionally, UPLC-HRMS indicated the loss of ethylene. Workup was then performed according to General Procedure DIBAL-H Reduction. UPLC-HRMS analysis was performed of the crude reaction mixture and showed to be mainly consisting of (*Z*s)-**MA** with minor amounts of reduced alkyne (*Z*s)-**MA** (<5%). Formation of macrocycle **OM** and dimer **OM** was confirmed by mass spectrometry. **OM** calculated for $C_{38}H_{48}FO_8Na^+$ ($[M+Na]^+$) 675.3304, found 675.3284. Mass error: 3.0 ppm. dimer **OM** calculated for $C_{76}H_{98}F_2NaO_{16}^+$ ($[M+Na]^+$) 1327.6715, found 1327.6670. Mass error: 3.4 ppm.

^{1}H NMR Before and After Covalent Capture by Grubbs Metathesis

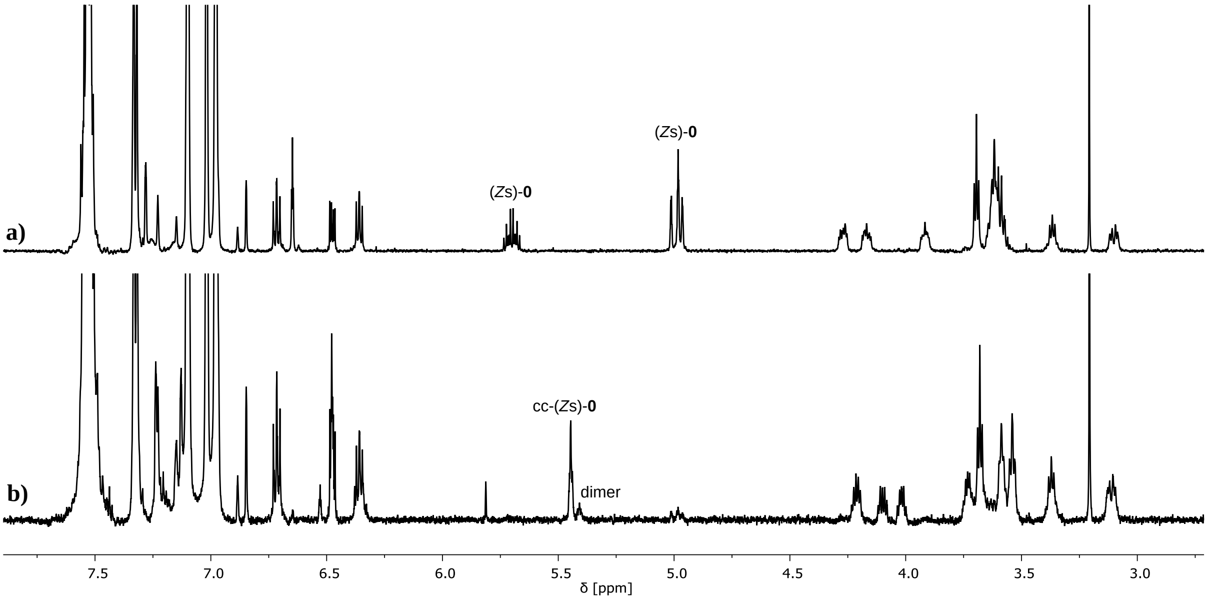

Supporting Figure S41 | Partial ^{1}H NMR spectra (600 MHz, toluene-d_8, c = 0.37 mM, 25 °C) of the covalent capture experiments of machine isomer (*Z*s)-**0**. a) Initial machine isomer (*Z*s)-**0** spectrum, b) after Grubbs catalyst M110 at 50 °C for 2 d.

^{19}F{^{1}H} NMR Before and After Covalent Capture by Grubbs Metathesis

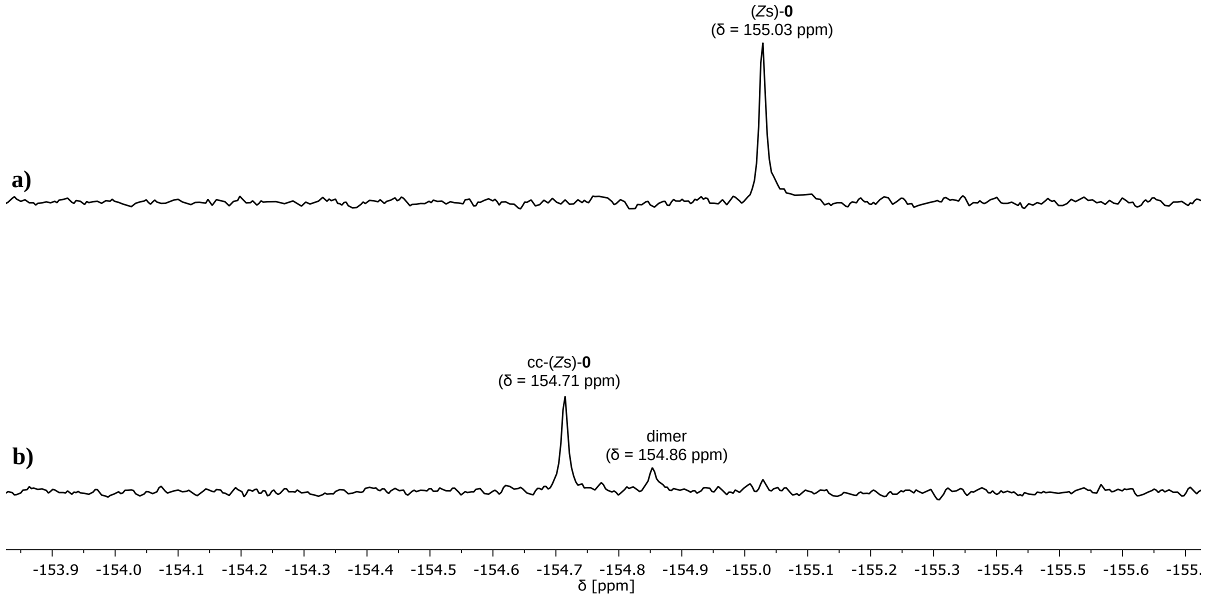

Supporting Figure S42 | Partial ^{19}F{^{1}H} NMR spectra (471 MHz, toluene-d_8, c = 0.37 mM, 25 °C) of the covalent capture experiments of machine isomer (*Z*s)-**0**. a) Initial machine isomer (*Z*s)-**0** spectrum, b) after Grubbs catalyst M110 at 50 °C for 2 d. Spectra referenced to 4-fluoranisole.

UPLC-HRMS Analysis of Covalent Capture and Release of (*Z*s)-0

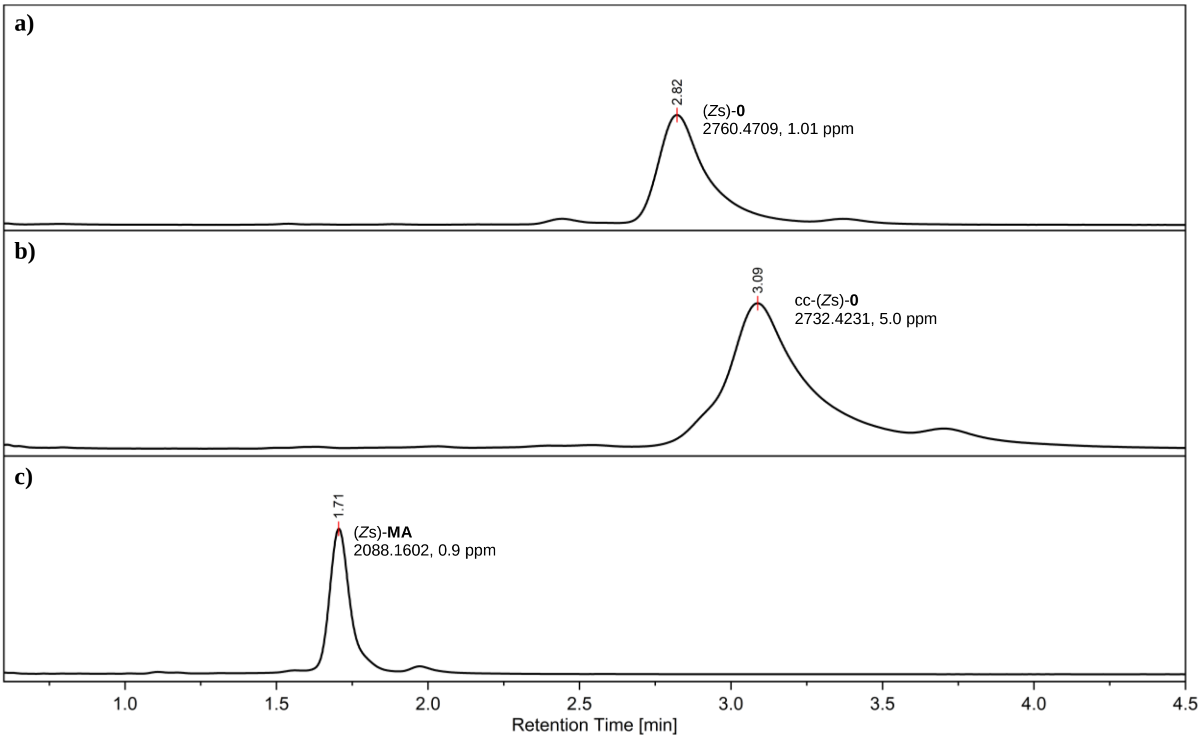

Supporting Figure S43 | UPLC-HRMS analysis of the covalent capture experiment of (*Z*s)-**0** (BEH phenyl cyclohexyl column, eluent: 98% MeCN/IPA 9:1, 2% H_2O, 0.1% FA, 40 °C), *m/z* is given as $[M+Na]^+$. a) Initial machine isomer (*Z*s)-**0** chromatogram, b) after Grubbs catalyst M110 at 50 °C for 2 d, c) after DIBAL-H reduction at 0 °C for 5 min. Peaks were assigned by UV-vis spectroscopy and HRMS. Macrocycles were found in the injection peak due to the solvent mixture that is required to elute the highly lipophilic compounds.

6.2 Covalent Capture and Release from Machine Isomers (*E*m)-1 and (*E*s)-1

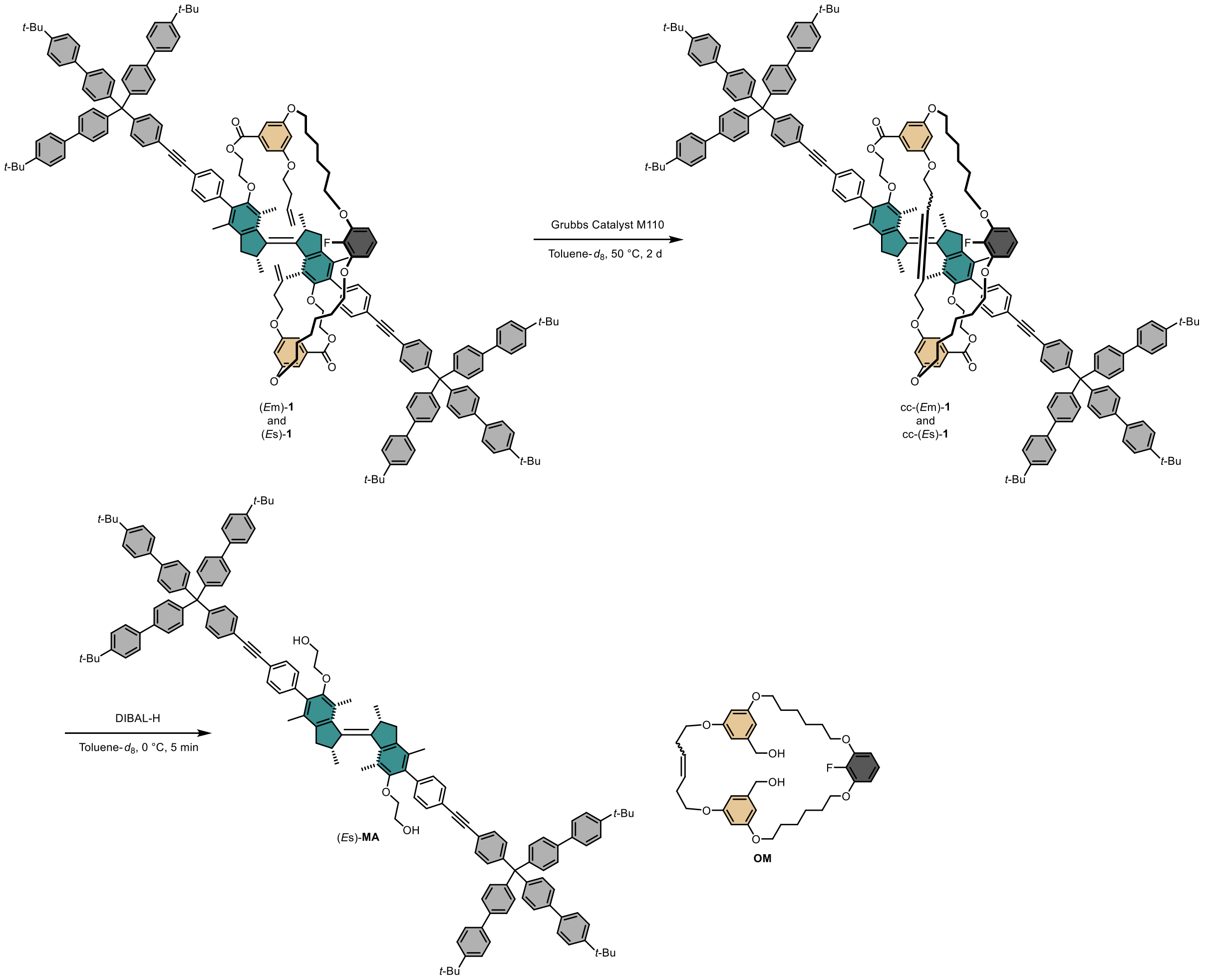

Supporting Figure S44 | Reaction scheme for the covalent capture and release from machine isomers (*E*m)-**1** and (*E*s)-**1**.

Machine isomer mixture of (*E*m)-**1** and (*E*s)-**1** reacted with Grubbs M110 at 50 °C in toluene-d_8 for 2 d forming covalently captured cc-(*E*m)-**1**/(*E*s)-**1**) in 65 ± 2% NMR conversion. Workup was then performed according to General Procedure DIBAL-H Reduction. UPLC-HRMS analysis was performed of the crude reaction mixture and showed to be mainly consisting of (*E*s)-**MA** with minor amounts of reduced alkyne (*E*s)-**MA** (<5%) and leftover (*Z*s)-**MA** originating from the PSS mixture. Formation of macrocycle **OM** was confirmed by mass spectrometry. **OM** calculated for $C_{38}H_{48}FO_8Na^+$ ($[M+Na]^+$) 675.3304, found 675.3286. Mass error: 2.7 ppm.

^{1}H NMR Before and After Covalent Capture by Grubbs Metathesis

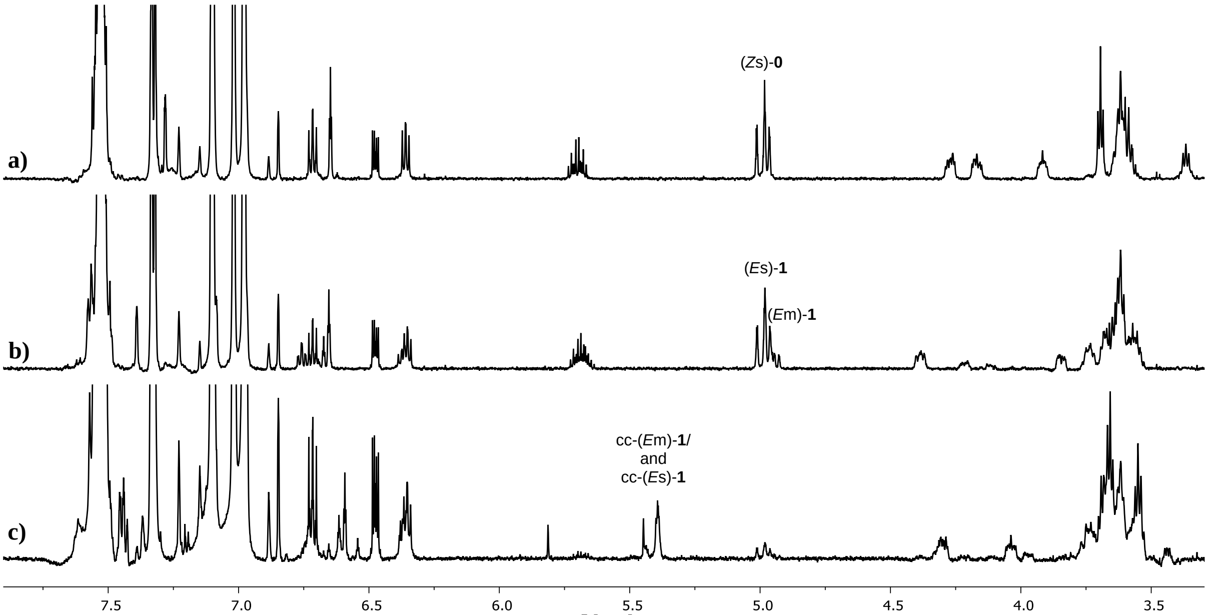

Supporting Figure S45 | Partial ^{1}H NMR spectra (600 MHz, toluene-d_8, c = 0.37 mM, 25 °C) of the covalent capture experiments of machine isomer mixture (*E*m)-**1**/(*E*s)-**1**. a) Initial machine isomer (*Z*s)-**0** spectrum, b) after irradiation with 312 nm at –50 °C for 2 h, c) after Grubbs catalyst M110 at 50 °C for 2 d.

^{19}F{^{1}H} NMR Before and After Covalent Capture by Grubbs Metathesis

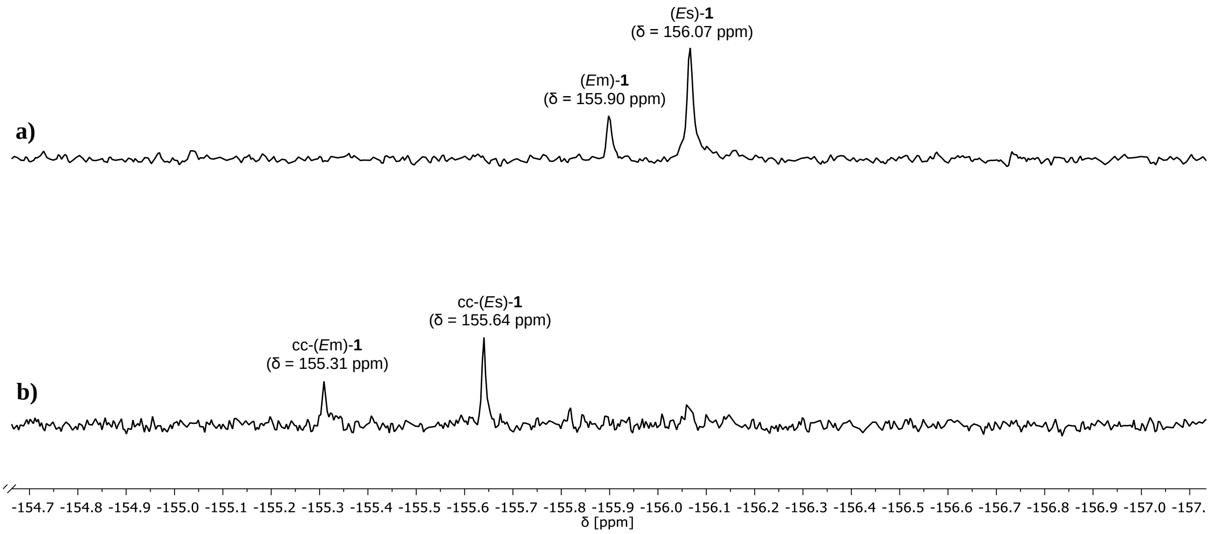

Supporting Figure S46 | Partial ^{19}F{^{1}H} NMR spectra (471 MHz, toluene-d_8, c = 0.37 mM, 25 °C) of the covalent capture experiments of machine isomer mixture (*E*m)-**1**/(*E*s)-**1**. a) After irradiation of (*Z*s)-**0** with 312 nm at –50 °C for 2 h, b) after Grubbs catalyst M110 at 50 °C for 2 d. Spectra referenced to 4-fluoranisole.

UPLC-HRMS Analysis of Covalent Capture and Release of Machine (*E*m)-1/(*E*s)-1

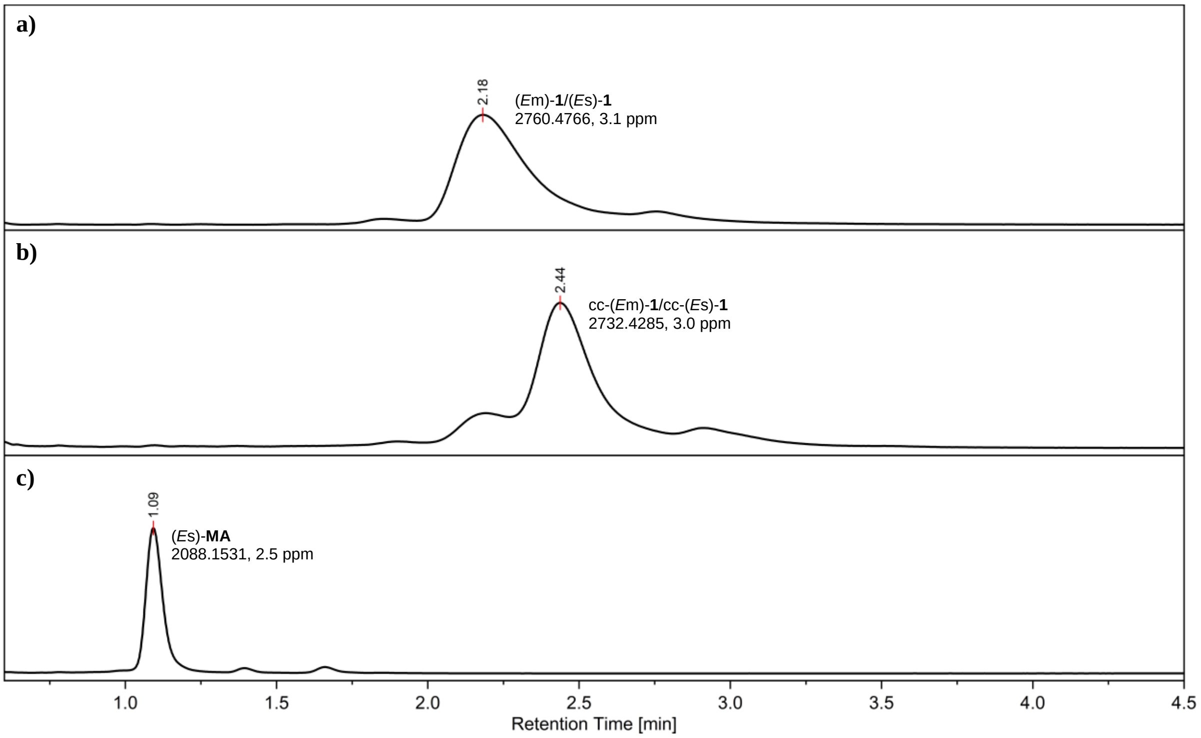

Supporting Figure S47 | UPLC-HRMS analysis of the covalent capture experiment of machine isomers (*E*m)-**1**/(*E*s)-**1** (BEH phenyl cyclohexyl column, eluent: 98% MeCN/IPA 9:1, 2% H_2O, 0.1% FA, 40 °C), *m/z* is given as $[M+Na]^+$. a) Chromatogram of machine isomers (*E*m)-**1**/(*E*s)-**1**, b) after Grubbs catalyst M110 at 50 °C for 2 d, c) after DIBAL-H reduction at 0 °C for 5 min. Peaks were assigned by UV-vis spectroscopy and HRMS. Macrocycle **OM** was found in the injection peak due to the solvent mixture that is required to elute the highly lipophilic compounds.

6.3 Covalent Capture and Release from Machine Isomer (*Z*m)-2

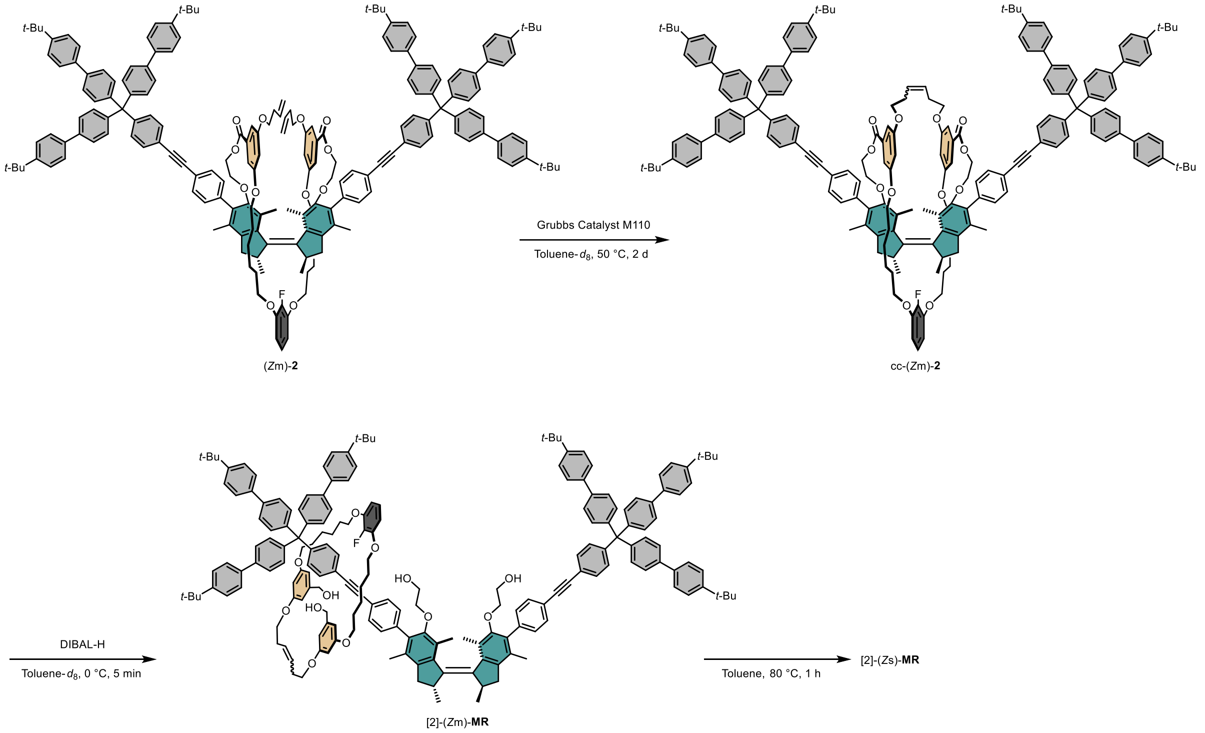

Supporting Figure S48 | Reaction scheme for the covalent capture and release from machine isomer (*Z*m)-**2**.

Machine isomer (*Z*m)-**2** was reacted with Grubbs M110 at 50 °C in toluene-d_8 for 2 d forming covalently captured cc-(*Z*m)-**2** in 83 ± 2% NMR conversion relative to the total available amount of machine isomer (*Z*m)-**2**. Two new signals formed in ^{19}F NMR which is assumed to be the *E* an *Z* olefin macrocycle of cc-(*Z*m)-**2** that start to resolve due to the limited freedom of rotation. Workup was then performed according to General Procedure DIBAL-H Reduction. UPLC-HRMS analysis was performed of the crude reaction mixture and showed formation of rotaxane [2]-(*Z*m)-**MR**.

^{1}H NMR Before and After Covalent Capture by Grubbs Metathesis

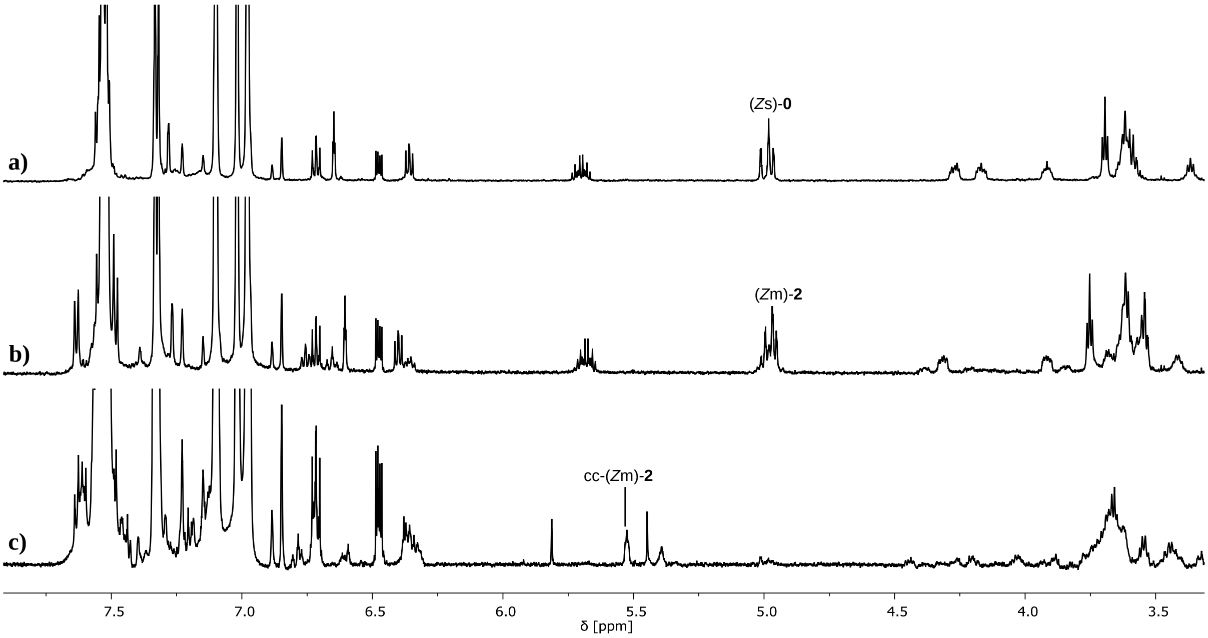

Supporting Figure S49 | Partial ^{1}H NMR spectra (600 MHz, toluene-d_8, c = 0.37 mM, 25 °C) of the covalent capture experiment of machine (*Z*m)-**2**. a) Initial machine isomer (*Z*s)-**0** spectrum, b) after irradiation with 312 nm at 20 °C for 2 h, c) after Grubbs catalyst M110 at 50 °C for 2 d. Note: remaining (*E*m)-**1**/(*E*s)-**1** from the PSS mixture will also be clipped resulting in the formation of the peak at 5.39 ppm.

^{19}F{^{1}H} NMR Before and After Covalent Capture by Grubbs Metathesis

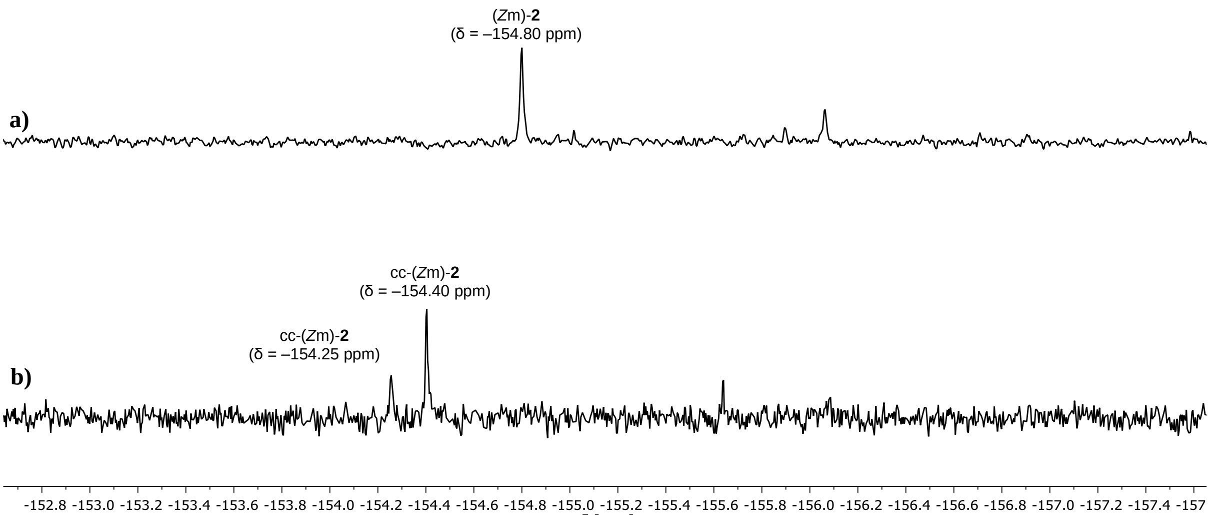

Supporting Figure S50 | Partial ^{19}F{^{1}H} NMR spectra (471 MHz, toluene-d_8, c = 0.37 mM, 25 °C) of the covalent capture experiment of machine (*Z*m)-**2**. a) After irradiation of (*Z*s)-**0** with 312 nm at –50 °C for 2 h, b) after Grubbs catalyst M110 at 50 °C for 2 d. Spectra referenced to 4-fluoranisole.

UPLC-HRMS Analysis of Covalent Capture and Release of (*Z*m)-2

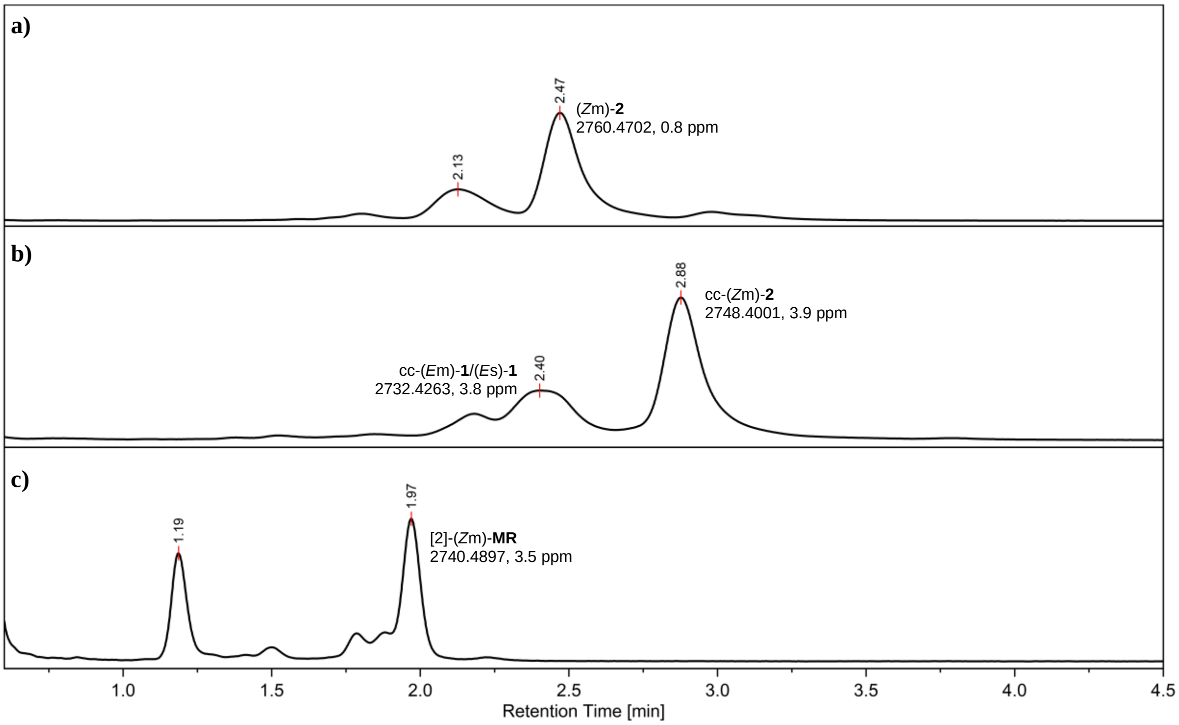

Supporting Figure S51 | UPLC-HRMS analysis of the covalent capture of machine isomer (*Z*m)-**2** (BEH phenyl cyclohexyl column, eluent: 98% MeCN/IPA 9:1, 2% H_2O, 0.1% FA, 40 °C), *m/z* is given as $[M+K]^+$ for covalently captured cc-(*Z*m)-**2**, all other *m/z*'s are given as $[M+Na]^+$. a) Chromatogram of machine isomer (*Z*m)-**2**, b) after Grubbs catalyst M110 at 50 °C for 2 d. c) after DIBAL-H reduction at 0 °C for 5 min. Peaks were assigned by UV-vis spectroscopy and HRMS.

7 Characterization of Rotaxane [2]-(*Z*s)-MR

7.1 NMR Spectroscopy Comparisons

Supporting Table S10 | Comparison of chemical shifts (ppm) in ^{1}H NMR (500 or 600 MHz, $CDCl_3$, 25 °C) of characteristic signals of rotaxane [2]-(*Z*s)-**MR** with its individual components. Center of multiplets are taken as reference point. Both *E* and *Z* isomers of the olefin macrocycle were compared.

Proton	(*Z*s)-**MA**	[2]-(*Z*s)-**MR**	**OM**	*Δ* ppm
**H–C^{2}**	3.45	3.40		0.05
**H–C^{3}**	3.14, 2.48	3.13, 2.46		0.01, 0.02
**CH_3–C^{5}**	2.03	2.00		0.03
**H–C^{29}**	3.66, 3.45	3.55, 3.35		0.11, 0.10
**H–C^{30}**		3.34	3.45	0.11
**H–C^{33}**		6.47	6.57	0.10
**H–C^{34}**		6.76	6.91	0.15
**H–C^{35}**		3.92	4.04	0.12
**H–C$^{42/45}$**		6.40	6.48	0.08
**H–C^{44}**		4.47	4.58, 4.56	0.11, 0.09
**H–C^{47}**		6.24	6.35	0.11
**H–C^{48}**		3.96	3.81	0.15
**H–C^{49}**		2.42, 2.34	2.56, 2.48	0.14, 0.14
**H–C^{50}**		5.54, 5.46	5.64, 5.63	0.10, 0.17

Supporting Table S11 | Comparison of some chemical shifts (ppm) in $^{13}C\{^{1}H\}$ NMR (126 MHz or 151 MHz, $CDCl_3$, 25 °C) of rotaxane [2]-(*Z*s)-**MR** with its individual components. Both *E* and *Z* isomers of the olefin macrocycle were compared.

Carbon	(*Z*s)-**MA**	[2]-(*Z*s)-**MR**	**OM**	*Δ* ppm
**C^{2}**	41.9	41.8		0.3
**C^{3}**	39.2	39.2		0.0
**CH_3–C^{5}**	16.9	16.9		0.0
**C^{29}**	73.5	73.4		0.1
**C^{30}**	62.0	61.8		0.2
**C^{33}**		108.5, 108.4	108.0, 108.0	0.4, 0.5
**C^{34}**		123.1	123.1	0.0
**C^{35}**		69.8	69.8	0.0
**C^{40}**		67.7	67.8	0.1
**C^{48}**		67.5, 67.4	67.7, 67.5	0.2, 0.1
**H–C^{50}**		128.5, 127.9	128.6, 128.0	0.1, 0.1

^{1}H NMR Comparison of Rotaxane [2]-(*Zs*)-MR with Individual Components

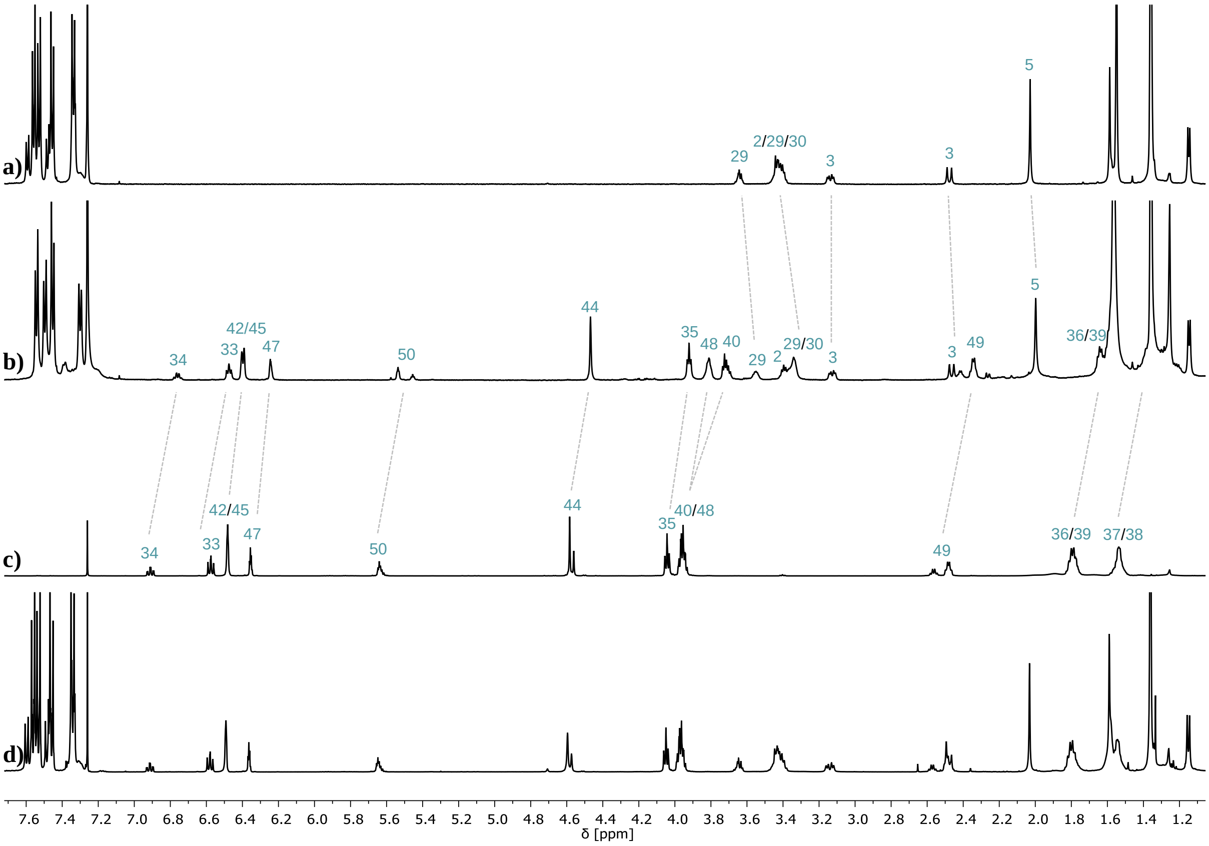

Supporting Figure S52 | ^{1}H NMR spectra comparison of characteristic signals of rotaxane [2]-(*Z*s)-**MR** and the individual components (500 or 600 MHz, $CDCl_3$, 25 °C). a), (*Z*s)-**MA,** b) [2]-(*Z*s)-**MR** (600 MHz, $CDCl_3$, 25 °C), c) macrocycle **OM**, d) ~1:1 mol ratio mixture of (*Z*s)-**MA** and macrocycle **OM**.

^{13}C{^{1}H} NMR Comparison of Rotaxane [2]-(*Zs*)-MR with Individual Components

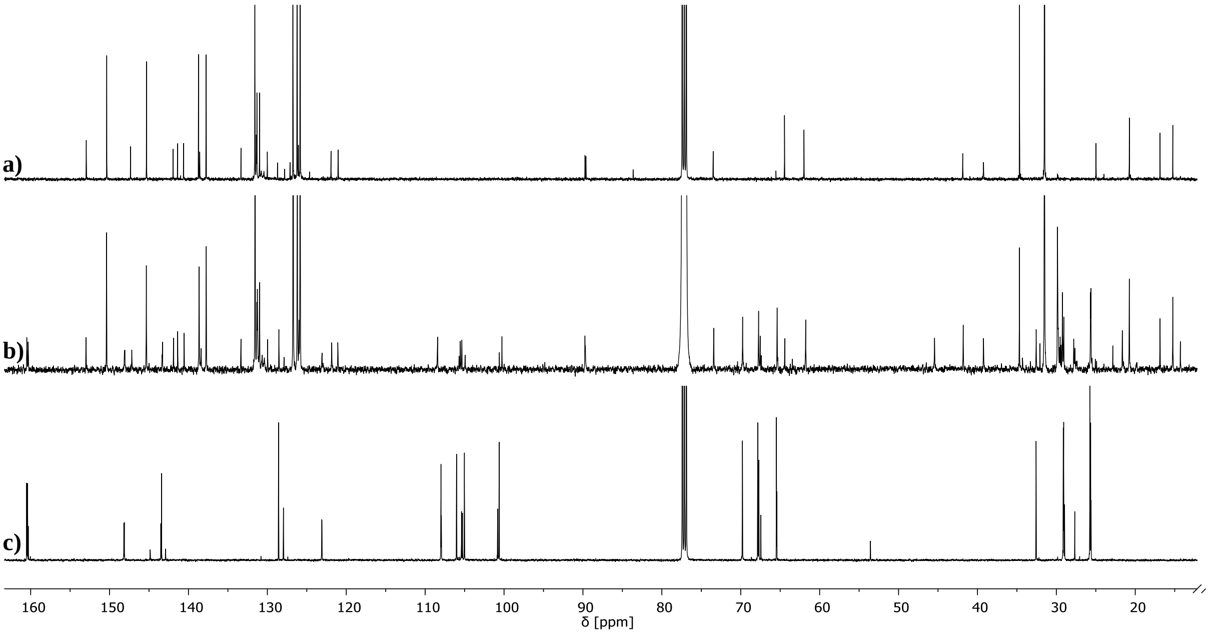

Supporting Figure S53 | ^{13}C{^{1}H} NMR spectra comparison of rotaxane [2]-(*Z*s)-**MR**) and the individual components (126 or 151 MHz, $CDCl_3$, 25 °C). a), (*Z*s)-**MA,** b) [2]-(*Z*s)-**MR**, c) **OM**. Minute changes in chemical shifts were observed between the interlocked compound and its individual components. This, along with the use of ^{1}H HSQC and ^{1}H HMBC allowed for complete assignment of all carbon signals of rotaxane [2]-(*Z*s)-**MR**, see section 2.3.

^{19}F NMR Comparison of Rotaxane [2]-(*Z*s)-MR with Individual Components

Supporting Table S12 | Chemical shifts (ppm) of the fluorine probe of rotaxane [2]-(*Z*s)-**MR** with its individual components, 471 or 476 MHz, $CDCl_3$, 25 °C.

Fluorine	(*Z*s)-**MA**	[2]-(*Z*s)-**MR**	**OM**	*Δ* ppm
**F–C**[31]	---	–155.30 ($Z_{macrocycle}$)	–156.52 ($Z_{macrocycle}$)	1.2
		–155.12 ($E_{macrocycle}$)	–156.49 ($E_{macrocycle}$)	1.4

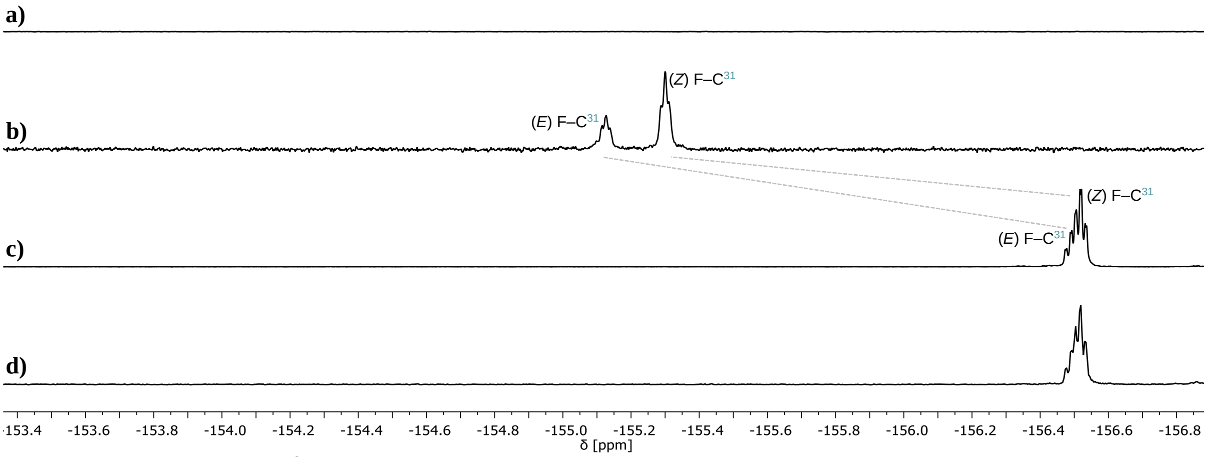

Supporting Figure S54 | ^{19}F NMR spectra comparison of characteristic signals of rotaxane [2]-(*Z*s)-**MR** and the individual components (471 MHz, $CDCl_3$, 25 °C). a), (*Z*s)-**MA,** b) rotaxane [2]-(*Z*s)-**MR** (476 MHz, $CDCl_3$, 25 °C), c) macrocycle **OM**, d) ~1:1 mixture of (*Z*s)-**MA** and macrocycle **OM**.

^{1}H COSY and ^{1}H ROESY NMR Analysis of Rotaxane [2]-(*Z*s)-MR

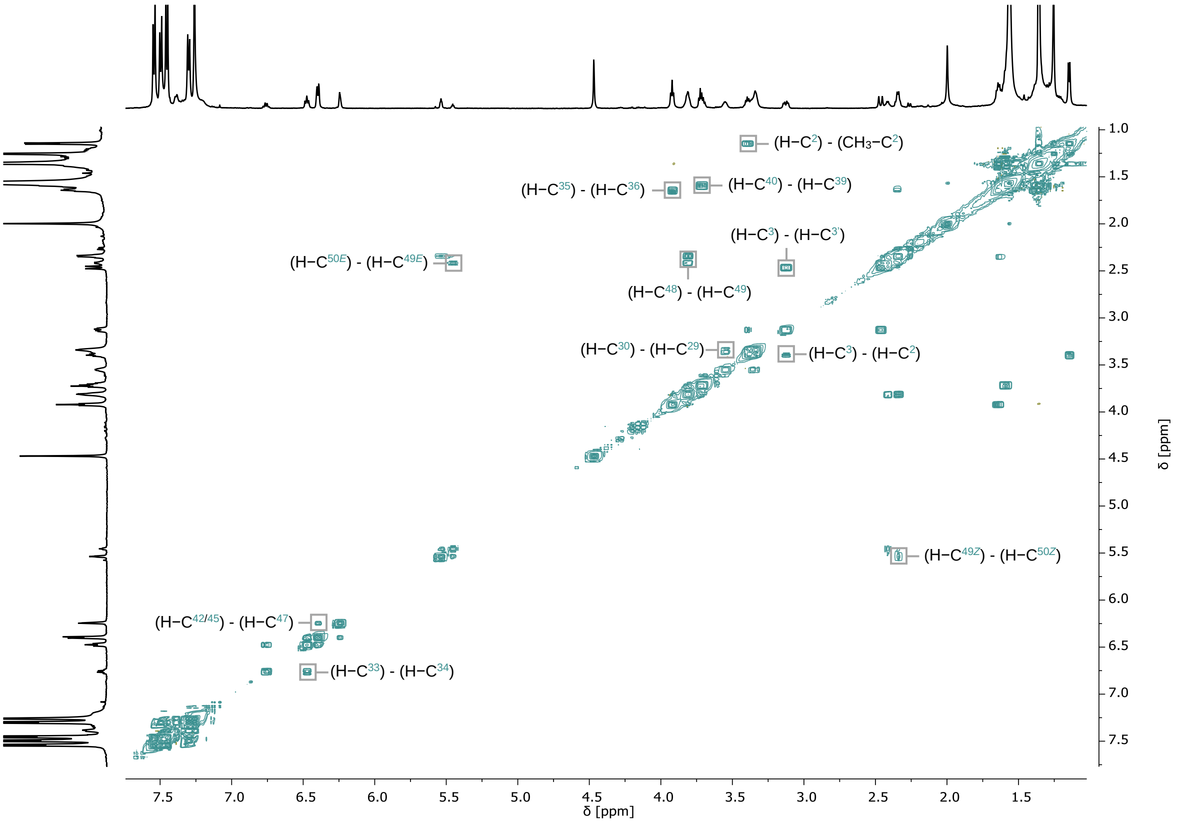

Supporting Figure S55 | ^{1}H COSY NMR of rotaxane [2]-(*Z*s)-**MR** (600 MHz, $CDCl_3$, 25 °C).

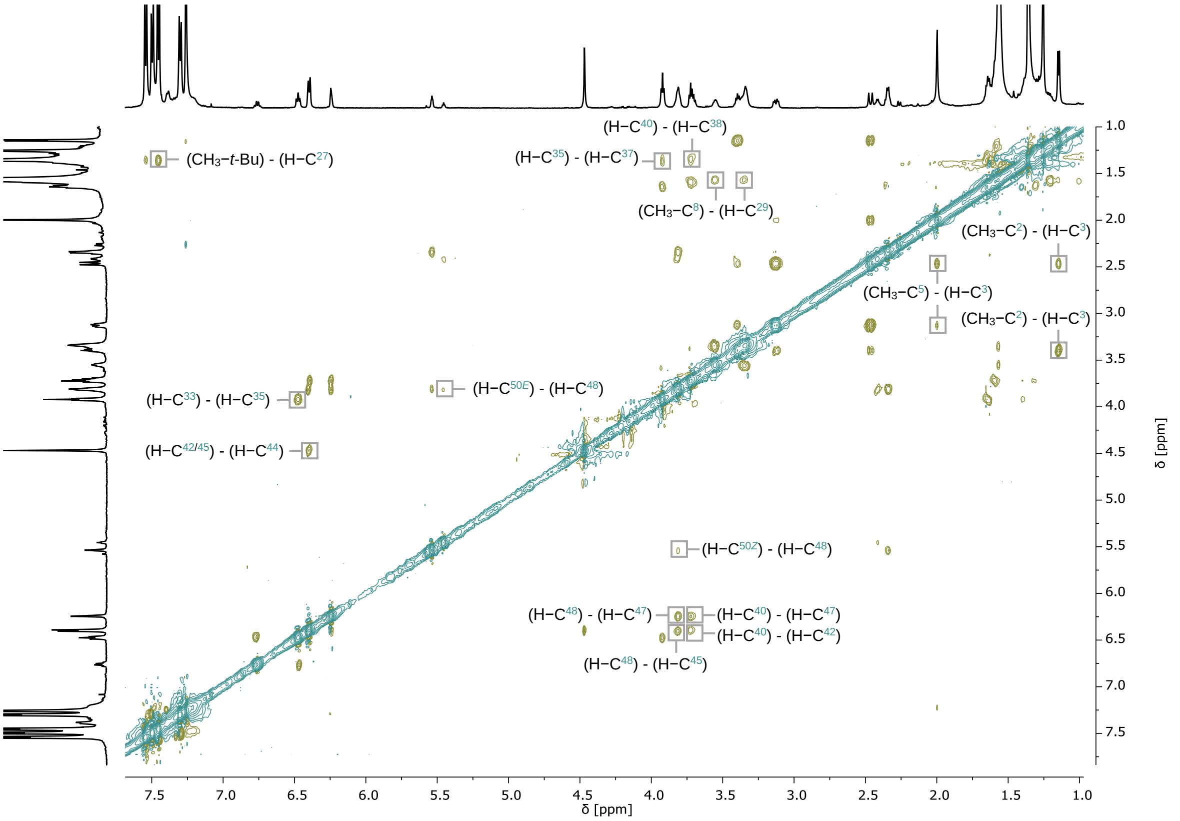

Supporting Figure S56 | ^{1}H ROESY NMR of rotaxane [2]-(*Z*s)-**MR** (600 MHz, $CDCl_3$, 25 °C). Cross-peaks that are also seen in the ^{1}H COSY NMR are not picked.

7.2 DOSY Experiments of Rotaxane [2]-(*Z*s)-MR and Olefin Macrocycle OM

The rotaxane [2]-(*Z*s)-**MR** and olefin macrocycle **OM** were analyzed by DOSY NMR spectroscopy (600 MHz, 25 °C). Assuming the classical spherical approximation for all species, the experimentally obtained diffusion coefficients D were converted to the hydrodynamic radii R_H using the Stokes-Einstein equation (7.1).

$$R_H = \frac{k_B T}{6\pi\eta D} \tag{7.1}$$

Using a viscosity of η = 0.57 mPa s for $CDCl_3$ at 25 °C.

Supporting Table S13 | Diffusion coefficient *D* and corresponding hydrodynamic radii R_H of rotaxane [2]-(*Z*s)-**MR** and olefin macrocycle **OM** in $CDCl_3$ at 25 °C. *We estimate an uncertainty of ±0.02 log(m^2 s^{-1}) in the determination of the diffusion coefficient, therefore hydrodynamic radii have an error of ±0.04 nm.

Species	*D* [log(m^2 s^{-1})]*	R_H [nm]*
[2]-(*Z*s)-**MR**	−9.36	0.89
**OM**	−8.93	0.33

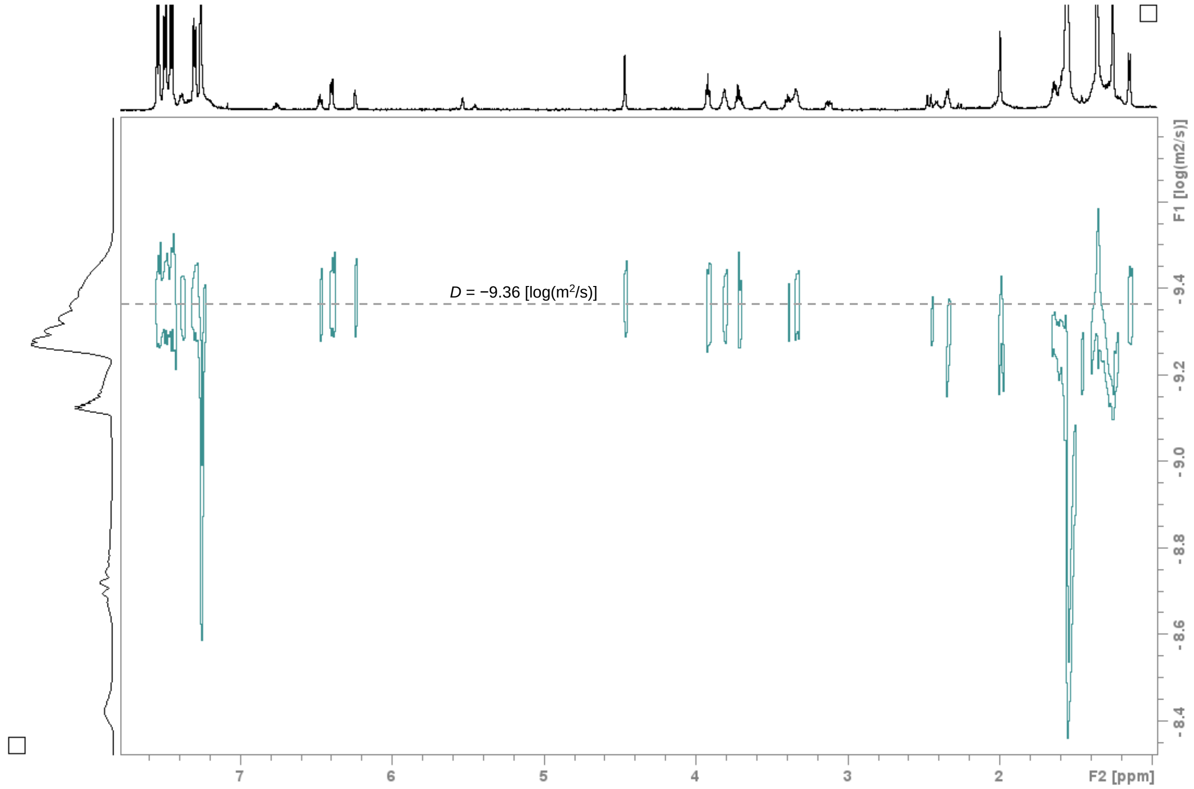

Supporting Figure S57 | DOSY-^{1}H NMR spectrum of [2]-(*Z*s)-**MR** ($CDCl_3$,600 MHz, 25 °C).

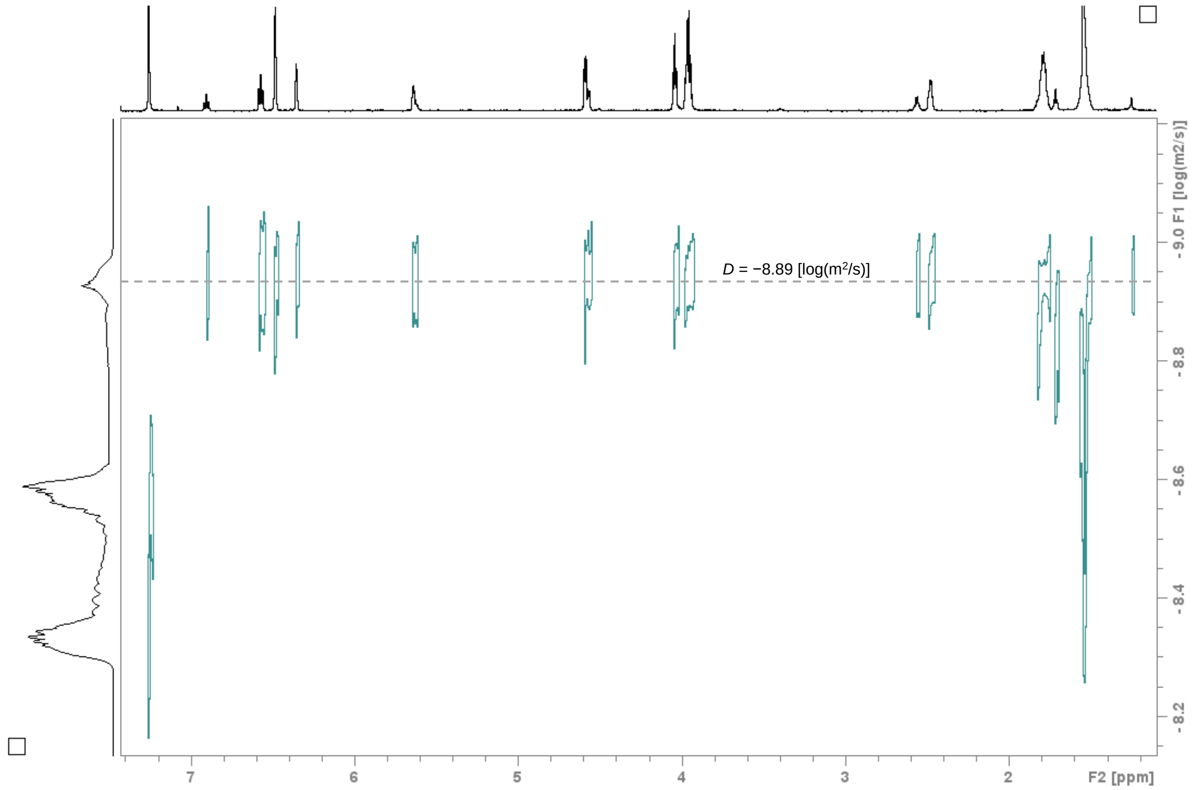

Supporting Figure S58 | DOSY-^{1}H NMR spectrum of olefin macrocycle **OM** ($CDCl_3$, 600 MHz, 25 °C).

7.3 UPLC-HRMS

UPLC-HRMS analysis was performed on (*Z*s)-**MA**, [2]-(*Z*s)-**MR** and olefin macrocycle **OM**. Samples were prepared in MeCN and measured on by UPLC (BEH phenyl cyclohexyl column, eluent: 98% MeCN, 2% water, 40 °C). UPLC traces were plotted from the intensities measured at 270 nm.

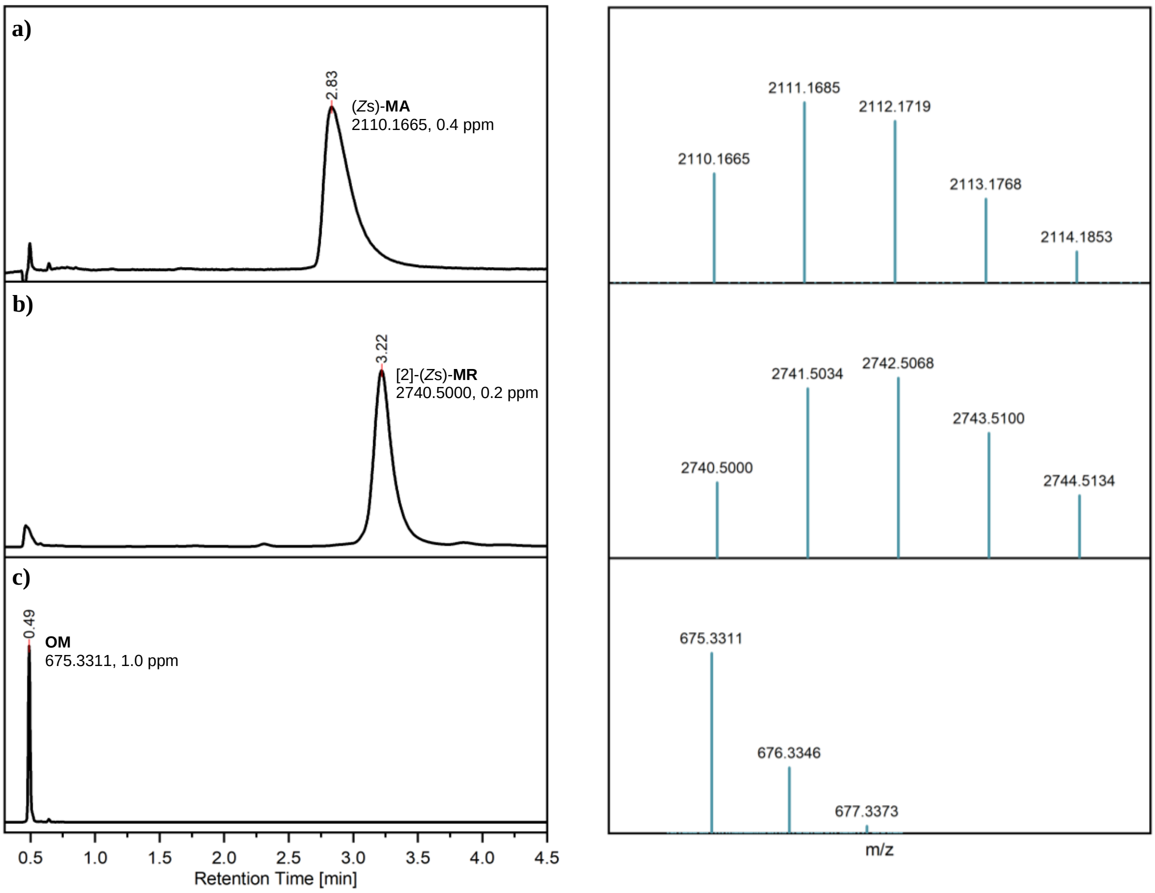

Supporting Figure S59 | UPLC-HRMS analysis (BEH phenyl cyclohexyl column, eluent: 98% MeCN, 2% H_2O, 40 °C). Left: UPLC chromatograms (diode array). Right: HR-MS spectra of the respective peaks of the left chromatogram. a) (*Z*s)-**MA**, $[M+CHOO]^-$, b) rotaxane [2]-(*Z*s)-**MR**, $[M+Na]^+$, c) olefin macrocycle **OM**, $[M+Na]^+$.

7.4 Rotation of rotaxane [2]-(*Z*s)-MR

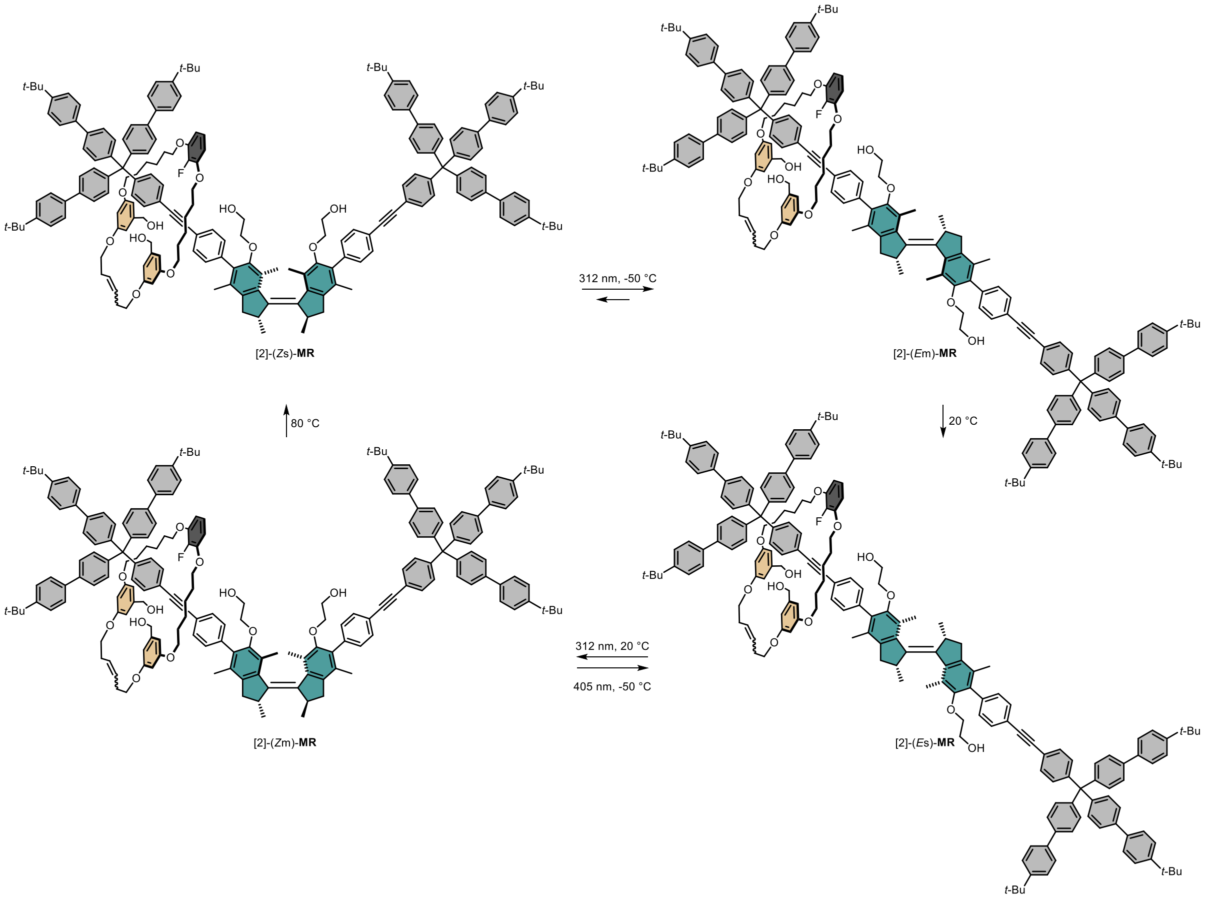

Supporting Figure S60 | Structural depiction of the rotational cycle of rotaxane [2]-(*Z*s)-**MR**.

UPLC-HRMS Analysis of the Rotation of Rotaxane [2]-(*Z*s)-MR

Supporting Table S14 | Rotational cycle of rotaxane [2]-(*Z*s)-**MR** followed by UPLC-HRMS (BEH phenyl cyclohexyl column, eluted: 98% MeCN, 2% H_2O, 40 °C). Retention times shown in min and absorption maxima shown in nm.

	[2]-(*Z*s)-**MR**	[2]-(*E*s)-**MR**	[2]-(*Z*m)-**MR**
**Retention time**	3.35	1.84	3.58
**Absorption maximum**	347	324	365

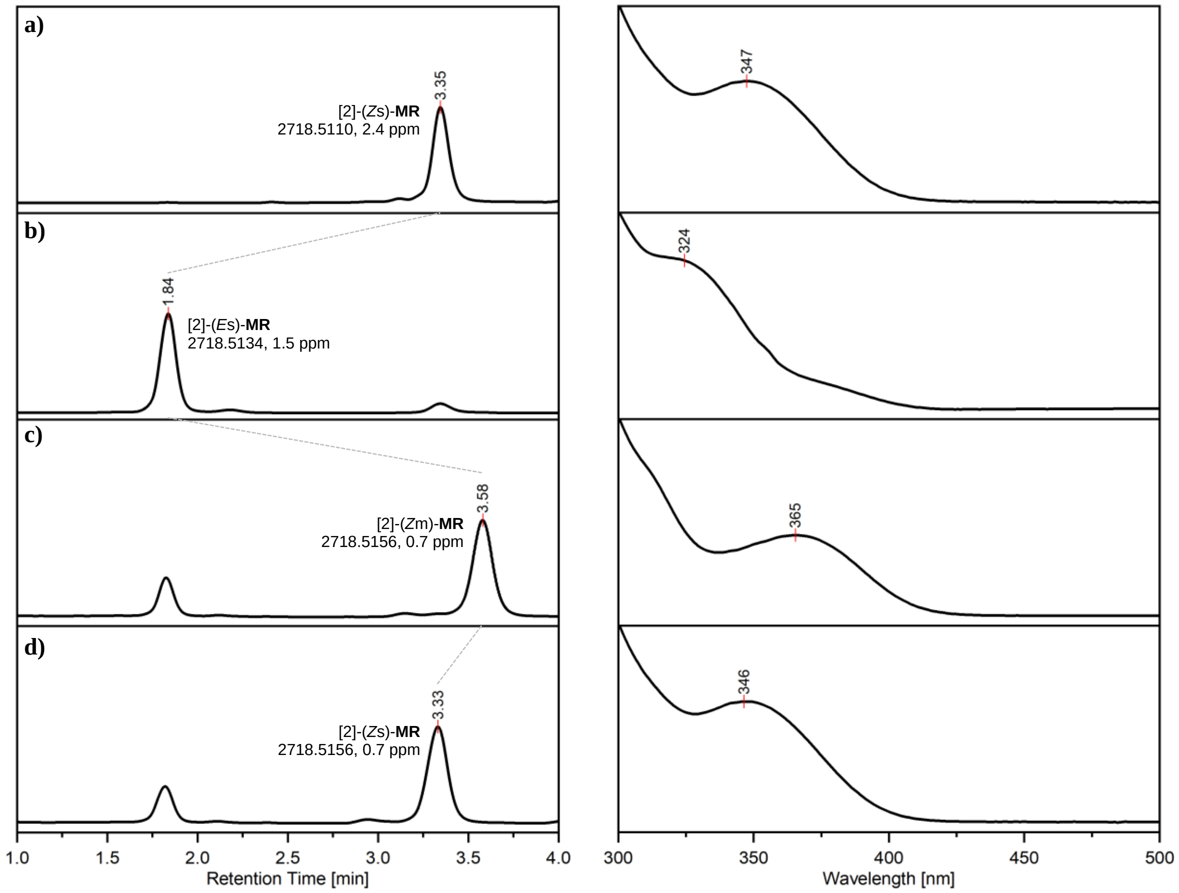

Supporting Figure S61 | UPLC-HRMS analysis of the rotational cycle of [2]-(*Z*s)-**MR** (BEH phenyl cyclohexyl column, eluent: 98% MeCN, 2% water, 40 °C). Left: UPLC chromatograms (diode array), highlighted peak indicates the newly formed species after each step. Masses are given as the proton adduct ($[M+H]^+$). Right: extracted UV-vis spectrum from the respective highlighted peak of the left chromatogram. a) Initial [2]-(*Z*s)-**MR** chromatogram, b) after irradiation with 312 nm at –50 °C and subsequent thermal relaxation at 20 °C, c) after irradiation with 312 nm at 20 °C, d) after thermal relaxation at 90 °C reforming the initial species.

^{1}H NMR Analysis of the Rotation of Rotaxane [2]-(*Z*s)-MR

Supporting Table S15 | Chemical shifts (ppm) of characteristic proton signals during the rotation of rotaxane [2]-(*Z*s)-**MR** (600 MHz, toluene-d_8, c = 0.75 mM, 25 °C). ^{1}H COSY and ^{1}H ROESY was used for the assignment of the protons.

	[2]-(*Z*s)-**MR**	[2]-(*E*s)-**MR**	[2]-(*Z*m)-**MR**
**CH_3–C^2**	1.16	1.27	1.42
**CH_3–C^5**	2.06	1.92	2.02
**CH_3–C^8**	1.89	2.58	1.71
**H–C^2**	3.36	3.12	3.45
**H–C^3**	3.10, 2.41	2.69, 2.24	3.01, 2.63

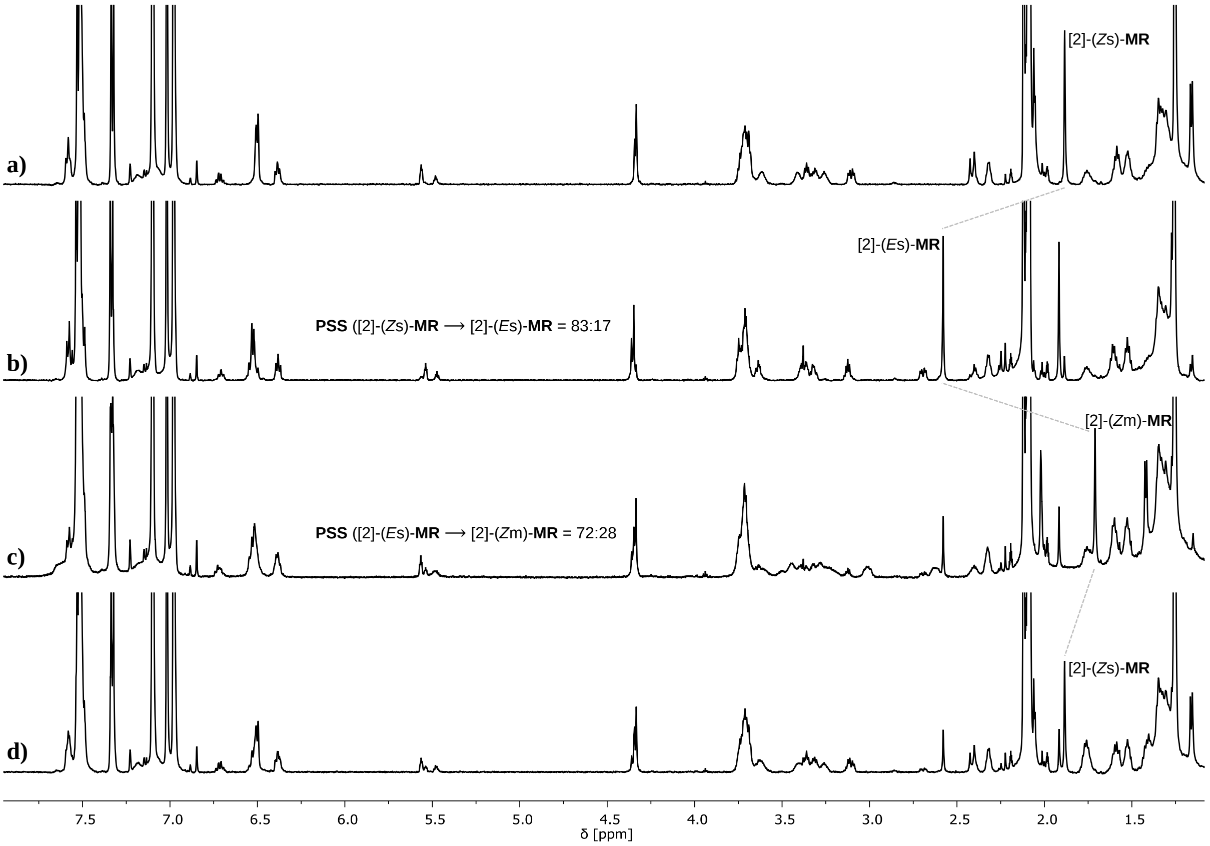

Supporting Figure S62 | ^{1}H NMR spectra (600 MHz, toluene-d_8, c = 0.75 mM, 25 °C) of the rotational cycle of [2]-(*Z*s)-**MR**, sequence from top to bottom. a) Initial rotaxane [2]-(*Z*s)-**MR** spectrum, b) after irradiation with 312 nm at –50 °C and subsequent thermal relaxation at 20 °C, c) after irradiation with 312 nm at 20 °C, d) after thermal relaxation at 90 °C.

^{19}F NMR Analysis of the Rotation of Rotaxane [2]-(*Z*s)-MR

Supporting Table S16 | Chemical shifts (ppm) of the fluorine probe during the rotation of rotaxane [2]-(*Z*s)-**MR** (476 MHz, toluene-d_8, c = 0.75 mM, 25 °C).

	[2]-(*Z*s)-**MR**	[2]-(*E*s)-**MR**	[2]-(*Z*m)-**MR**
(*Z*) **F–C**[31]	–154.67	–154.62	–154.67
(*E*) **F–C**[31]	–154.49	–154.43	–154.48

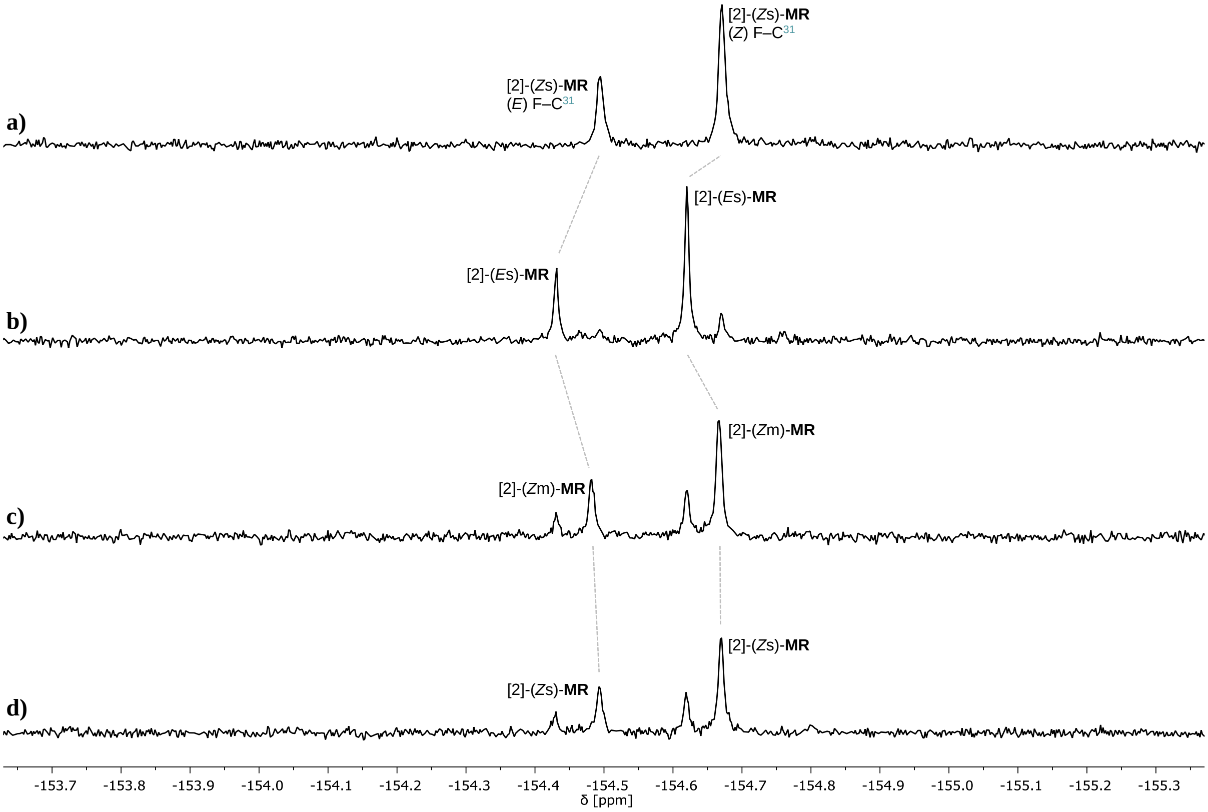

Supporting Figure S63 | ^{19}F{^{1}H} NMR spectra (476 MHz, toluene-d_8, c = 0.75 mM, 25 °C) of the rotational cycle of [2]-(*Z*s)-**MR**, sequence from top to bottom. a) Initial rotaxane [2]-(*Z*s)-**MR** spectrum, b) after irradiation with 312 nm at –50 °C and subsequent thermal relaxation at 20 °C, c) after irradiation with 312 nm at 20 °C, d) after thermal relaxation at 90 °C.

8. Single Crystal X-Ray Crystallography

8.1 Crystal Structure of S7

Crystal obtained from slow evaporation of EtOAc.

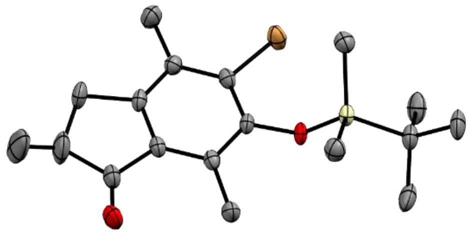

Supporting Table S17 | Crystal data and structure refinement for **S7**.

Identification code	2410222
Empirical formula	$C_{18}H_{27}BrO_2Si$
Formula weight	383.39
Temperature/K	102.00
Crystal system	orthorhombic
Space group	$Pca2_1$
a/Å	12.0895(8)
b/Å	14.3333(9)
c/Å	10.9048(6)
α/°	90
β/°	90
γ/°	90
Volume/Å^3	1889.6(2)
Z	4
ρ_{calc}g/cm^3	1.348
μ/mm^{-1}	2.244
F(000)	800.0
Crystal size/mm^3	0.1 × 0.1 × 0.1
Radiation	MoKα (λ = 0.71073)
2Θ range for data collection/°	4.408 to 52.832
Index ranges	$-15 \le h \le 15$, $-17 \le k \le 17$, $-13 \le l \le 13$
Reflections collected	15699
Independent reflections	3816 [R_{int} = 0.0423, R_{sigma} = 0.0482]
Data/restraints/parameters	3816/1/207
Goodness-of-fit on F^2	1.037
Final R indexes [I>=2σ (I)]	R_1 = 0.0311, wR_2 = 0.0692
Final R indexes [all data]	R_1 = 0.0360, wR_2 = 0.0709
Largest diff. peak/hole / e Å^{-3}	0.62/-0.33
Flack parameter	0.046(5)

8.2 Crystal Structure of S10

Crystal obtained from slow evaporation of $CDCl_3$.

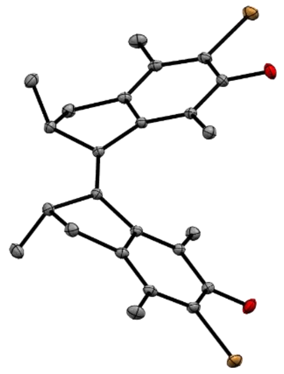

Supporting Table S18 | Crystal data and structure refinement for **S10**.

Identification code	2410147
Empirical formula	$C_{24}H_{26}O_2Br_2$
Formula weight	506.27
Temperature/K	100.00
Crystal system	monoclinic
Space group	*C*2/c
a/Å	5.7774(4)
b/Å	16.5549(13)
c/Å	21.7553(11)
α/°	90
β/°	93.240(2)
γ/°	90
Volume/Å^3	2077.4(2)
Z	4
ρ_{calc}g/cm^3	1.619
μ/mm^{-1}	3.920
F(000)	1024.0
Crystal size/mm^3	0.768 × 0.33 × 0.152
Radiation	MoKα (λ = 0.71073)
2Θ range for data collection/°	3.75 to 72.992
Index ranges	$-9 \le h \le 9$, $-27 \le k \le 27$, $-36 \le l \le 36$
Reflections collected	97344
Independent reflections	5095 [R_{int} = 0.0910, R_{sigma} = 0.0300]
Data/restraints/parameters	5095/0/131
Goodness-of-fit on F^2	1.046
Final R indexes [I>=2σ (I)]	R_1 = 0.0327, wR_2 = 0.0833
Final R indexes [all data]	R_1 = 0.0425, wR_2 = 0.0885
Largest diff. peak/hole / e Å^{-3}	0.87/-0.75

9 References

[1] G. M. Sheldrick, *SADABS: Program for Empirical Absorption Correction of Area Detector Data*, University of Göttingen, **1996**.

[2] G. M. Sheldrick, "SHELXT – Integrated space-group and crystal-structure determination" *Acta Crystallogr. A* **2015**, *71*, 3–8.

[3] G. M. Sheldrick, "Crystal structure refinement with SHELXL" *Acta Crystallogr. C* **2015**, *71*, 3–8.

[4] O. V. Dolomanov, L. J. Bourhis, R. J. Gildea, J. a. K. Howard, H. Puschmann, "OLEX2: a complete structure solution, refinement and analysis program" *J. Appl. Cryst.* **2009**, *42*, 339–341.

[5] A. B. S. Elliott, R. Horvath, X. Z. Sun, M. G. Gardiner, K. Müllen, N. T. Lucas, M. W. George, K. C. Gordon, "Long-Lived Charge Transfer Excited States in HBC-Polypyridyl Complex Hybrids" *Inorg. Chem.* **2016**, *55*, 4710–4719.

[6] H. Kawai, T. Umehara, K. Fujiwara, T. Tsuji, T. Suzuki, "Dynamic covalently bonded rotaxanes cross-linked by imine bonds between the axle and ring: Inverse temperature dependence of subunit mobility" *Angew. Chem. Int. Ed.* **2006**, *45*, 4281–4286.

[7] T. M. Neubauer, T. van Leeuwen, D. Zhao, A. S. Lubbe, J. C. M. Kistemaker, B. L. Feringa, "Asymmetric Synthesis of First Generation Molecular Motors" *Org. Lett.* **2014**, *16*, 4220–4223.

[8] A. V. Aggarwal, S.-S. Jester, S. M. Taheri, S. Förster, S. Höger, "Molecular Spoked Wheels: Synthesis and Self-Assembly Studies on Rigid Nanoscale 2D Objects" *Chem. Eur. J.* **2013**, *19*, 4480–4495.

[9] N. G. White, P. J. Costa, S. Carvalho, V. Félix, P. D. Beer, "Increased Halide Recognition Strength by Enhanced Intercomponent Preorganisation in Triazolium Containing [2]Rotaxanes" *Chem. Eur. J.* **2013**, *19*, 17751–17765.

[10] R. Dorel, B. L. Feringa, "Stereodivergent Anion Binding Catalysis with Molecular Motors" *Angew. Chem. Int. Ed.* **2020**, *59*, 785–789.

[11] N. Hoffmann, J.-P. Pete, "Intramolecular [2+2] Photocycloaddition of Bichromophoric Derivatives of 3,5-Dihydroxybenzoic Acid and 3,5-Dihydroxybenzonitrile" *Synthesis* **2004**, *112*, 1236–1242.

[12] T. Wachsmuth, R. Kluifhooft, M. Müller, L. Zeiß, M. Kathan, "A molecular machine directs the synthesis of a catenane" *Science* **2025**, *389*, 526–531.

[13] T. Van Leeuwen, J. Gan, J. C. M. Kistemaker, S. F. Pizzolato, M. C. Chang, B. L. Feringa, "Enantiopure Functional Molecular Motors Obtained by a Switchable Chiral-Resolution Process" *Chem. Eur. J.* **2016**, *22*, 7054–7058.

[14] M. Kathan, S. Crespi, N. O. Thiel, D. L. Stares, D. Morsa, J. de Boer, G. Pacella, T. van den Enk, P. Kobauri, G. Portale, C. A. Schalley, B. L. Feringa, "A light-fuelled nanoratchet shifts a coupled chemical equilibrium" *Nat. Nanotechnol.* **2022**, *17*, 159–165.

[15] S. van Vliet, J. Sheng, C. N. Stindt, B. L. Feringa, "All-visible-light-driven salicylidene schiff-base-functionalized artificial molecular motors" *Nat. Commun.* **2024**, *15*, 6461.

10 Spectra Appendix

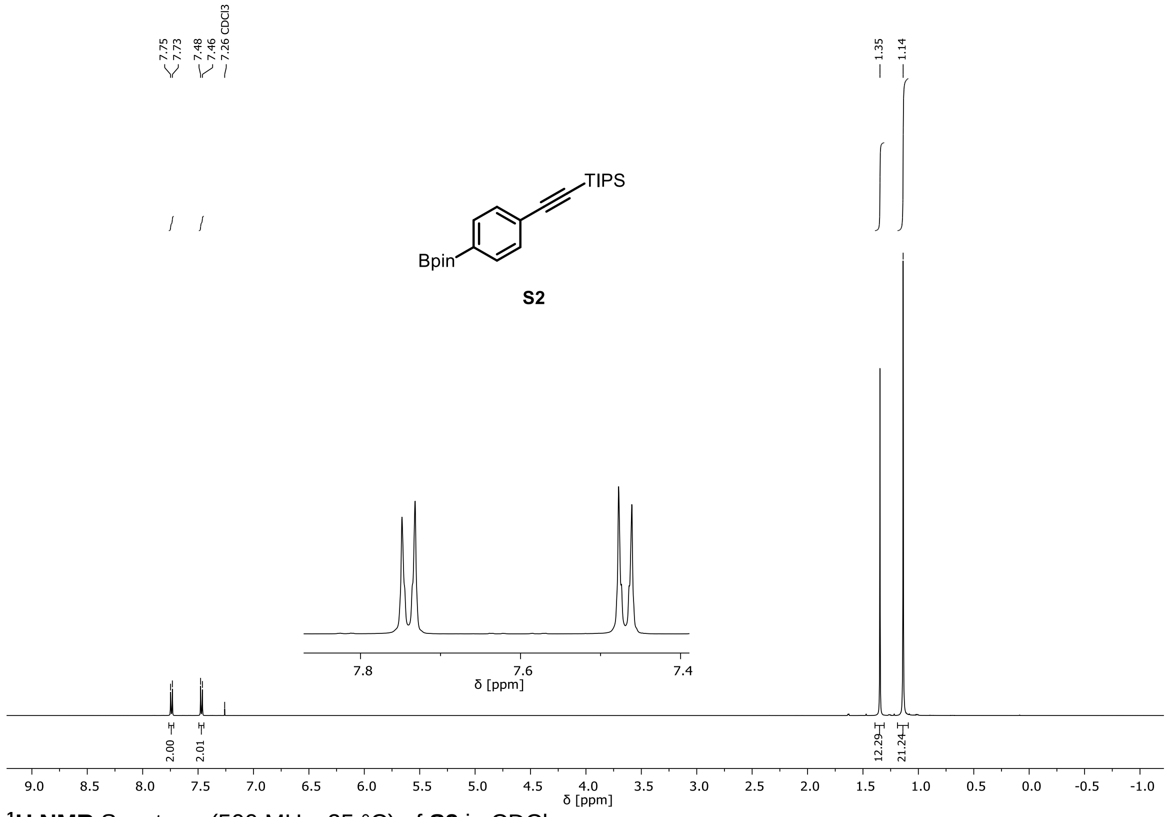

^{1}H NMR Spectrum (500 MHz, 25 °C) of **S2** in $CDCl_3$.

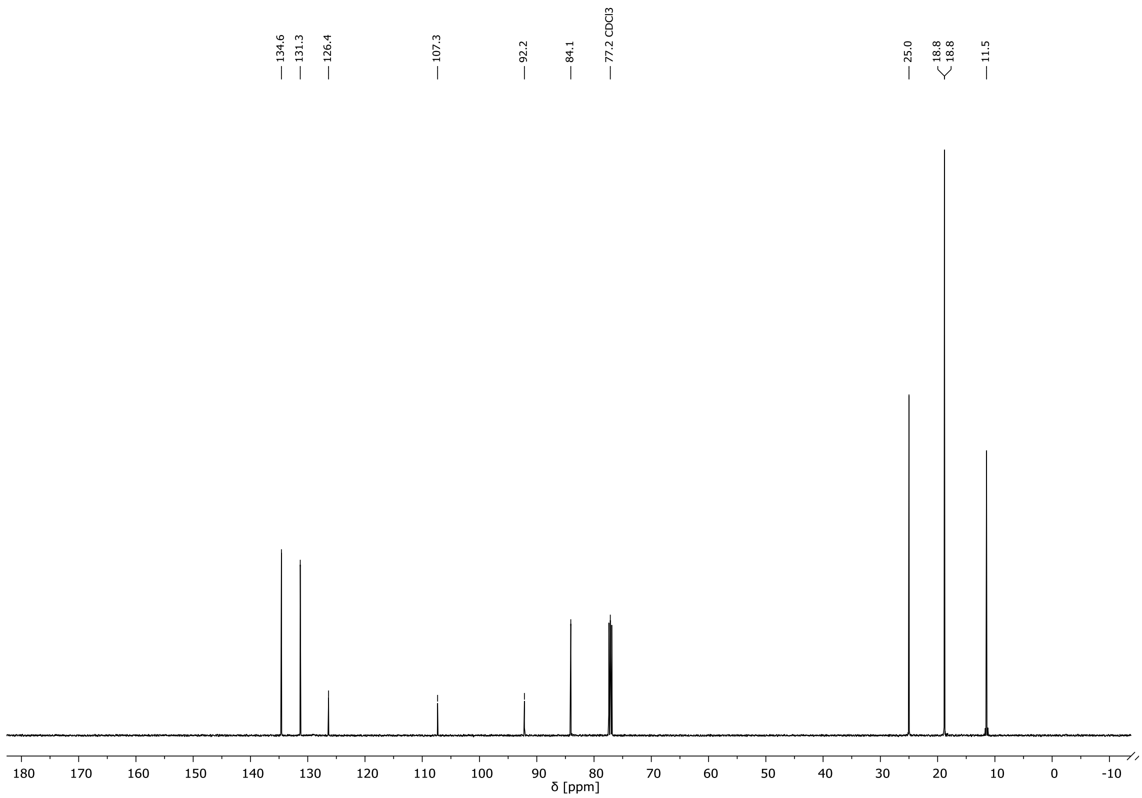

^{13}C NMR Spectrum (126 MHz, 25 °C) of **S2** in $CDCl_3$.

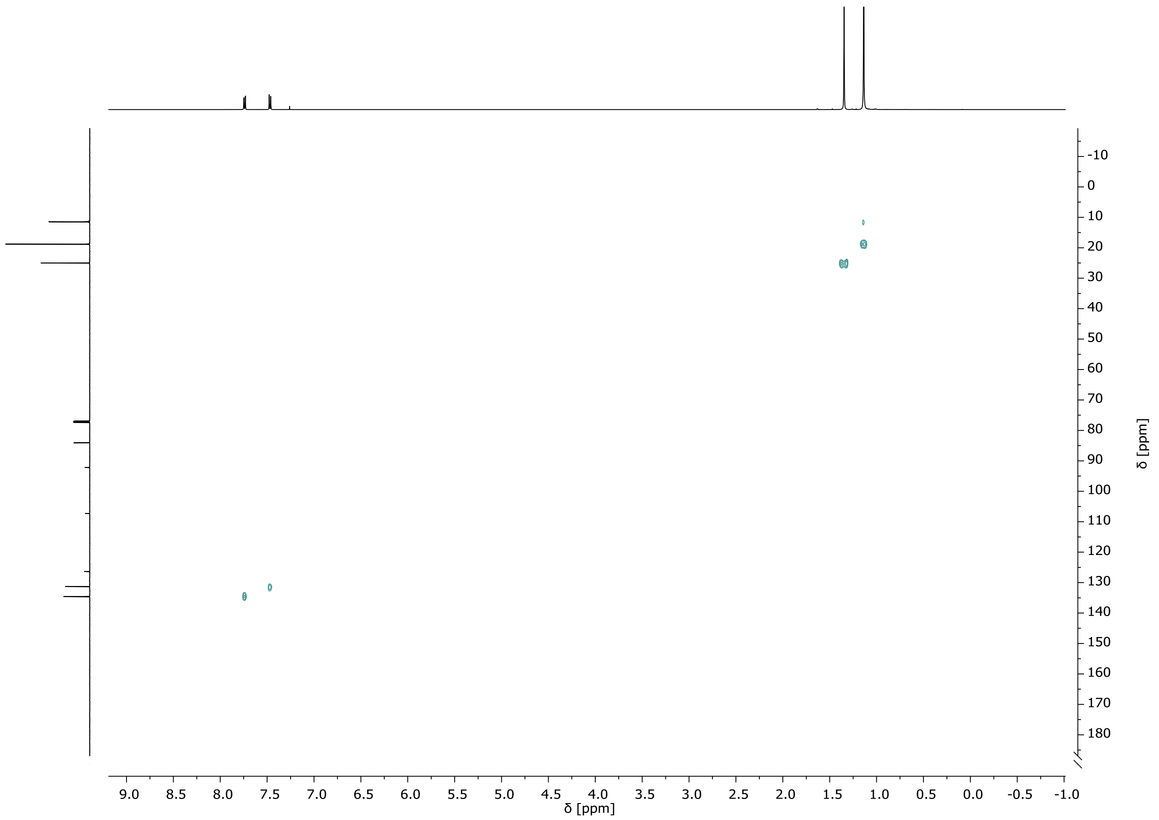

^{1}H, ^{13}C HSQC NMR Spectrum (500 MHz, 25 °C) of **S2** in $CDCl_3$.

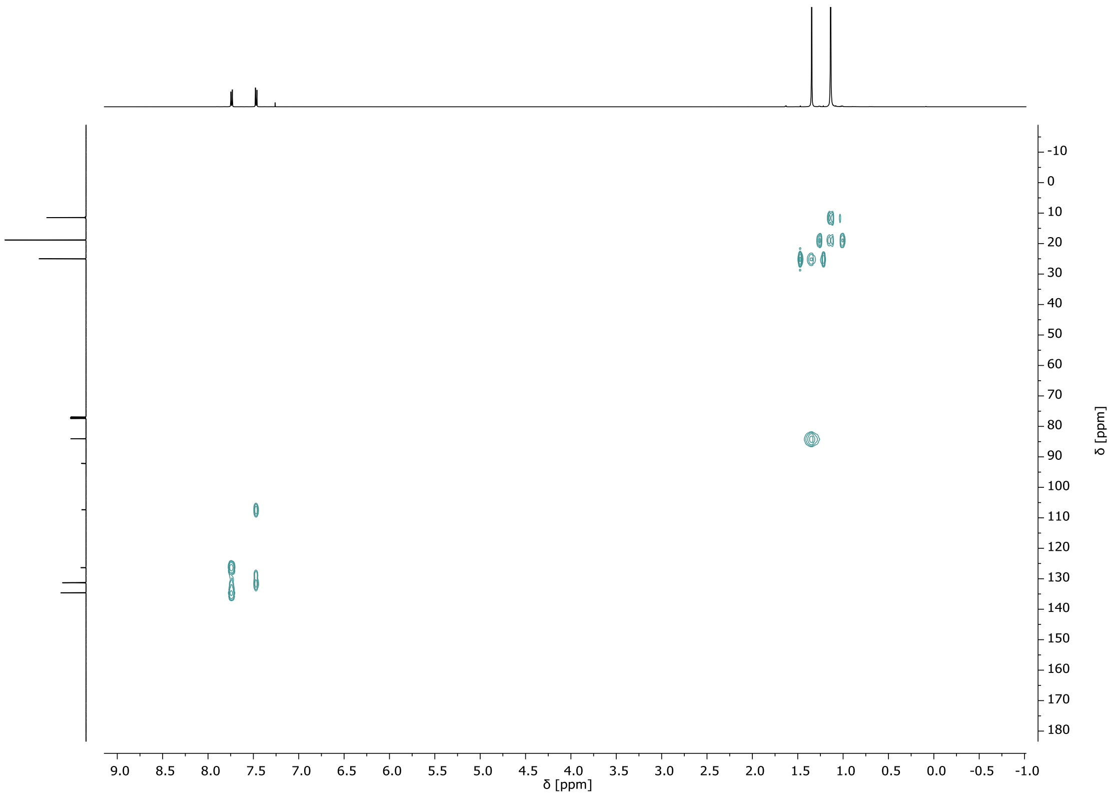

^{1}H, ^{13}C HMBC NMR Spectrum (500 MHz, 25 °C) of **S2** in $CDCl_3$.

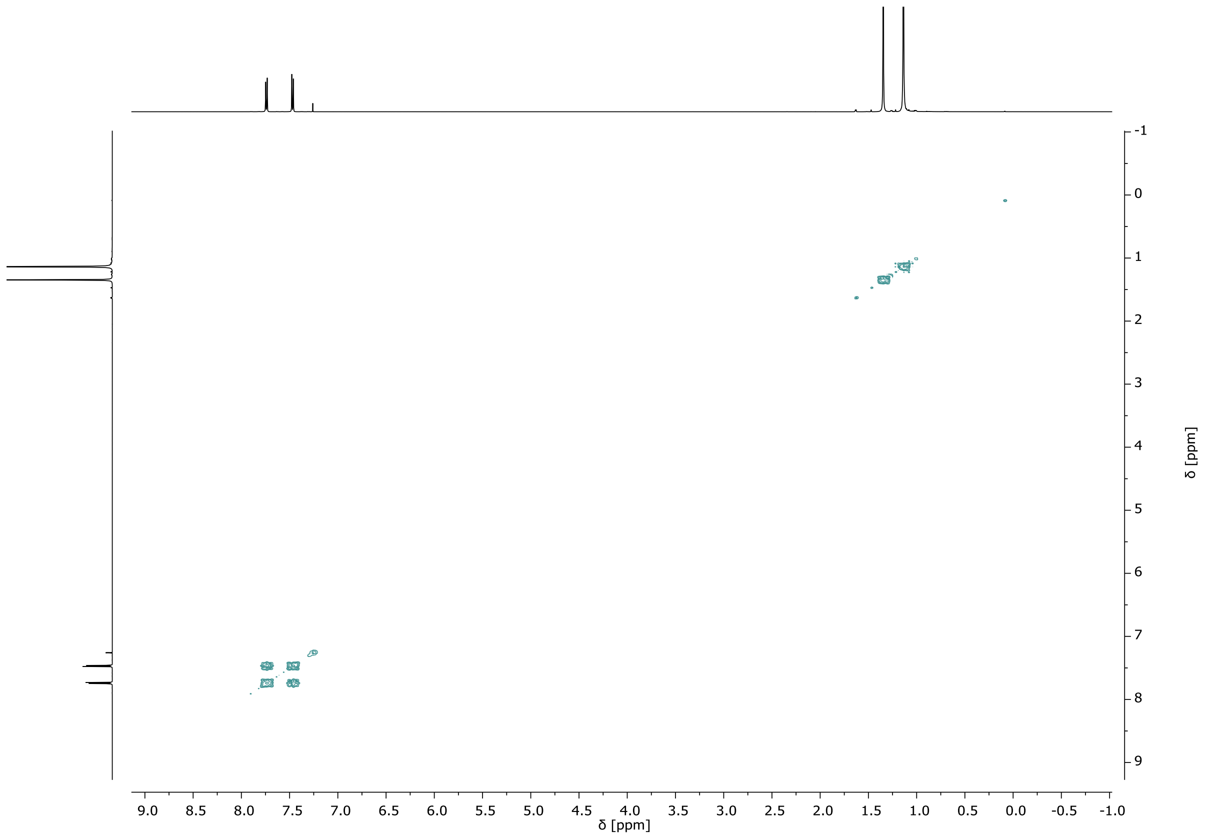

^{1}**H COSY NMR** Spectrum (500 MHz, 25 °C) of **S2** in $CDCl_3$.

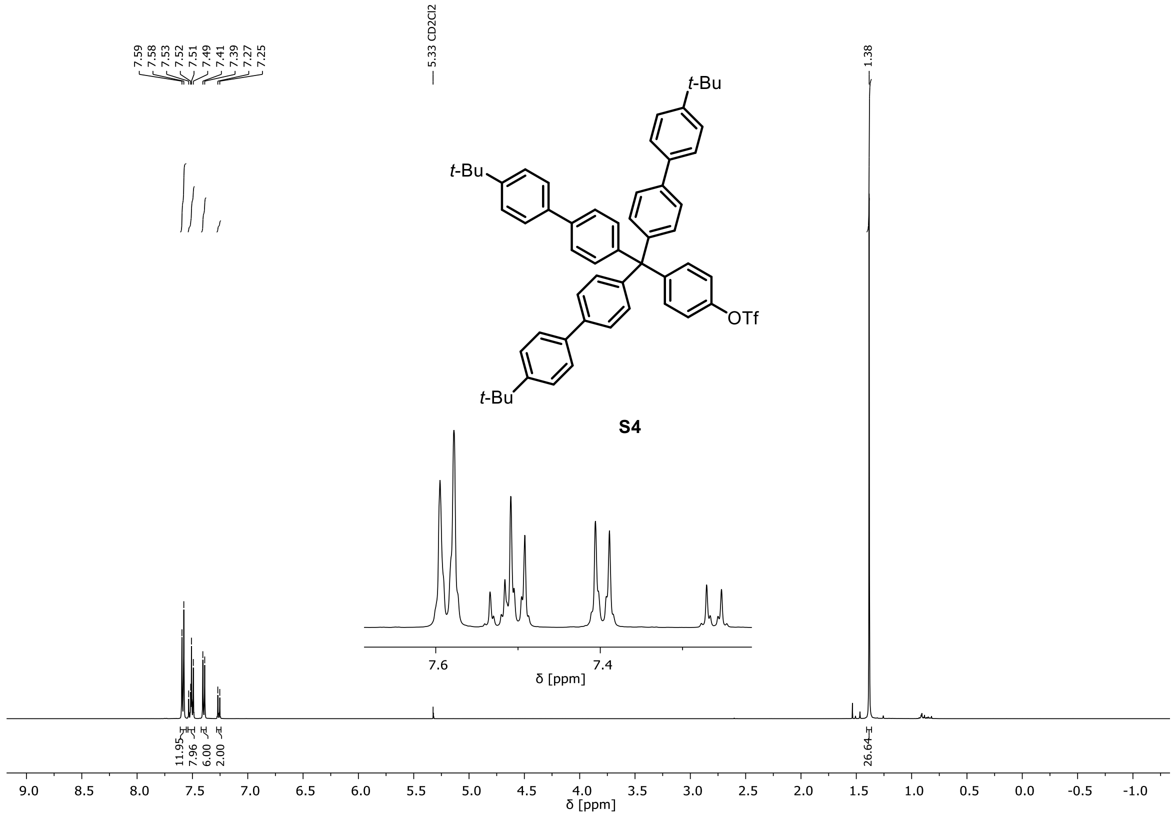

^{1}H NMR Spectrum (500 MHz, 25 °C) of **S4** in CD_2Cl_2.

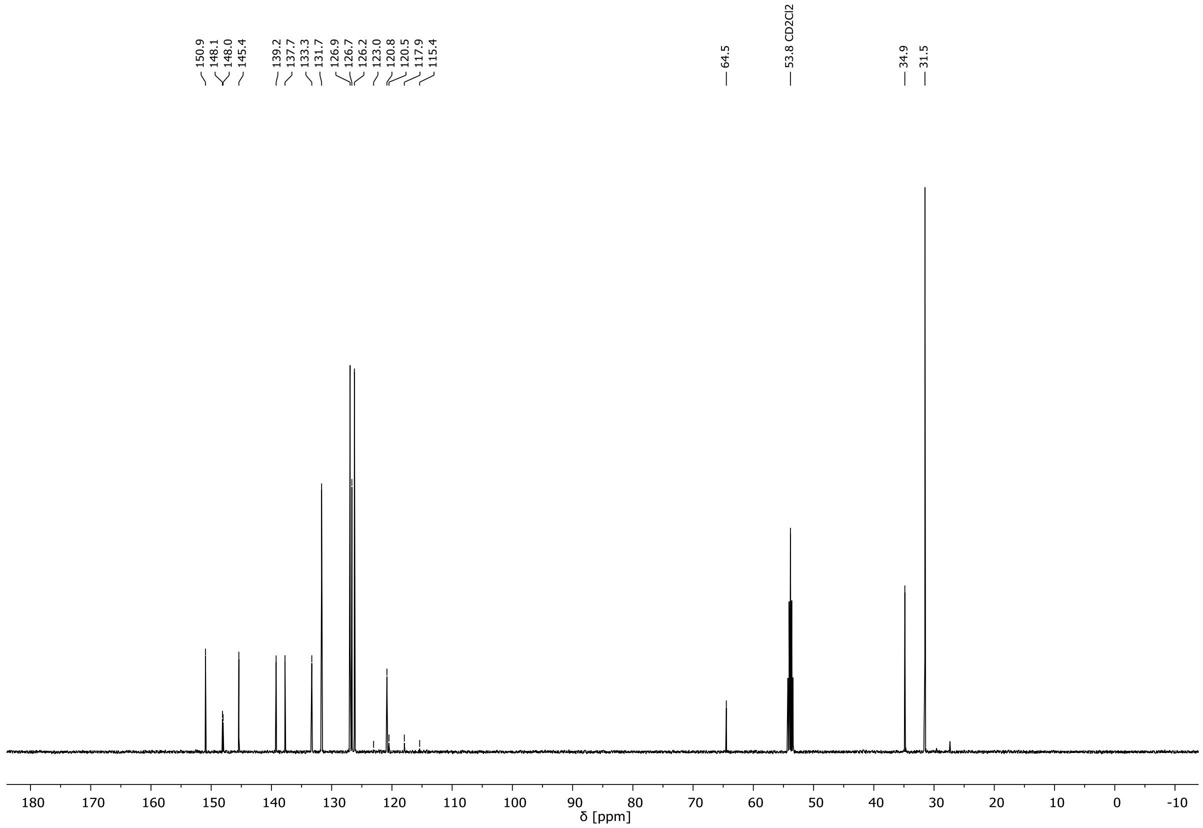

^{13}C NMR Spectrum (126 MHz, 25 °C) of **S4** in CD_2Cl_2.

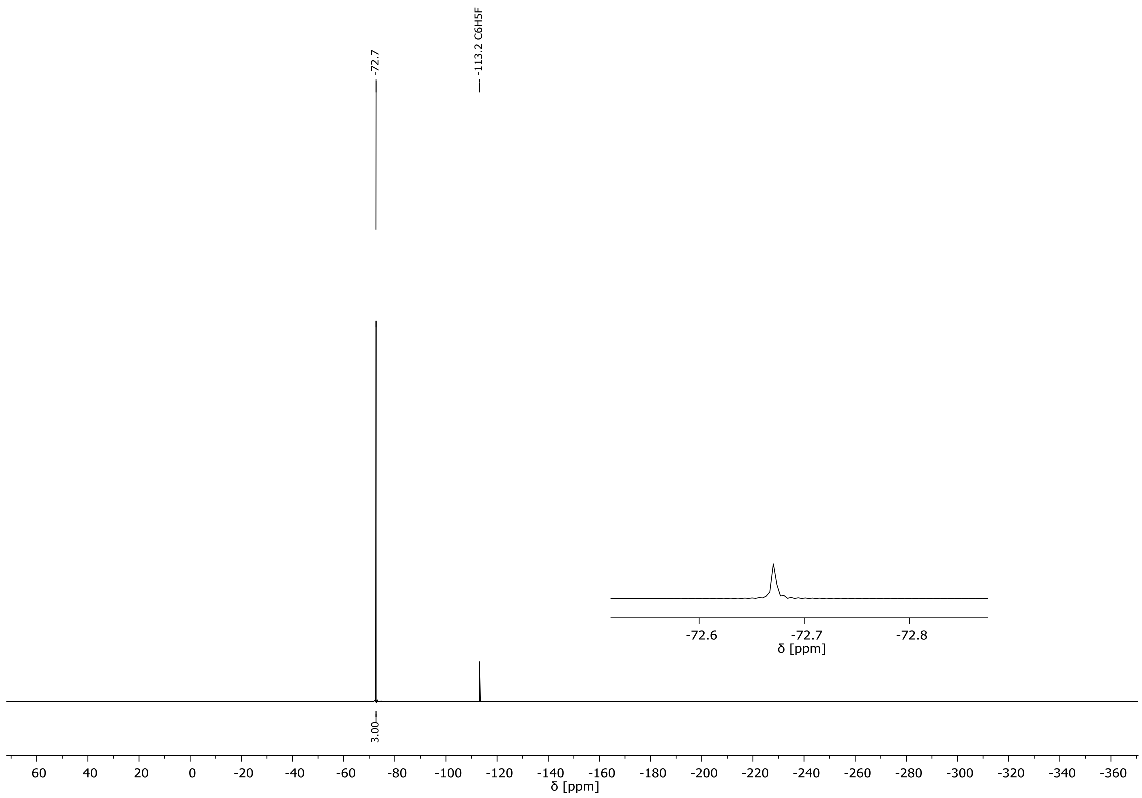

^{19}F NMR Spectrum (471 MHz, 25 °C) of **S4** in CD_2Cl_2.

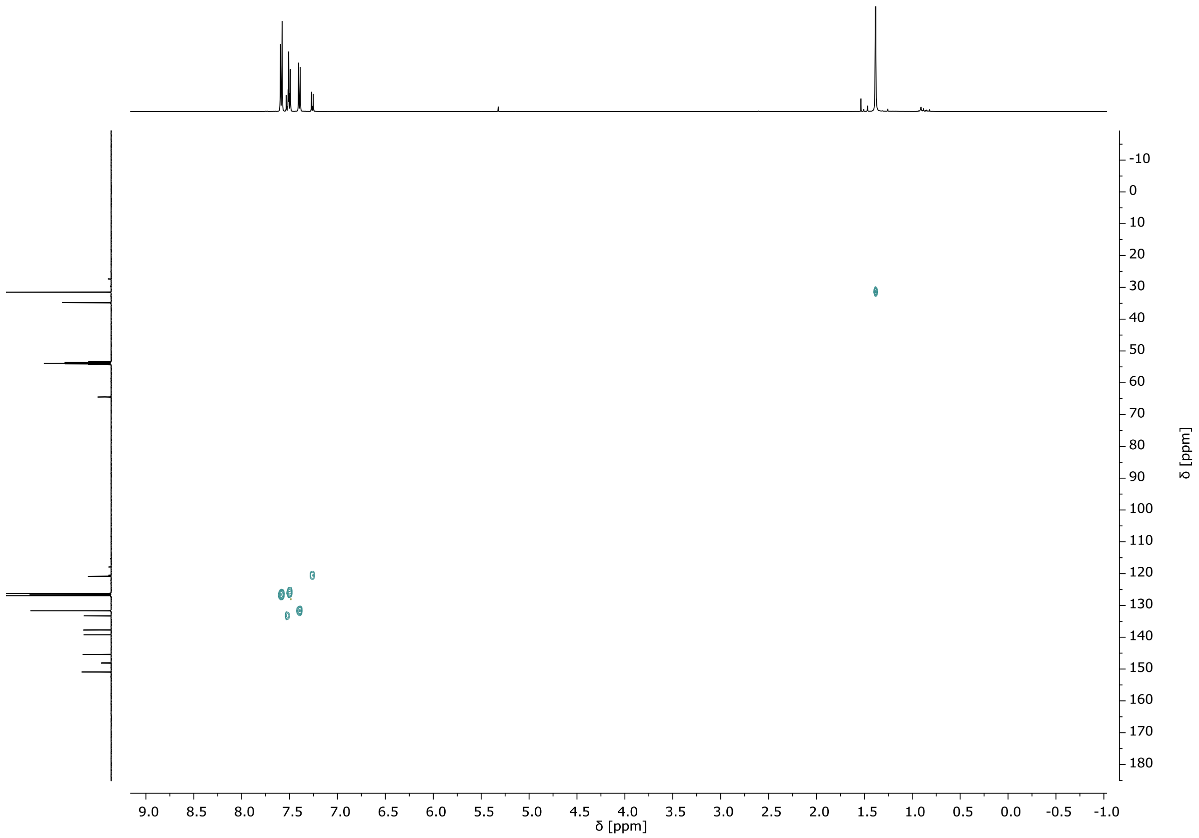

^{1}H, ^{13}C HSQC NMR Spectrum (500 MHz, 25 °C) of **S4** in CD_2Cl_2.

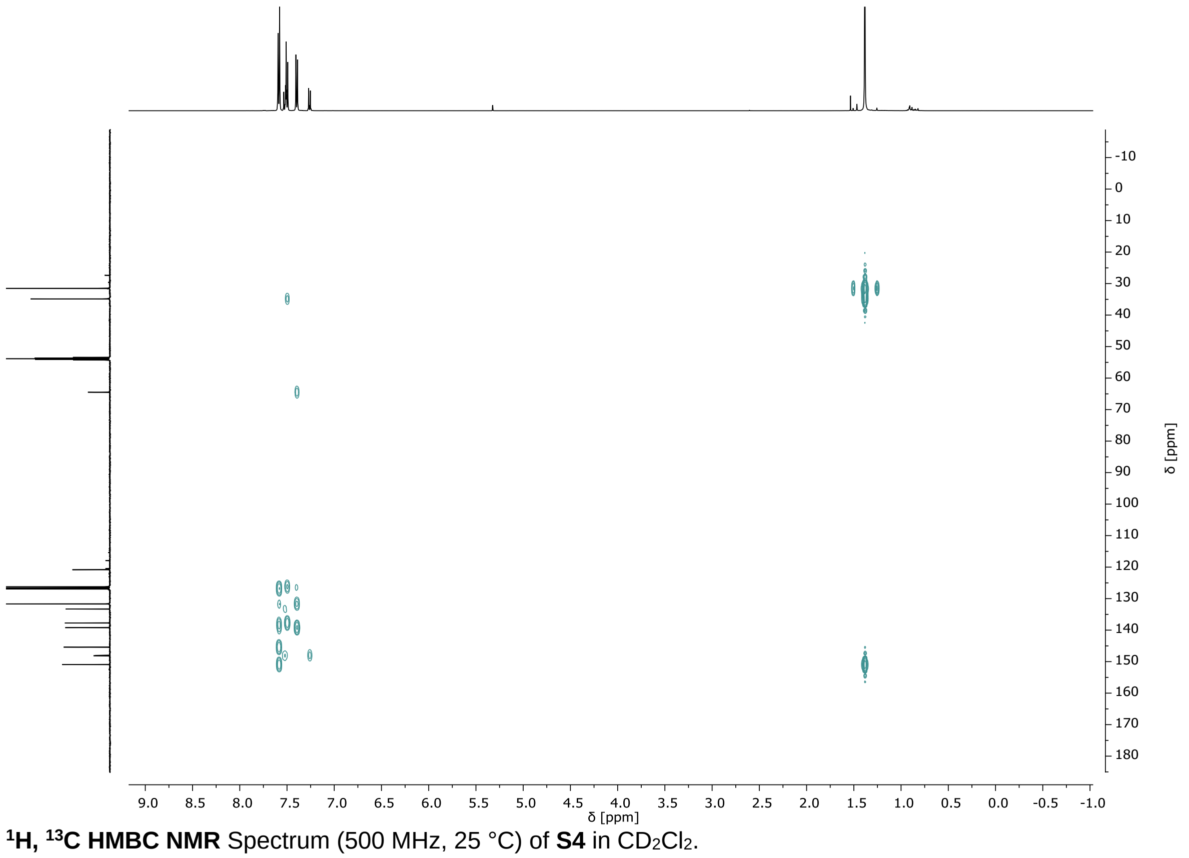

^{1}H, ^{13}C **HMBC NMR** Spectrum (500 MHz, 25 °C) of **S4** in CD_2Cl_2.

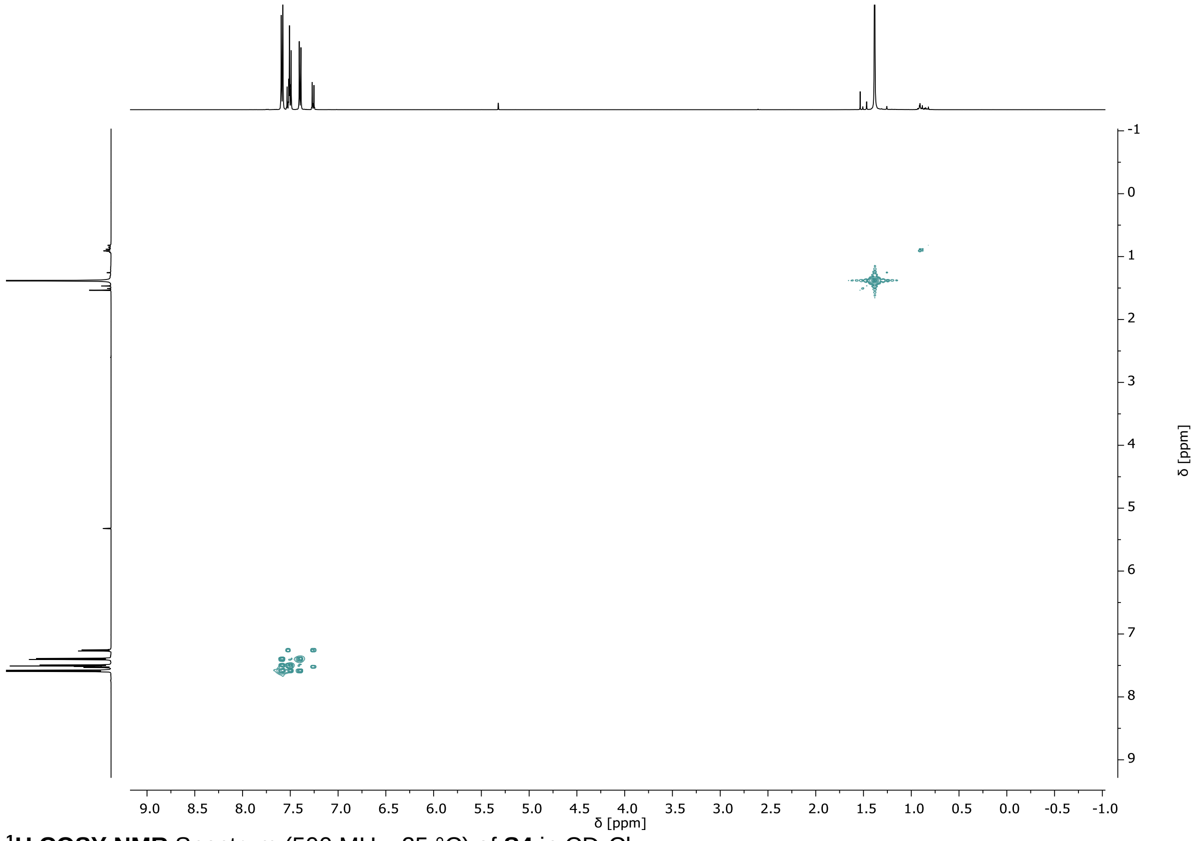

^{1}**H COSY NMR** Spectrum (500 MHz, 25 °C) of **S4** in CD_2Cl_2.

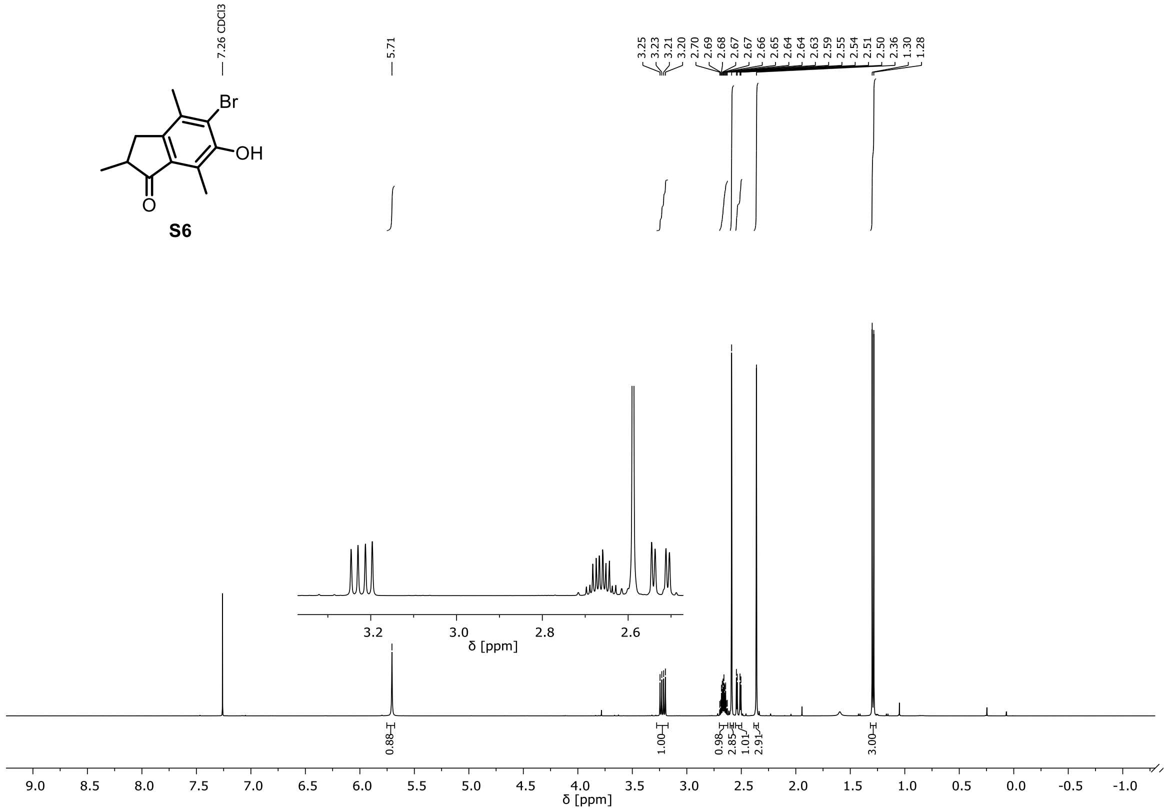

S6

¹H NMR Spectrum (500 MHz, 25 °C) of **S6** in $CDCl_3$.

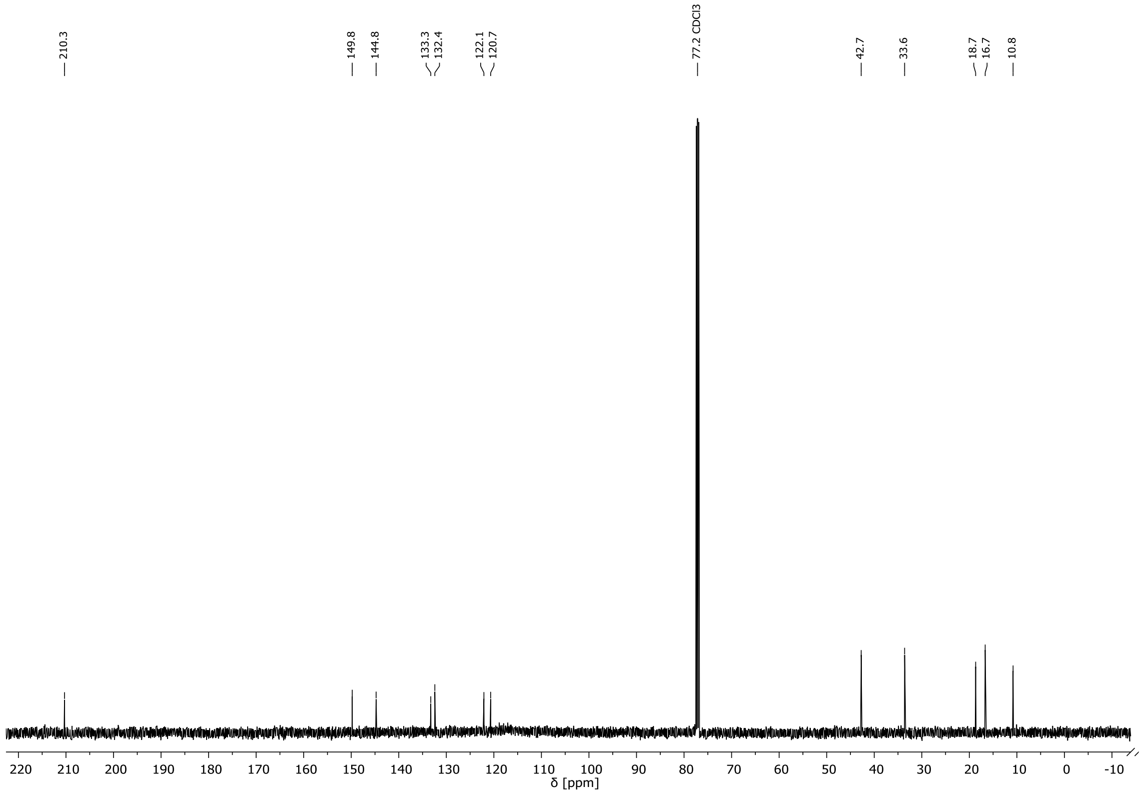

¹³C NMR Spectrum (126 MHz, 25 °C) of **S6** in $CDCl_3$.

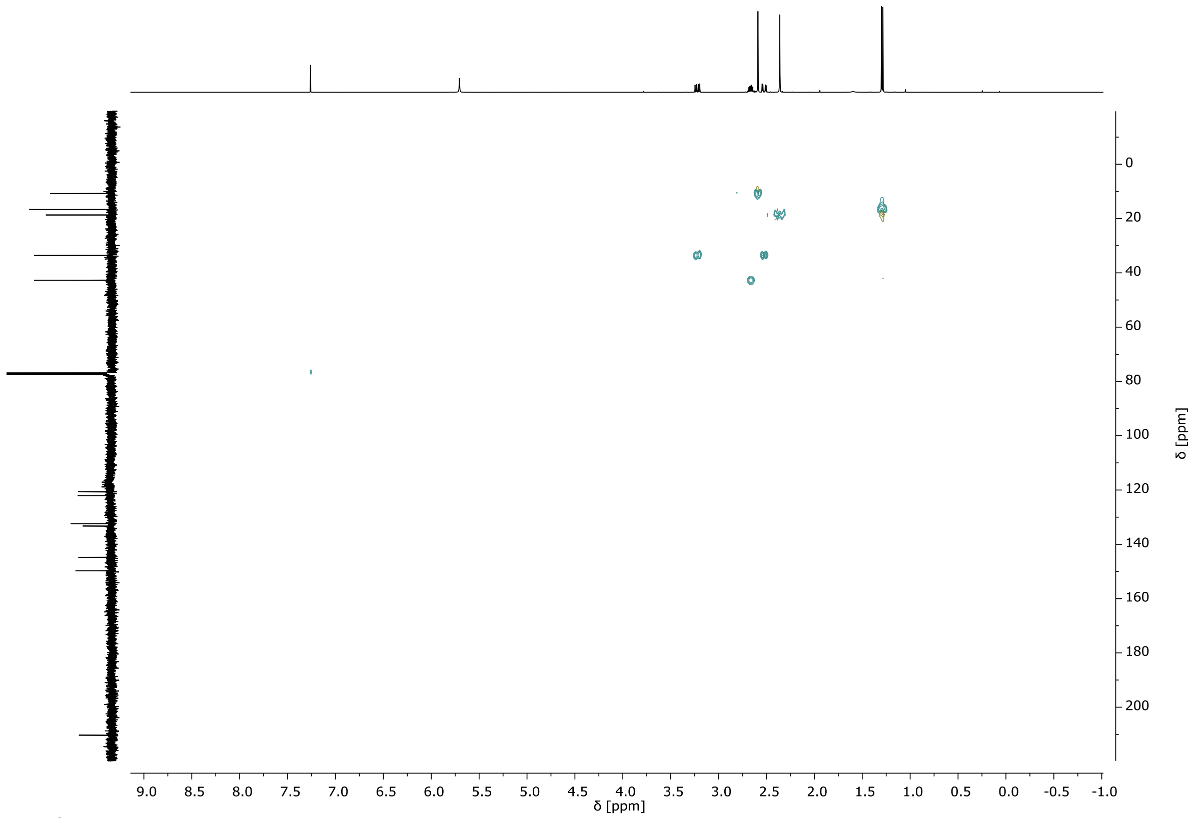

^{1}H, ^{13}C HSQC NMR Spectrum (500 MHz, 25 °C) of **S6** in $CDCl_3$.

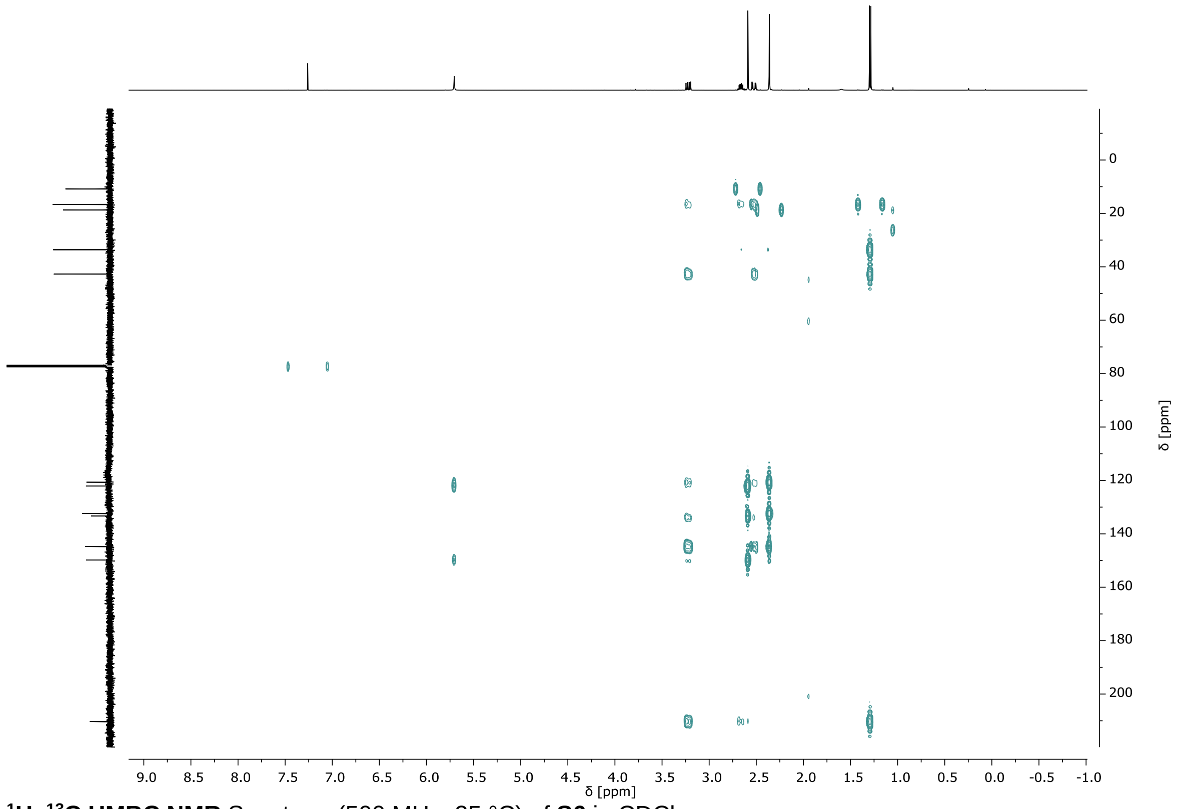

^{1}H, ^{13}C HMBC NMR Spectrum (500 MHz, 25 °C) of **S6** in $CDCl_3$.

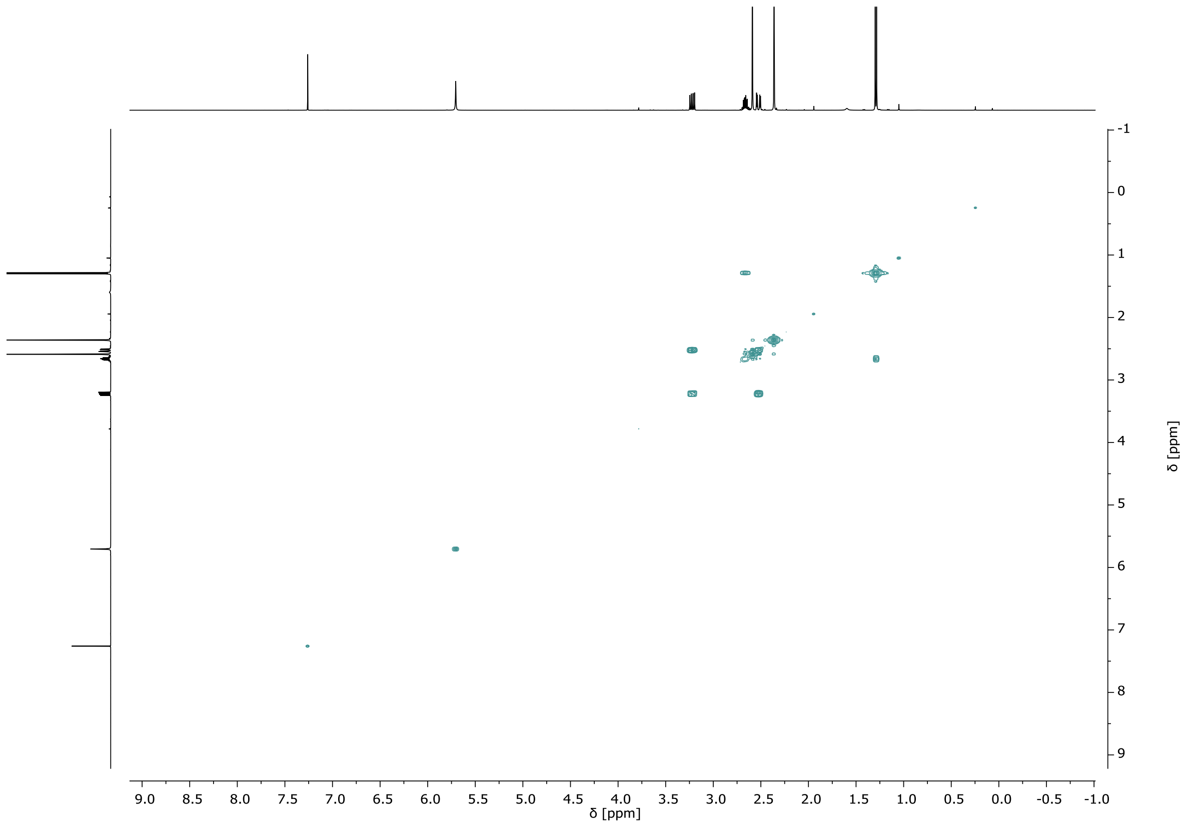

^{1}**H COSY** Spectrum (500 MHz, 25 °C) of **S6** in $CDCl_3$.

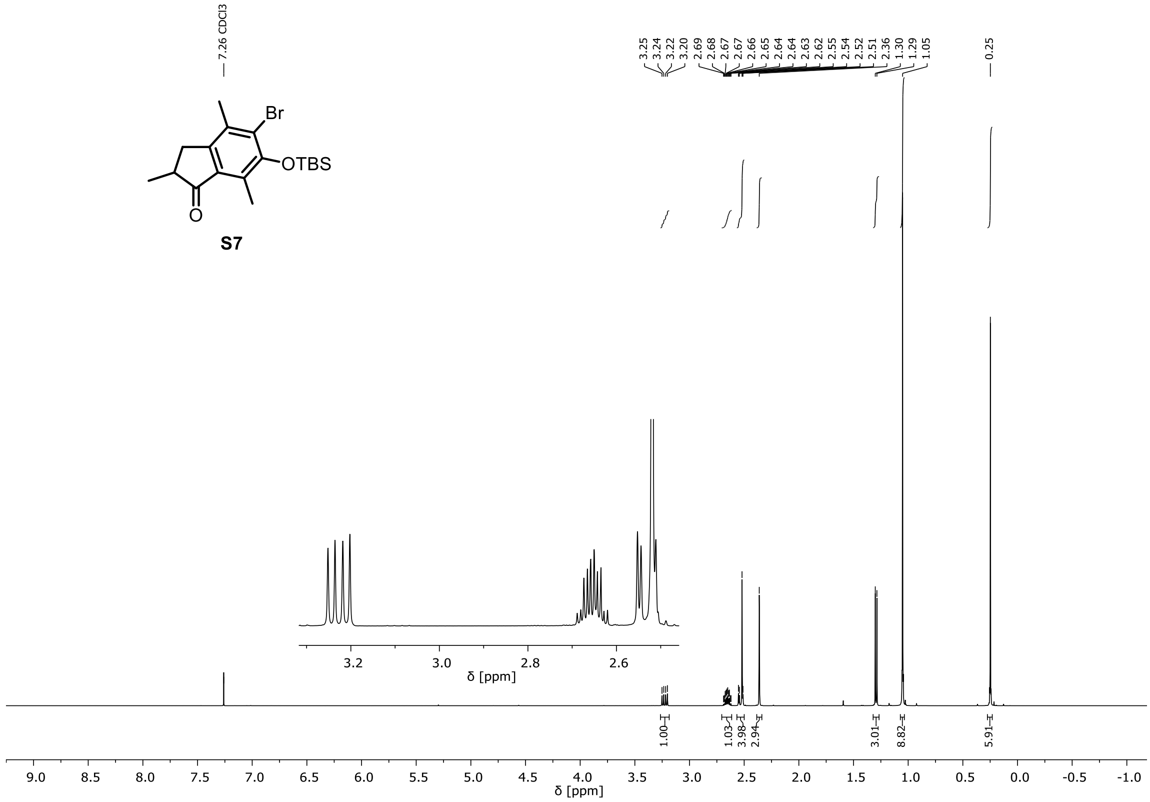

S7

[1]**H NMR** Spectrum (500 MHz, 25 °C) of **S7** in $CDCl_3$.

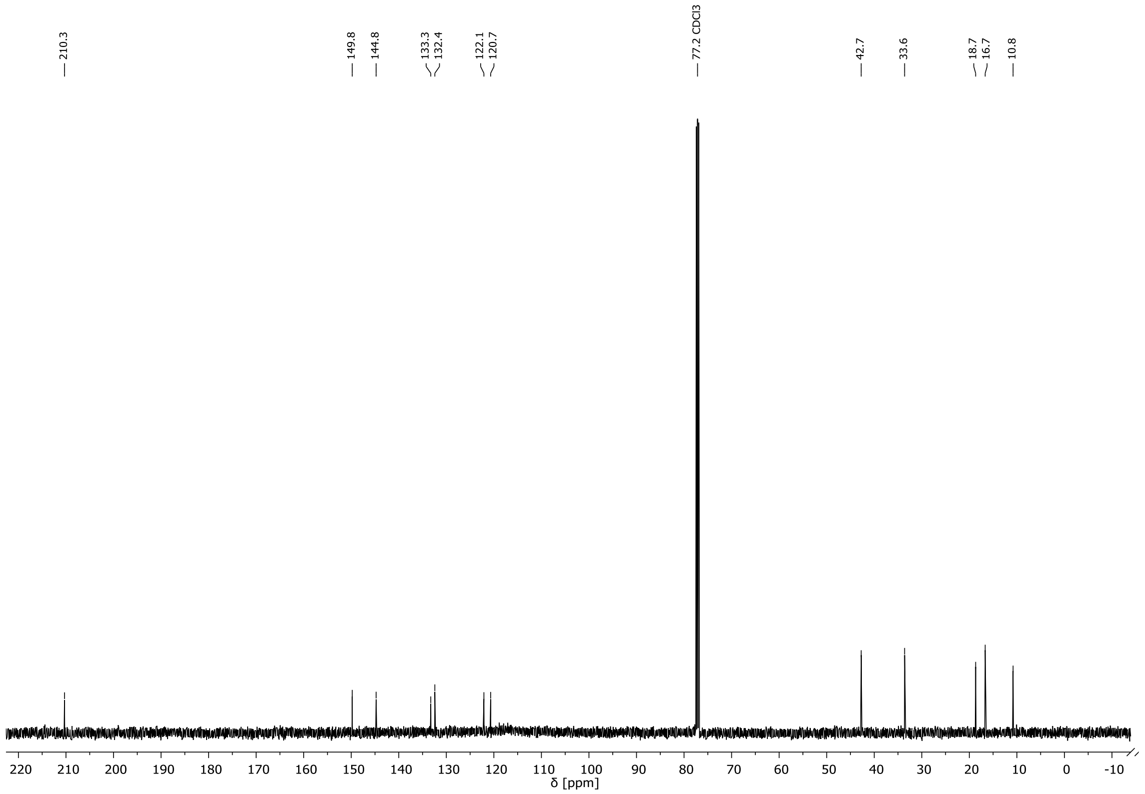

[13]**C NMR** Spectrum (126 MHz, 25 °C) of **S7** in $CDCl_3$.

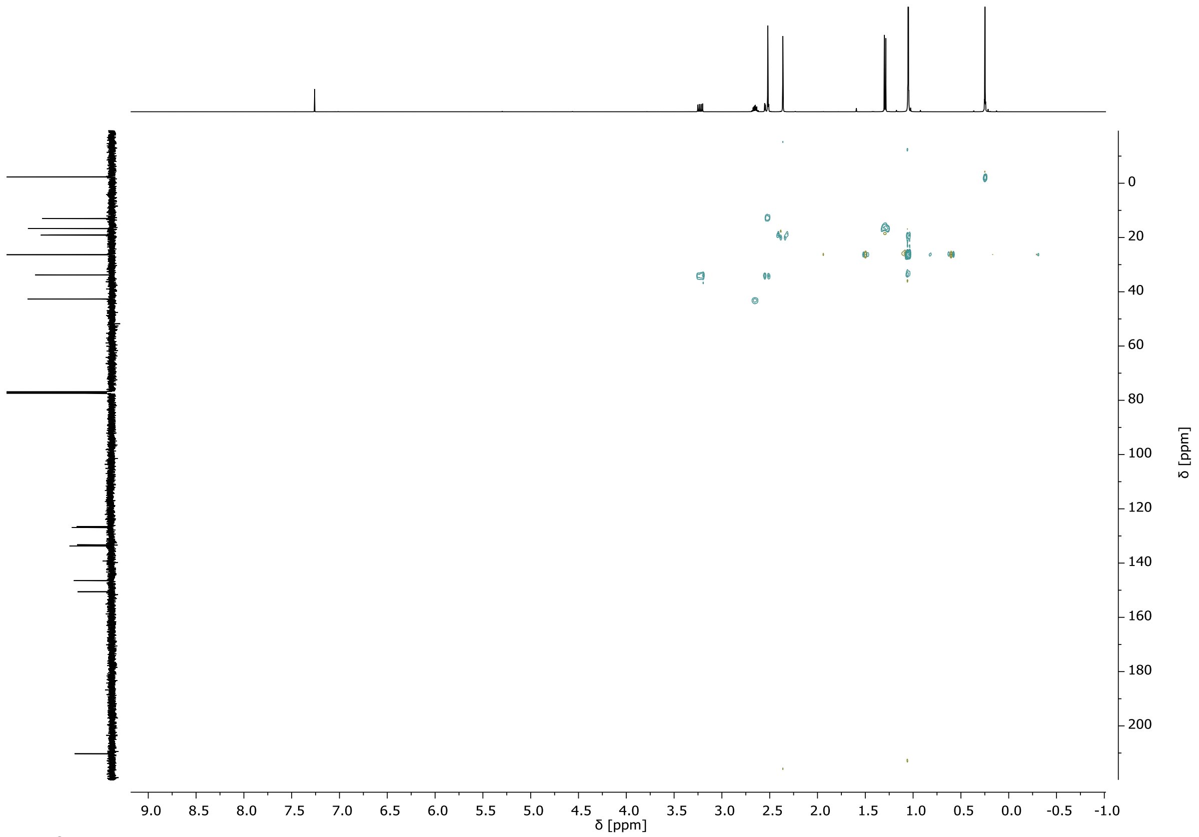

^{1}H, ^{13}C **HSQC NMR** Spectrum (500 MHz, 25 °C) of **S7** in $CDCl_3$.

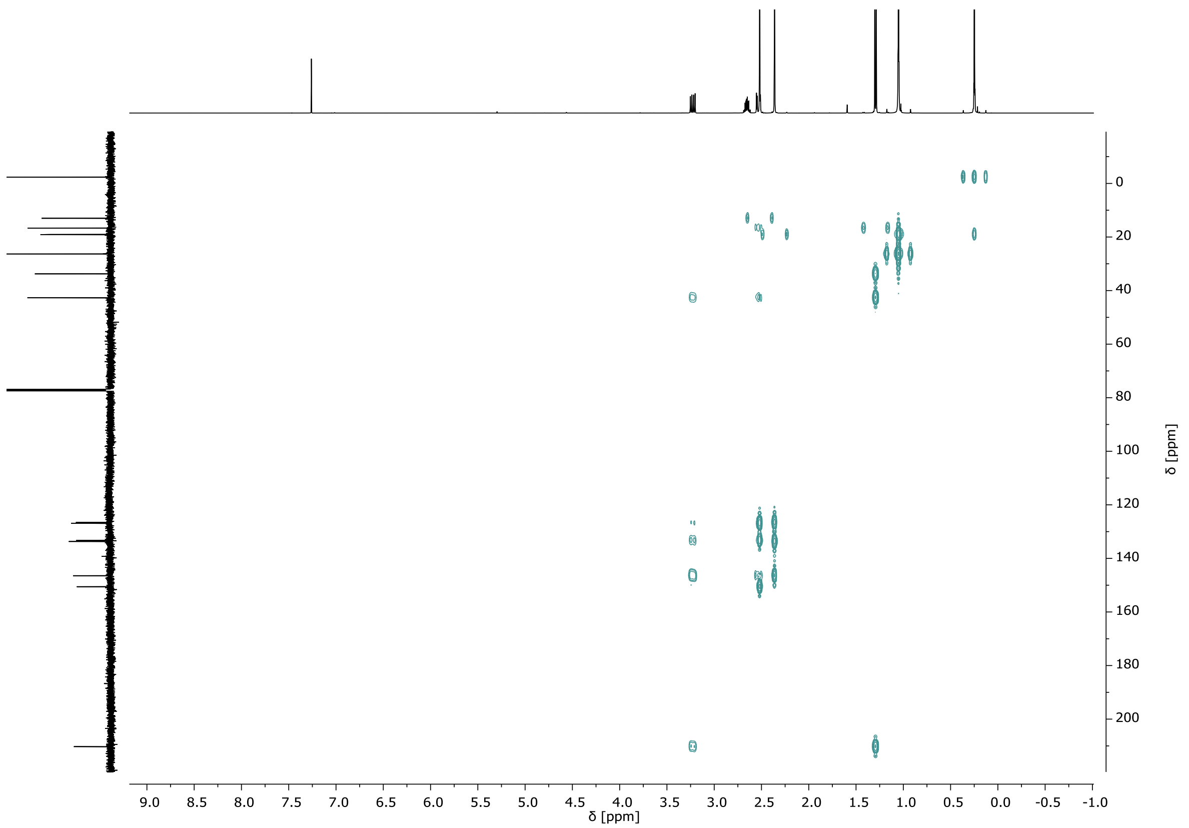

^{1}H, ^{13}C **HMBC NMR** Spectrum (500 MHz, 25 °C) of **S7** in $CDCl_3$.

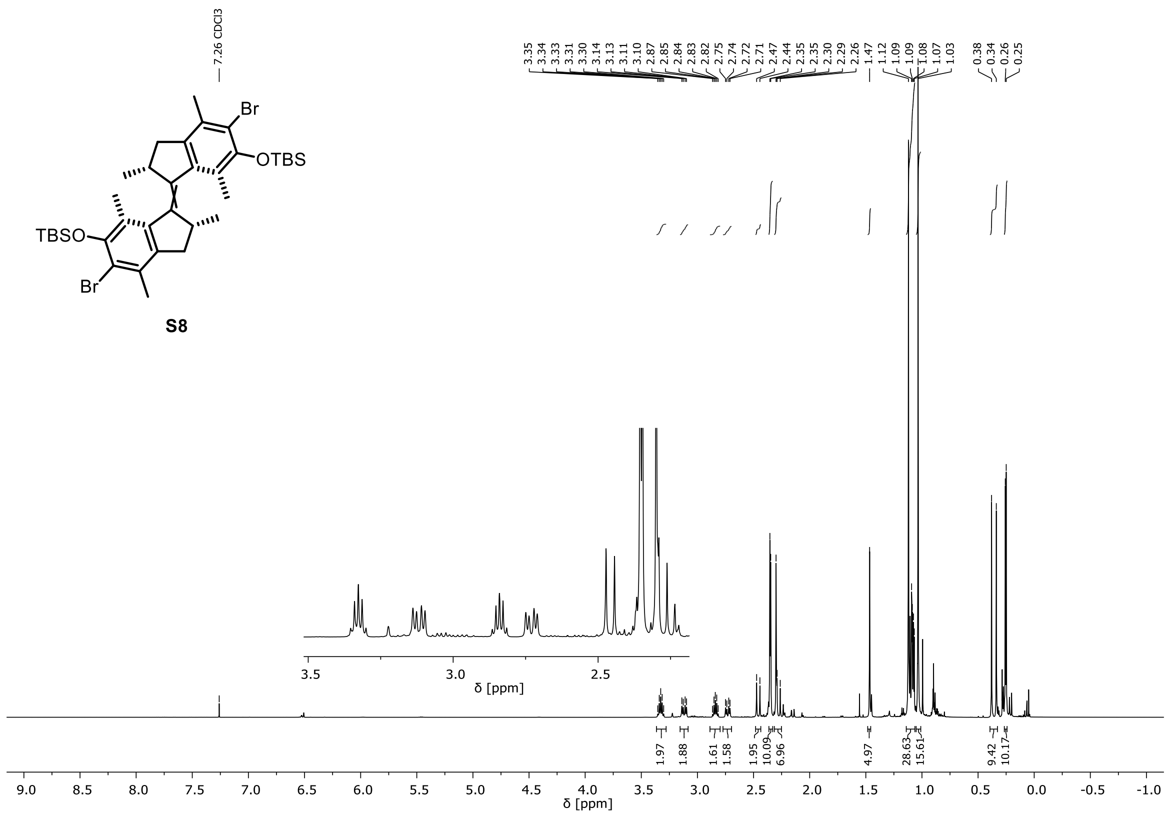

^{1}H NMR Spectrum (500 MHz, 25 °C) of **S8** in $CDCl_3$.

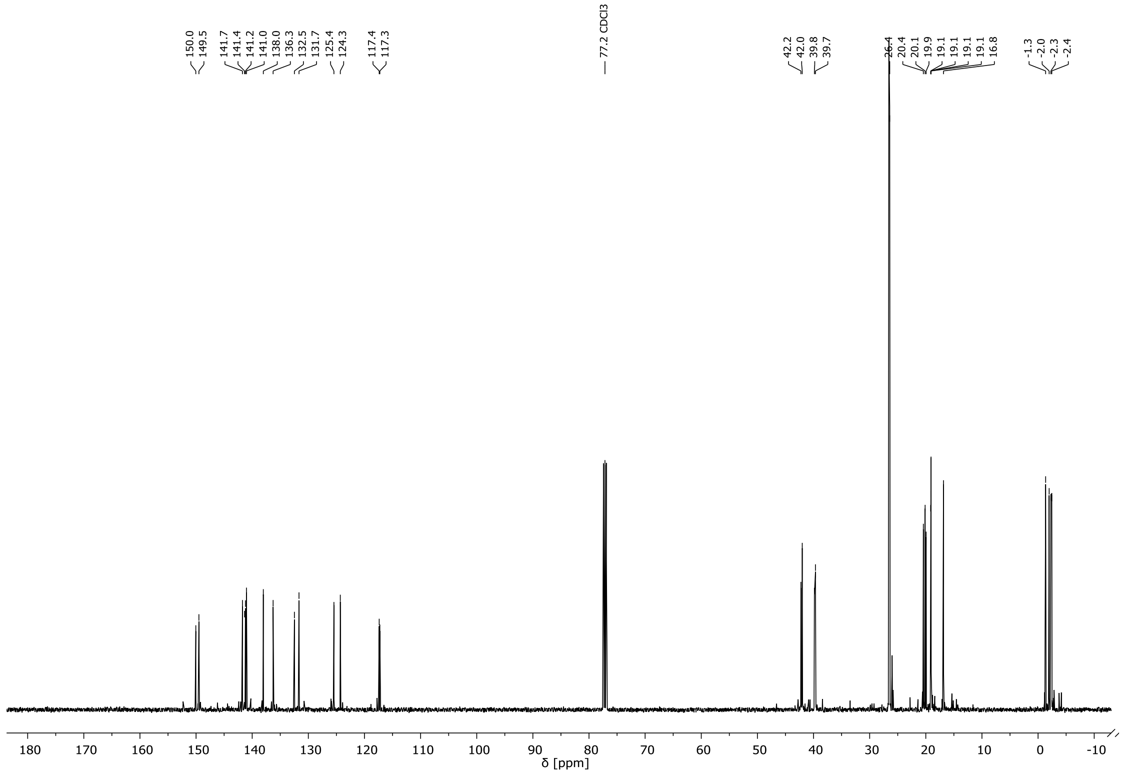

^{13}C NMR Spectrum (126 MHz, 25 °C) of **S8** in $CDCl_3$.

S9

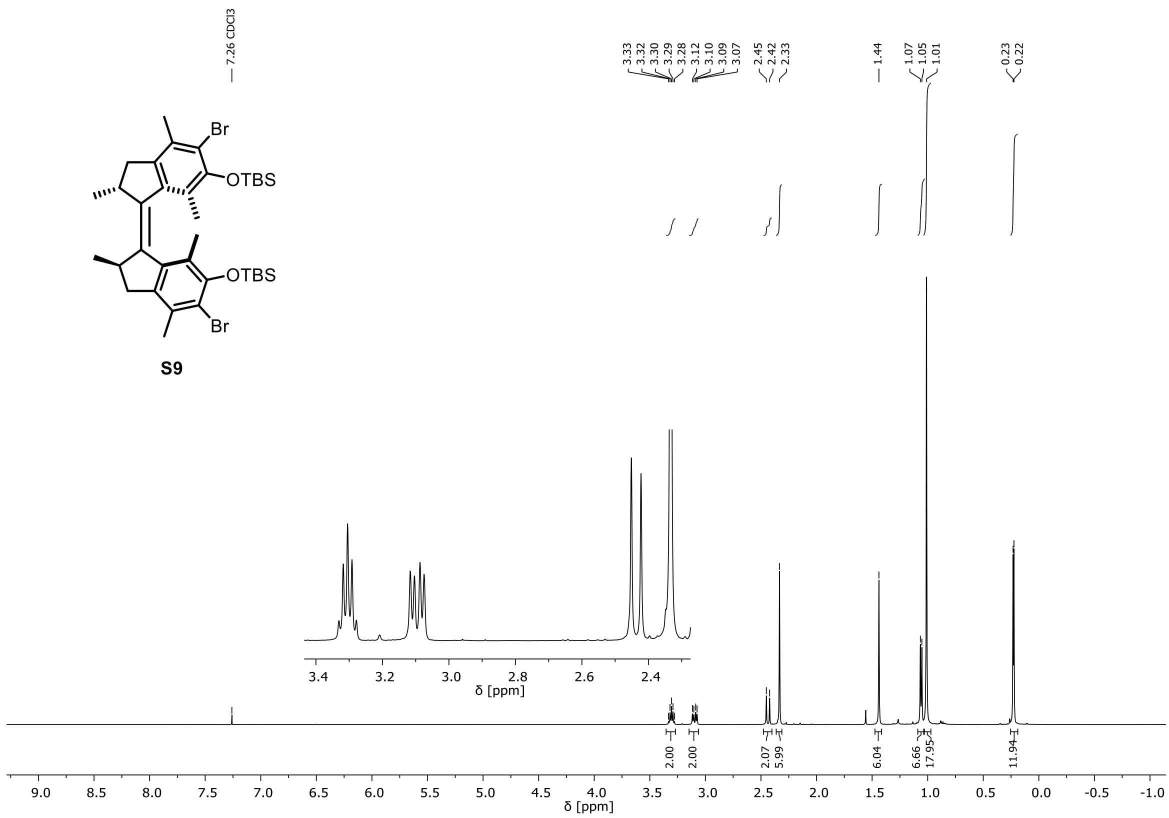

¹H NMR Spectrum (500 MHz, 25 °C) of **S9** in $CDCl_3$.

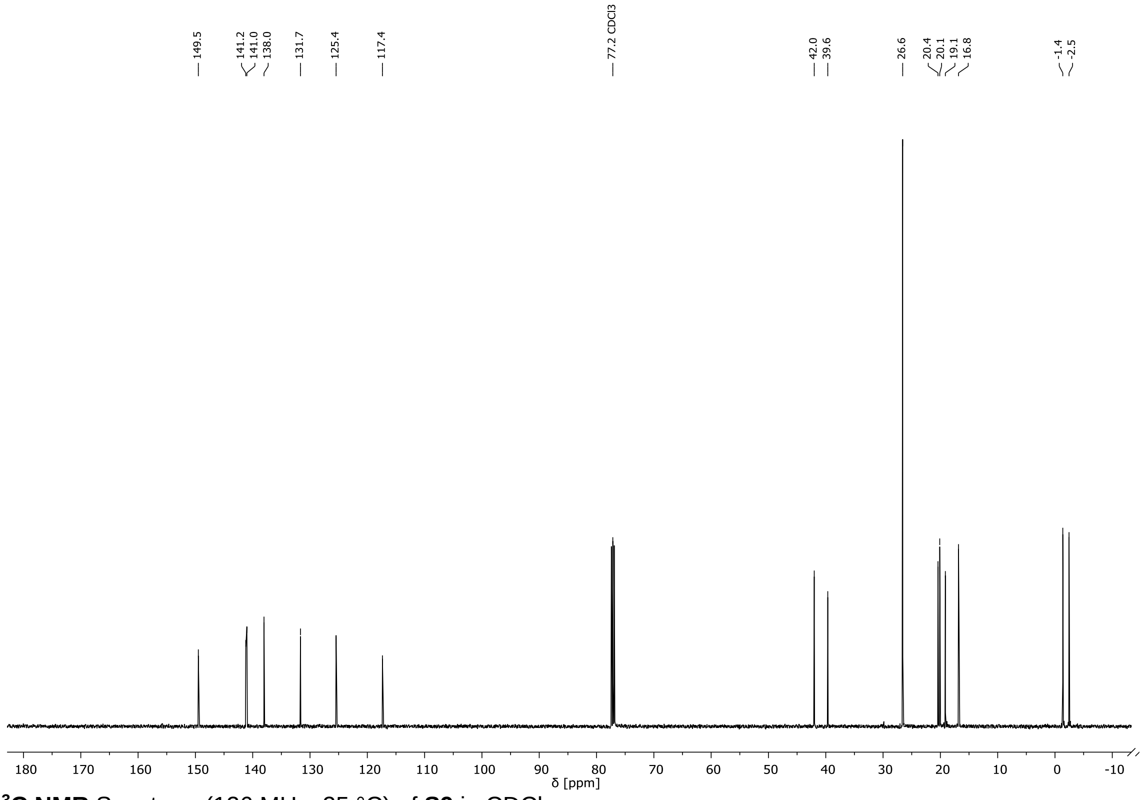

¹³C NMR Spectrum (126 MHz, 25 °C) of **S9** in $CDCl_3$.

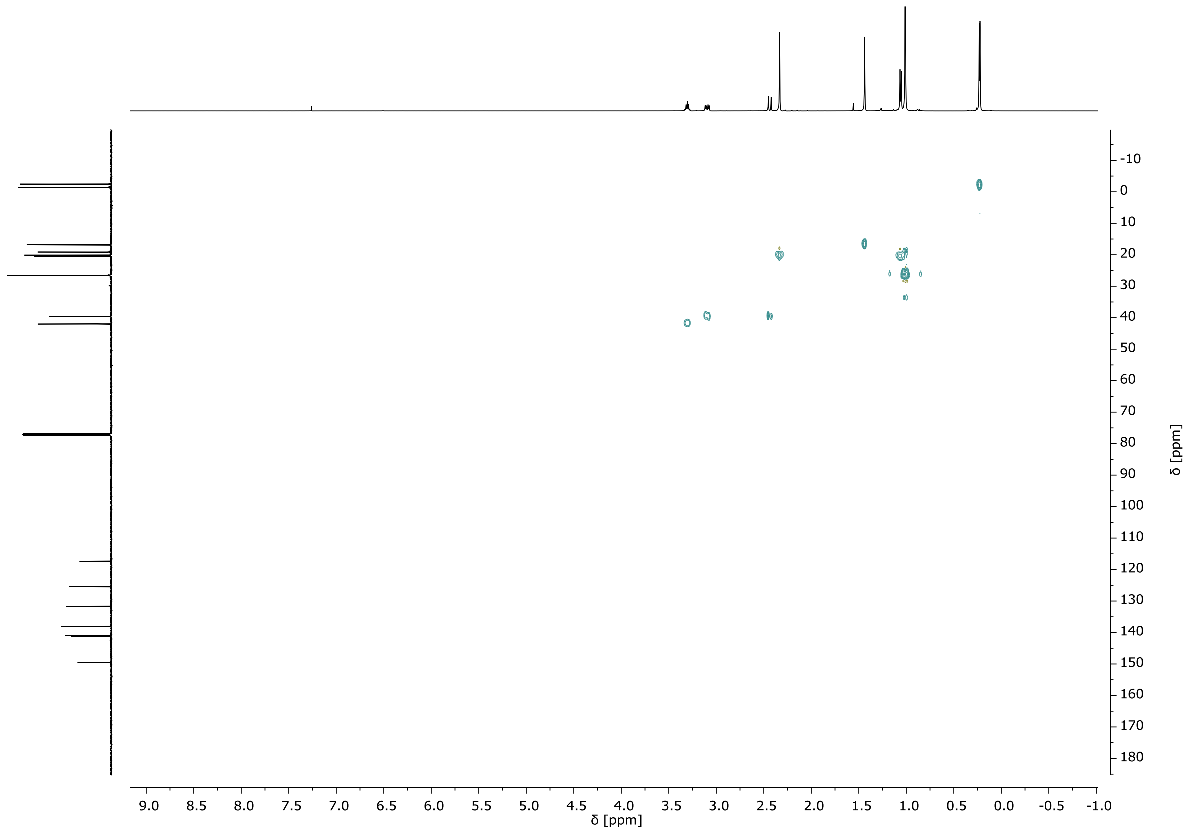

^{1}H, ^{13}C HSQC NMR Spectrum (500 MHz, 25 °C) of **S9** in $CDCl_3$.

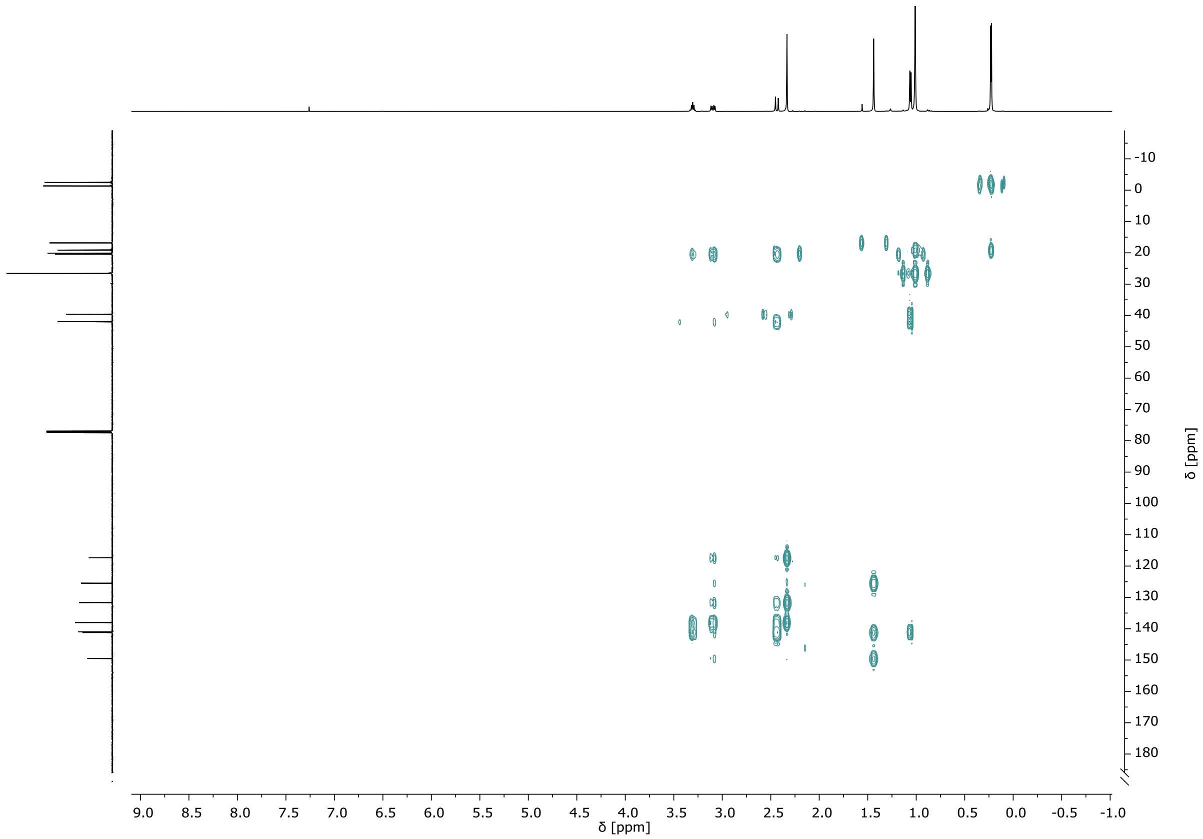

^{1}H, ^{13}C HMBC NMR Spectrum (500 MHz, 25 °C) of **S9** in $CDCl_3$.

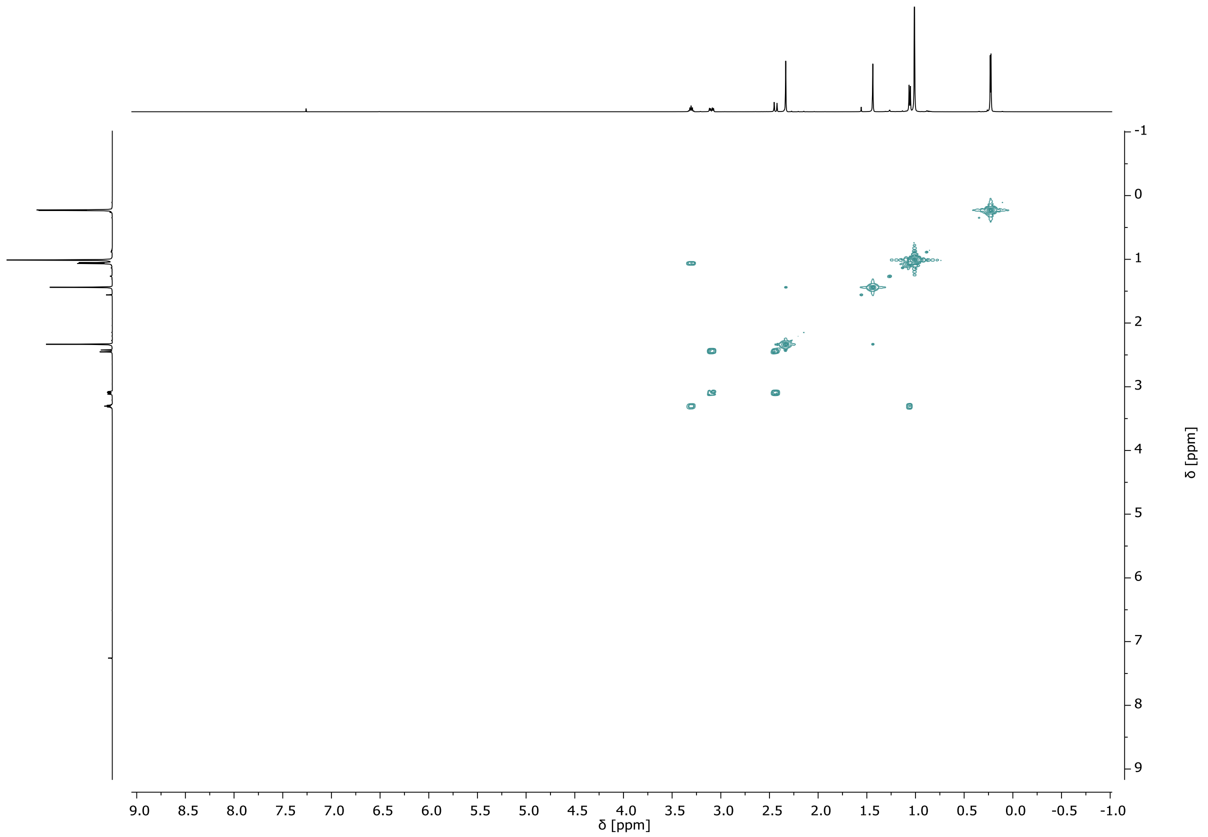

^{1}H COSY NMR Spectrum (500 MHz, 25 °C) of **S9** in $CDCl_3$.

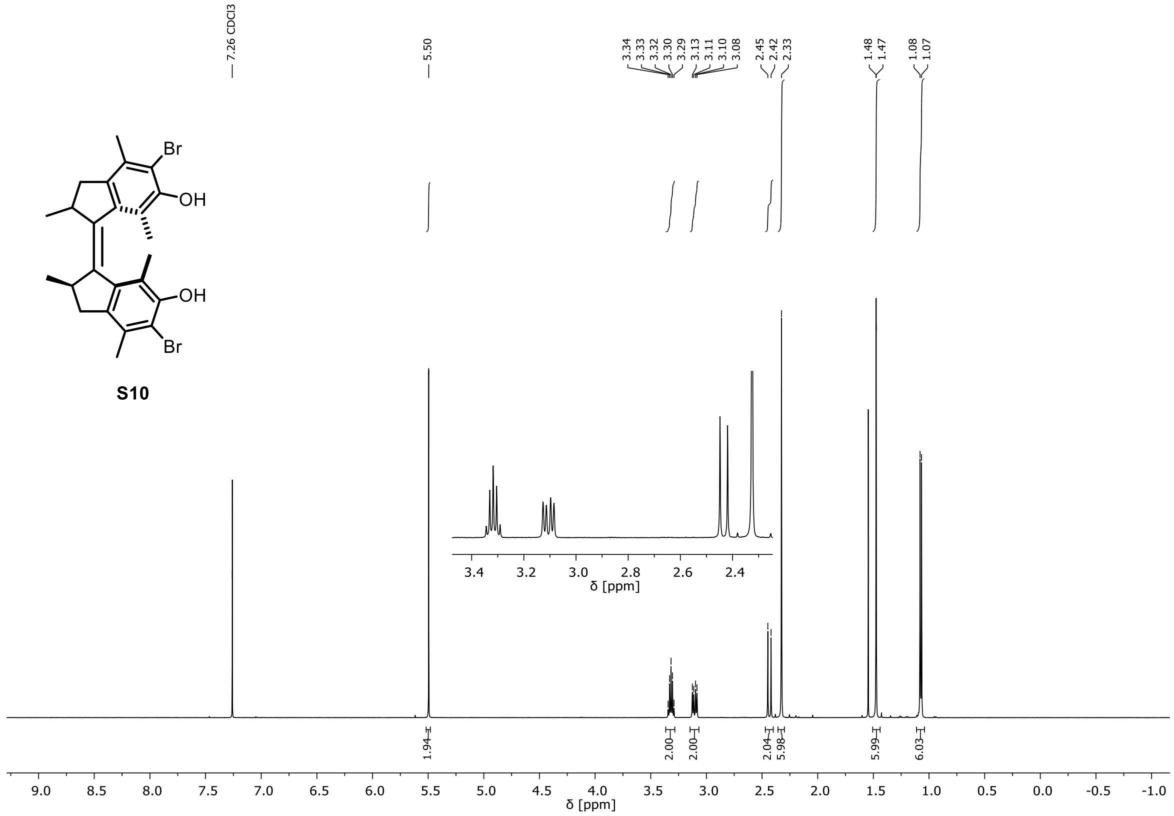

^{1}H NMR Spectrum (500 MHz, 25 °C) of **S10** in $CDCl_3$.

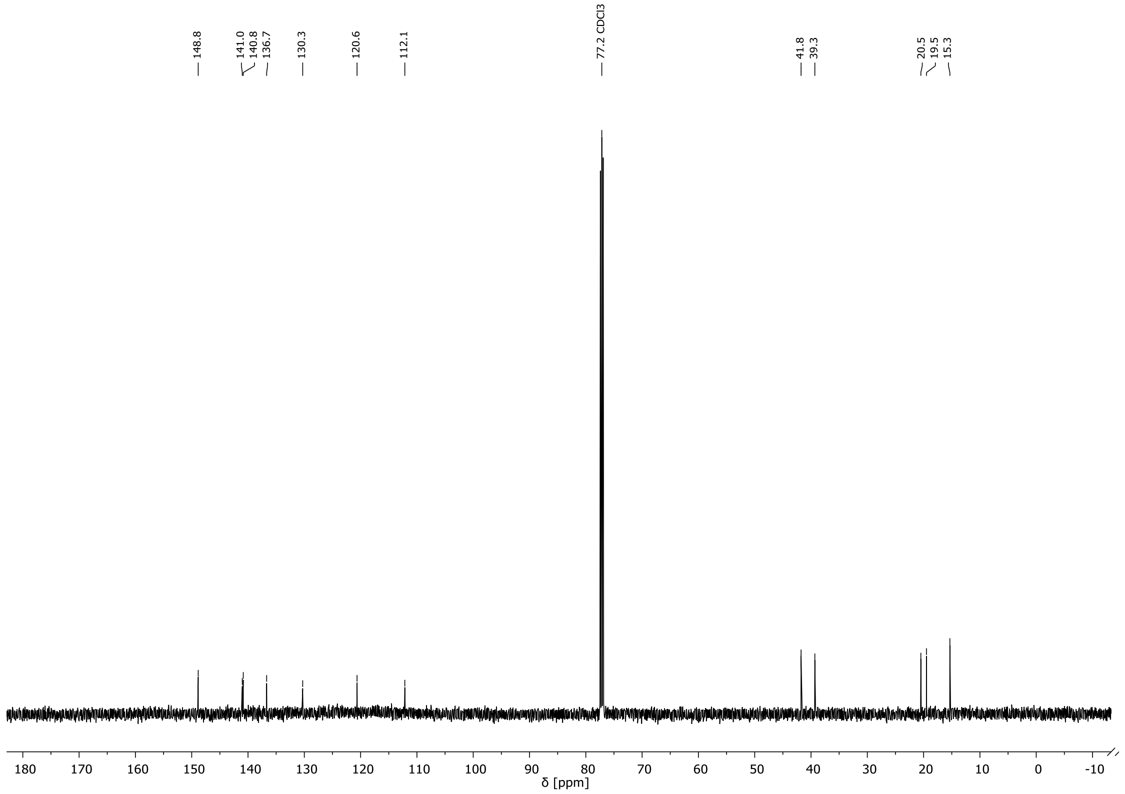

^{13}C NMR Spectrum (126 MHz, 25 °C) of **S10** in $CDCl_3$.

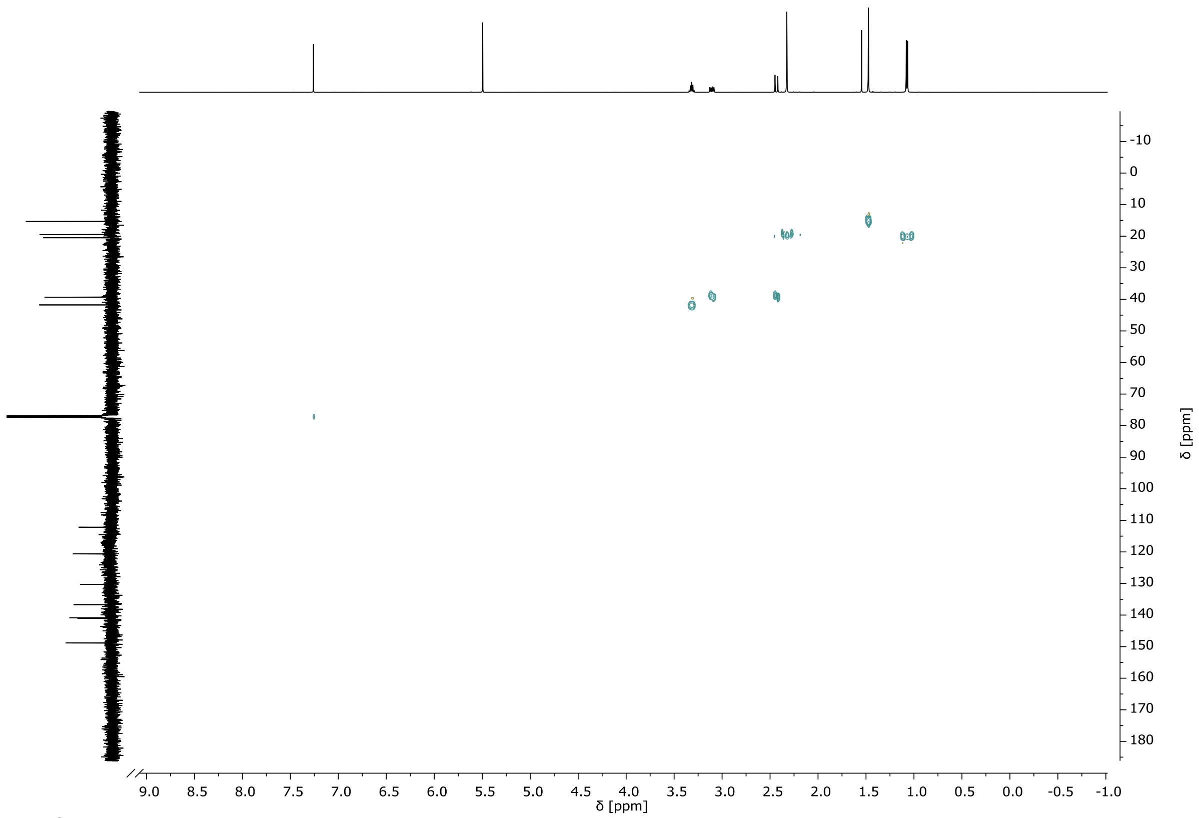

¹H, ¹³C HSQC NMR Spectrum (500 MHz, 25 °C) of **S10** in $CDCl_3$.

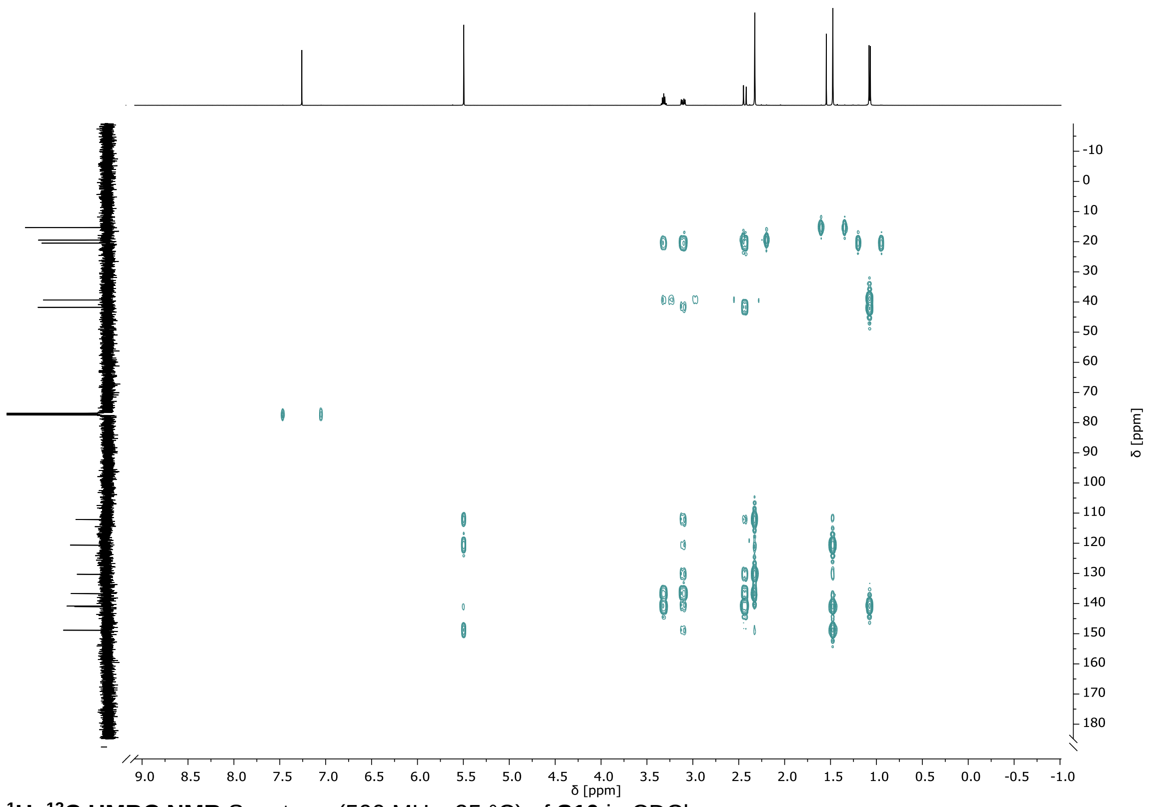

¹H, ¹³C HMBC NMR Spectrum (500 MHz, 25 °C) of **S10** in $CDCl_3$.

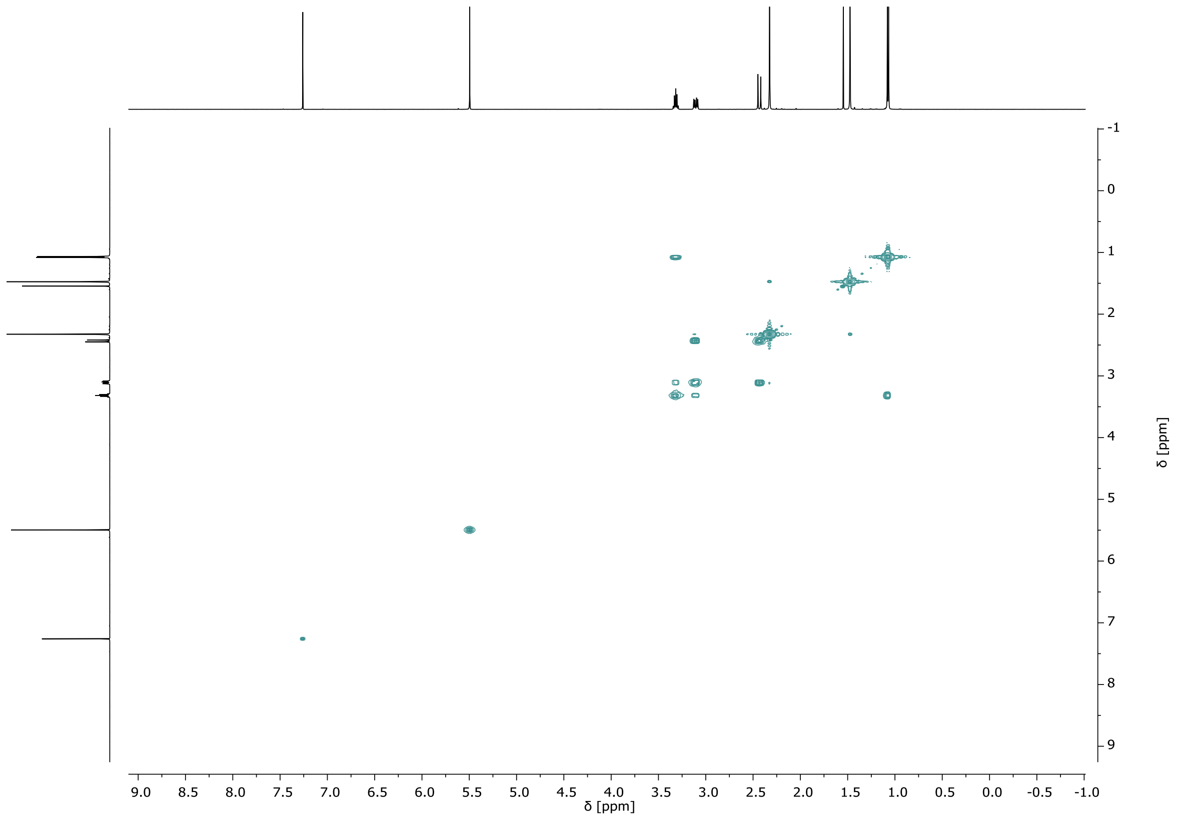

^{1}H COSY NMR Spectrum (500 MHz, 25 °C) of **S10** in $CDCl_3$.

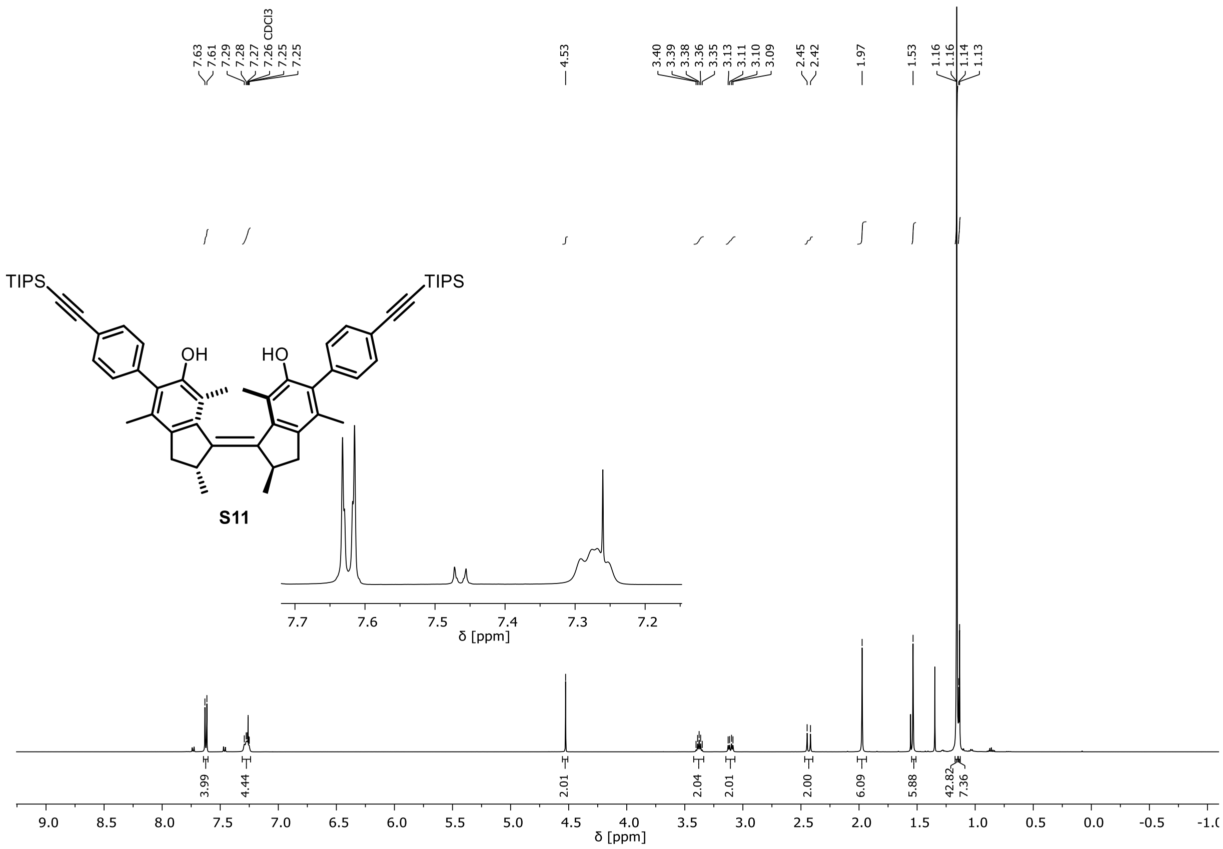

^{1}H NMR Spectrum (500 MHz, 25 °C) of **S11** in $CDCl_3$.

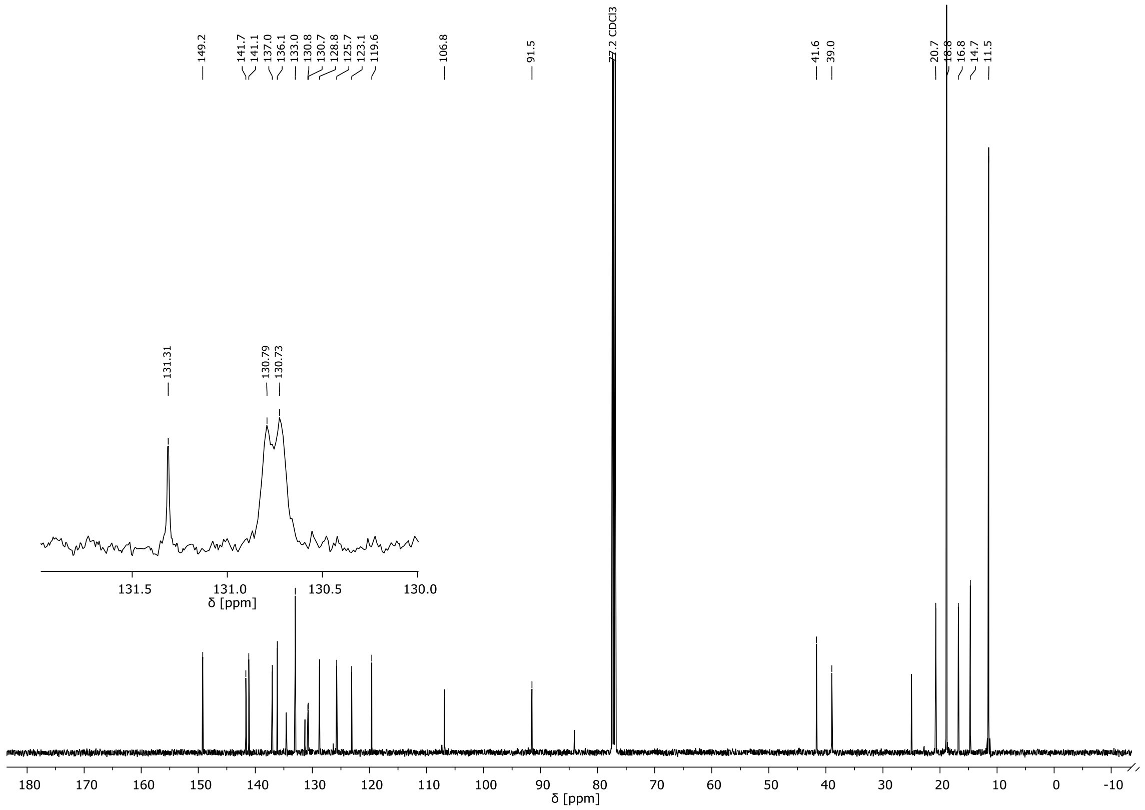

^{13}C NMR Spectrum (126 MHz, 25 °C) of **S11** in $CDCl_3$.

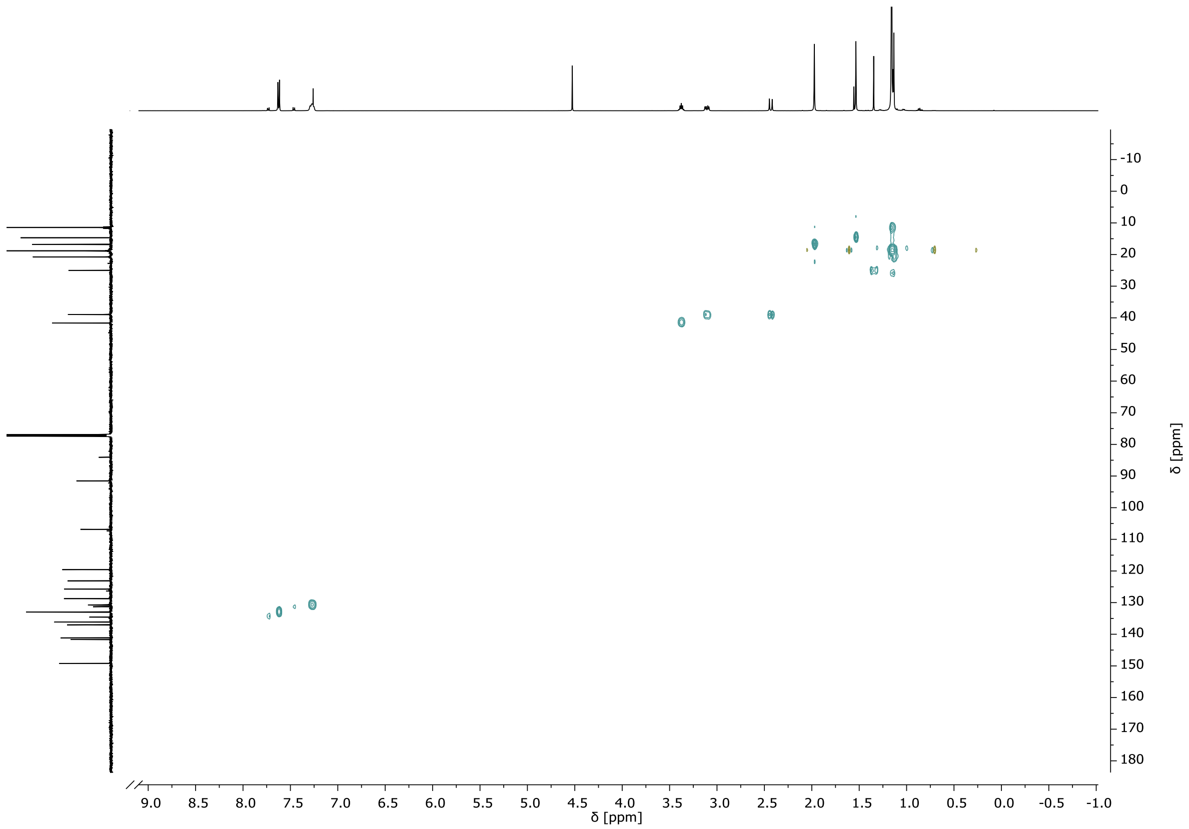

1H, 13C HSQC NMR Spectrum (500 MHz, 25 °C) of **S11** in $CDCl_3$.

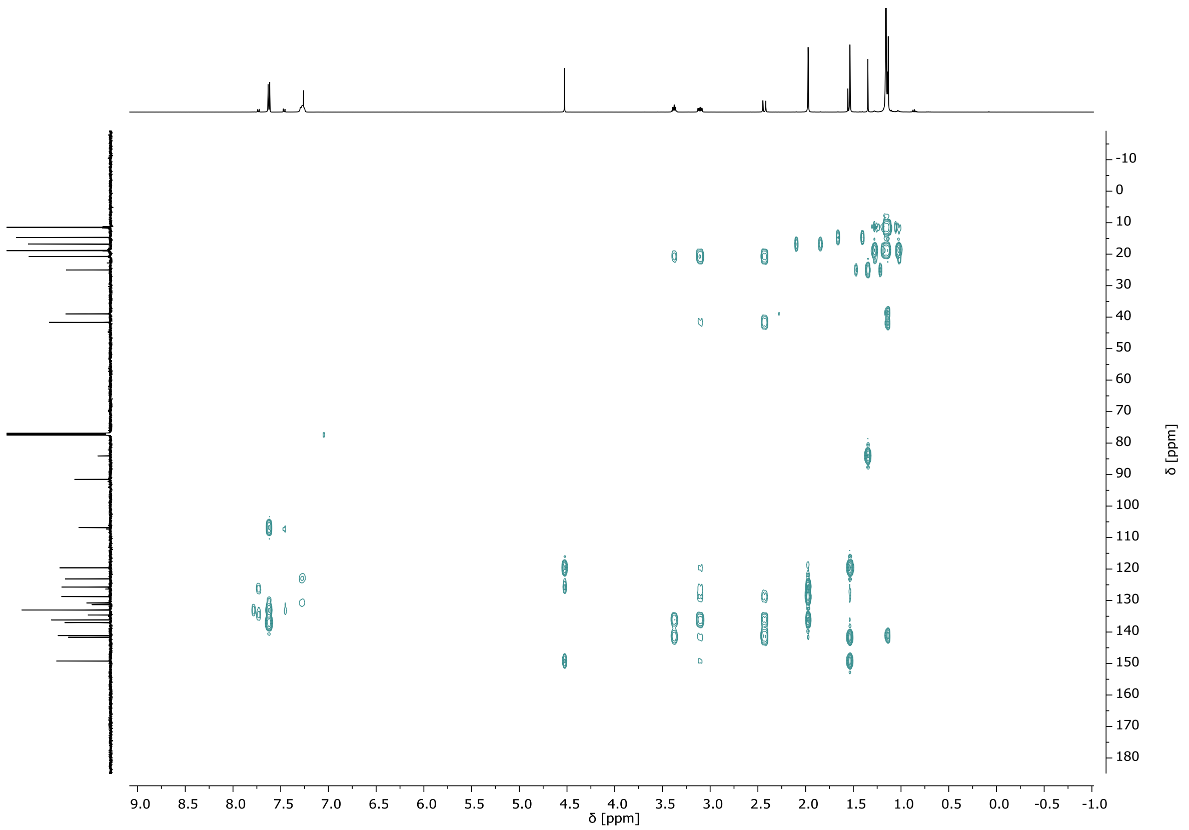

1H, 13C HMBC NMR Spectrum (500 MHz, 25 °C) of **S11** in $CDCl_3$.

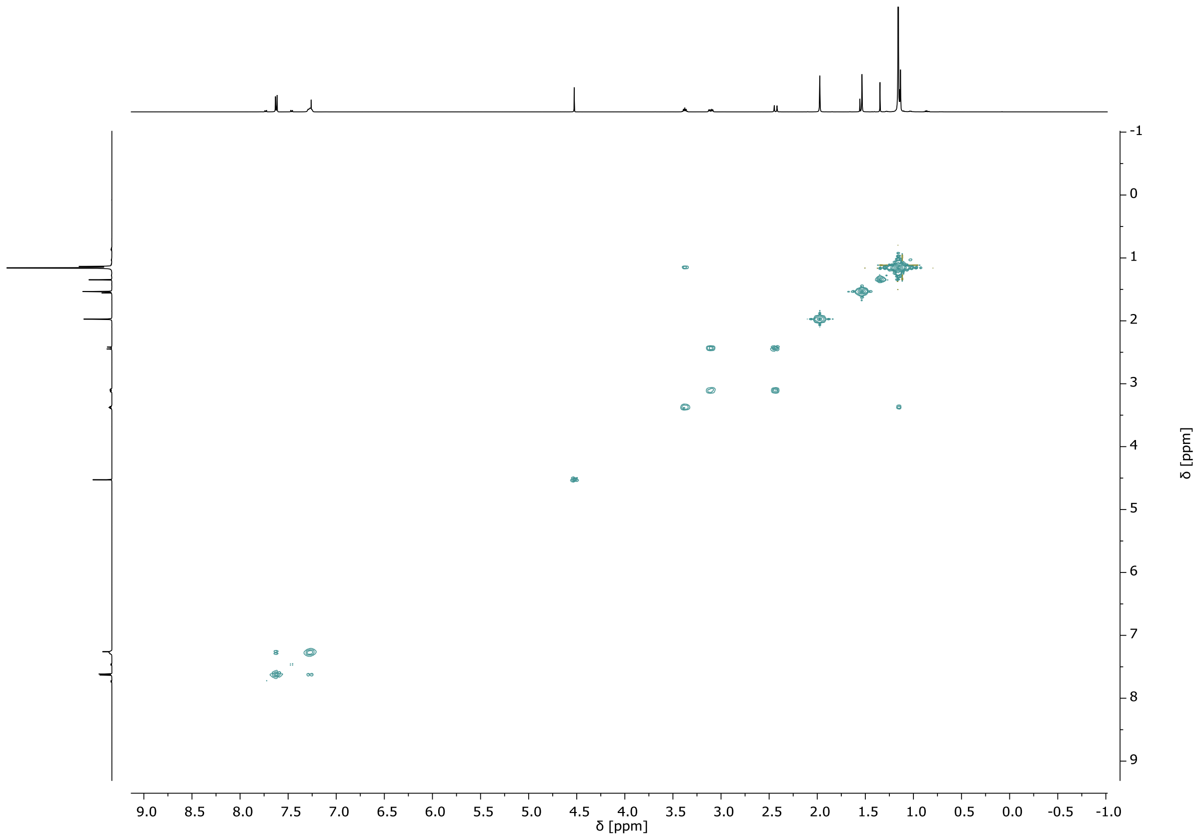

^{1}H COSY NMR Spectrum (500 MHz, 25 °C) of **S11** in $CDCl_3$.

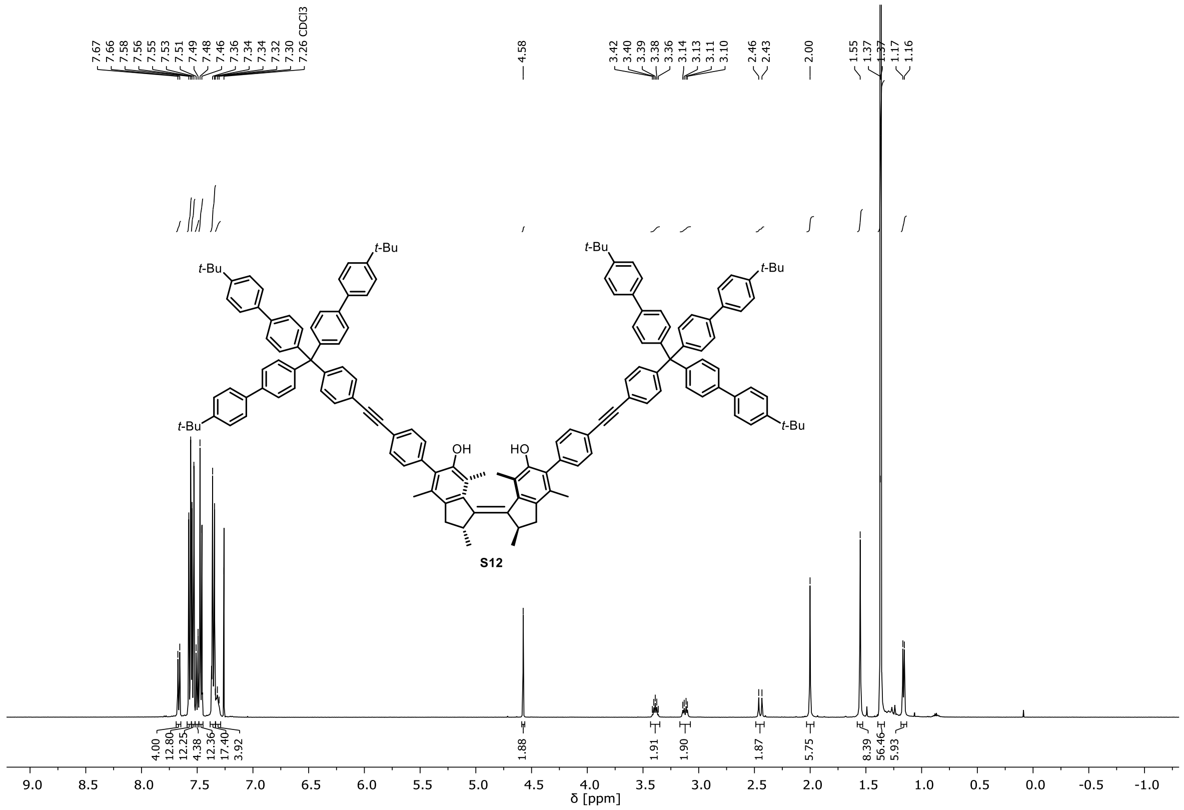

^{1}H NMR Spectrum (500 MHz, 25 °C) of **S12** in $CDCl_3$.

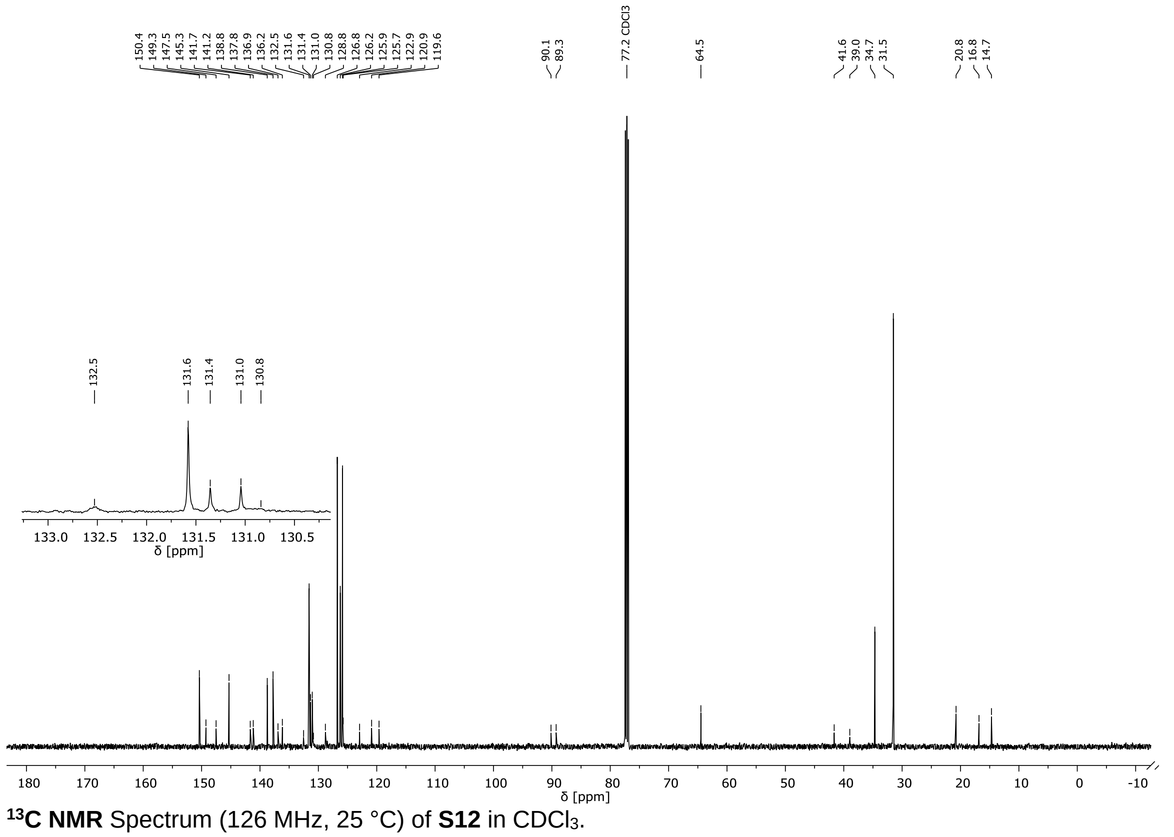

^{13}C NMR Spectrum (126 MHz, 25 °C) of **S12** in $CDCl_3$.

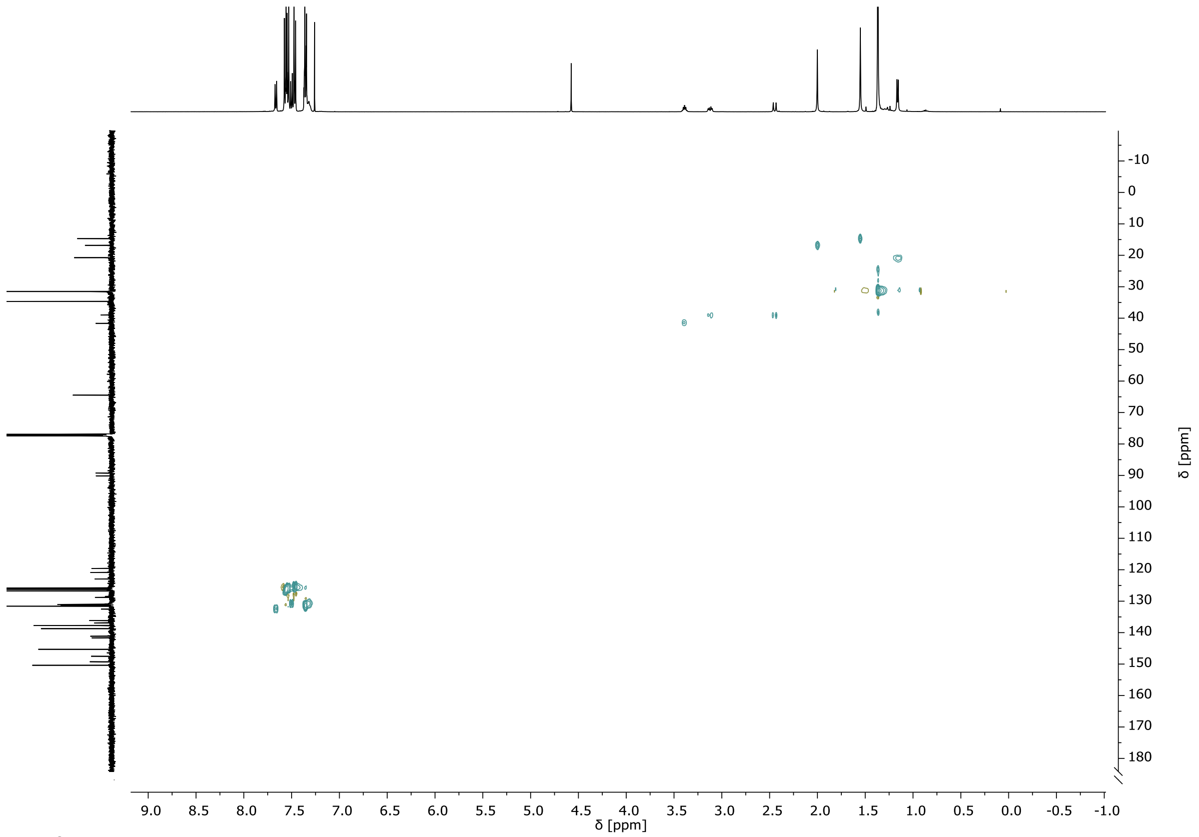

^{1}H, ^{13}C HSQC NMR Spectrum (500 MHz, 25 °C) of **S12** in $CDCl_3$.

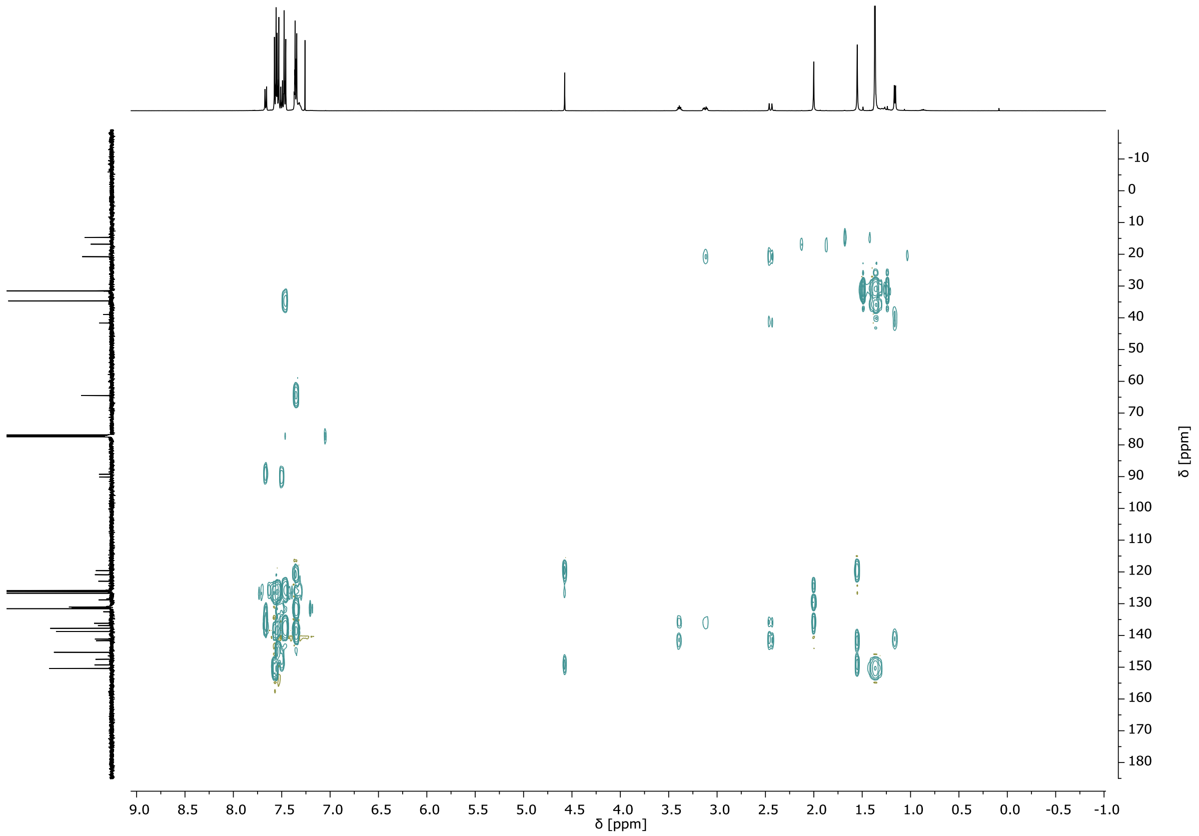

^{1}H, ^{13}C HMBC NMR Spectrum (500 MHz, 25 °C) of **S12** in $CDCl_3$.

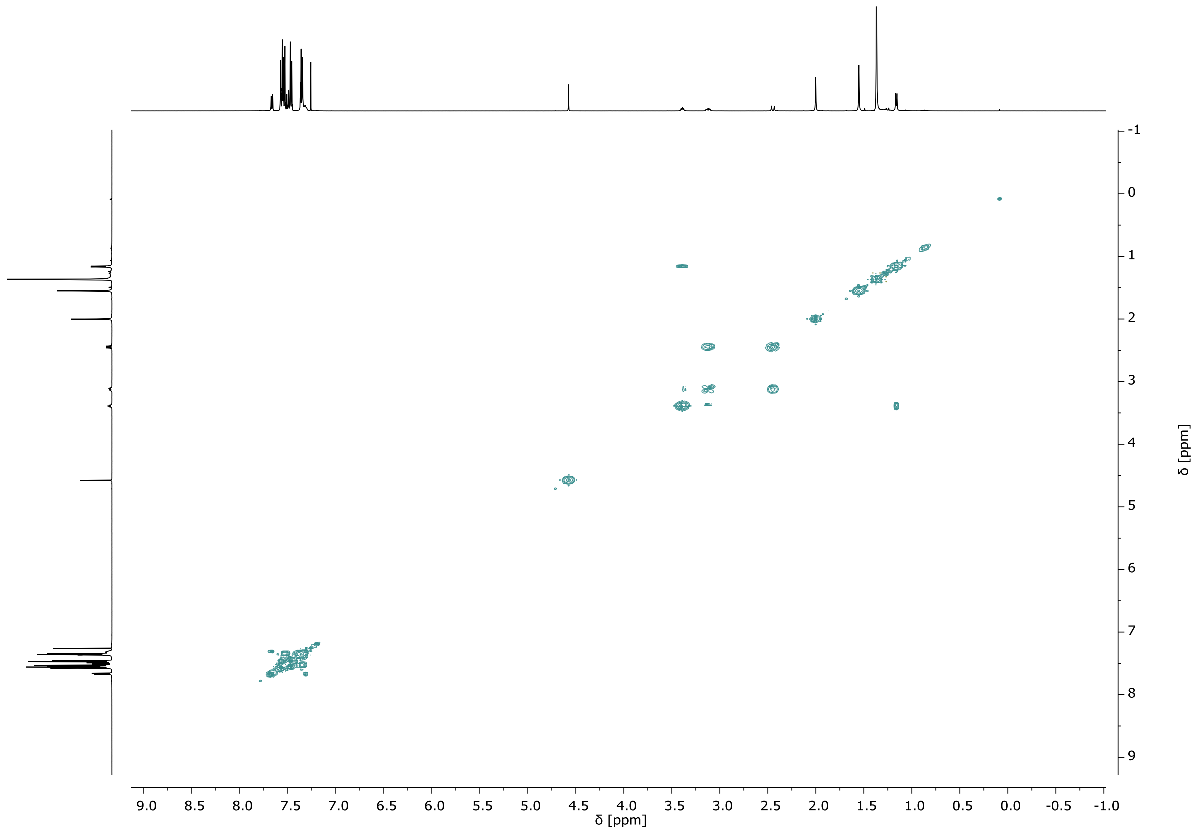

[1]**H COSY NMR** Spectrum (500 MHz, 25 °C) of **S12** in $CDCl_3$.

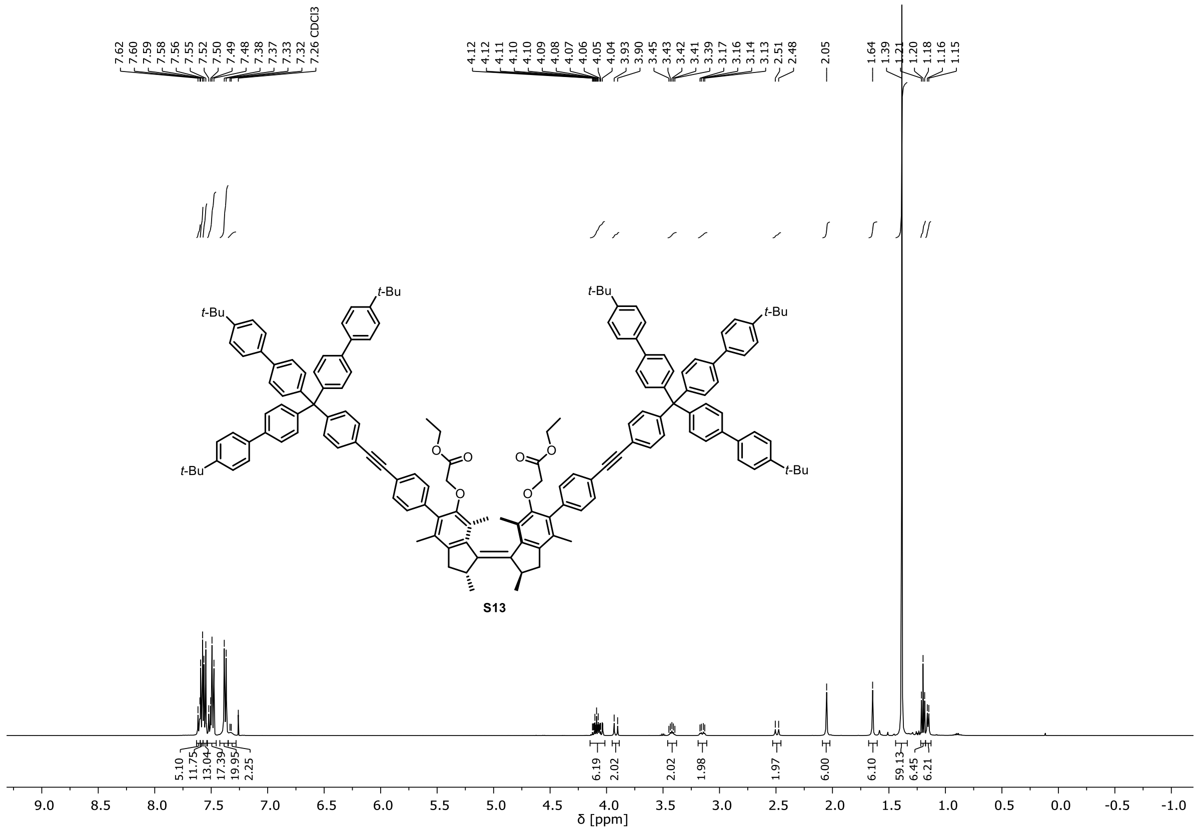

^{1}H NMR Spectrum (500 MHz, 25 °C) of **S13** in $CDCl_3$.

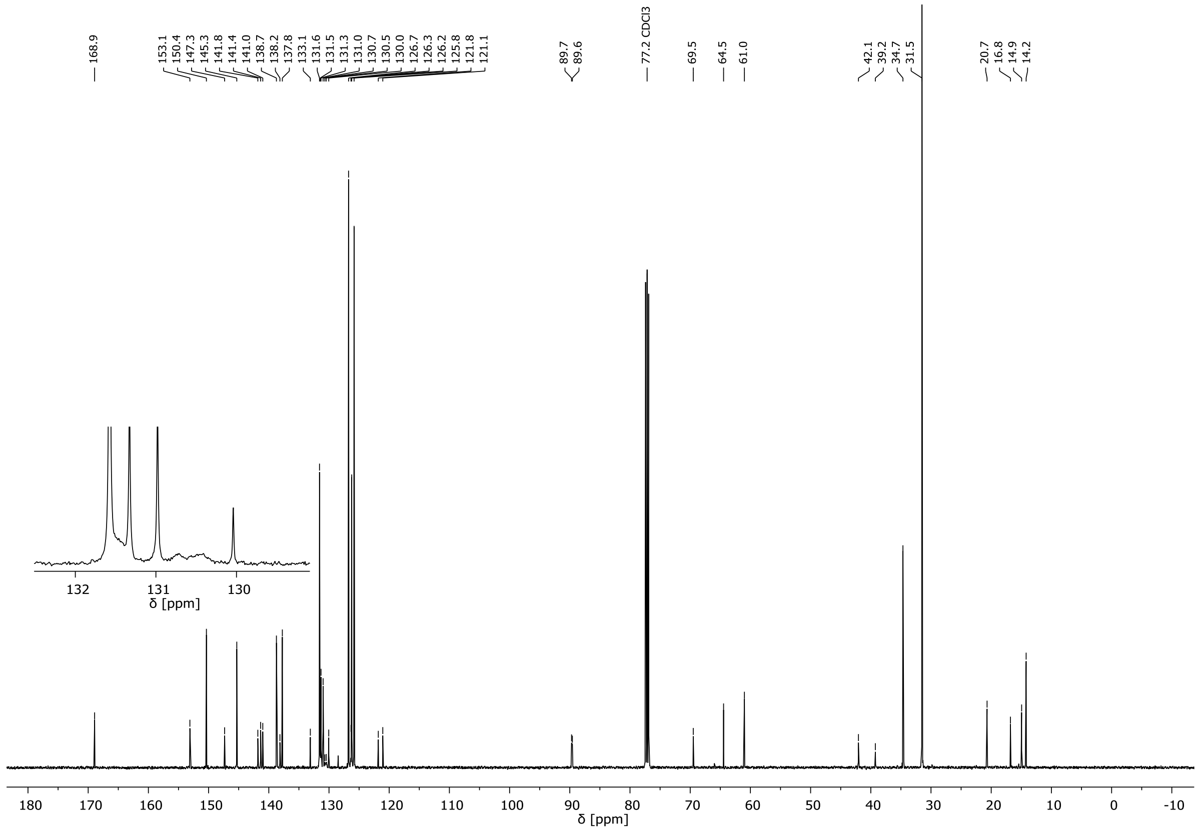

^{13}C NMR Spectrum (126 MHz, 25 °C) of **S13** in $CDCl_3$.

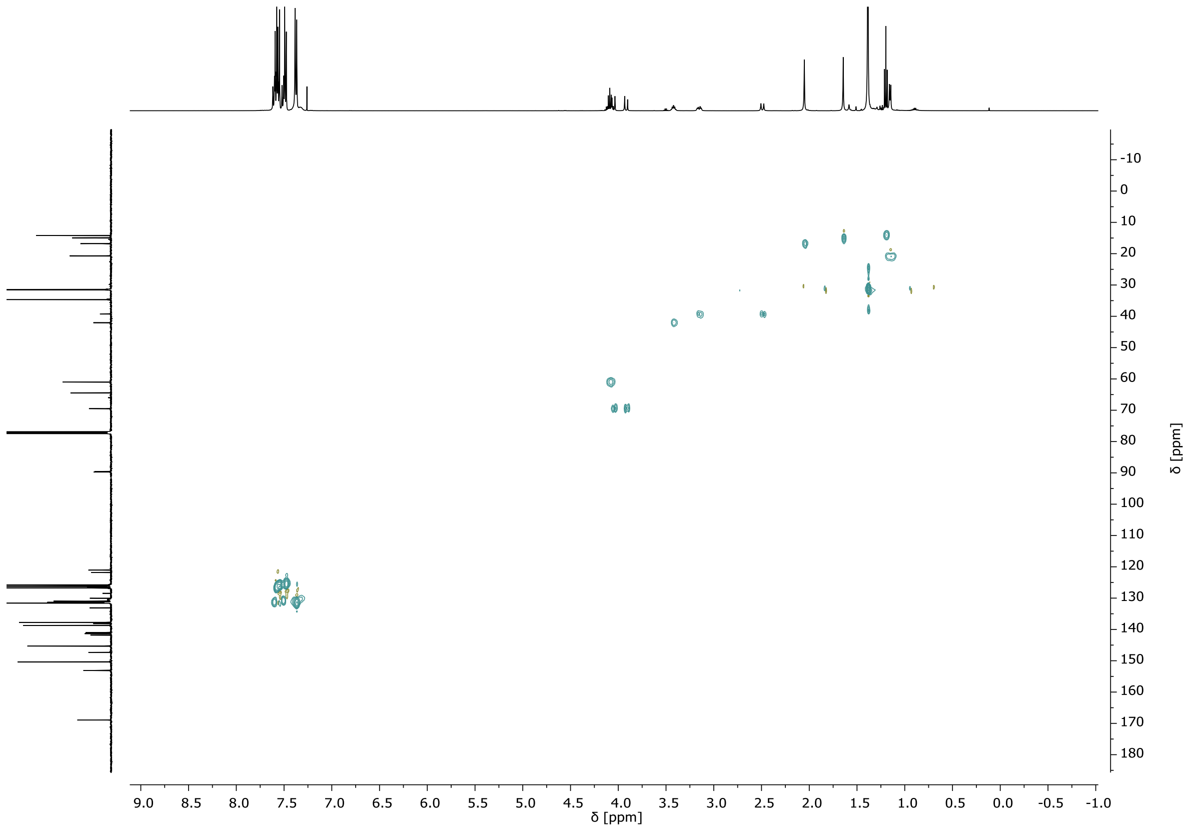

¹H, ¹³C HSQC NMR Spectrum (500 MHz, 25 °C) of **S13** in $CDCl_3$.

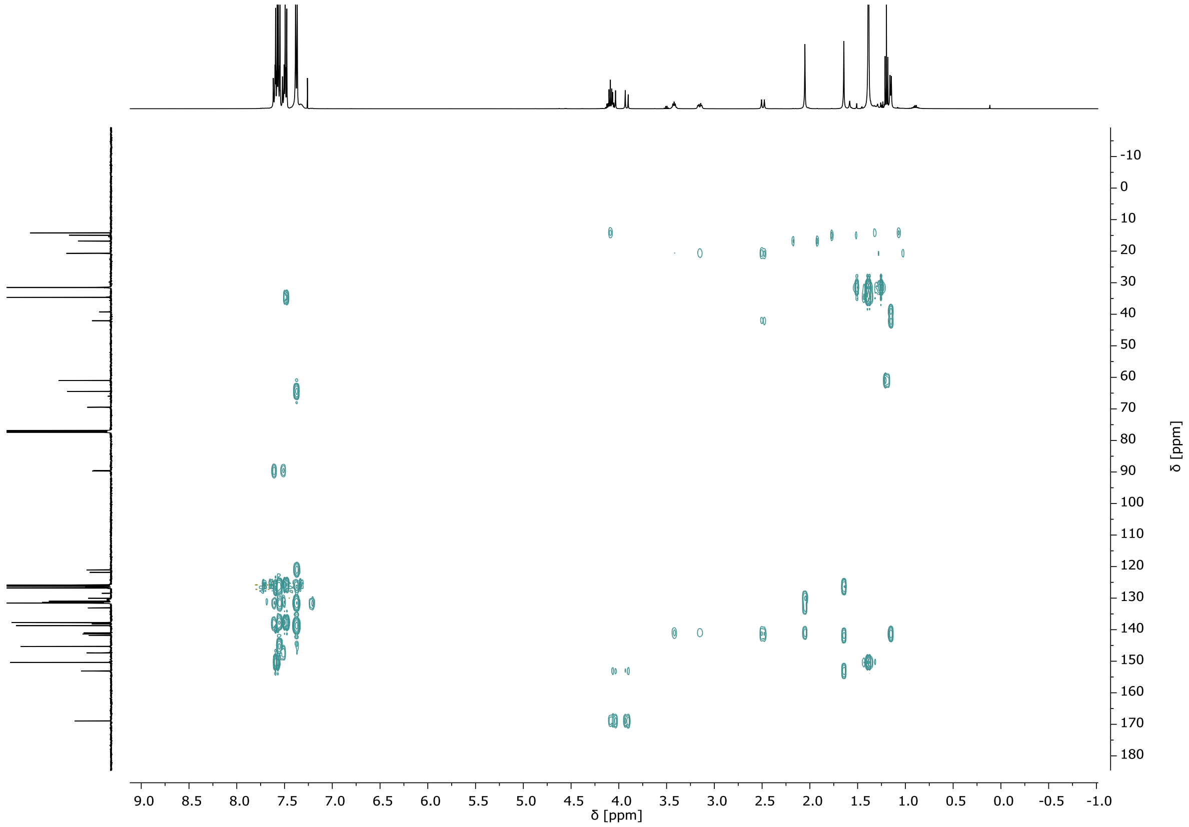

¹H, ¹³C HMBC NMR Spectrum (500 MHz, 25 °C) of **S13** in $CDCl_3$.

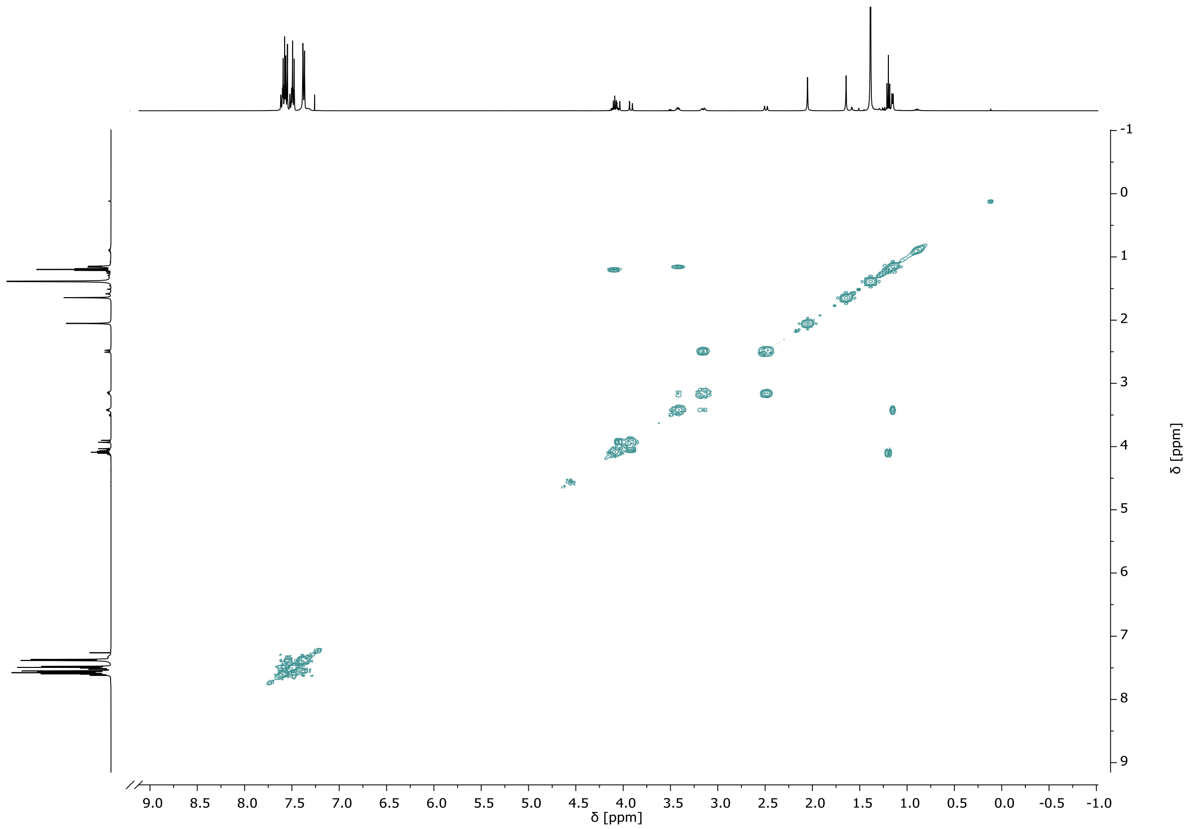

^{1}H COSY NMR Spectrum (500 MHz, 25 °C) of **S13** in $CDCl_3$.

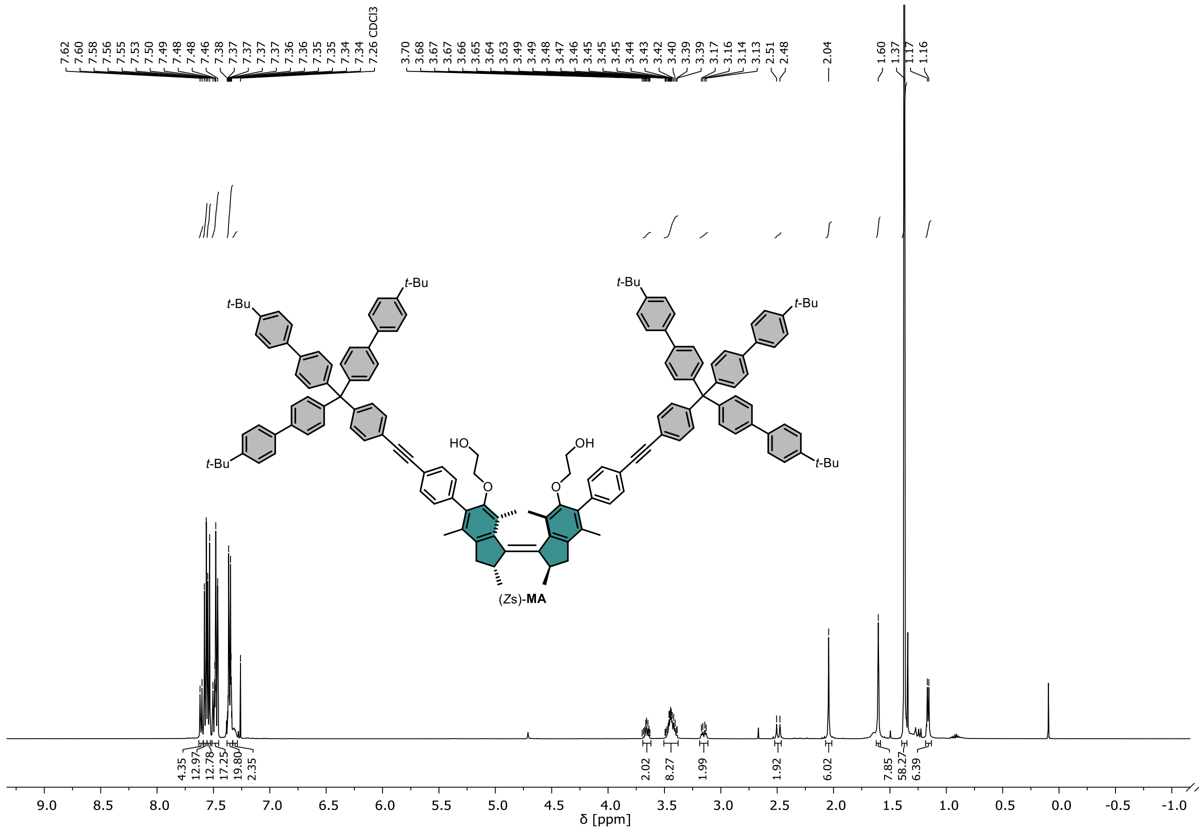

^{1}H NMR Spectrum (500 MHz, 25 °C) of motor axle (*Z*s)-**MA** in $CDCl_3$.

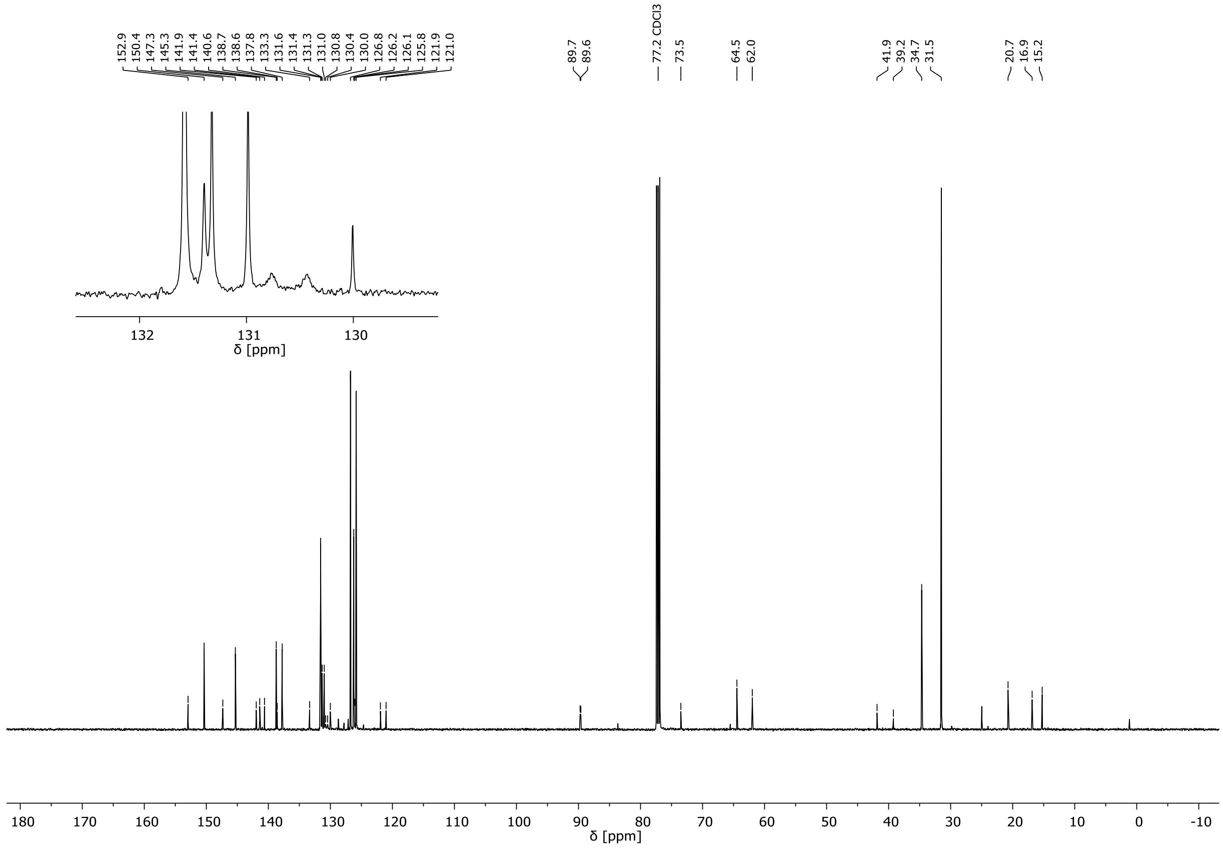

^{13}C NMR Spectrum (126 MHz, 25 °C) of motor axle (*Z*s)-**MA** in $CDCl_3$.

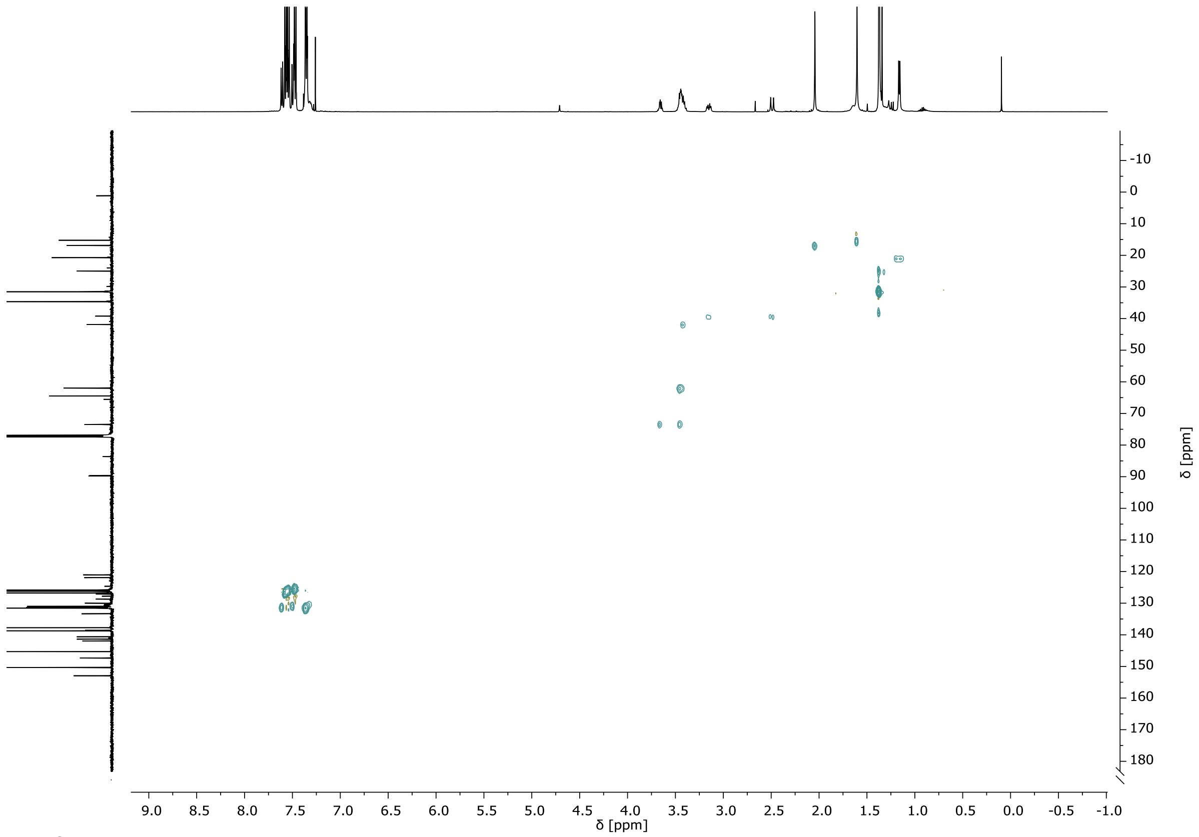

¹H, ¹³C HSQC NMR Spectrum (500 MHz, 25 °C) of motor axle (*Z*s)-**MA** in $CDCl_3$.

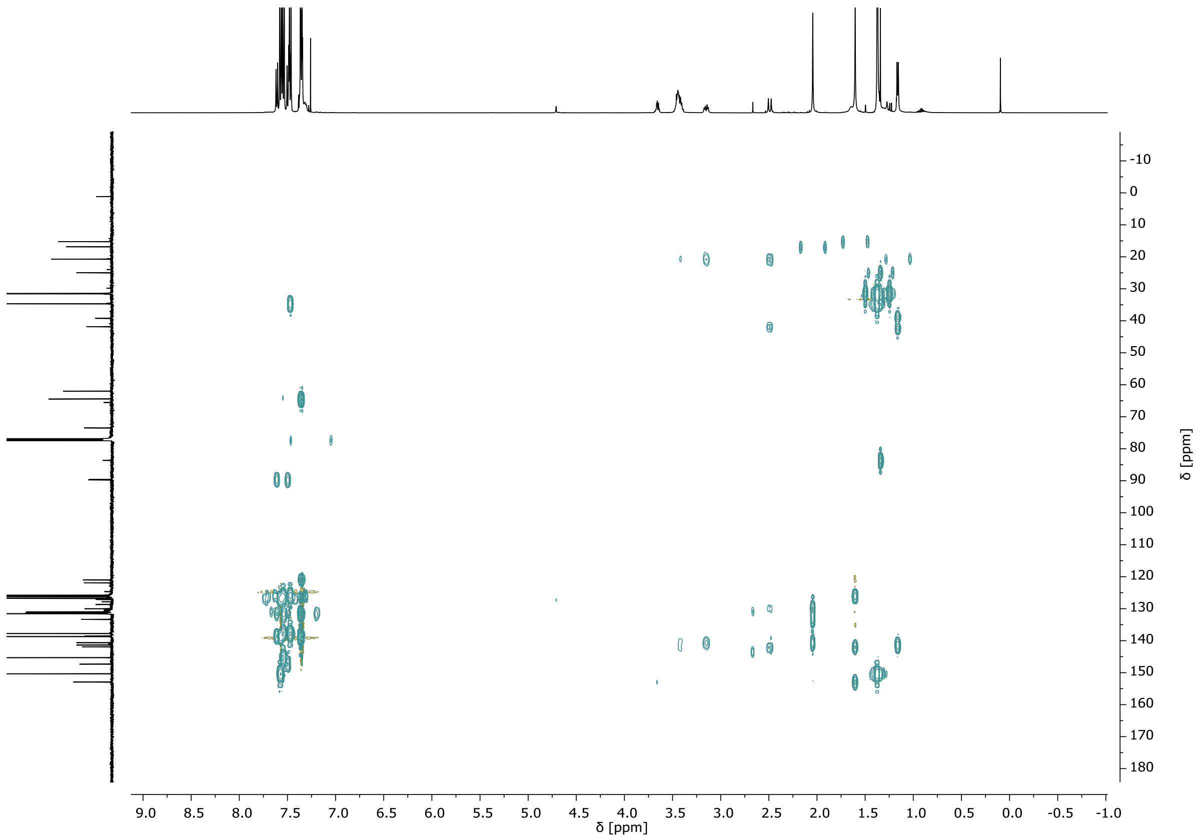

¹H, ¹³C HMBC NMR Spectrum (500 MHz, 25 °C) of motor axle (*Z*s)-**MA** in $CDCl_3$.

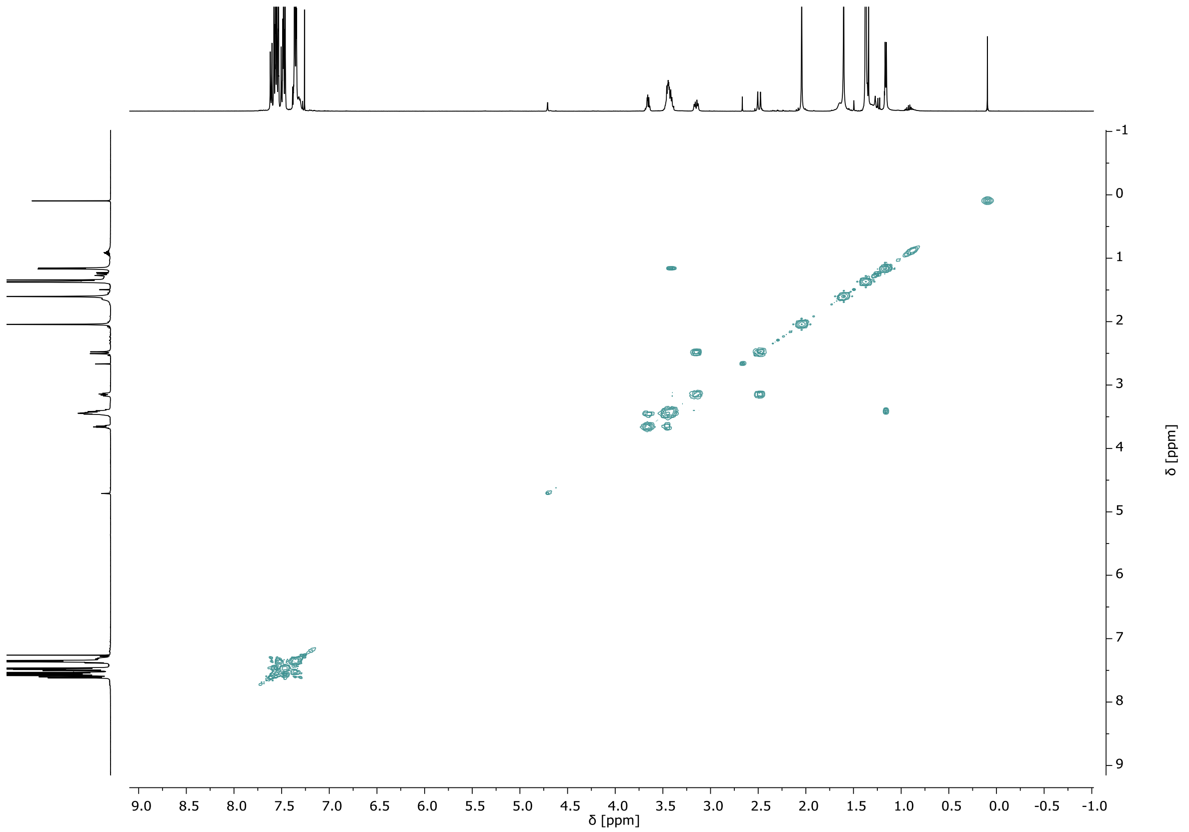

^{1}**H COSY NMR** Spectrum (500 MHz, 25 °C) of motor axle (*Z*s)-**MA** in $CDCl_3$.

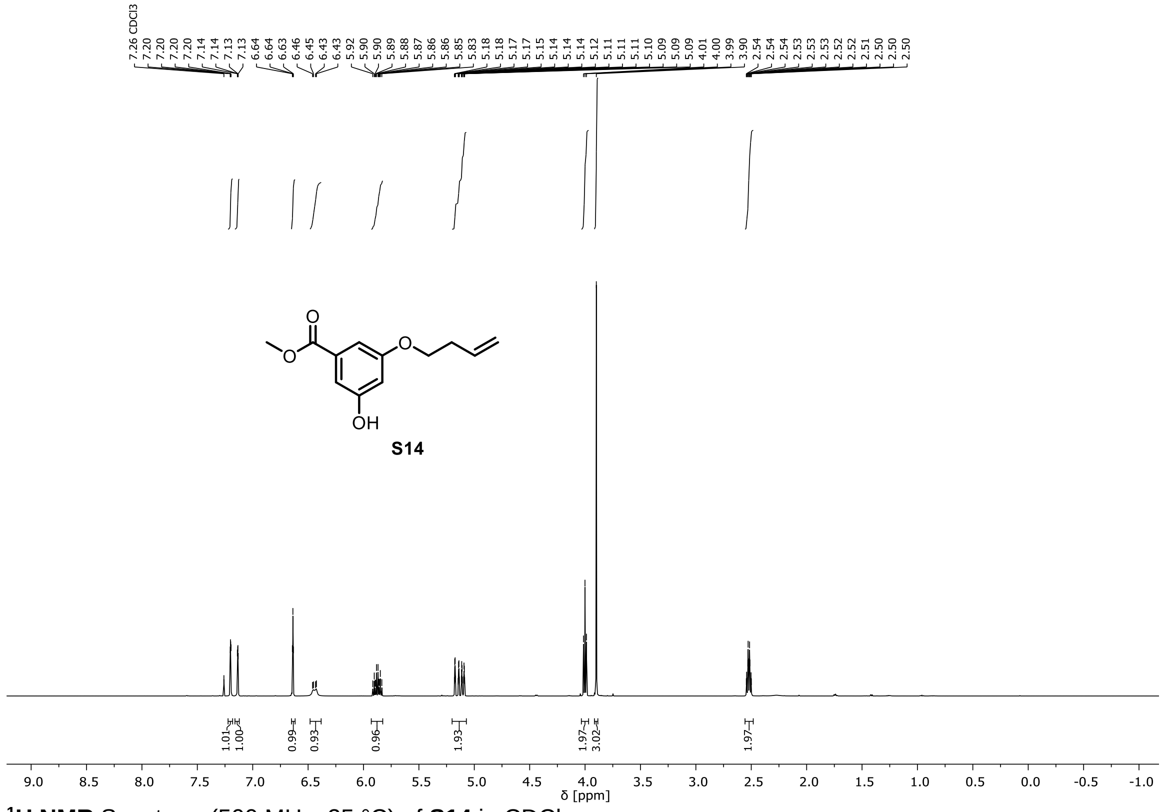

^{1}H NMR Spectrum (500 MHz, 25 °C) of **S14** in $CDCl_3$.

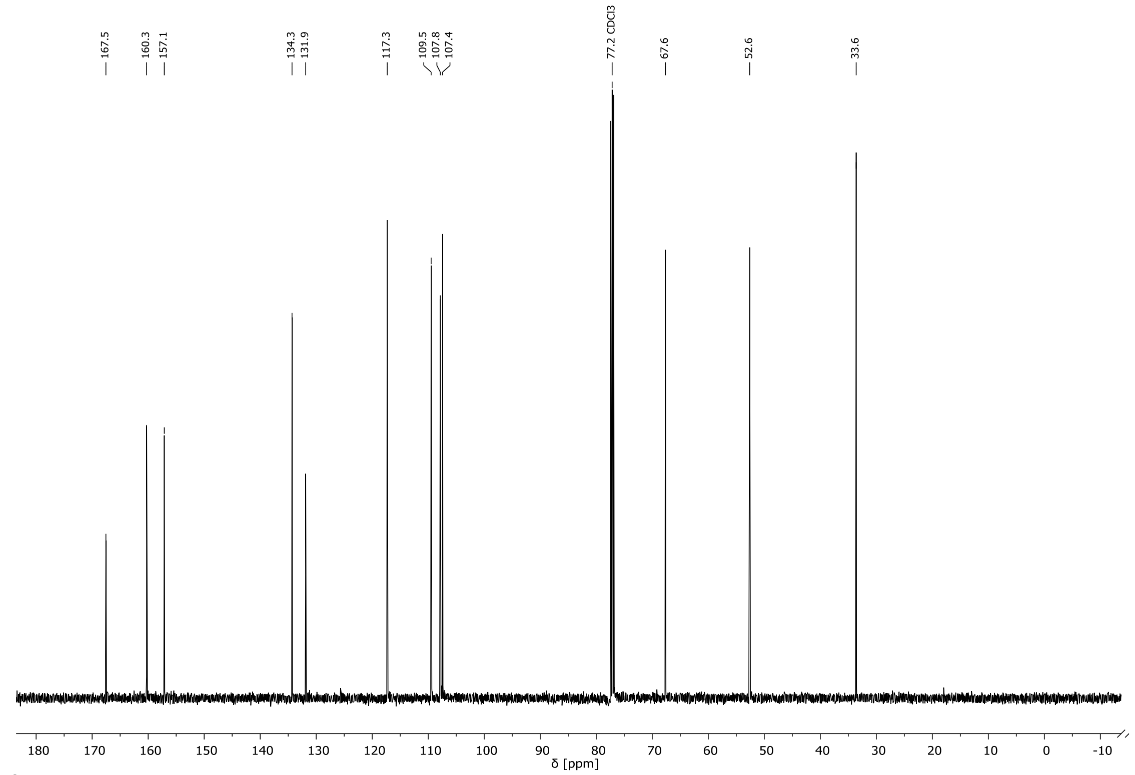

^{13}C NMR Spectrum (126 MHz, 25 °C) of **S14** in $CDCl_3$.

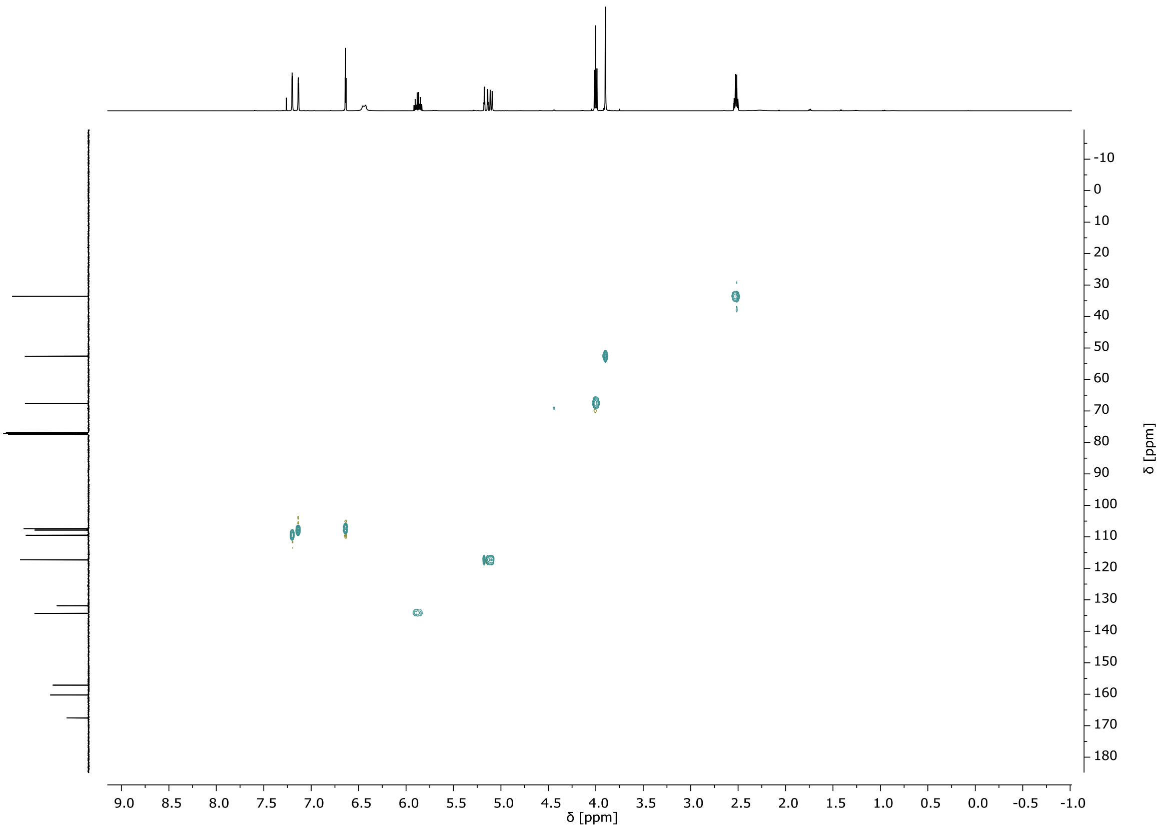

¹H, ¹³C HSQC NMR Spectrum (500 MHz, 25 °C) of **S14** in $CDCl_3$.

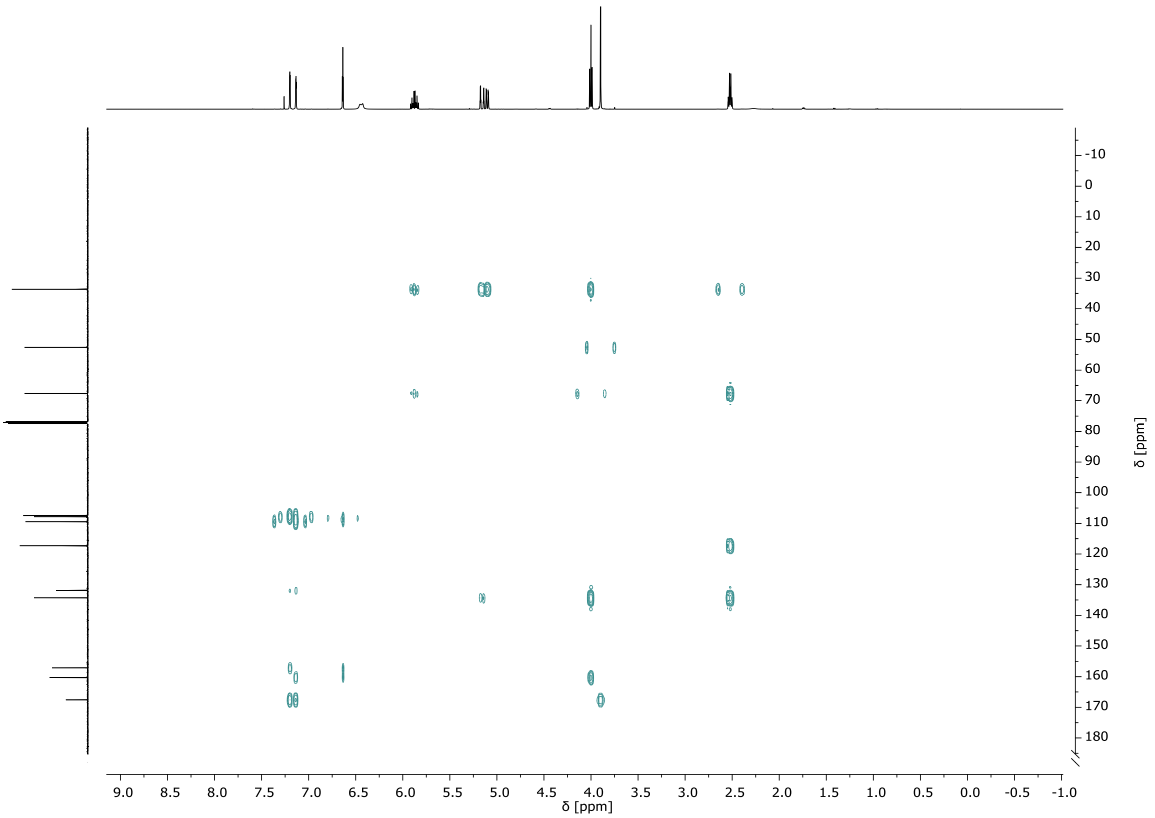

¹H, ¹³C HMBC NMR Spectrum (500 MHz, 25 °C) of **S14** in $CDCl_3$.

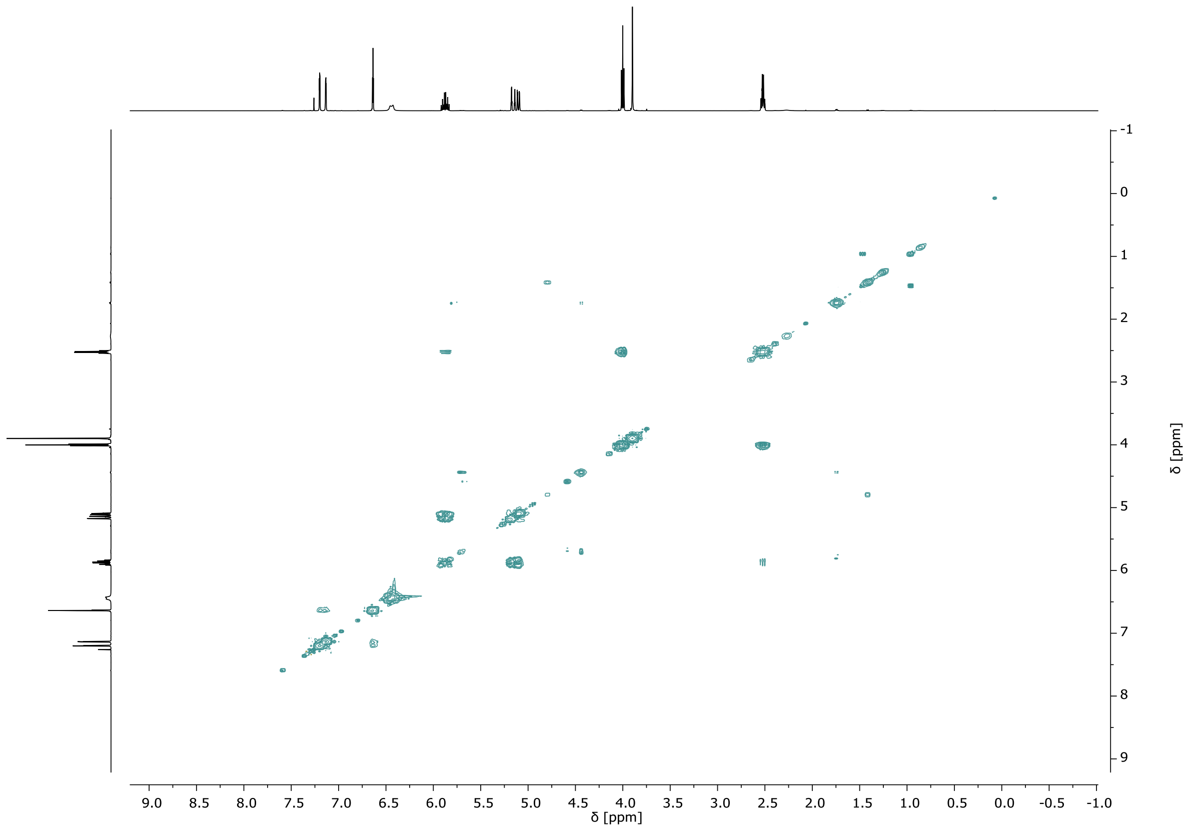

^{1}H COSY NMR Spectrum (500 MHz, 25 °C) of **S14** in $CDCl_3$.

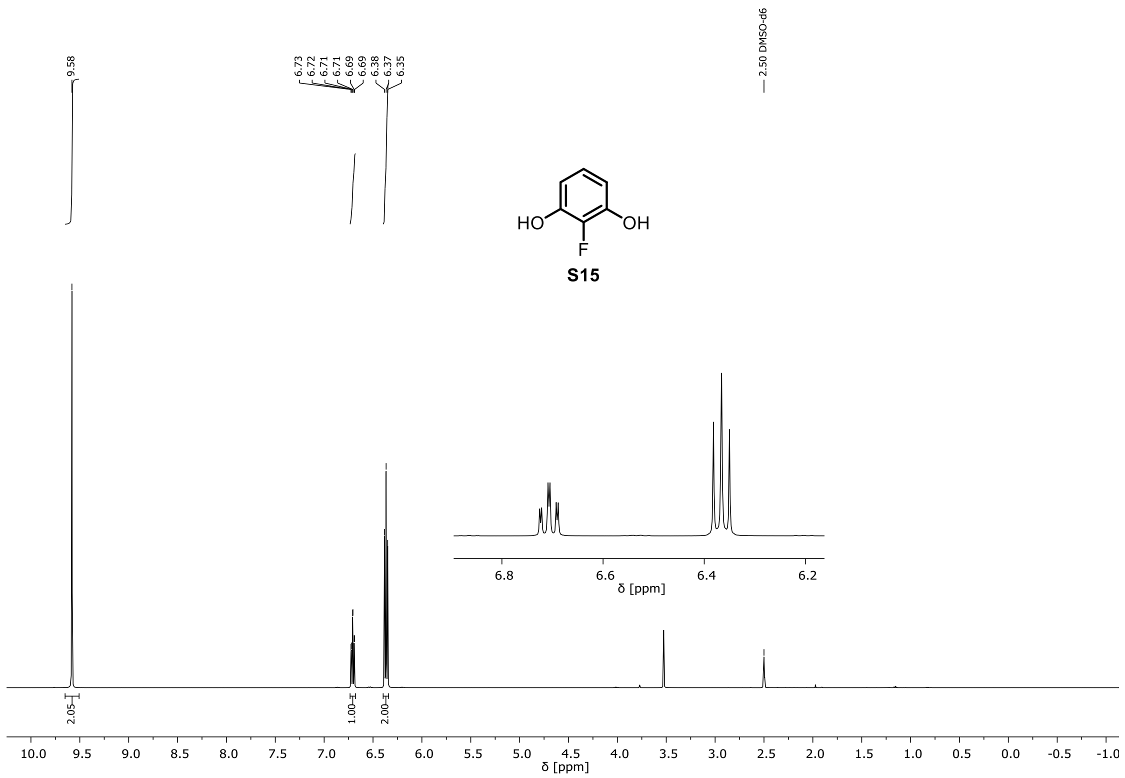

^{1}H NMR Spectrum (500 MHz, 25 °C) of **S15** in DMSO-d_6.

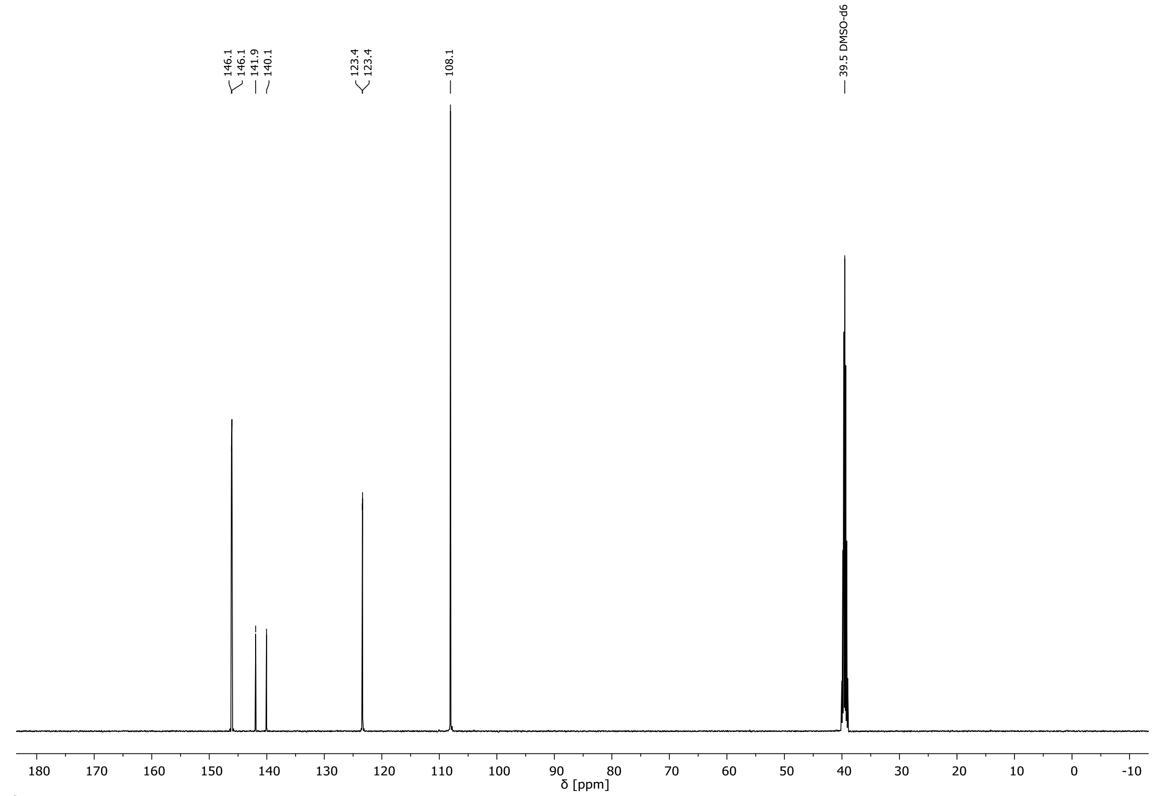

^{13}C NMR Spectrum (126 MHz, 25 °C) of **S15** in DMSO-d_6.

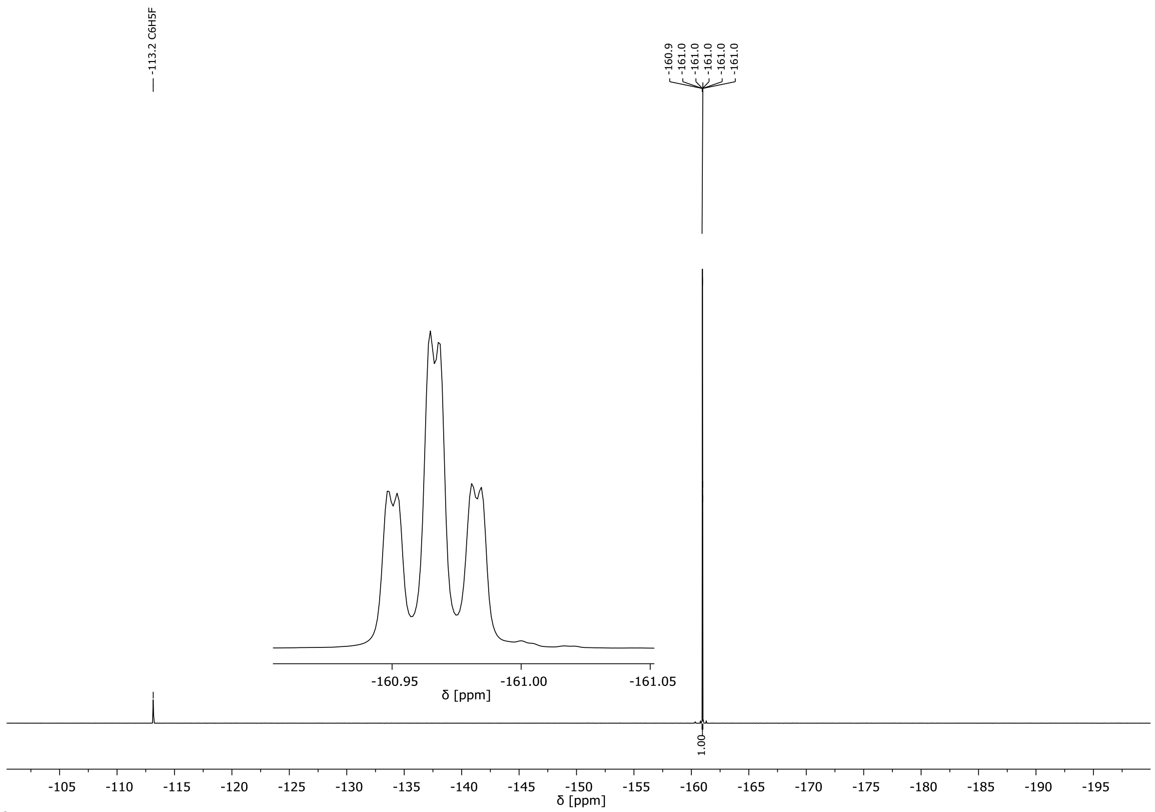

^{19}F NMR Spectrum (471 MHz, 25 °C) of **S15** in DMSO-d_6.

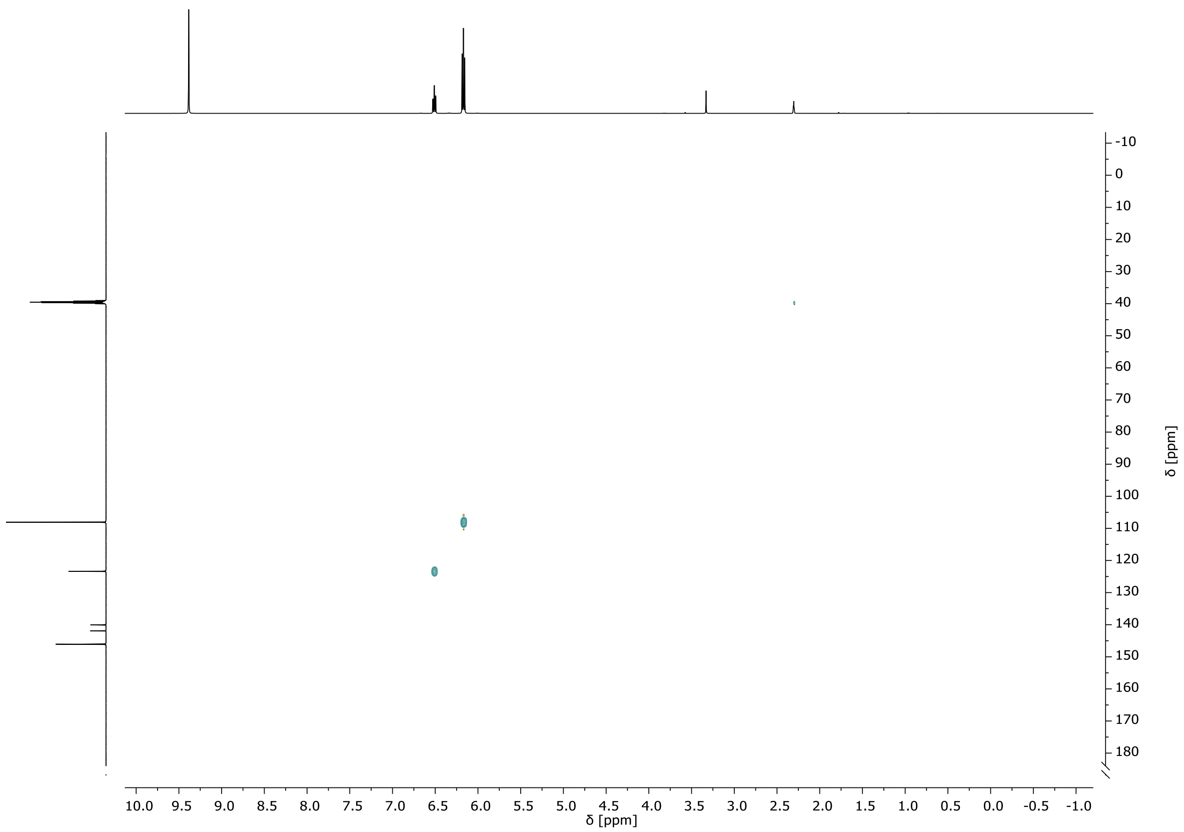

^{1}H, ^{13}C HSQC NMR Spectrum (500 MHz, 25 °C) of **S15** in DMSO-d_6.

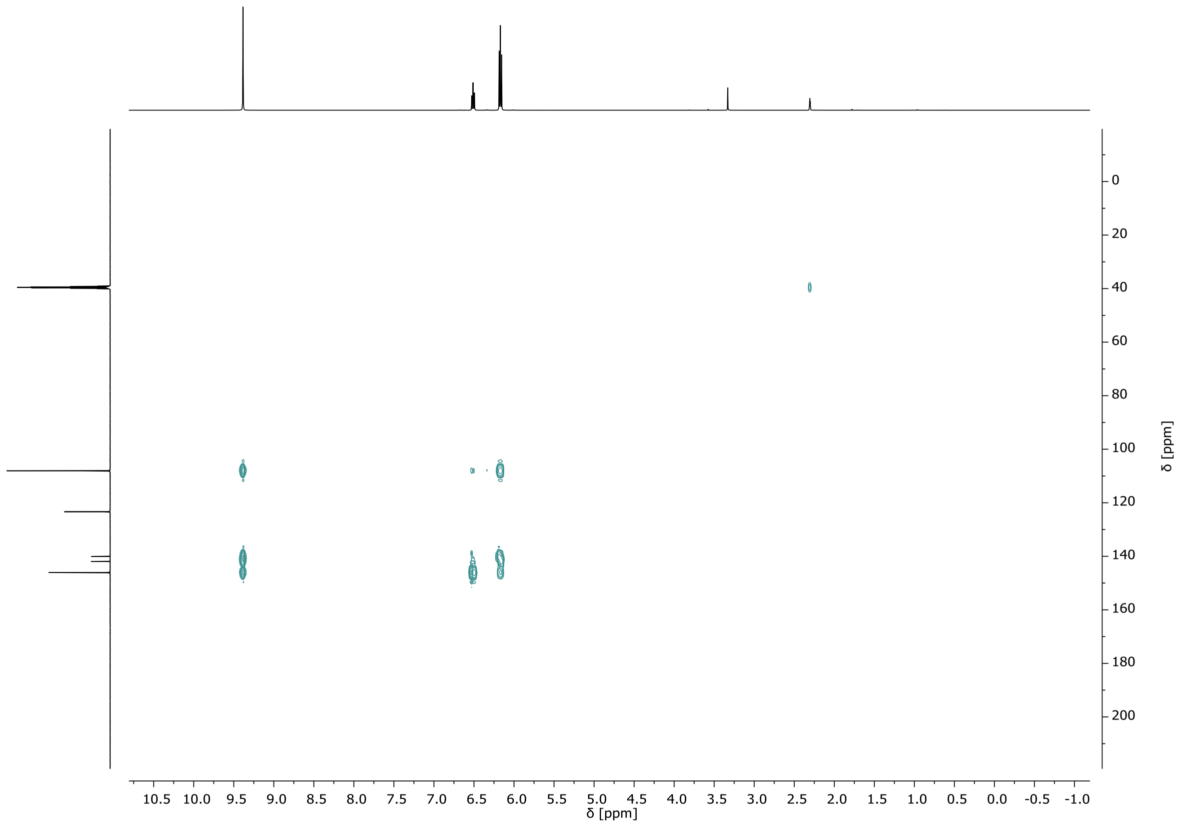

^{1}H, ^{13}C HMBC NMR Spectrum (500 MHz, 25 °C) of **S15** in DMSO-d_6.

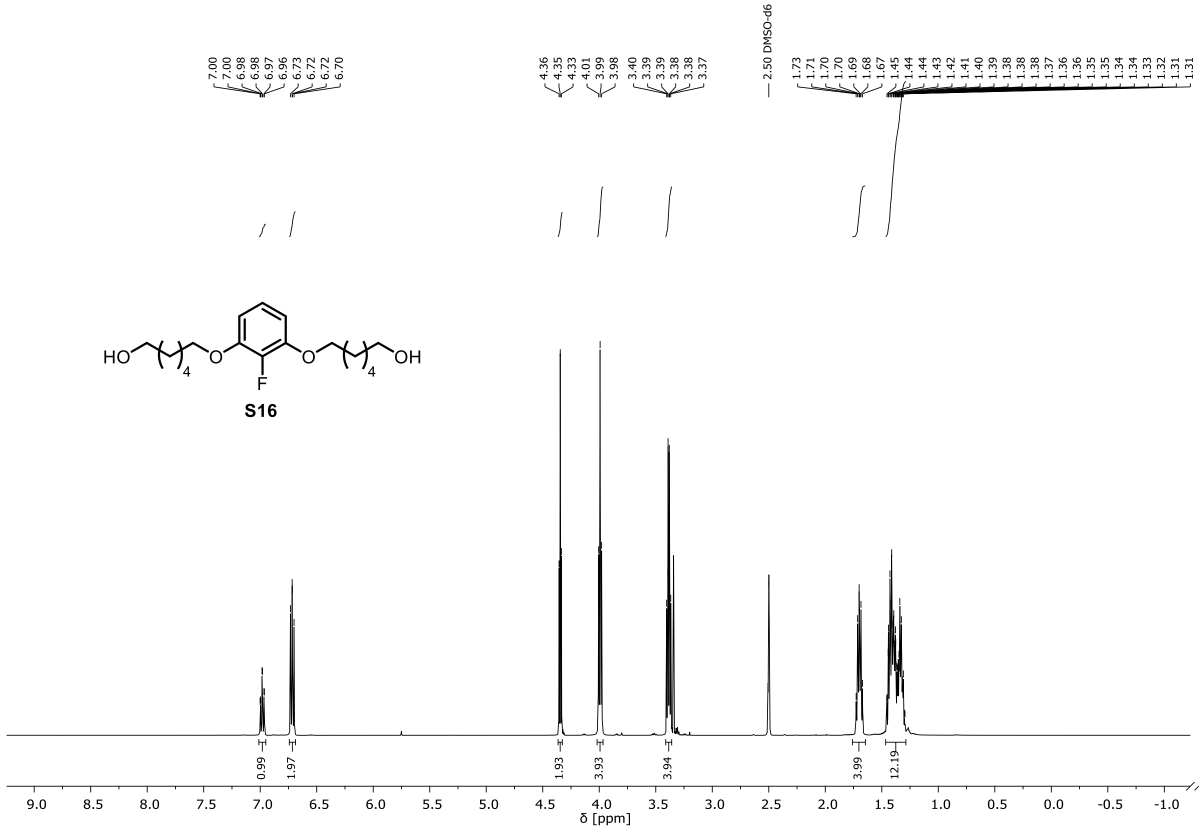

^{1}H NMR Spectrum (500 MHz, 25 °C) of **S16** in DMSO-d_6.

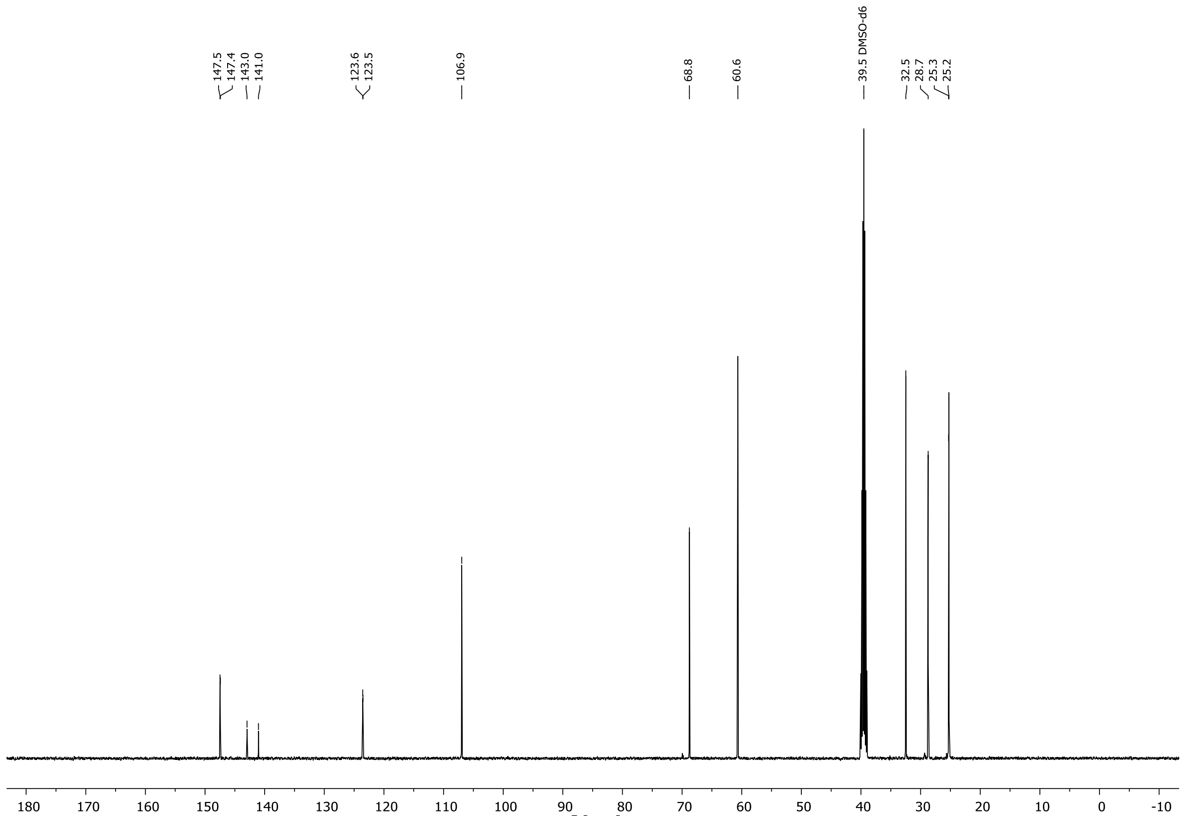

^{13}C NMR Spectrum (126 MHz, 25 °C) of **S16** in DMSO-d_6.

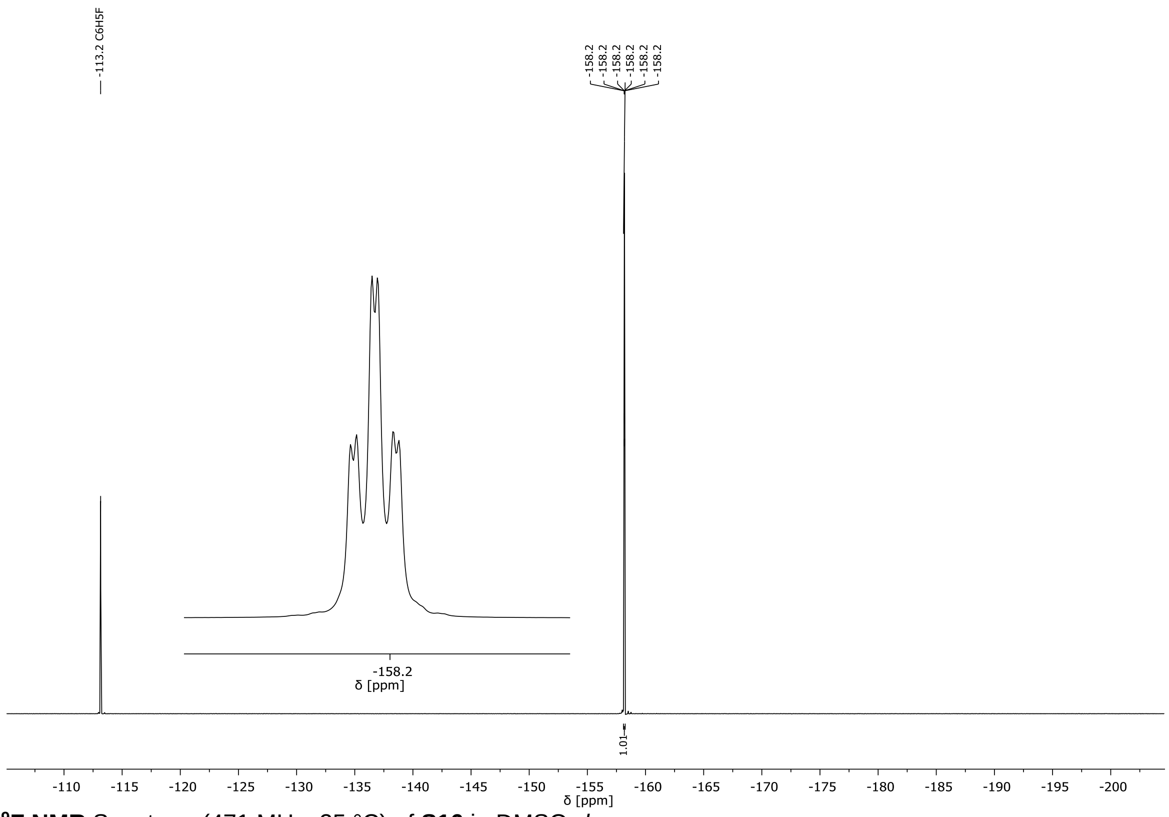

^{19}F NMR Spectrum (471 MHz, 25 °C) of **S16** in DMSO-d_6.

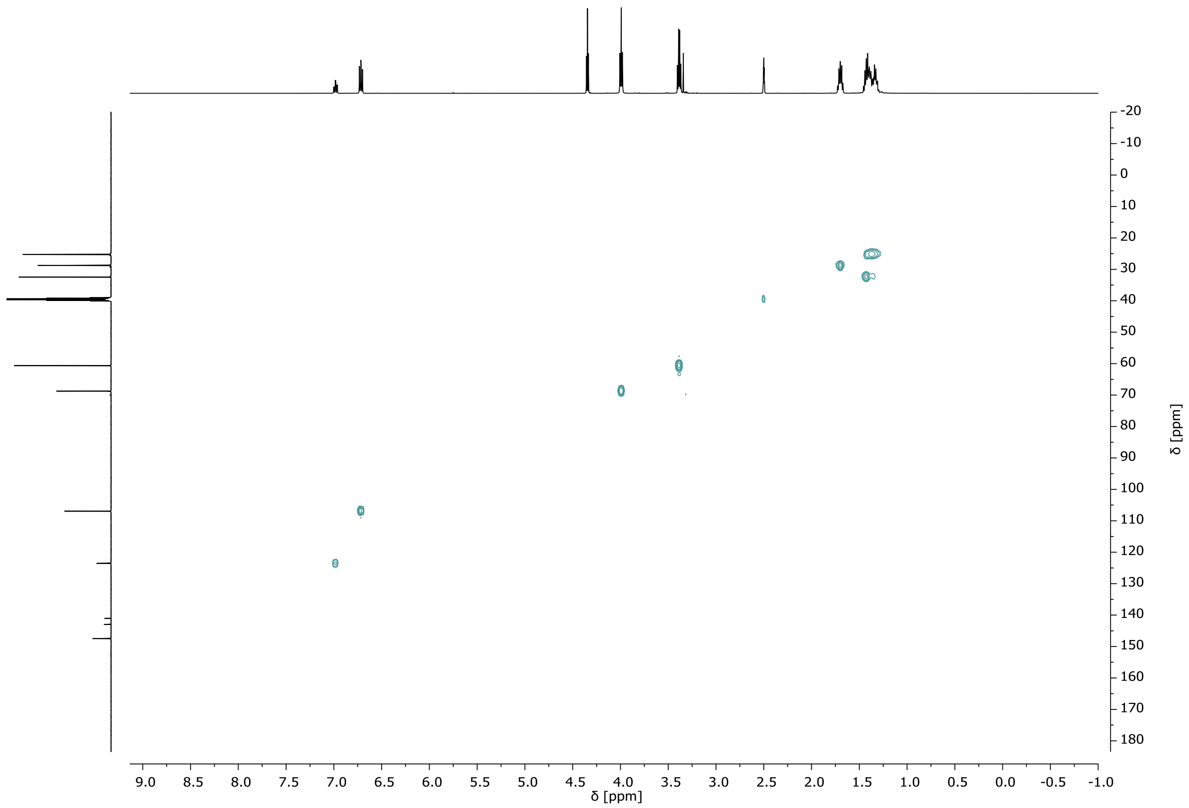

^{1}H, ^{13}C HSQC NMR Spectrum (500 MHz, 25 °C) of **S16** in DMSO-d_6.

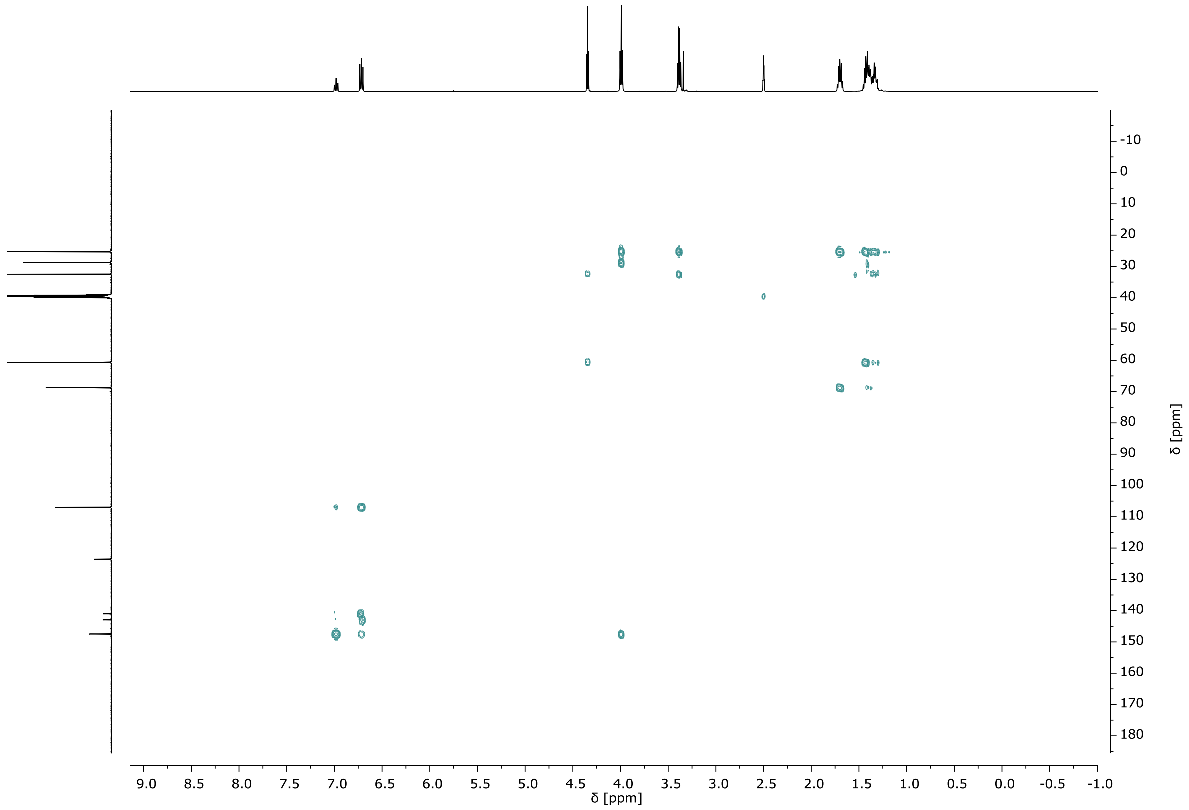

^{1}H, ^{13}C HMBC NMR Spectrum (500 MHz, 25 °C) of **S16** in DMSO-*d*6.

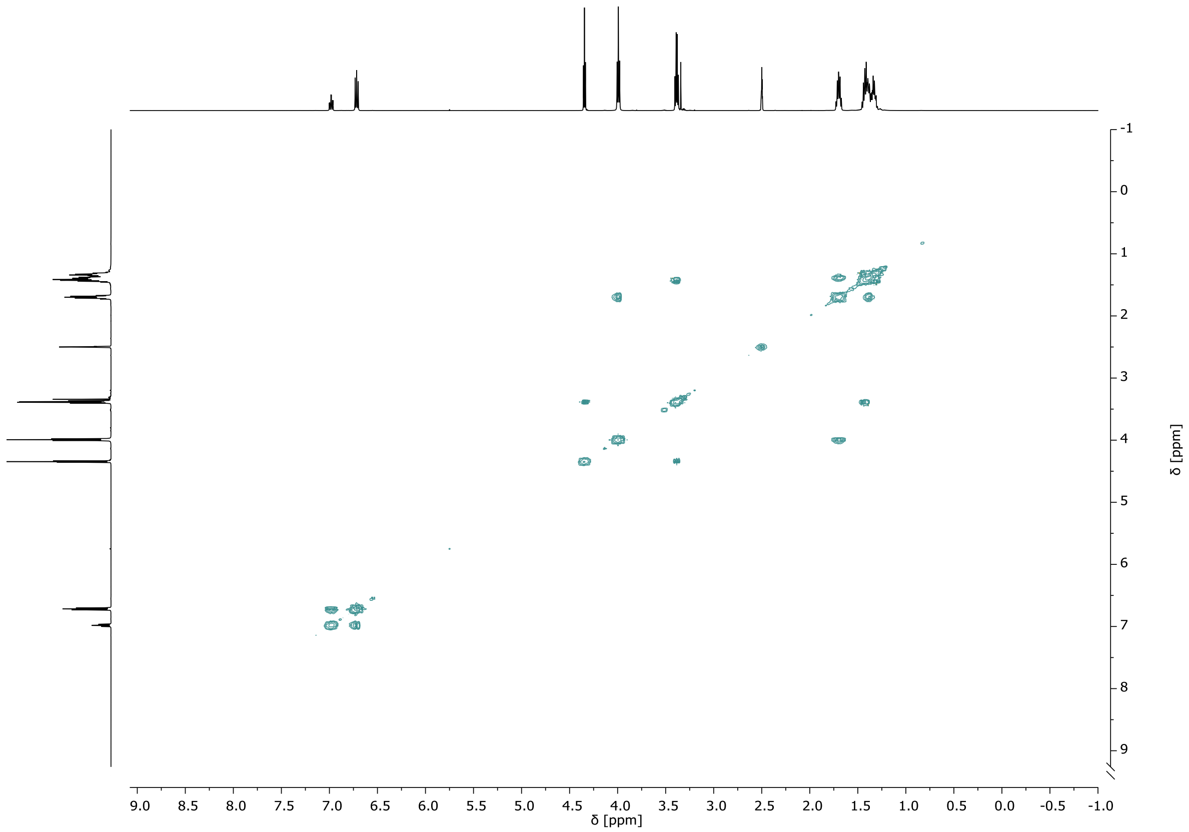

^{1}H COSY NMR Spectrum (500 MHz, 25 °C) of **S16** in DMSO-*d*6.

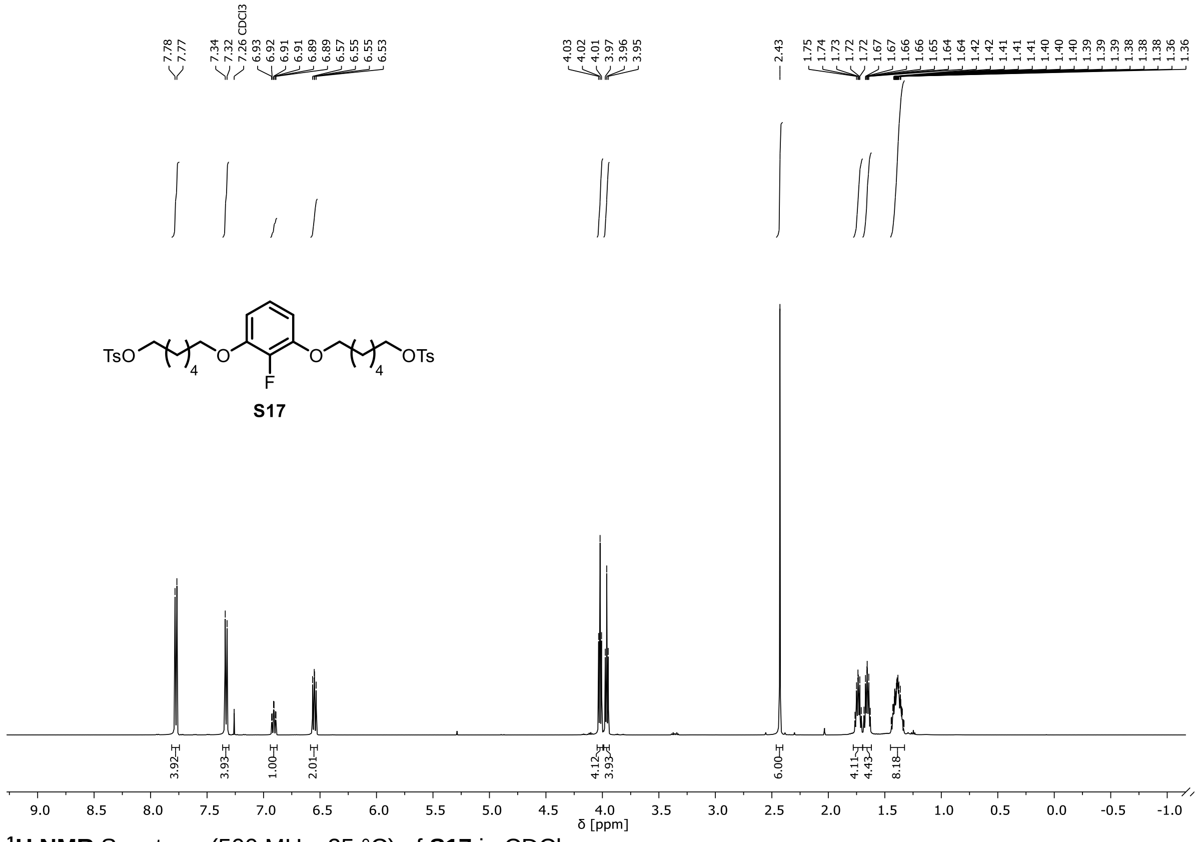

¹H NMR Spectrum (500 MHz, 25 °C) of **S17** in $CDCl_3$.

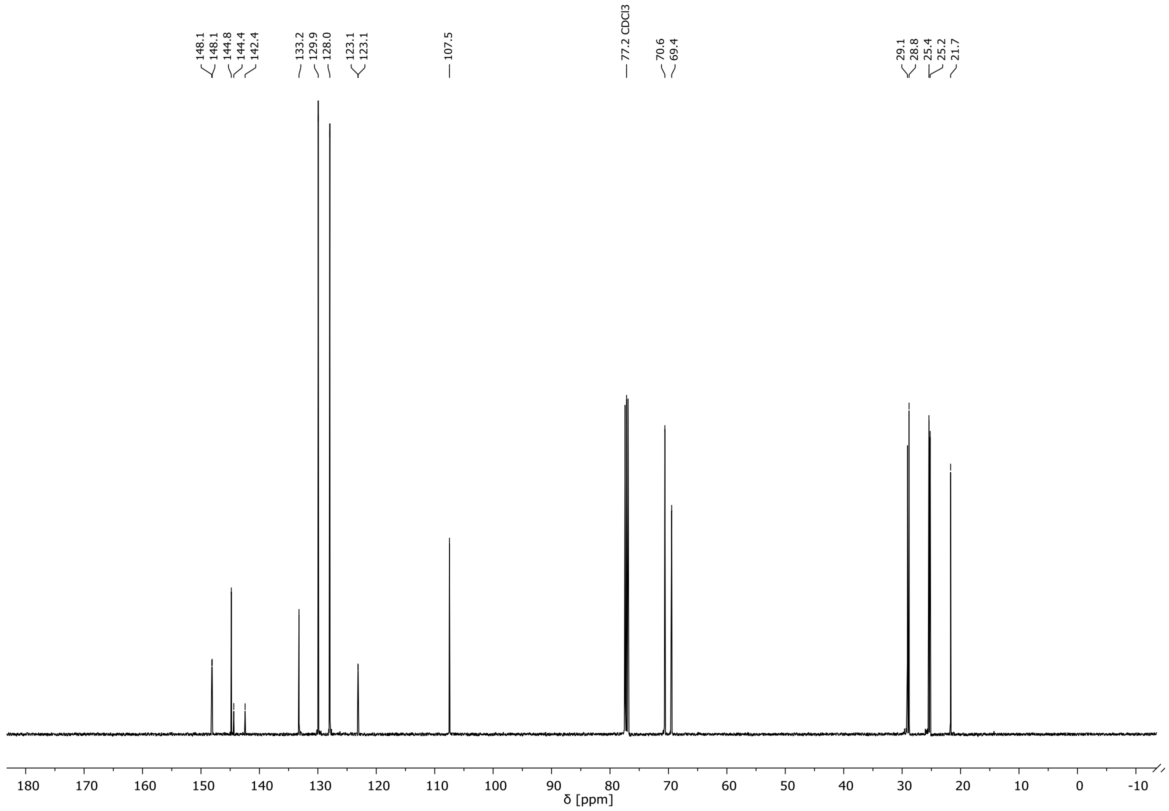

¹³C NMR Spectrum (126 MHz, 25 °C) of **S17** in $CDCl_3$.

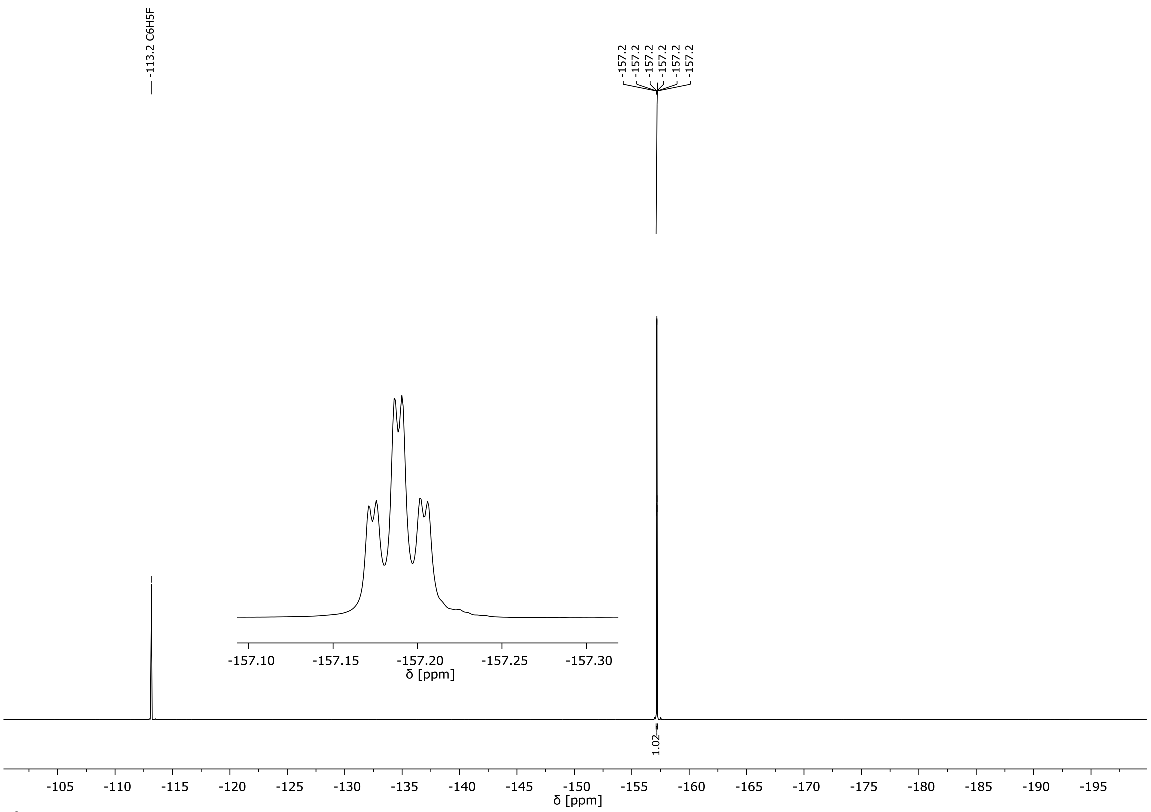

^{19}F NMR Spectrum (471 MHz, 25 °C) of **S17** in $CDCl_3$.

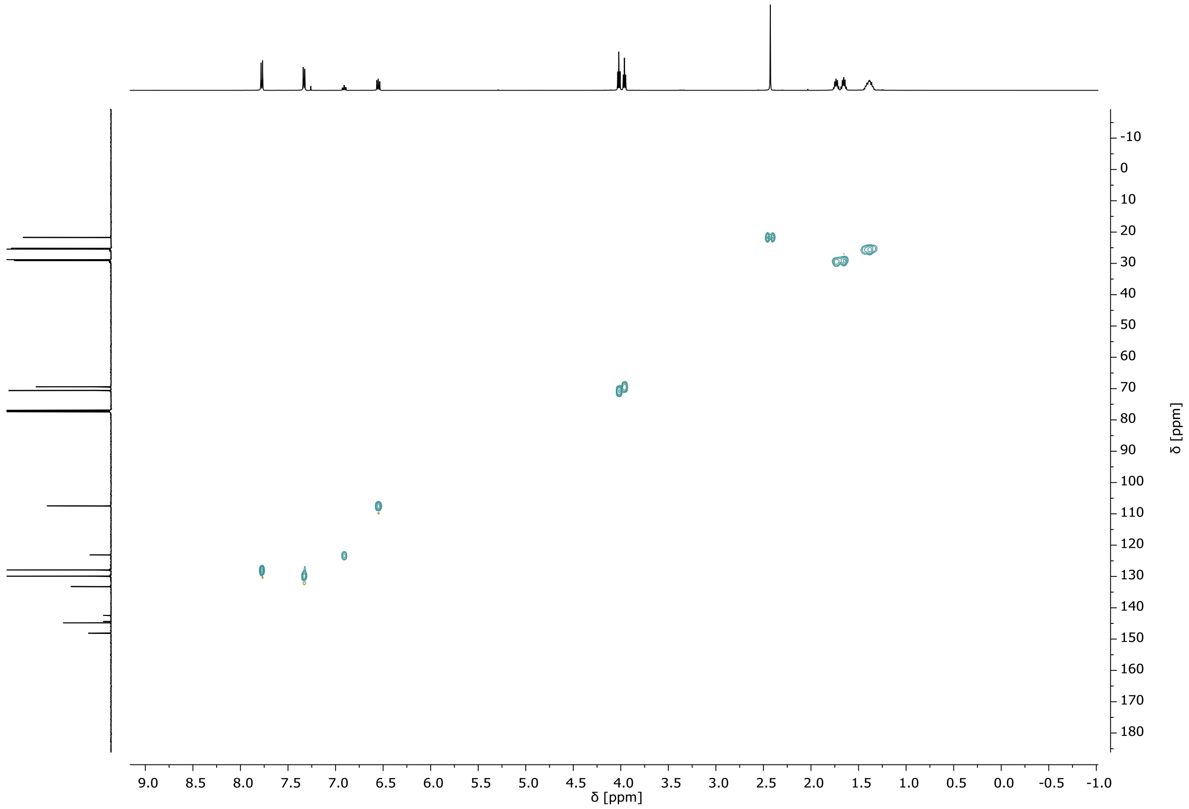

^{1}H, ^{13}C HSQC NMR Spectrum (500 MHz, 25 °C) of **S17** in $CDCl_3$.

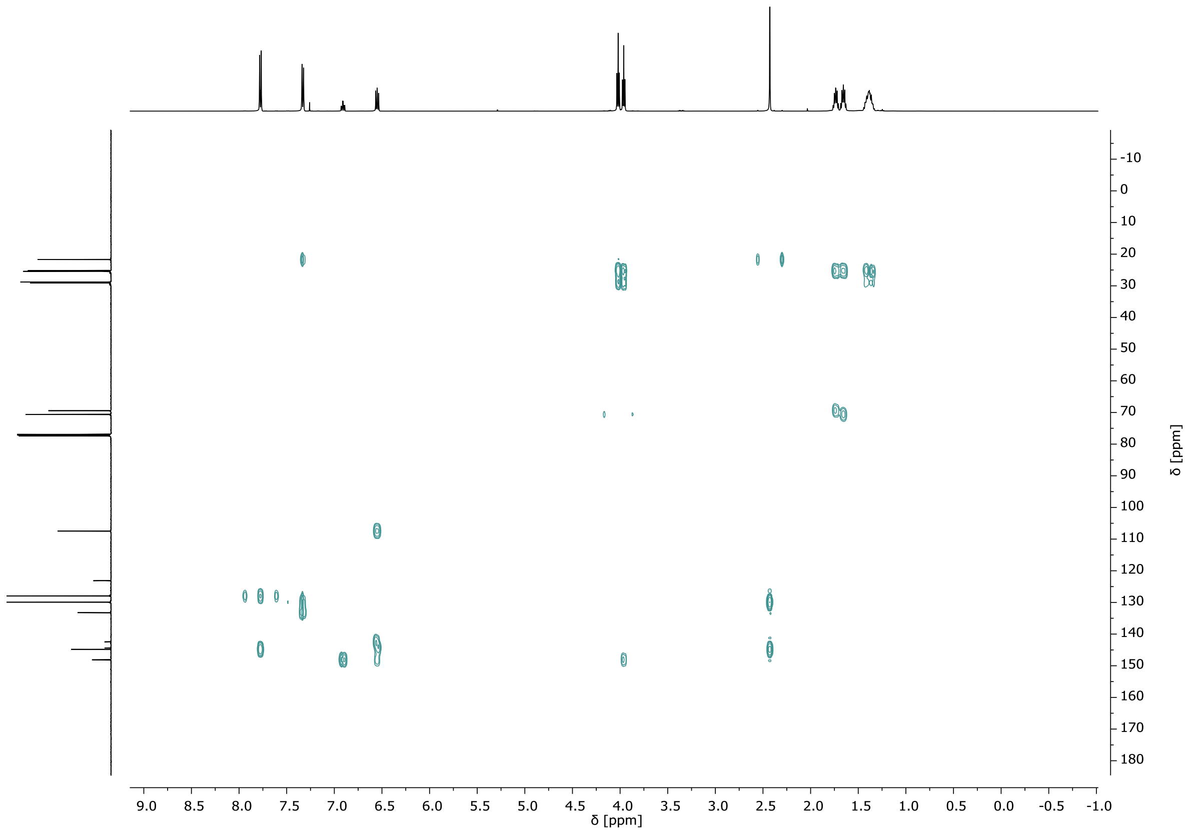

^{1}H, ^{13}C HMBC NMR Spectrum (500 MHz, 25 °C) of **S17** in $CDCl_3$.

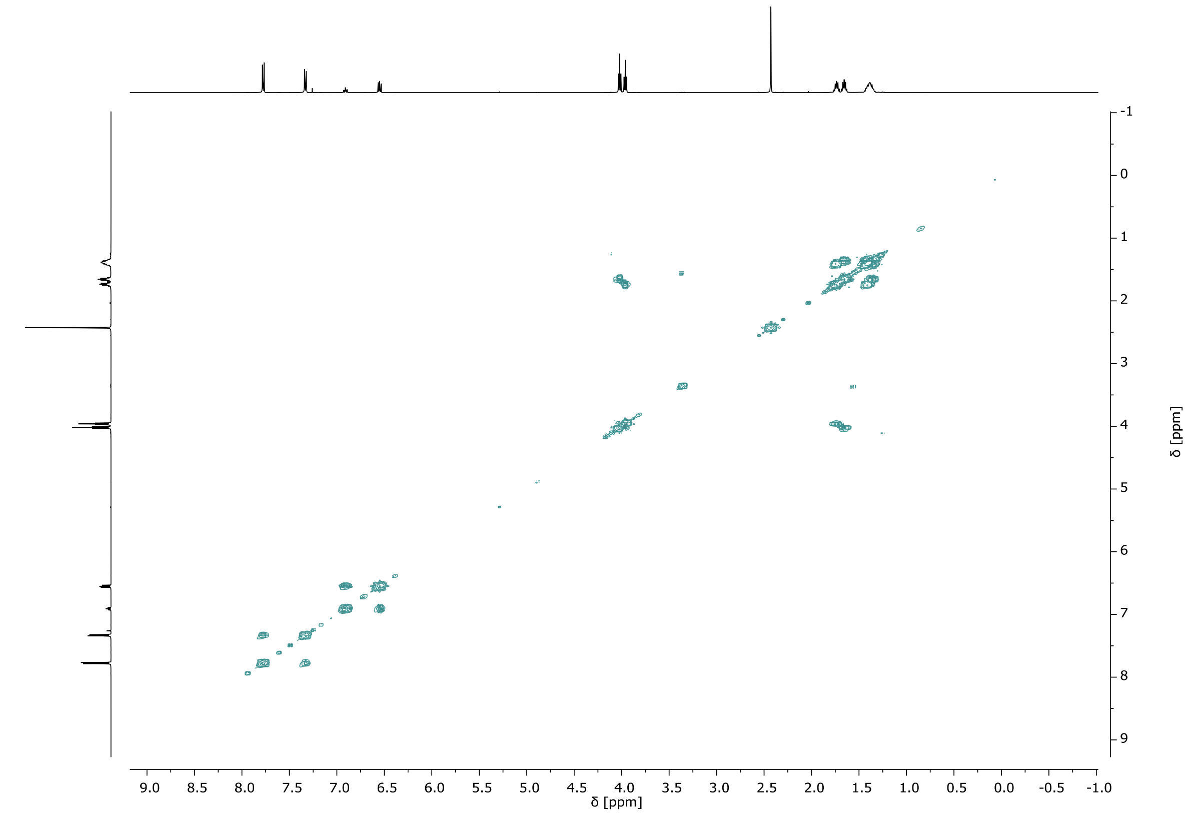

^{1}H COSY NMR Spectrum (500 MHz, 25 °C) of **S17** in $CDCl_3$.

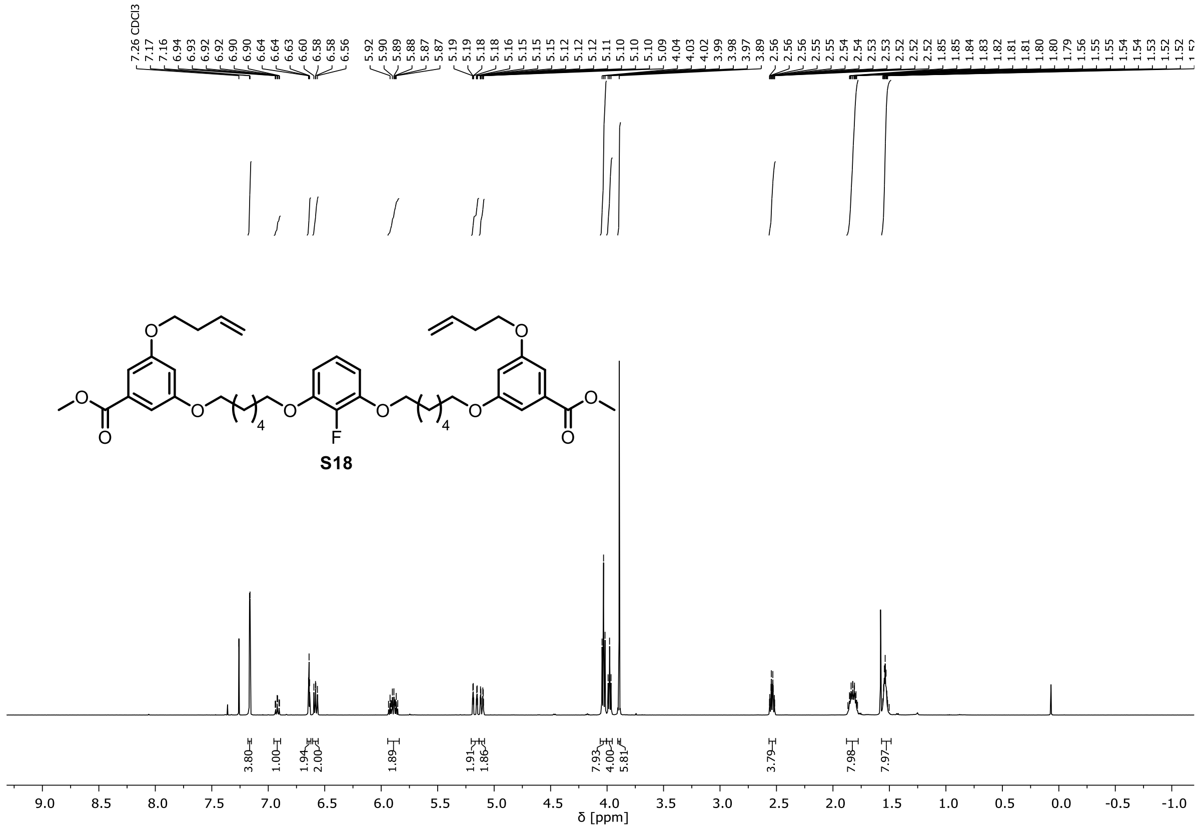

^{1}H NMR Spectrum (500 MHz, 25 °C) of **S18** in $CDCl_3$.

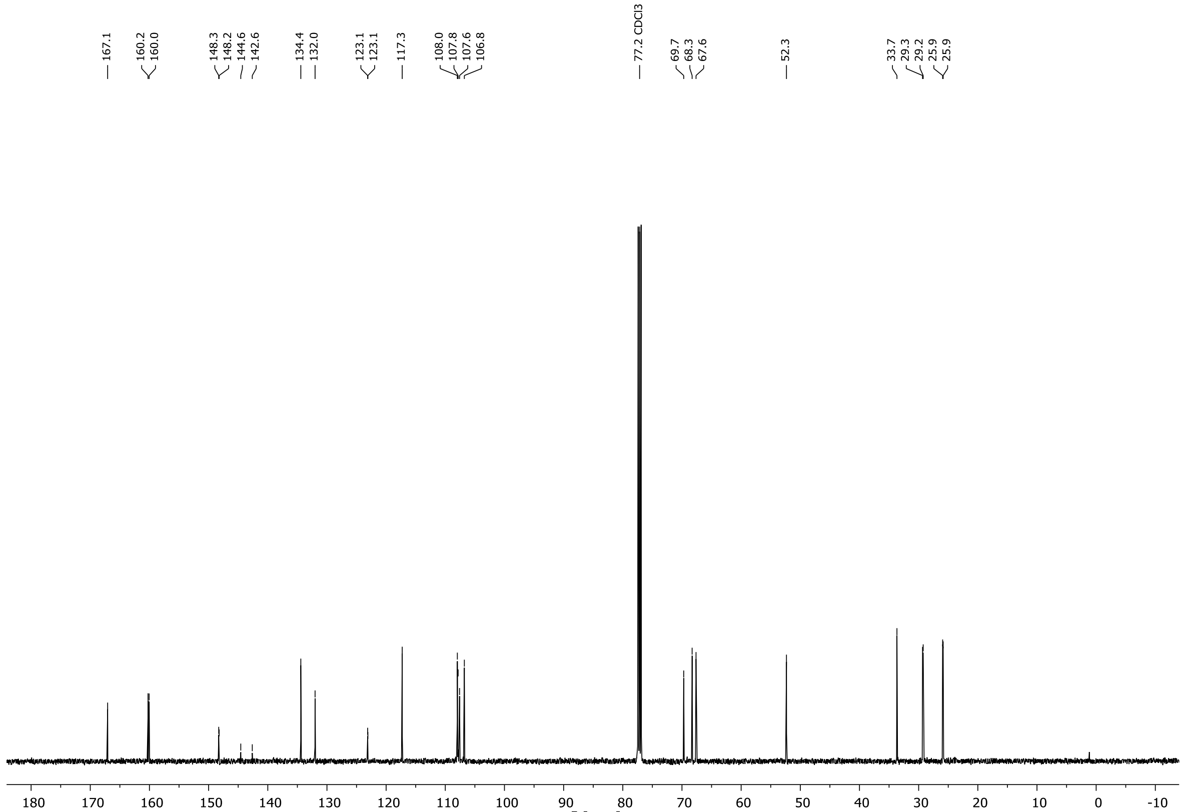

^{13}C NMR Spectrum (126 MHz, 25 °C) of **S18** in $CDCl_3$.

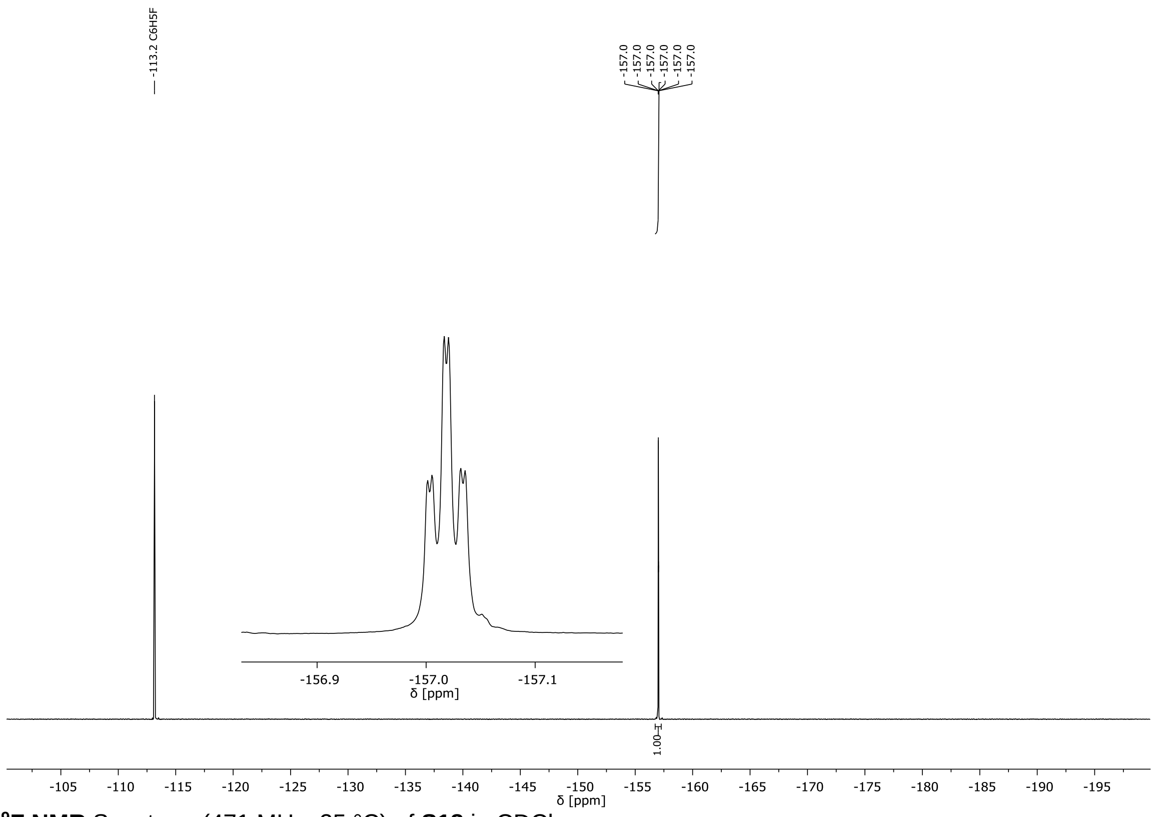

^{19}F NMR Spectrum (471 MHz, 25 °C) of **S18** in $CDCl_3$.

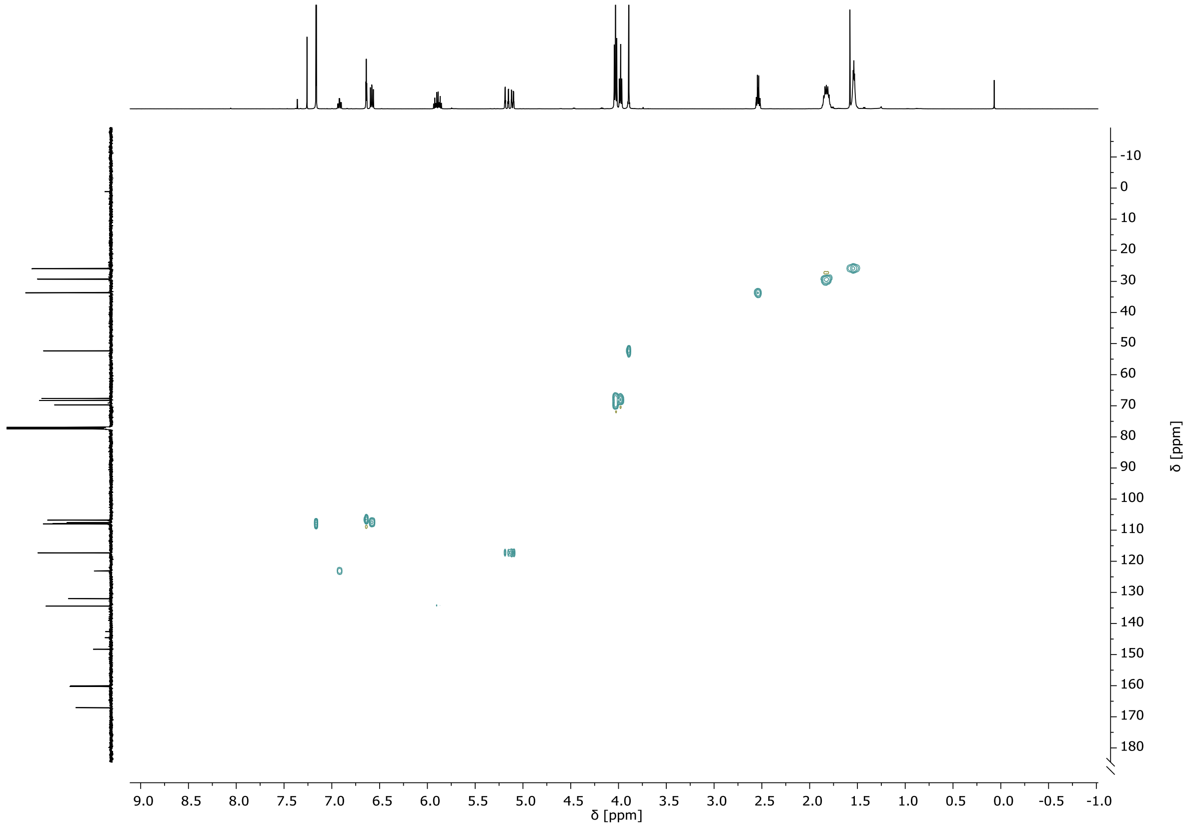

^{1}H, ^{13}C HSQC NMR Spectrum (500 MHz, 25 °C) of **S18** in $CDCl_3$.

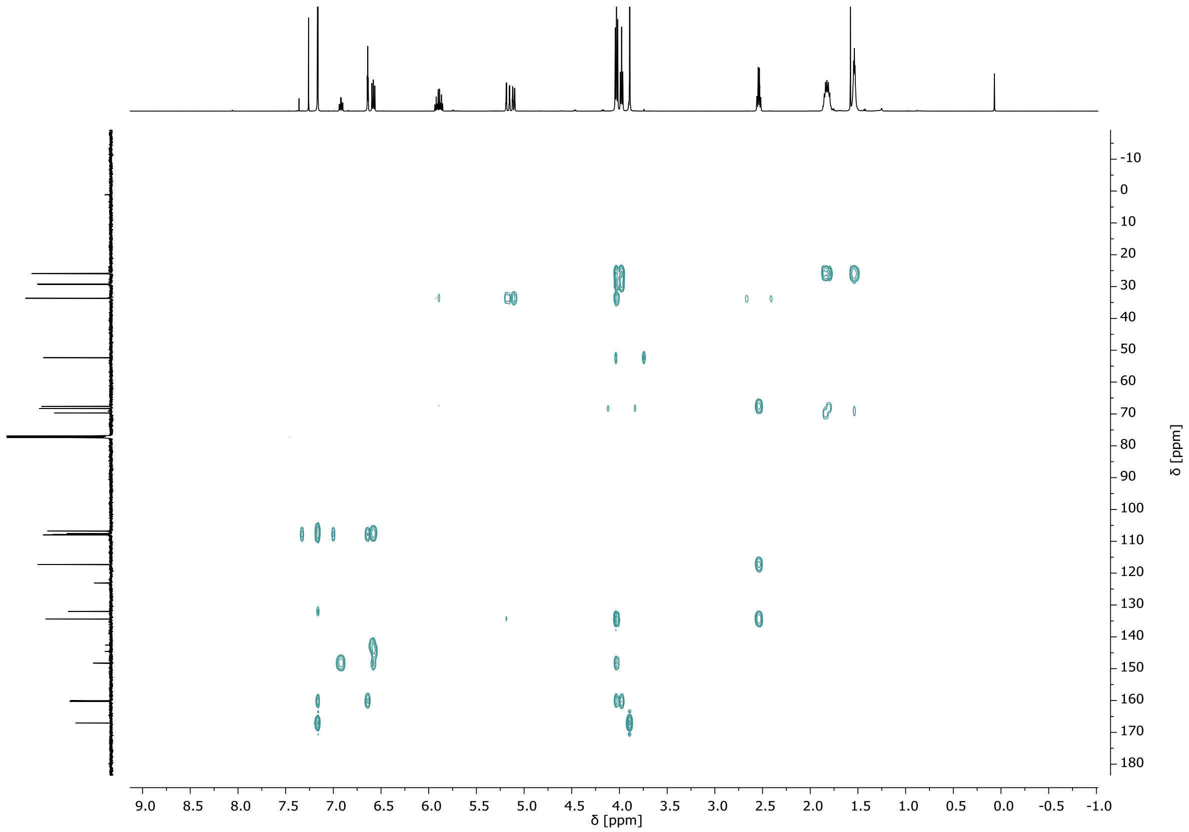

^{1}H, ^{13}C HMBC NMR Spectrum (500 MHz, 25 °C) of **S18** in $CDCl_3$.

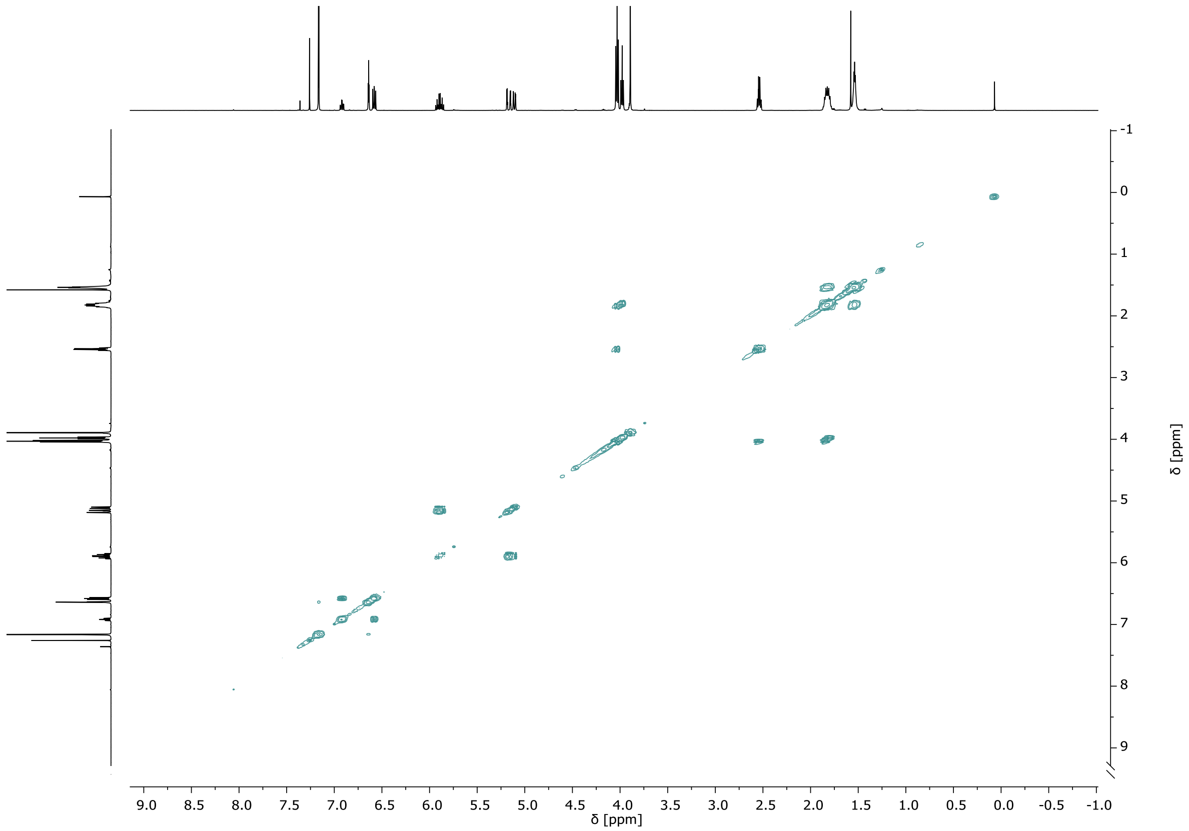

^{1}H COSY NMR Spectrum (500 MHz, 25 °C) of **S18** in $CDCl_3$.

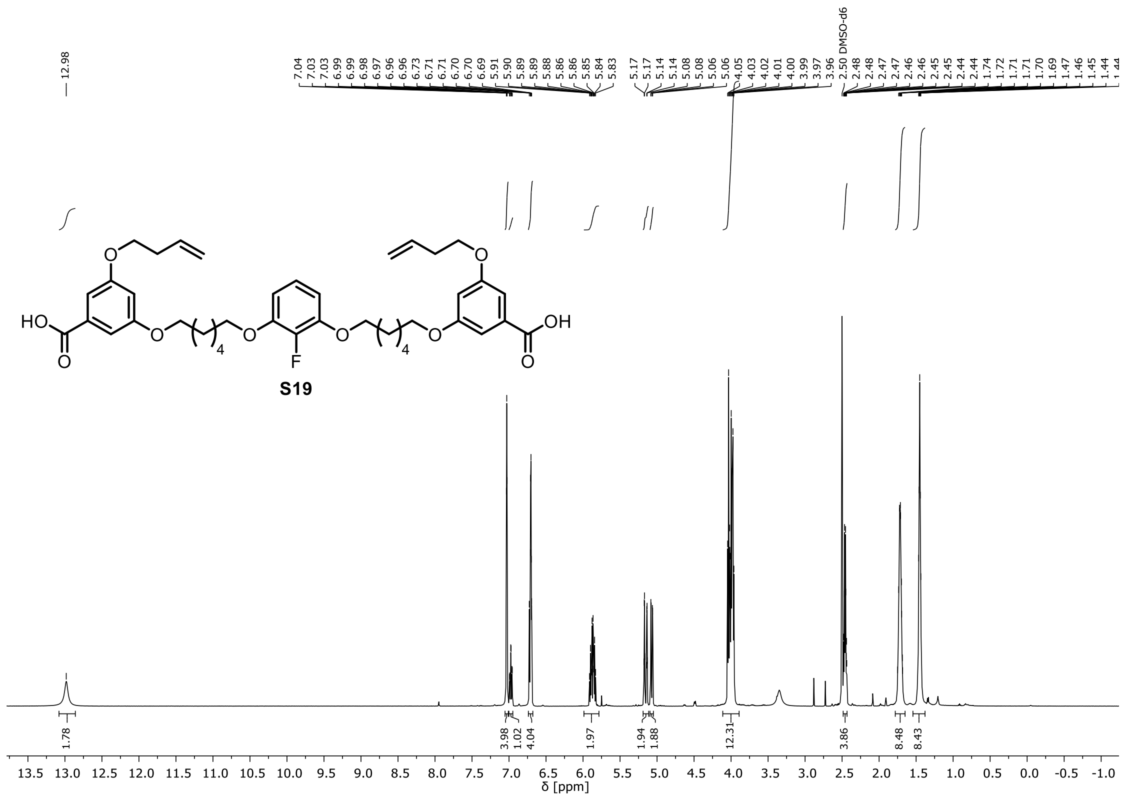

^{1}H NMR Spectrum (500 MHz, 25 °C) of **S19** in DMSO-d_6.

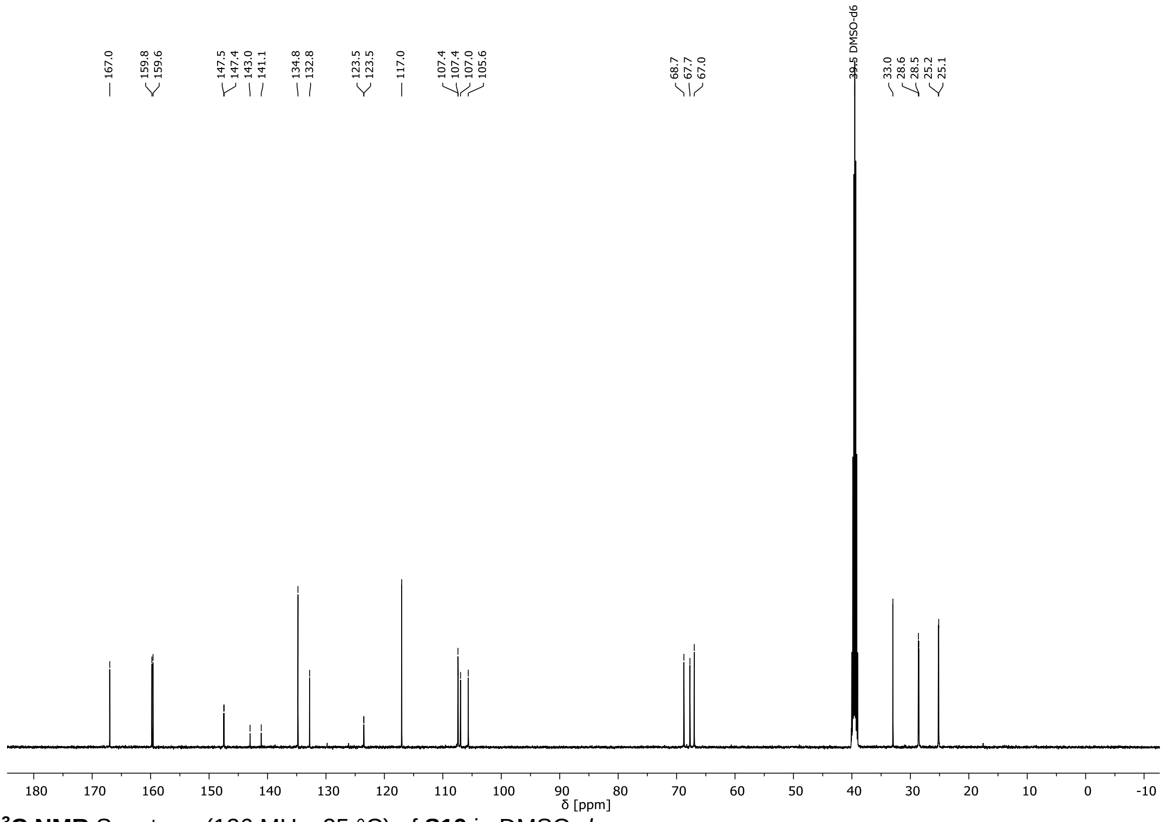

^{13}C NMR Spectrum (126 MHz, 25 °C) of **S19** in DMSO-d_6.

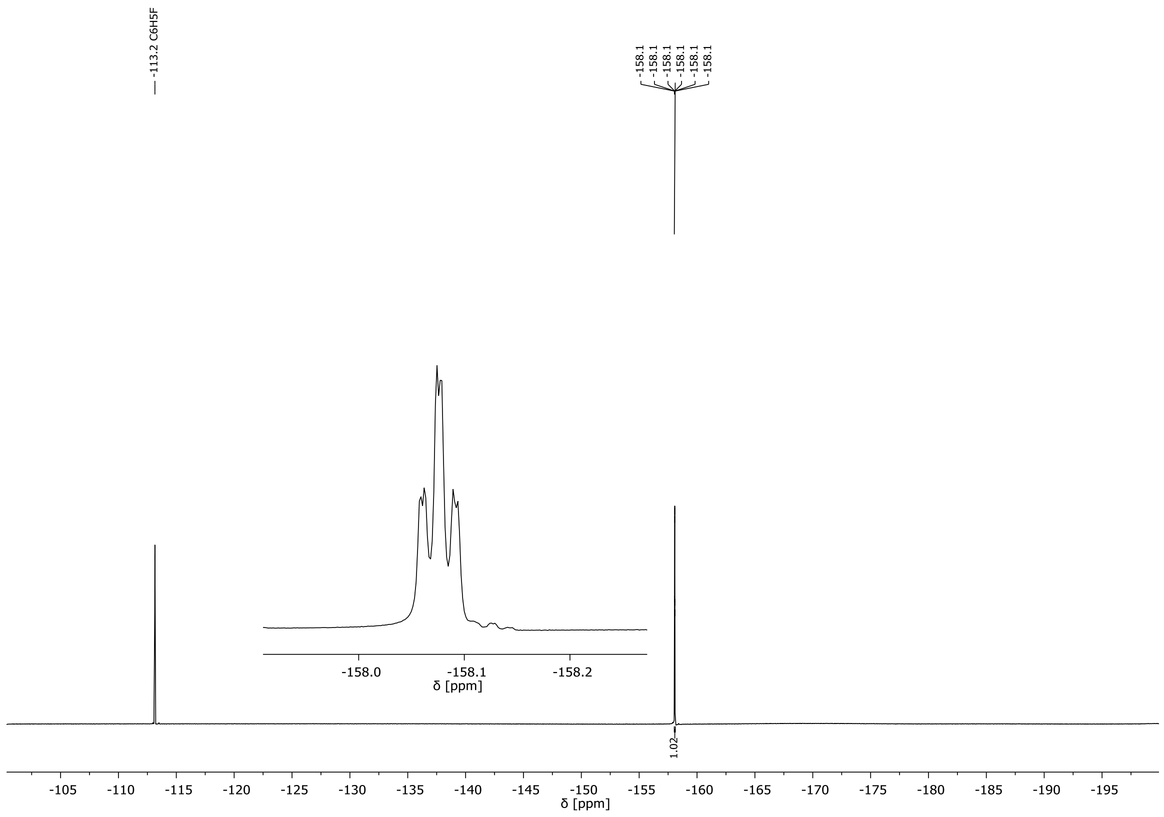

^{19}**F NMR** Spectrum (471 MHz, 25 °C) of **S19** in DMSO-d_6.

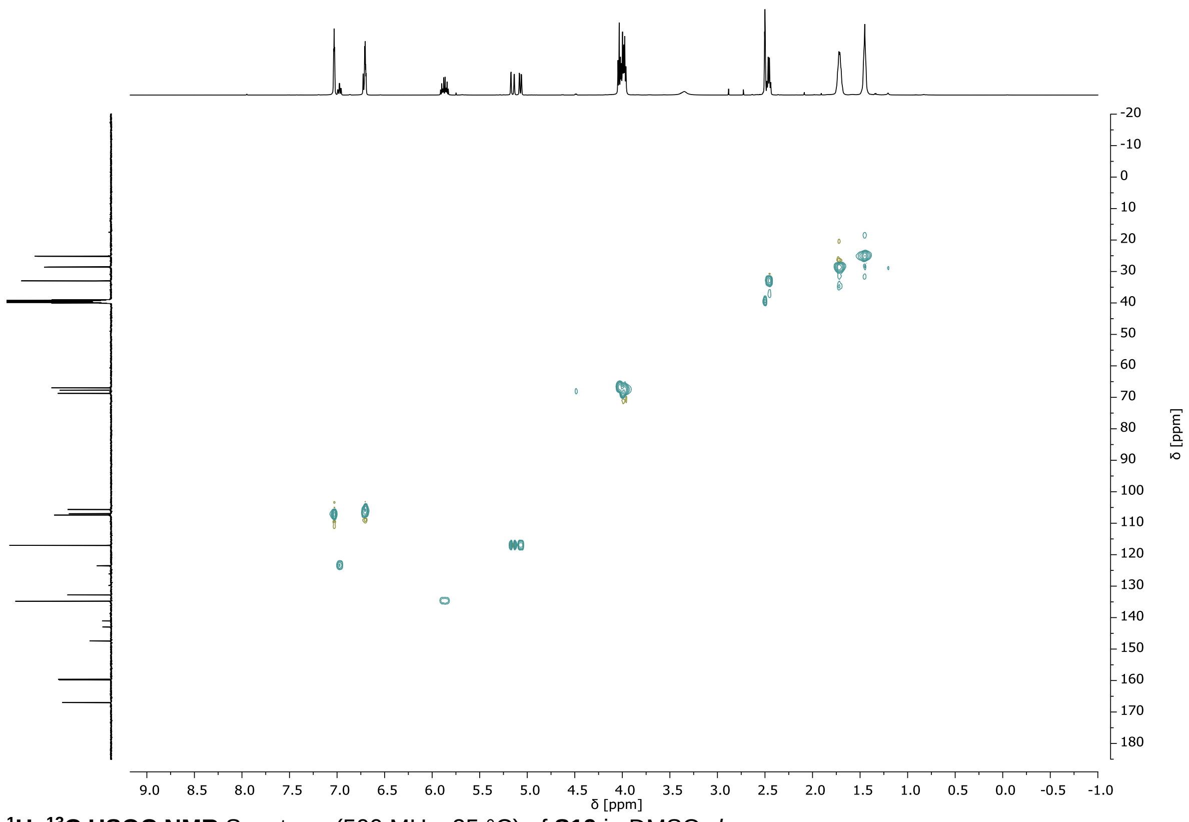

^{1}**H,** ^{13}**C HSQC NMR** Spectrum (500 MHz, 25 °C) of **S19** in DMSO-d_6.

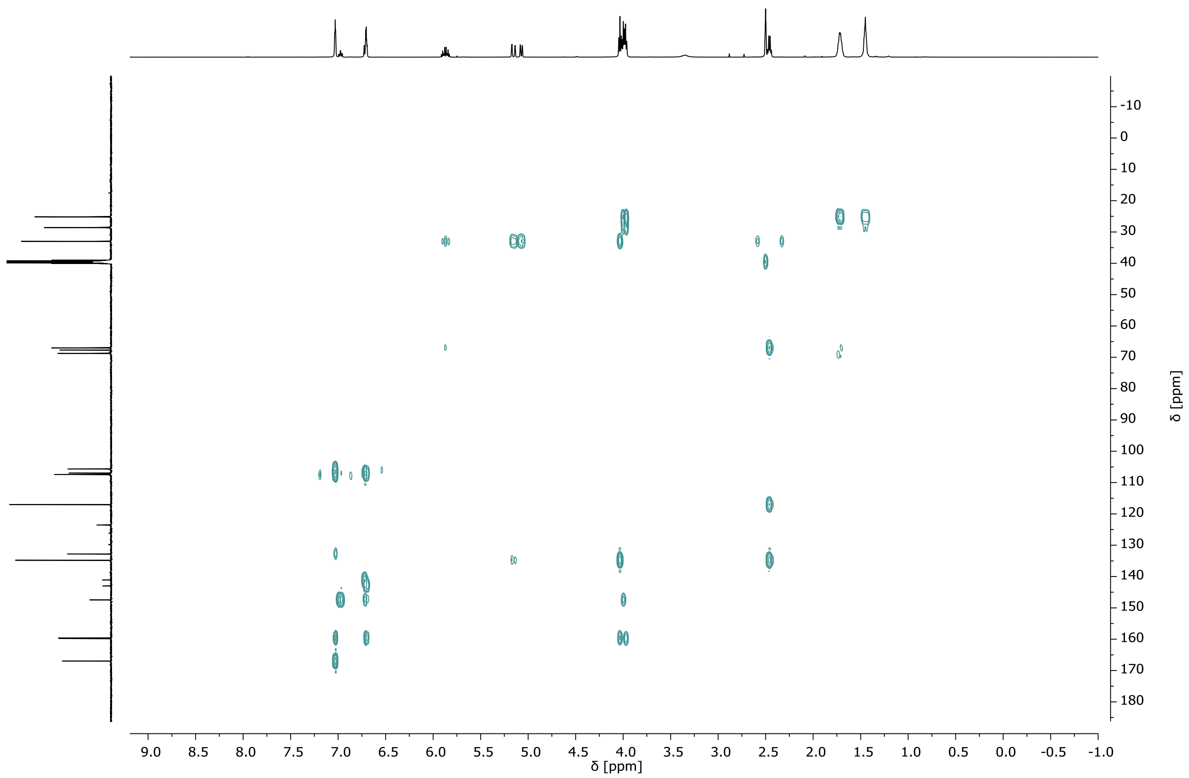

^{1}**H,** ^{13}**C HMBC NMR** Spectrum (500 MHz, 25 °C) of **S19** in DMSO-*d*$_6$.

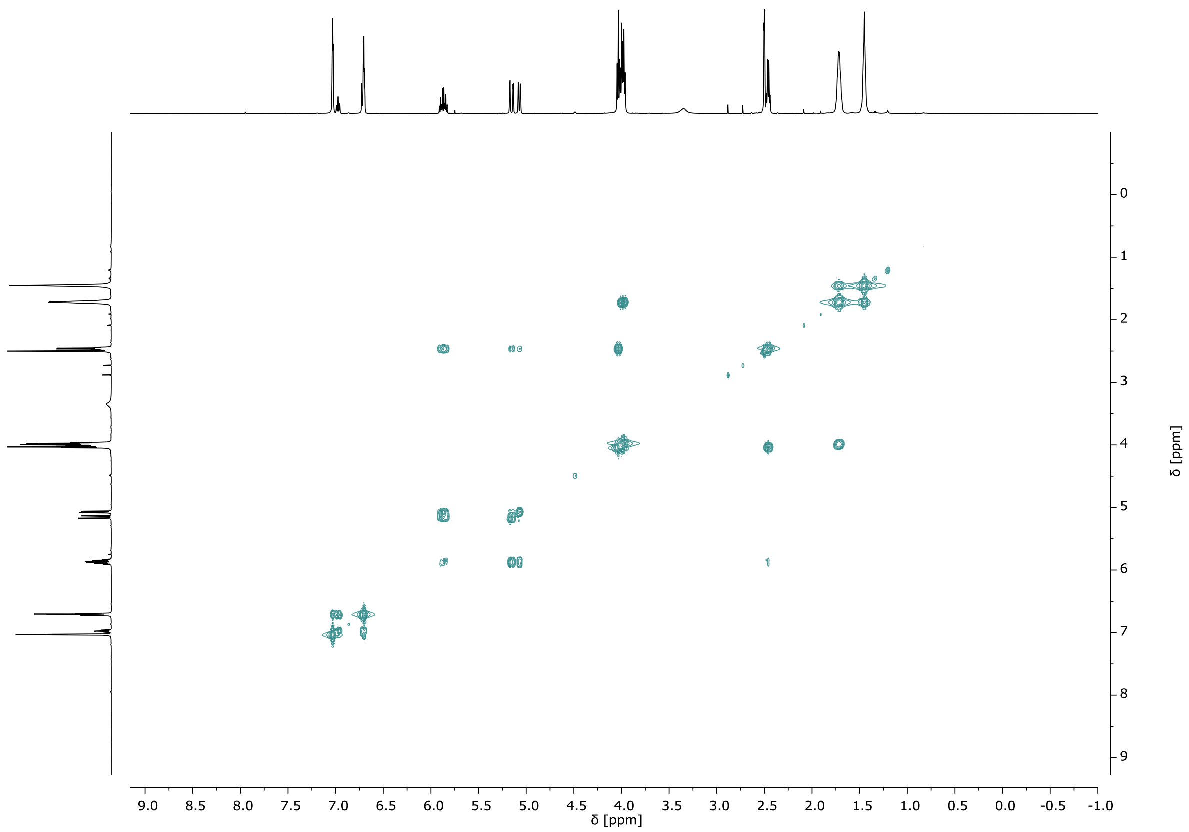

^{1}**H COSY NMR** Spectrum (500 MHz, 25 °C) of **S19** in DMSO-*d*$_6$.

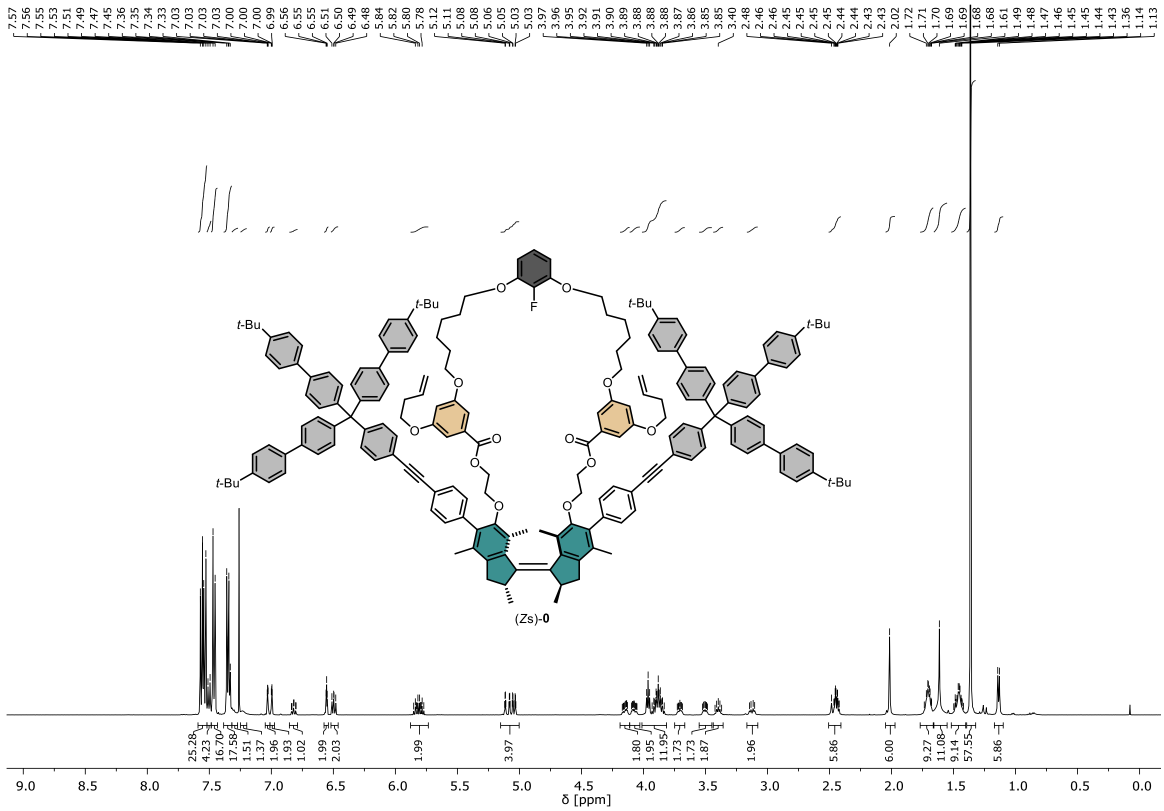

^{1}H NMR Spectrum (500 MHz, 25 °C) of machine (*Z*s)-**0** in $CDCl_3$.

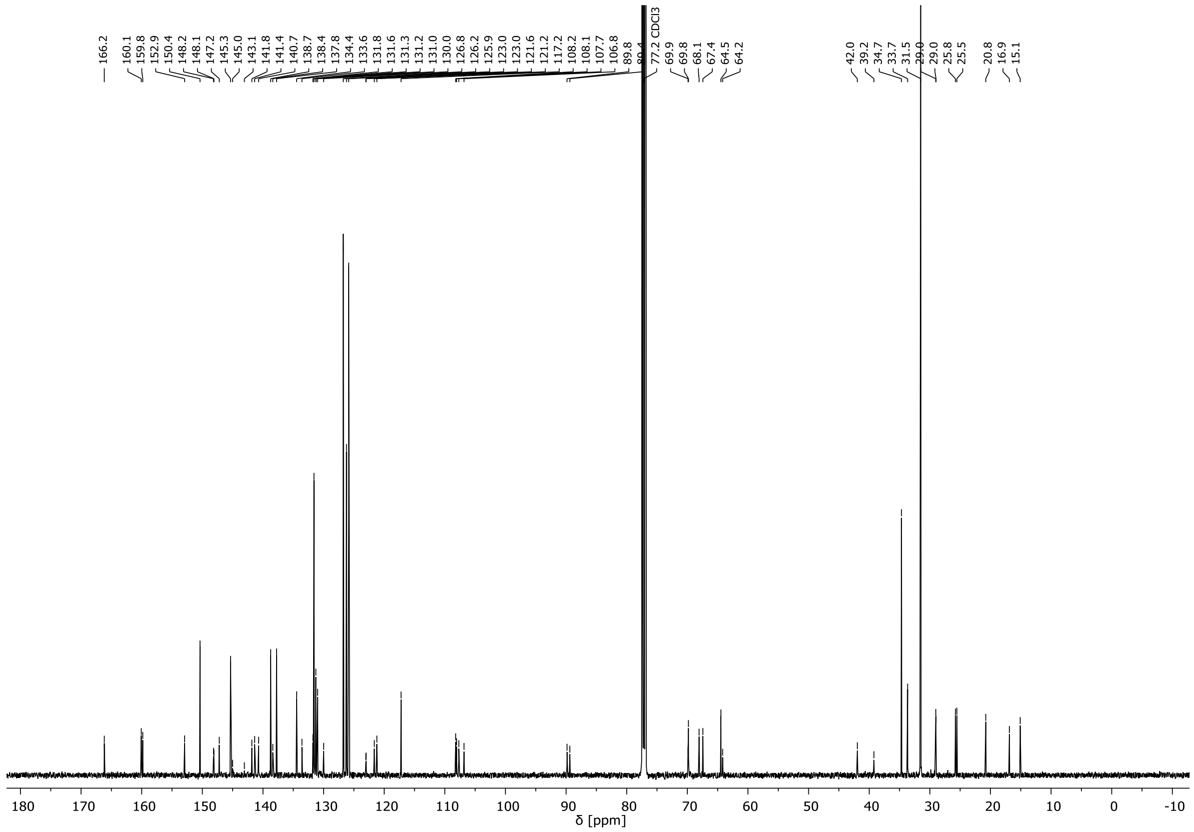

^{13}C NMR Spectrum (126 MHz, 25 °C) of machine (*Z*s)-**0** in $CDCl_3$.

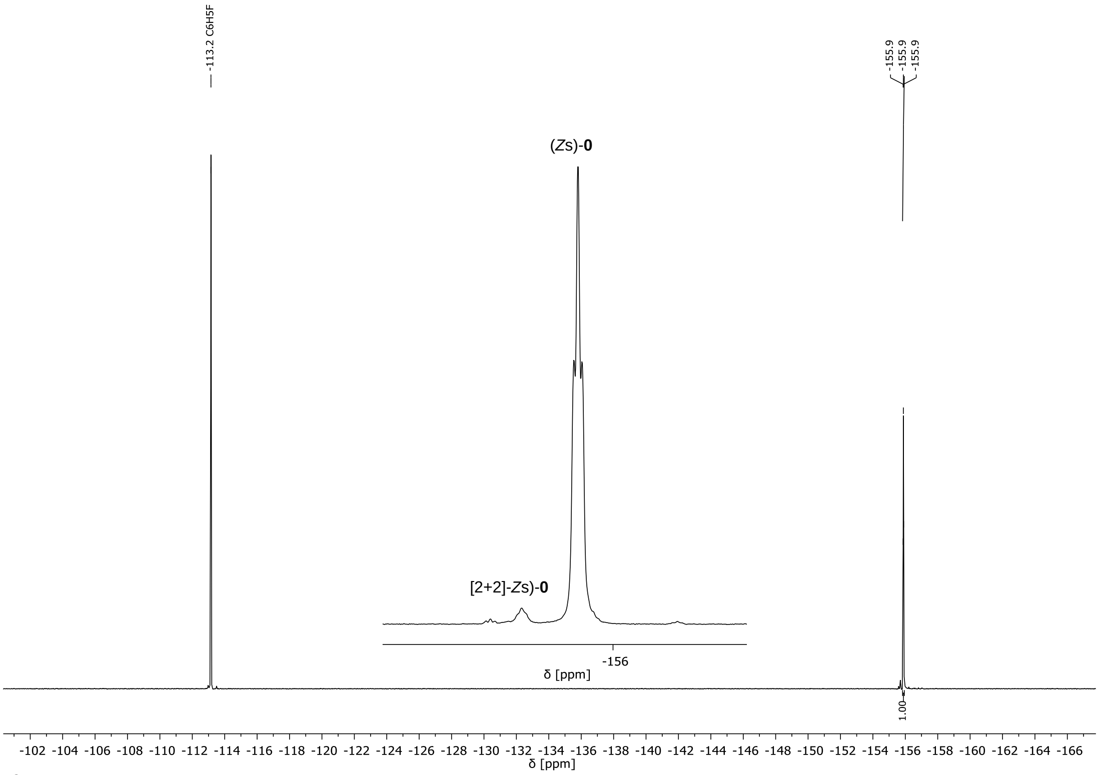

^{19}F NMR Spectrum (471 MHz, 25 °C) of machine (*Z*s)-**0** in $CDCl_3$.

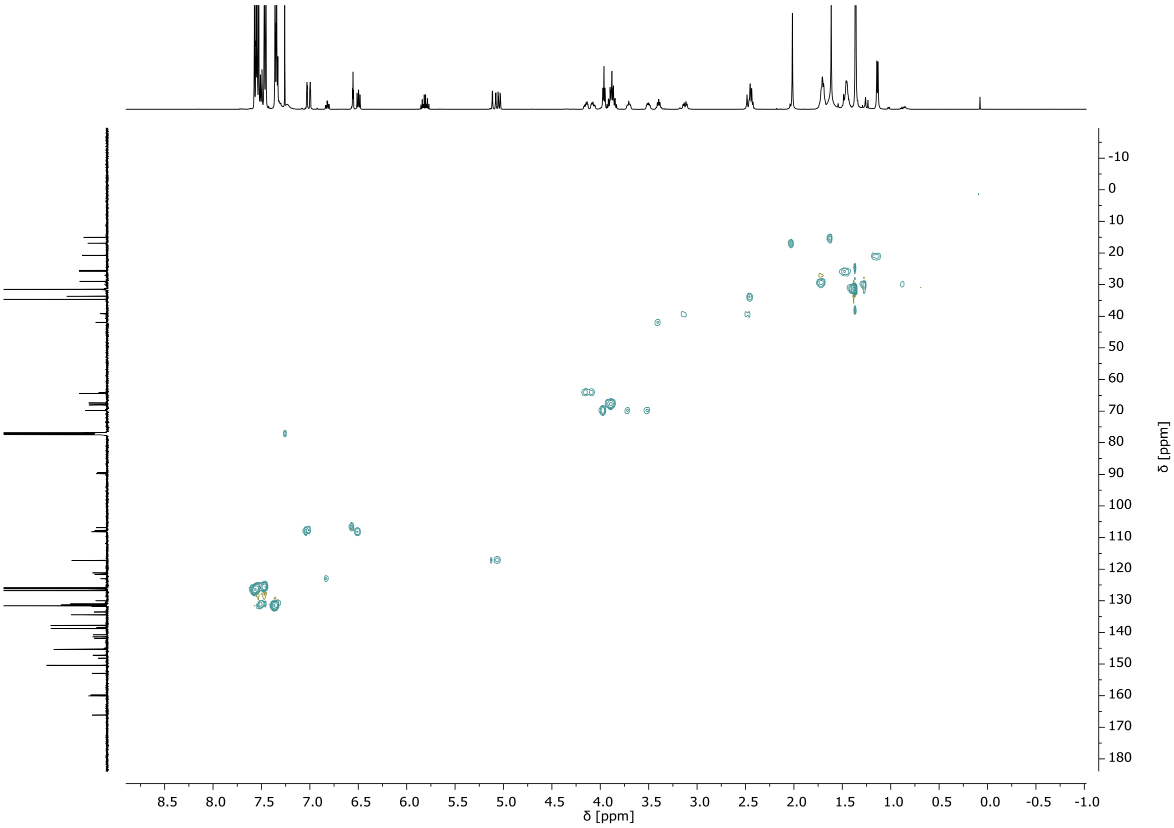

^{1}H, ^{13}C HSQC NMR Spectrum (500 MHz, 25 °C) of machine (*Z*s)-**0** in $CDCl_3$.

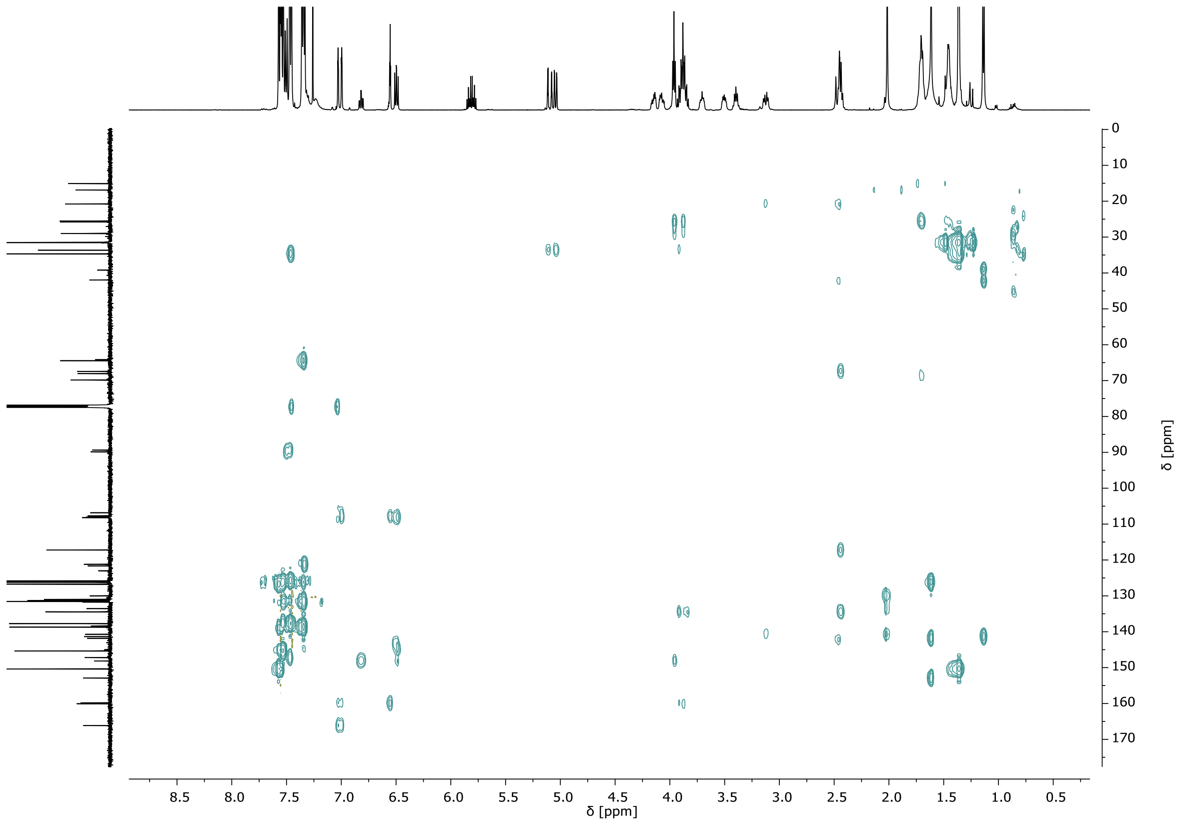

¹H, ¹³C HMBC NMR Spectrum (500 MHz, 25 °C) of machine (*Z*s)-**0** in $CDCl_3$.

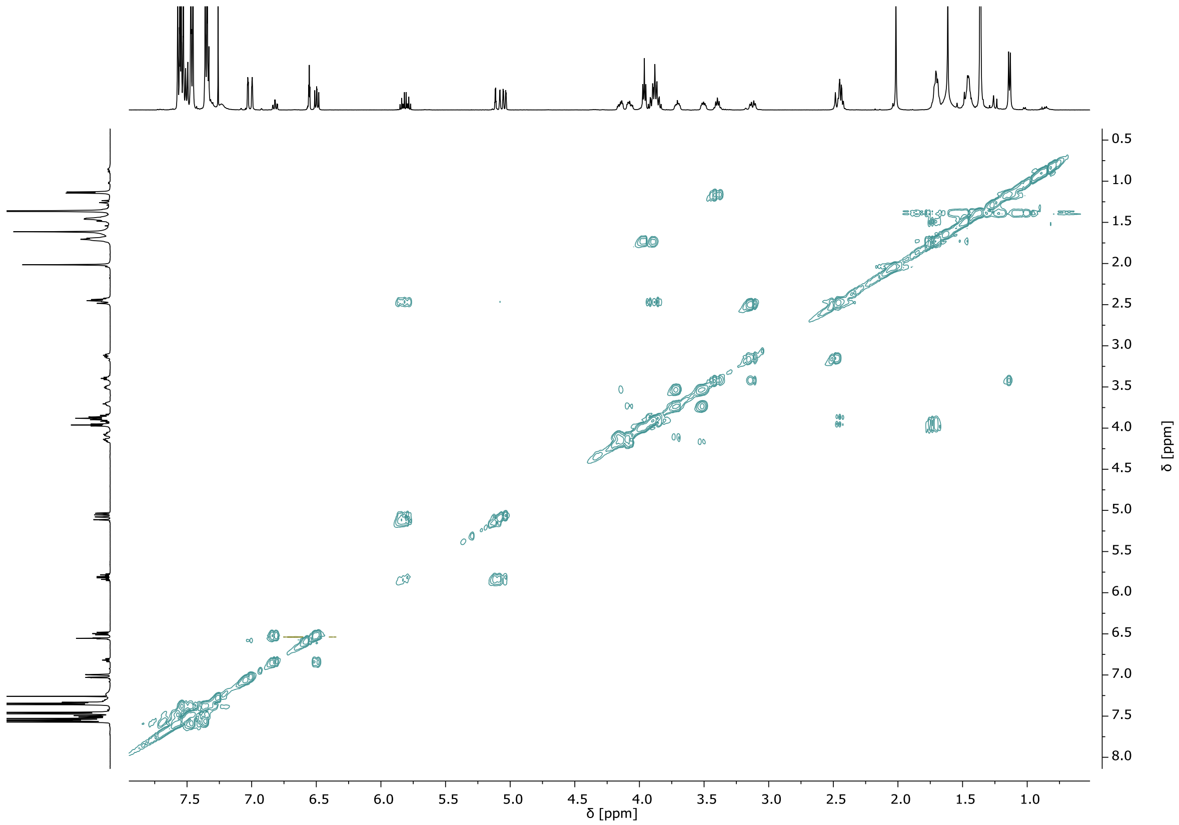

¹H COSY NMR Spectrum (500 MHz, 25 °C) of machine (*Z*s)-**0** in $CDCl_3$.

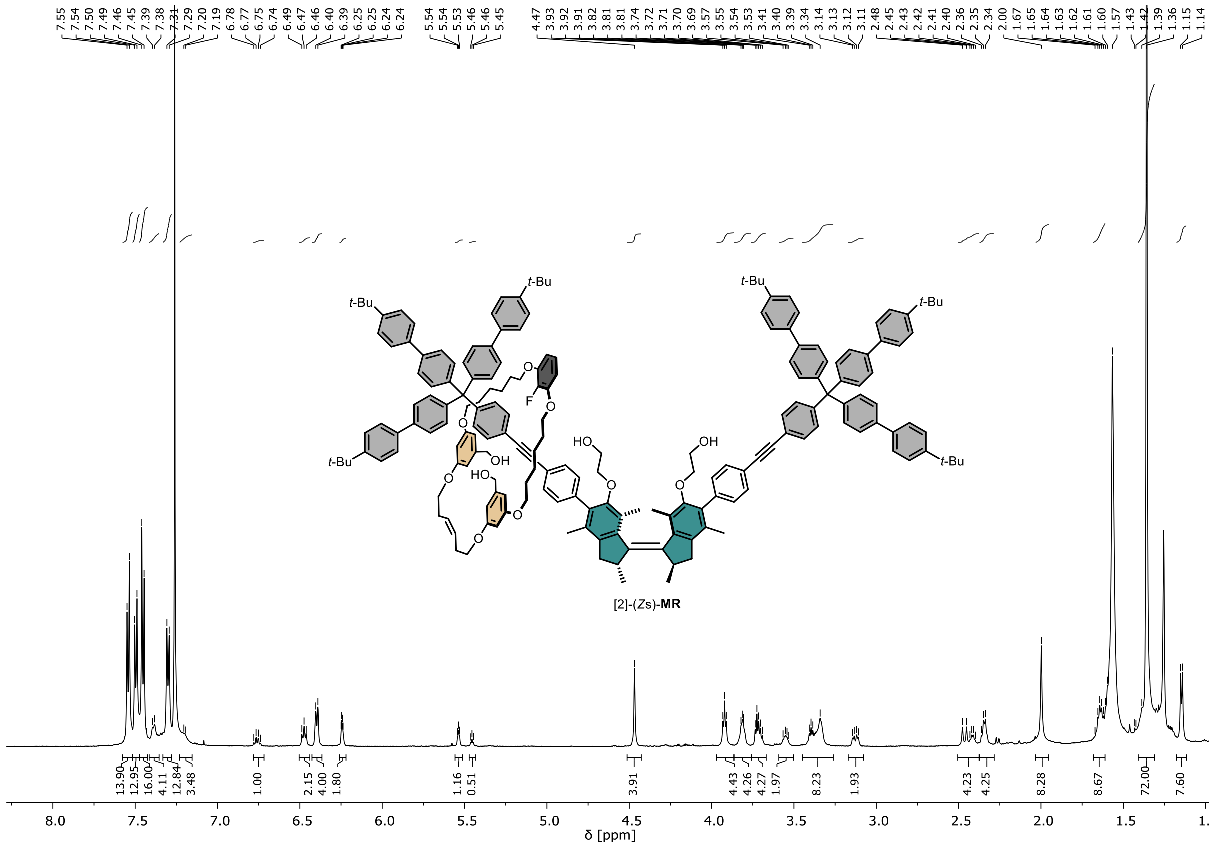

^{1}**H NMR** Spectrum (500 MHz, 25 °C) of motor rotaxane [2]-(*Z*s)-**MR** in $CDCl_3$.

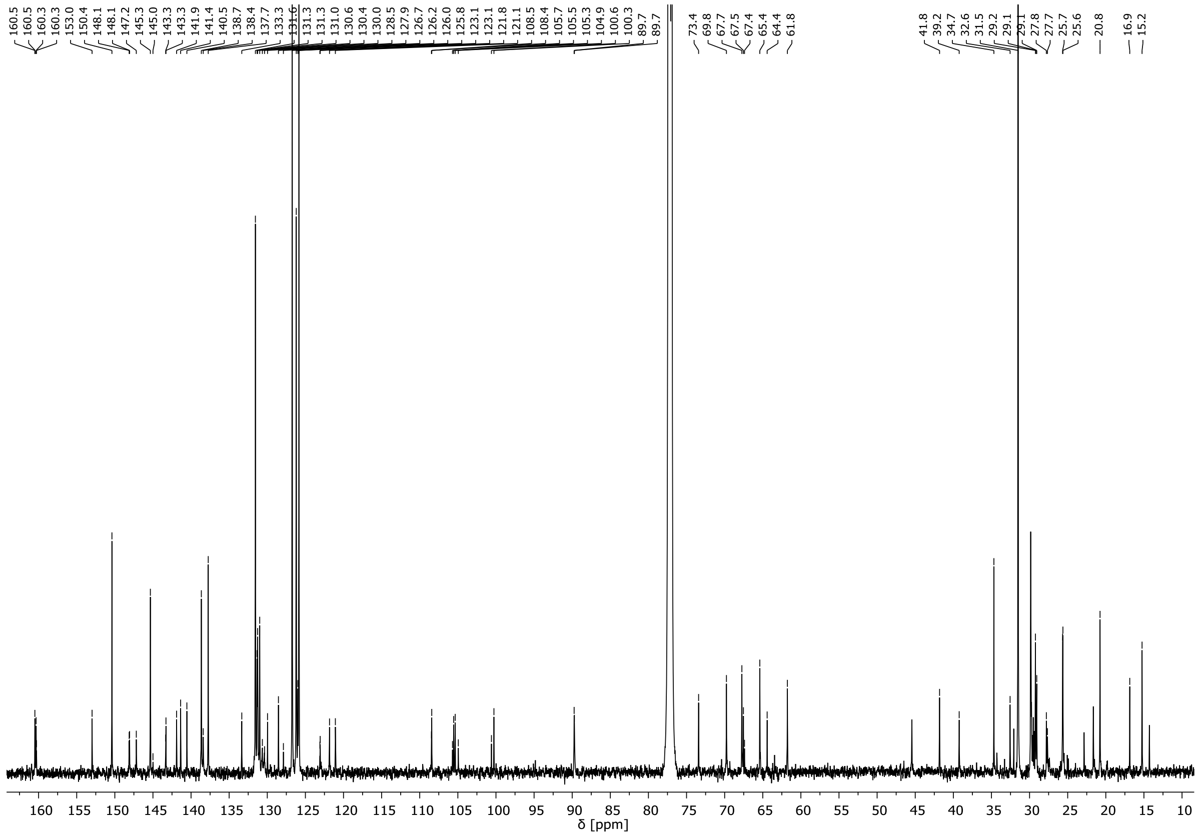

^{13}**C NMR** Spectrum (151 MHz, 25 °C) of motor rotaxane [2]-(*Z*s)-**MR** in $CDCl_3$.

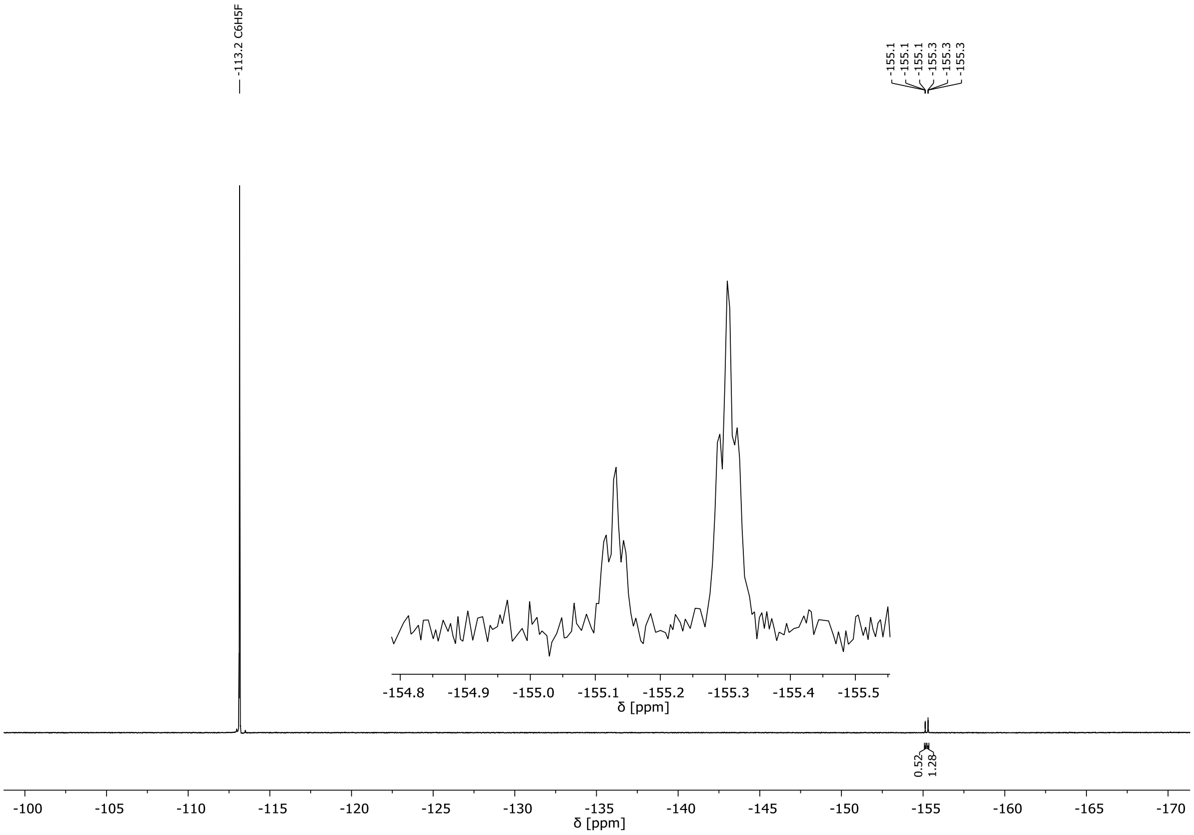

^{19}F NMR Spectrum (471 MHz, 25 °C) of motor rotaxane [2]-(Zs)-**MR** in $CDCl_3$.

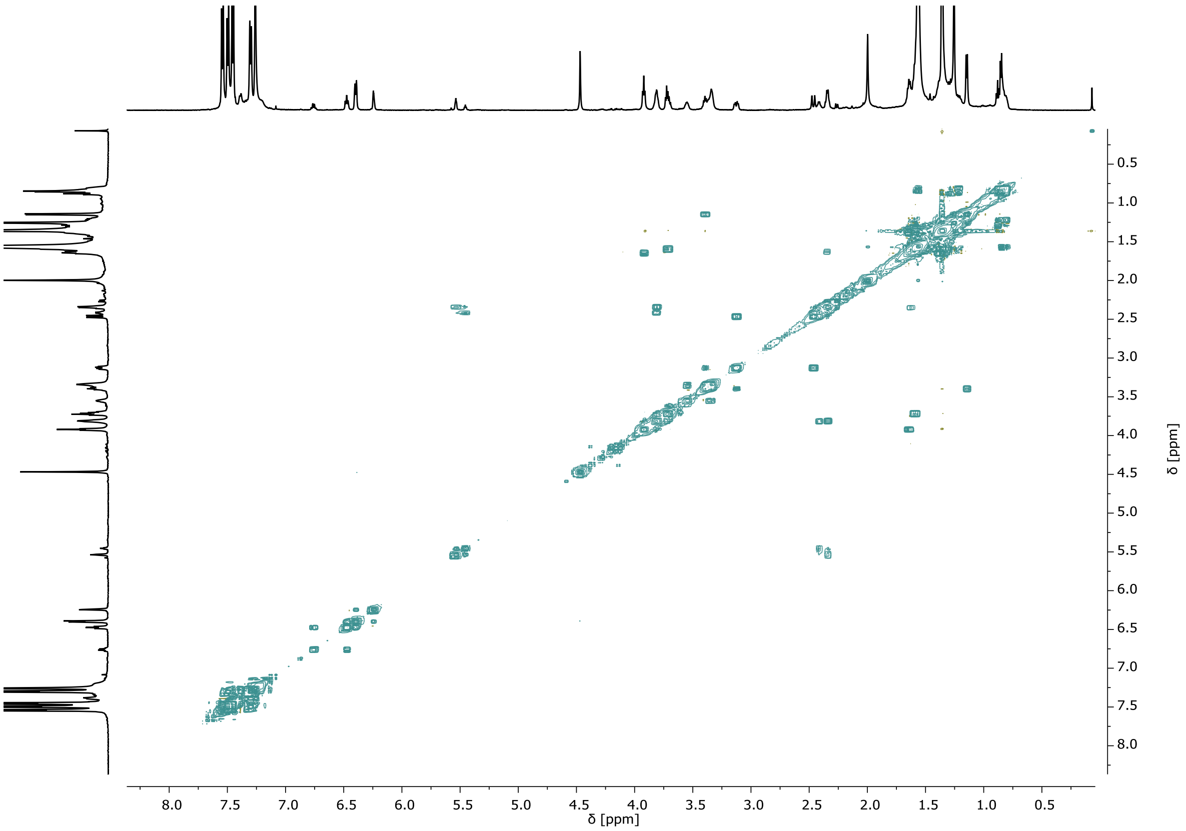

^{1}H COSY NMR Spectrum (500 MHz, 25 °C) of motor rotaxane [2]-(*Z*s)-**MR** in $CDCl_3$.

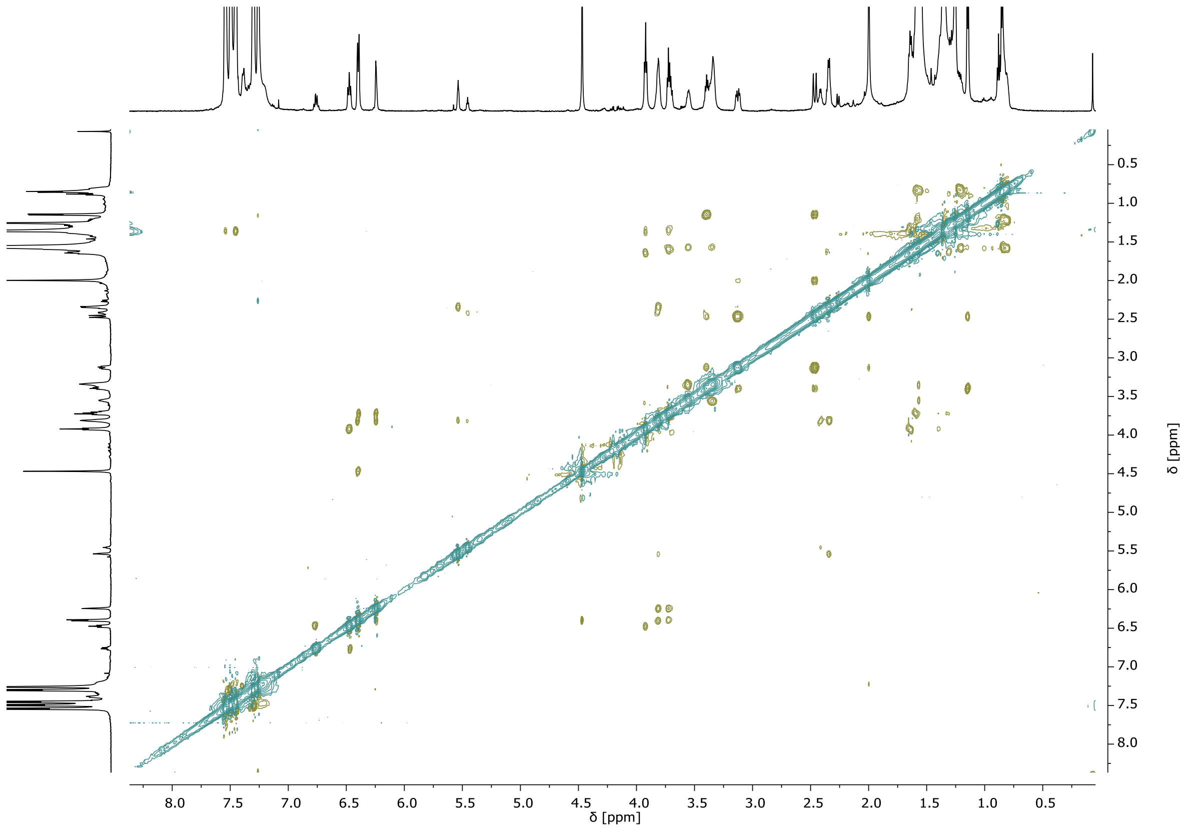

^{1}**H ROESY NMR** Spectrum (600 MHz, 25 °C) of motor rotaxane [2]-(*Z*s)-**MR** in $CDCl_3$.

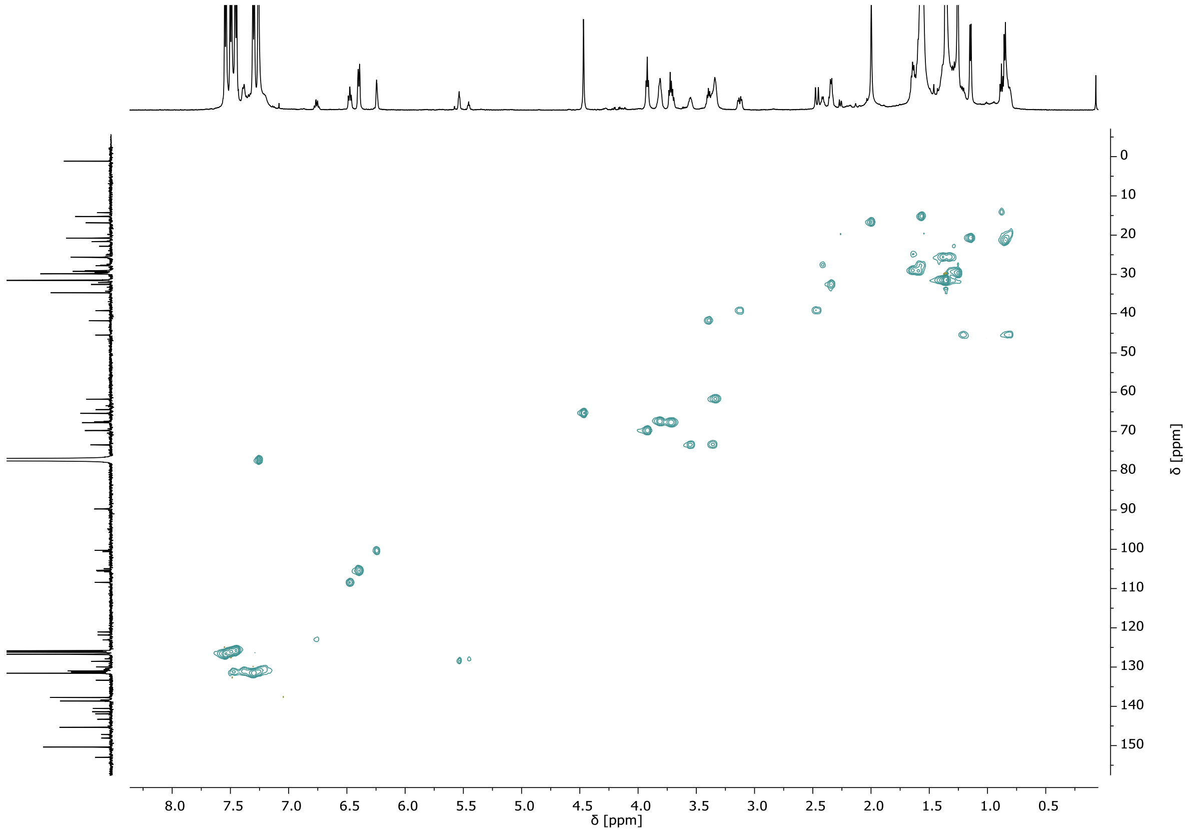

^{1}**H,** ^{13}**C HSQC NMR** Spectrum (600 MHz, 25 °C) of motor rotaxane [2]-(*Z*s)-**MR** in $CDCl_3$.

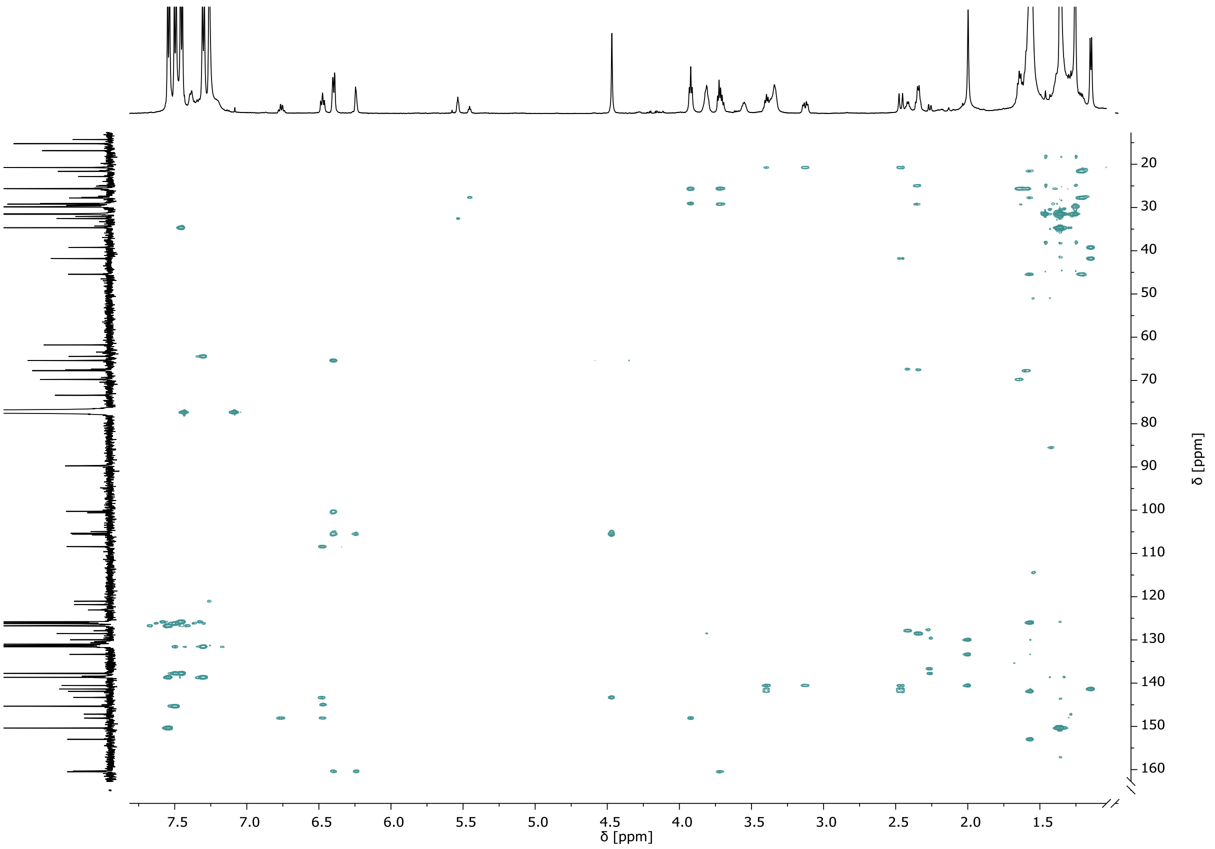

^{1}H, ^{13}C HMBC NMR Spectrum (600 MHz, 25 °C) of motor rotaxane [2]-(*Z*s)-**MR** in $CDCl_3$.

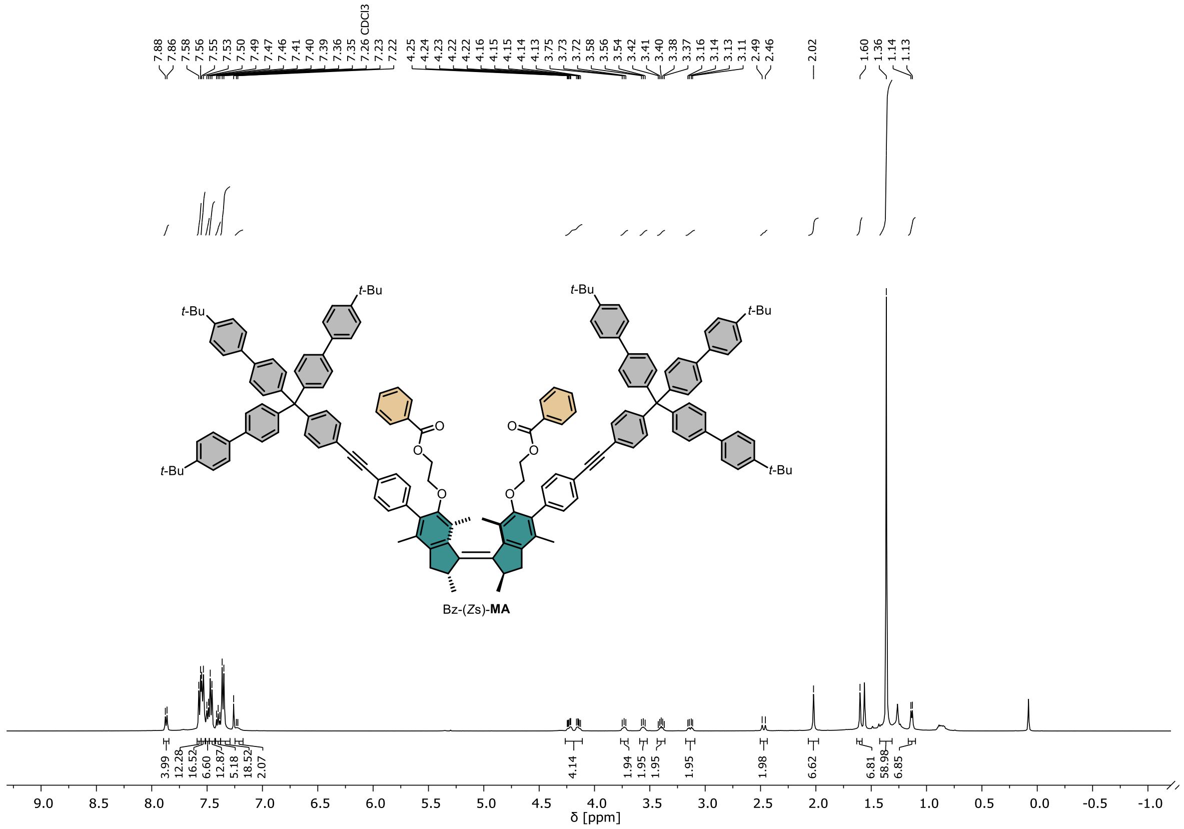

^{1}H NMR Spectrum (500 MHz, 25 °C) of axle Bz-(*Z*s)-**MA** in CDCl3.

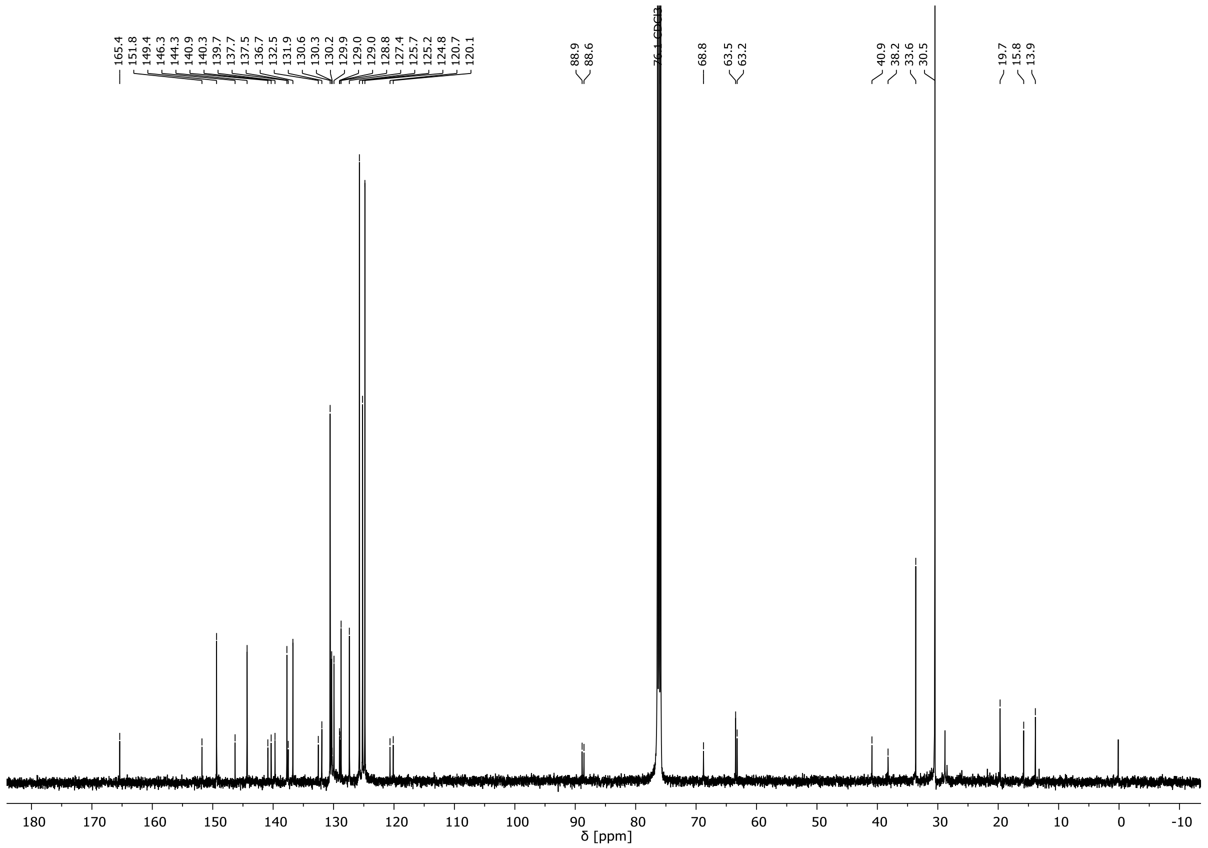

^{13}C NMR Spectrum (126 MHz, 25 °C) of axle Bz-(*Z*s)-**MA** in CDCl3.

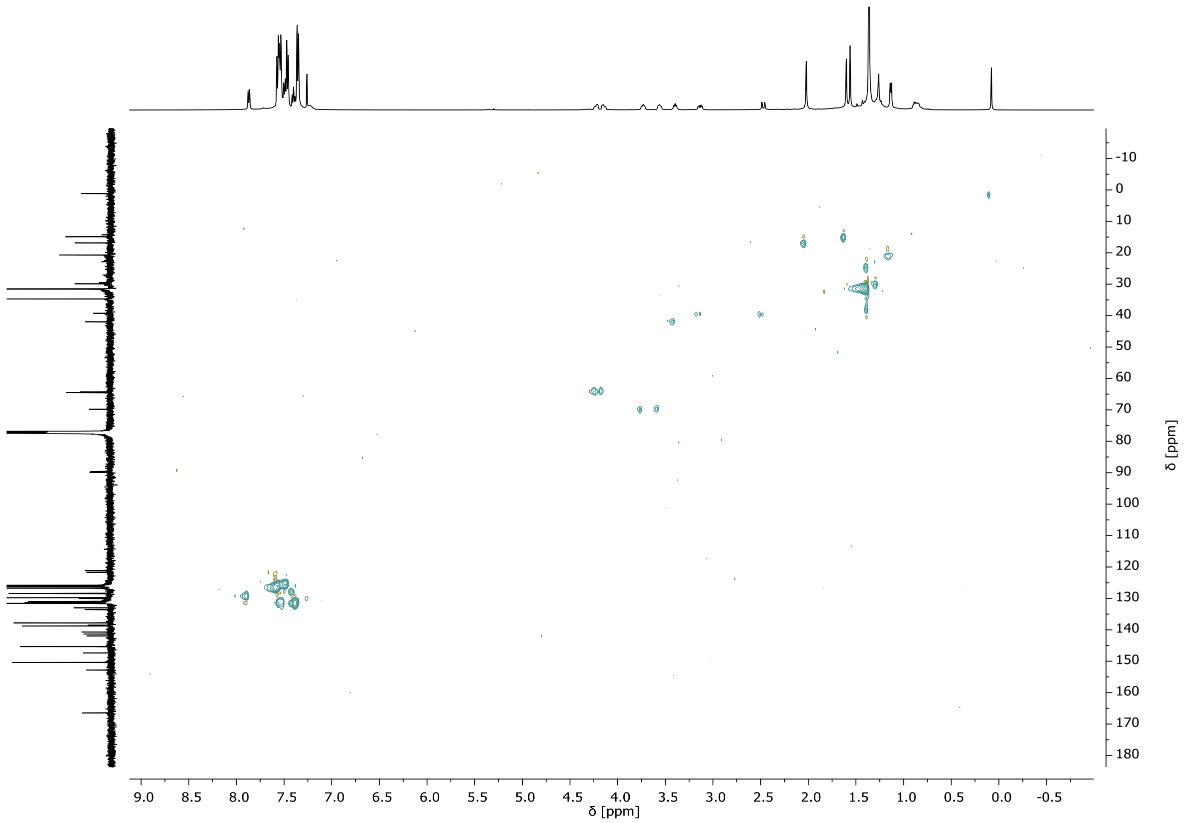

^{1}H, ^{13}C HSQC NMR Spectrum (500 MHz, 25 °C) of axle Bz-(*Z*s)-**MA** in $CDCl_3$.

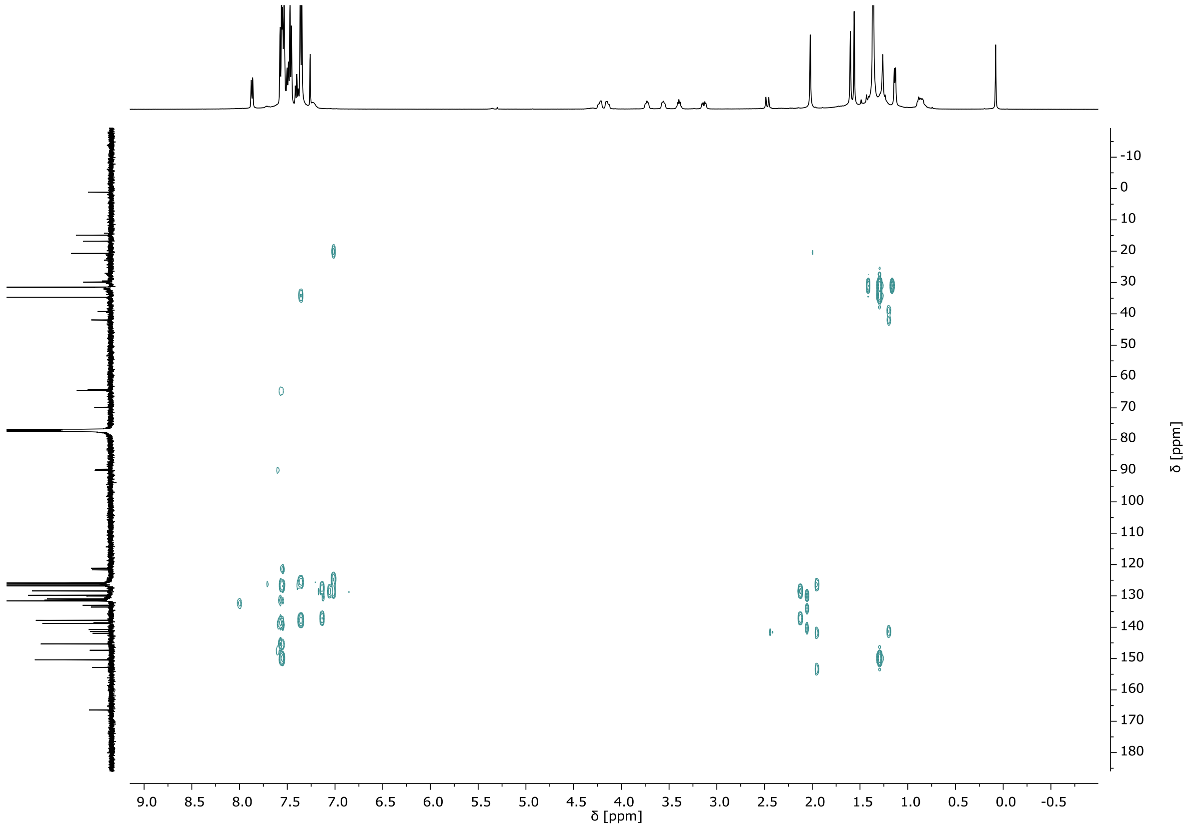

^{1}H, ^{13}C HMBC NMR Spectrum (500 MHz, 25 °C) of axle Bz-(*Z*s)-**MA** in $CDCl_3$.

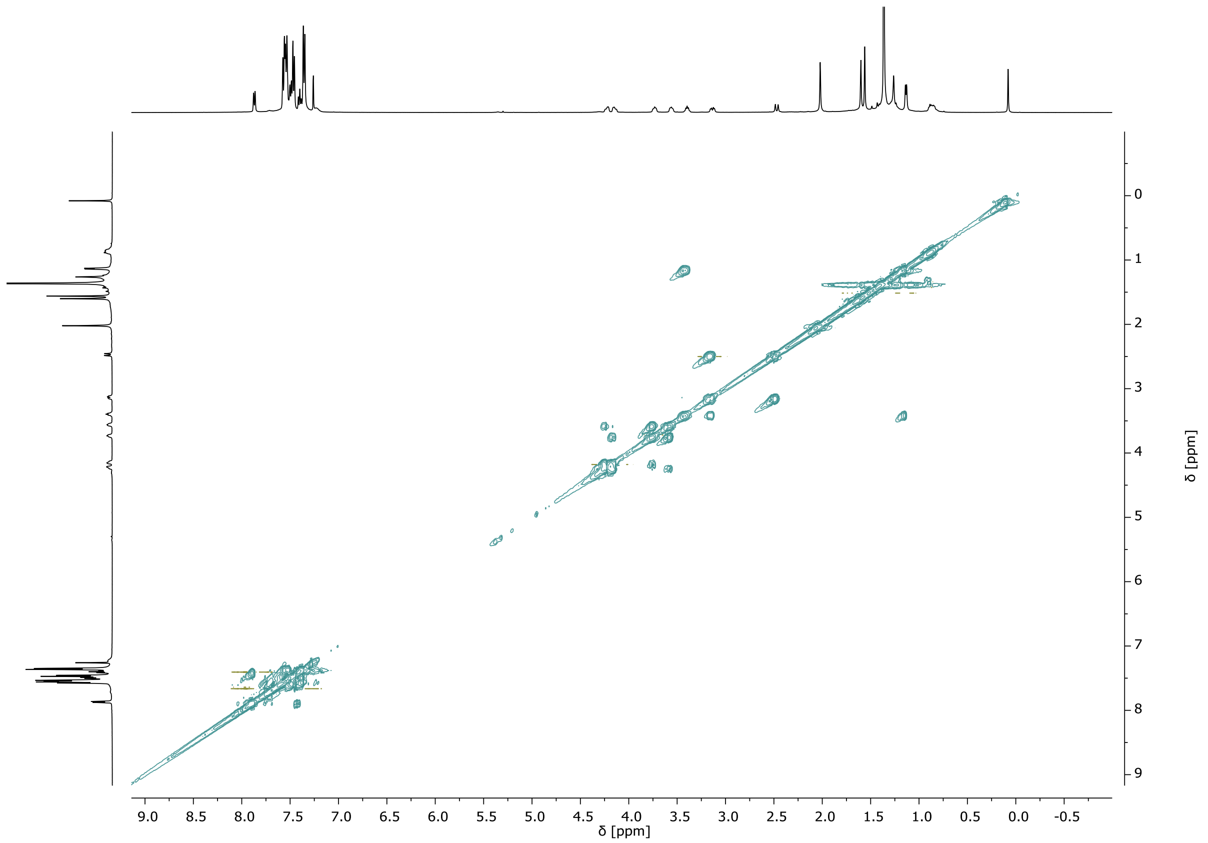

^{1}**H COSY NMR** Spectrum (500 MHz, 25 °C) of axle Bz-(*Z*s)-**MA** in $CDCl_3$.

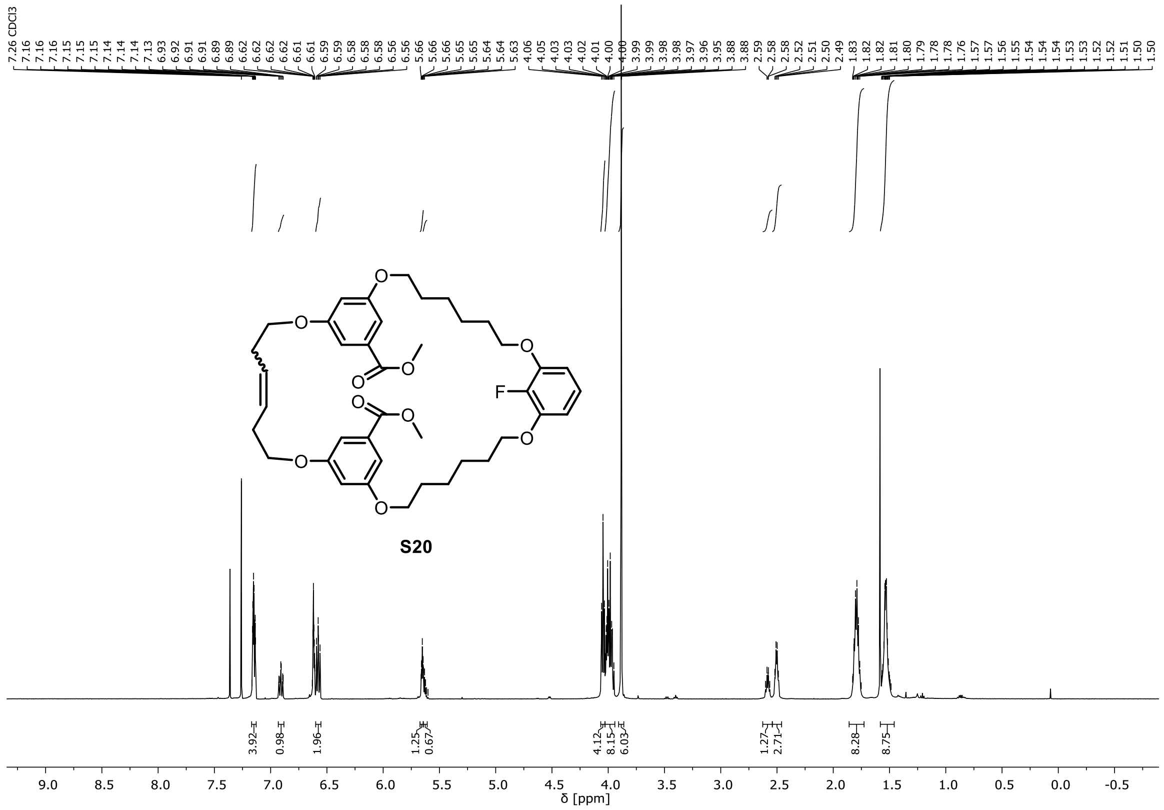

^{1}H NMR Spectrum (500 MHz, 25 °C) of **S20** in $CDCl_3$.

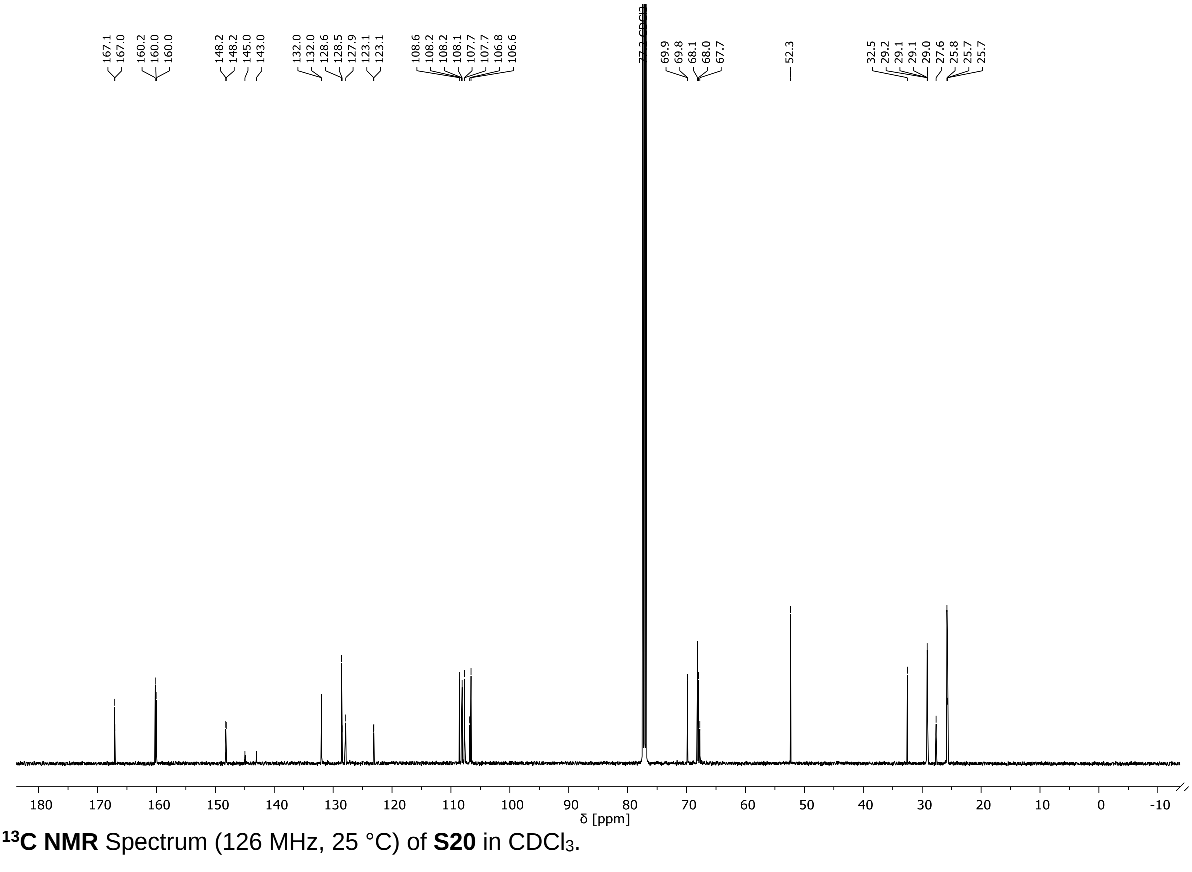

^{13}C NMR Spectrum (126 MHz, 25 °C) of **S20** in $CDCl_3$.

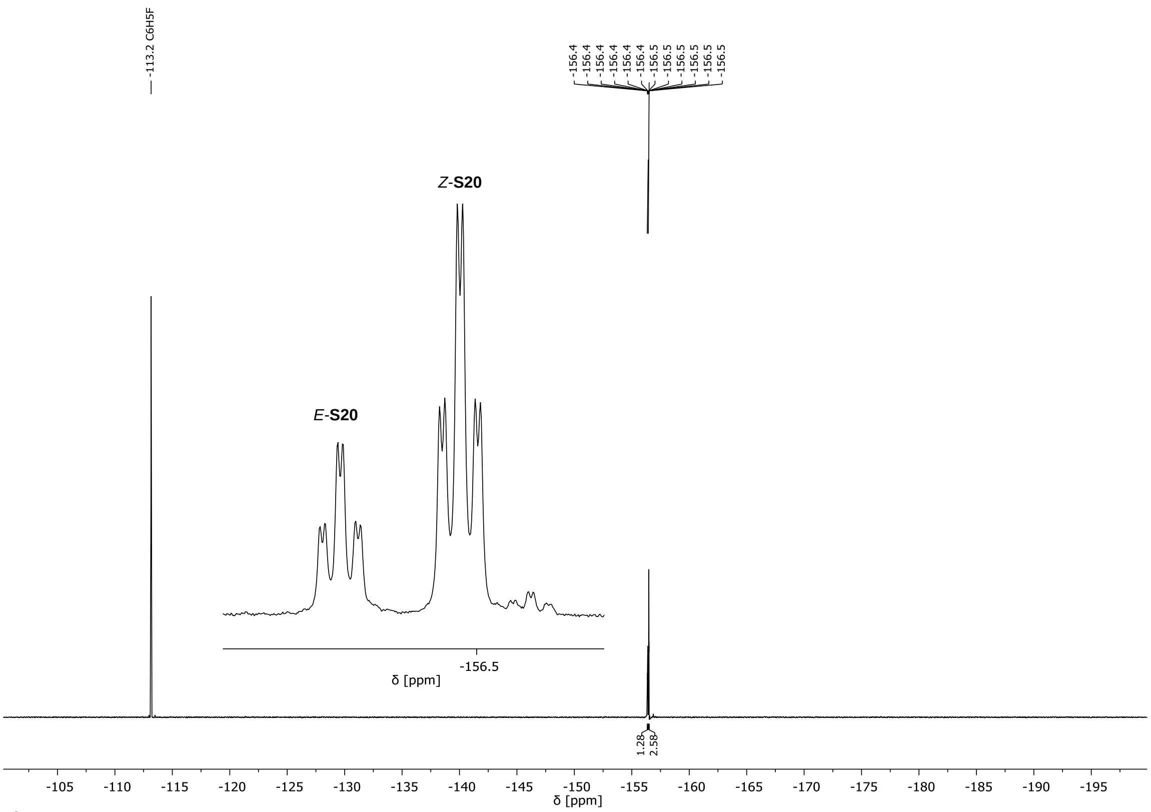

^{19}F NMR Spectrum (471 MHz, 25 °C) of **S20** in $CDCl_3$.

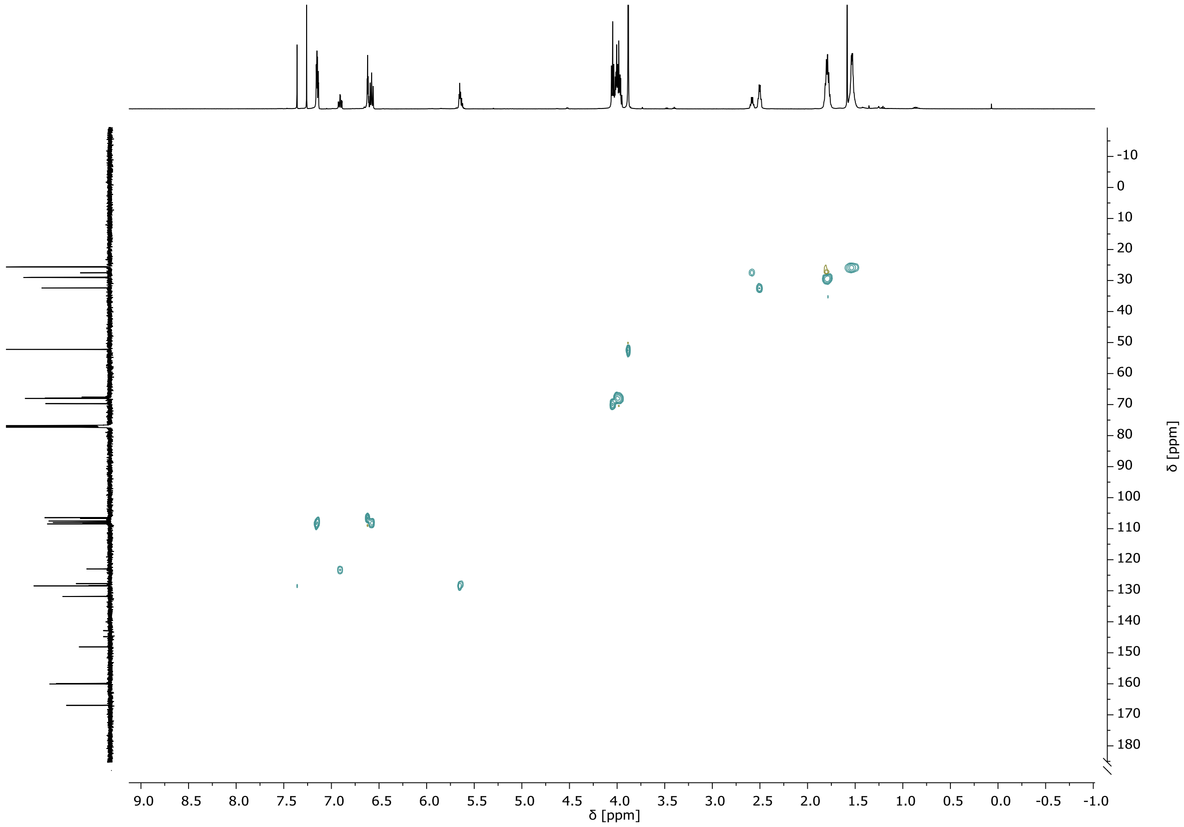

^{1}H, ^{13}C HSQC NMR Spectrum (500 MHz, 25 °C) of **S20** in $CDCl_3$.

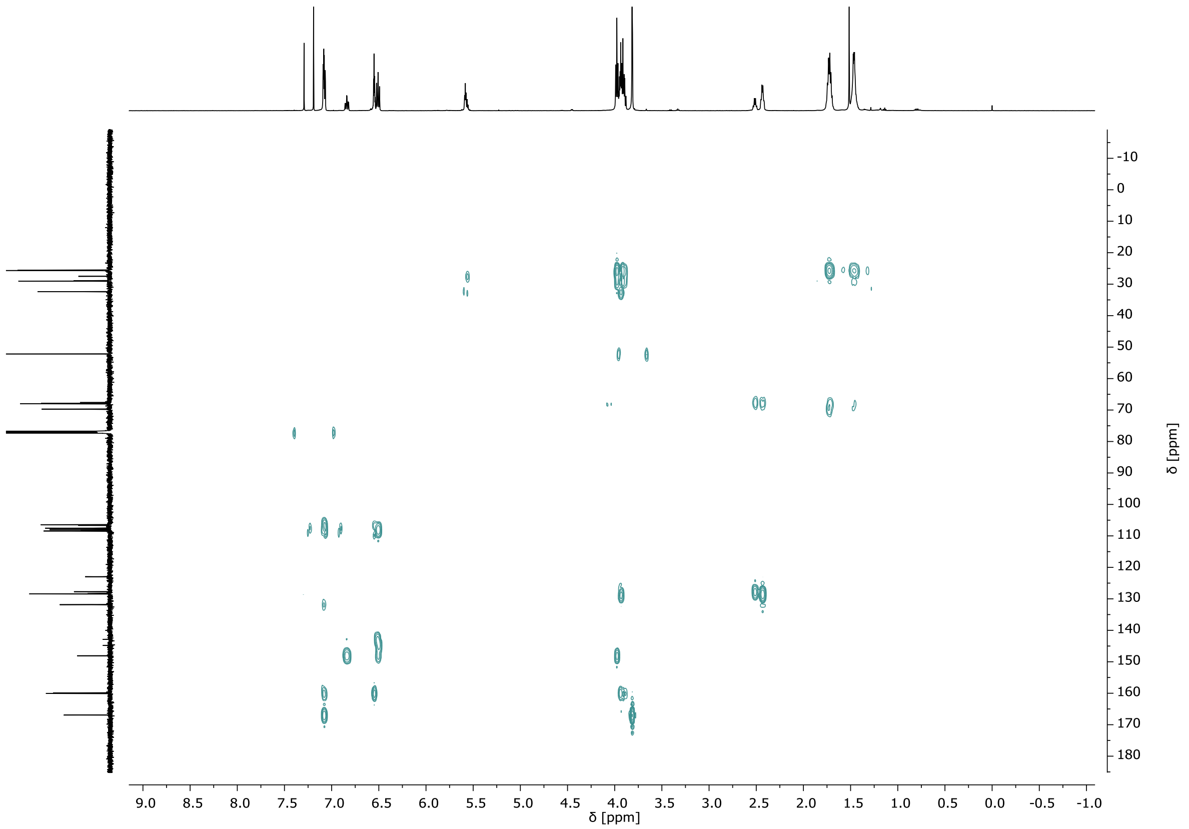

^{1}H, ^{13}C HMBC NMR Spectrum (500 MHz, 25 °C) of **S20** in $CDCl_3$.

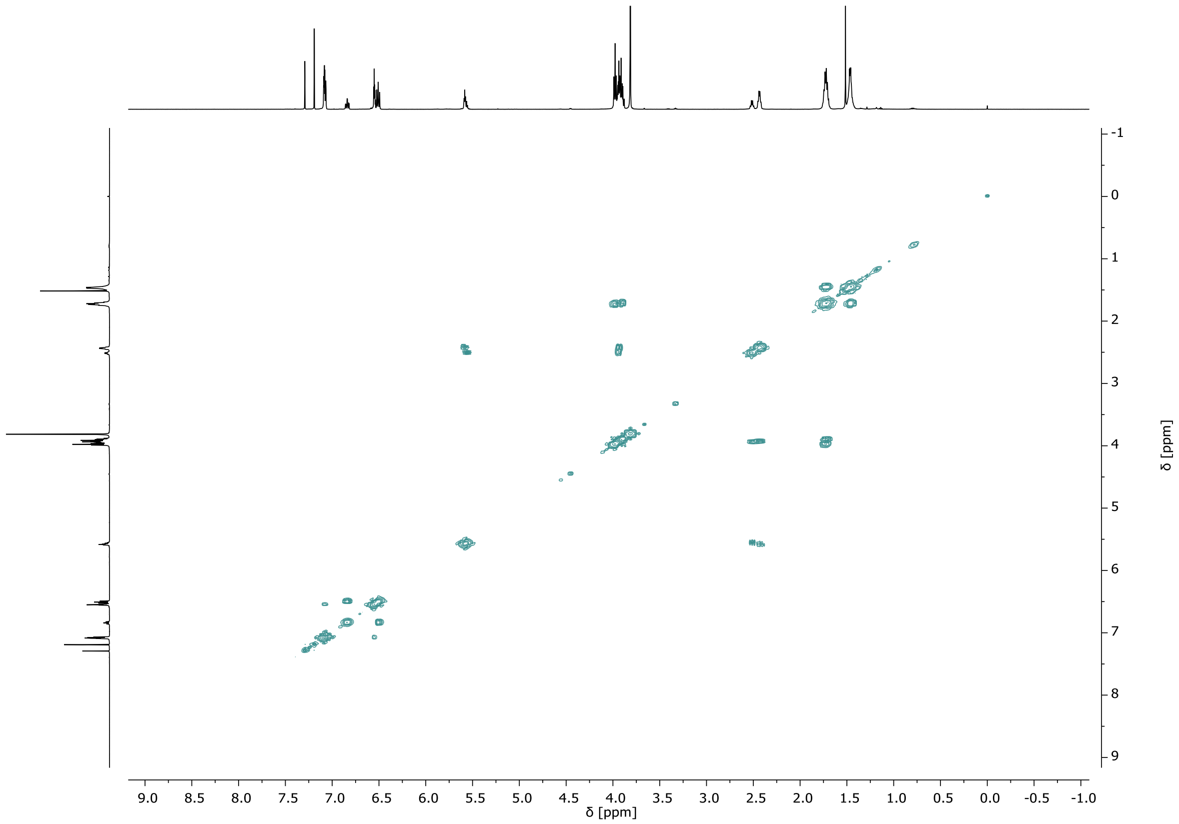

^{1}H COSY NMR Spectrum (500 MHz, 25 °C) of **S20** in $CDCl_3$.

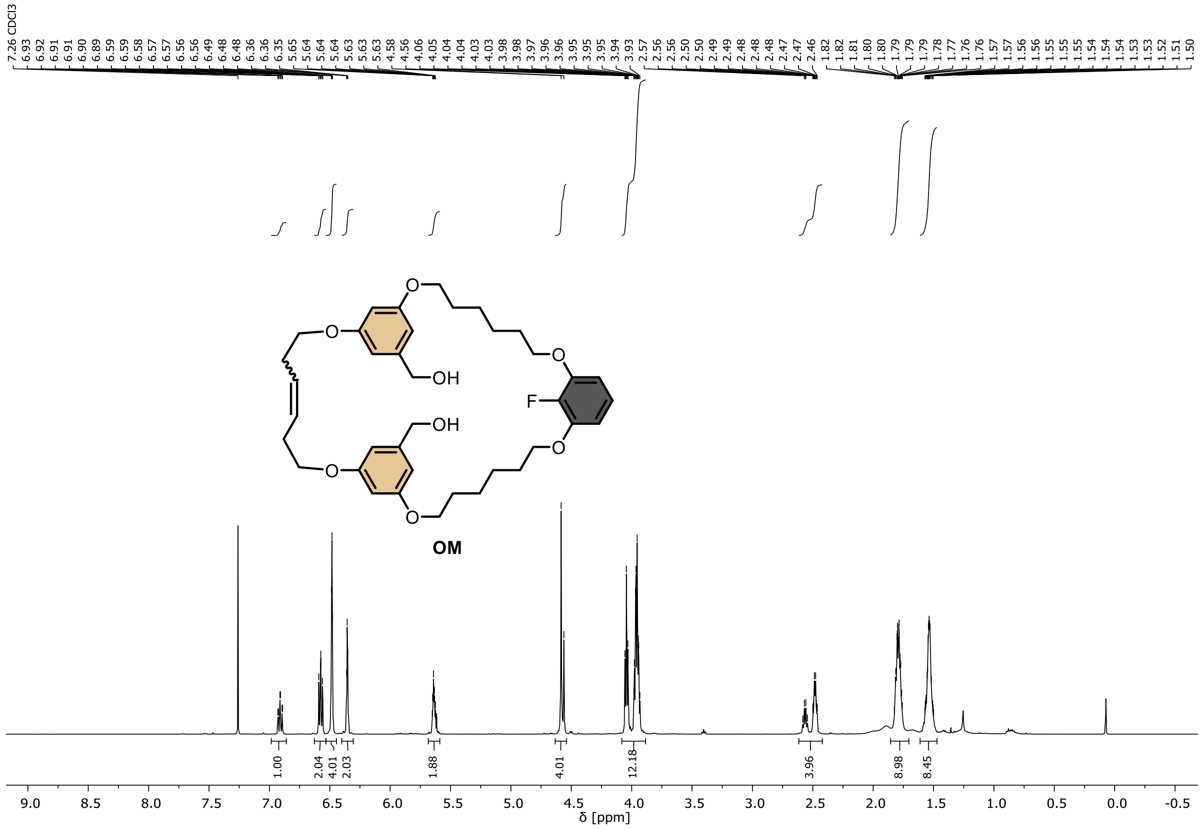

^{1}H NMR Spectrum (500 MHz, 25 °C) of olefin macrocycle **OM** in $CDCl_3$.

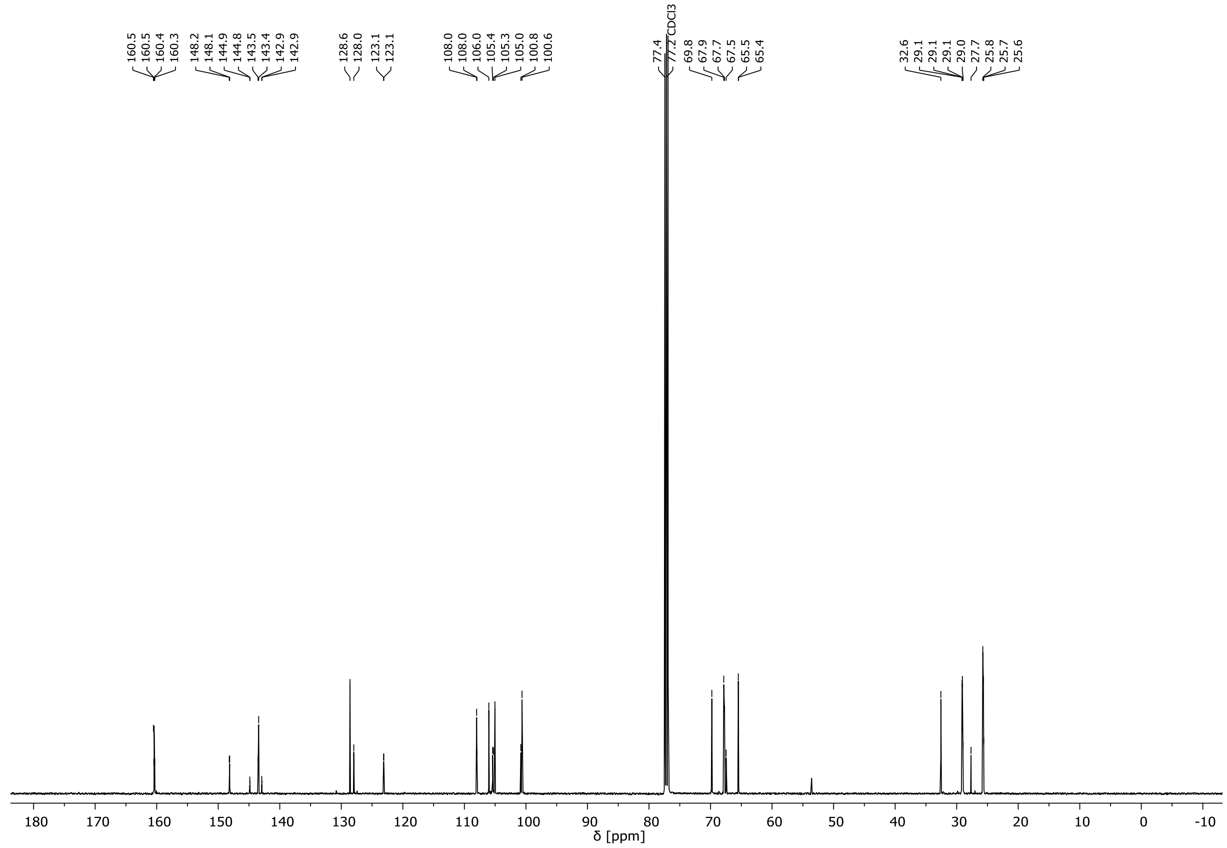

^{13}C NMR Spectrum (126 MHz, 25 °C) of olefin macrocycle **OM** in $CDCl_3$.

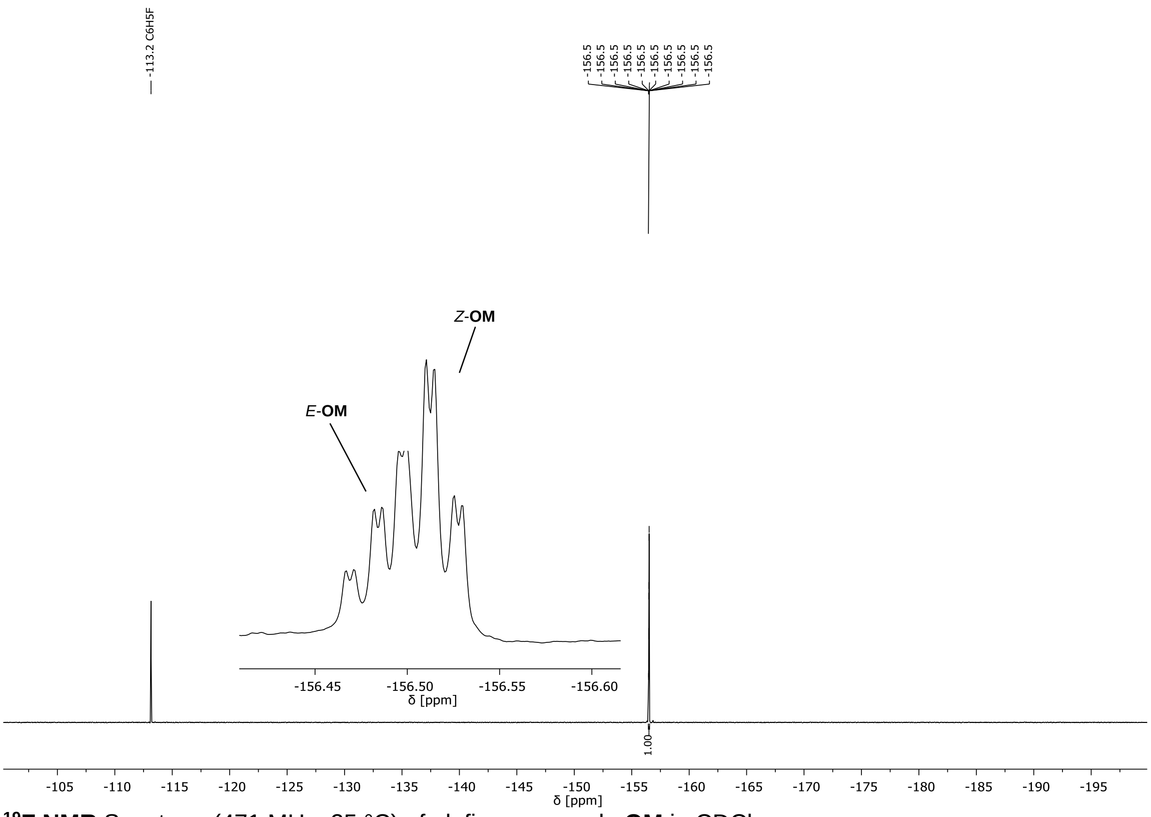

^{19}F NMR Spectrum (471 MHz, 25 °C) of olefin macrocycle **OM** in $CDCl_3$.

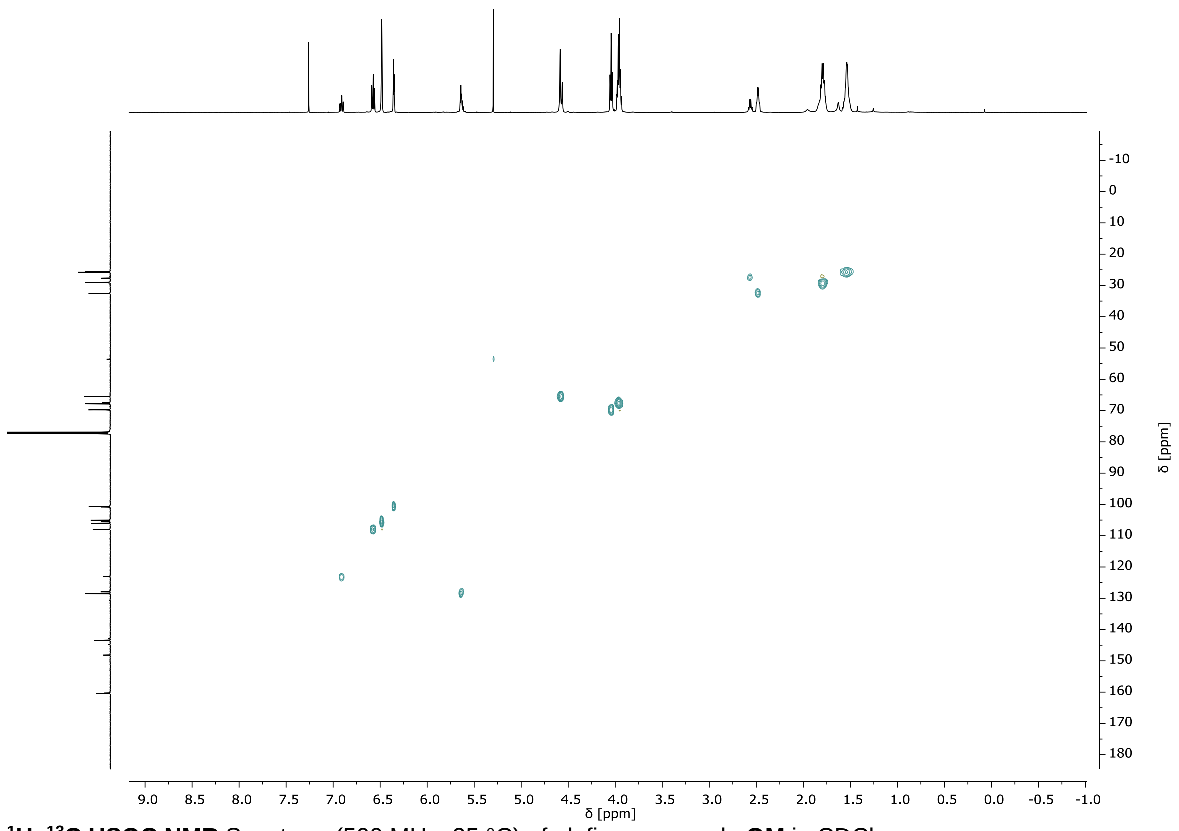

^{1}H, ^{13}C HSQC NMR Spectrum (500 MHz, 25 °C) of olefin macrocycle **OM** in $CDCl_3$.

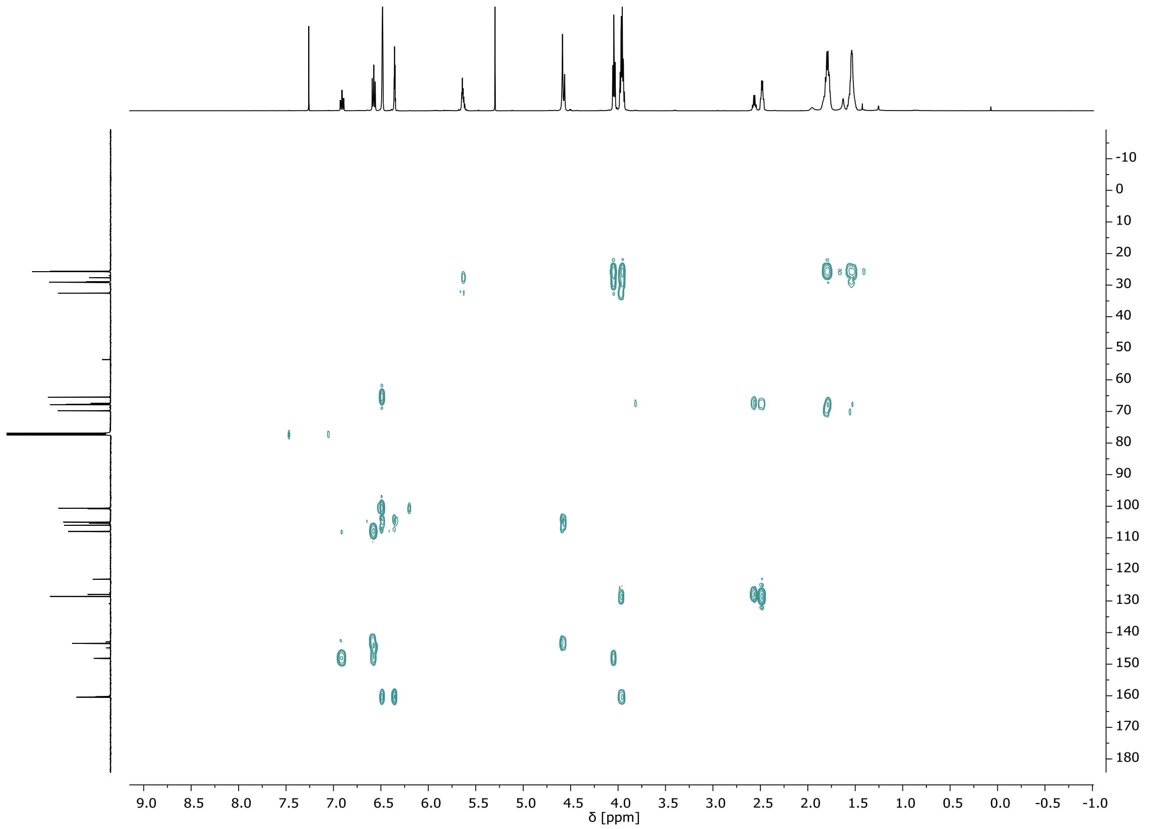

^{1}H, ^{13}C HMBC NMR Spectrum (500 MHz, 25 °C) of olefin macrocycle **OM** in $CDCl_3$.

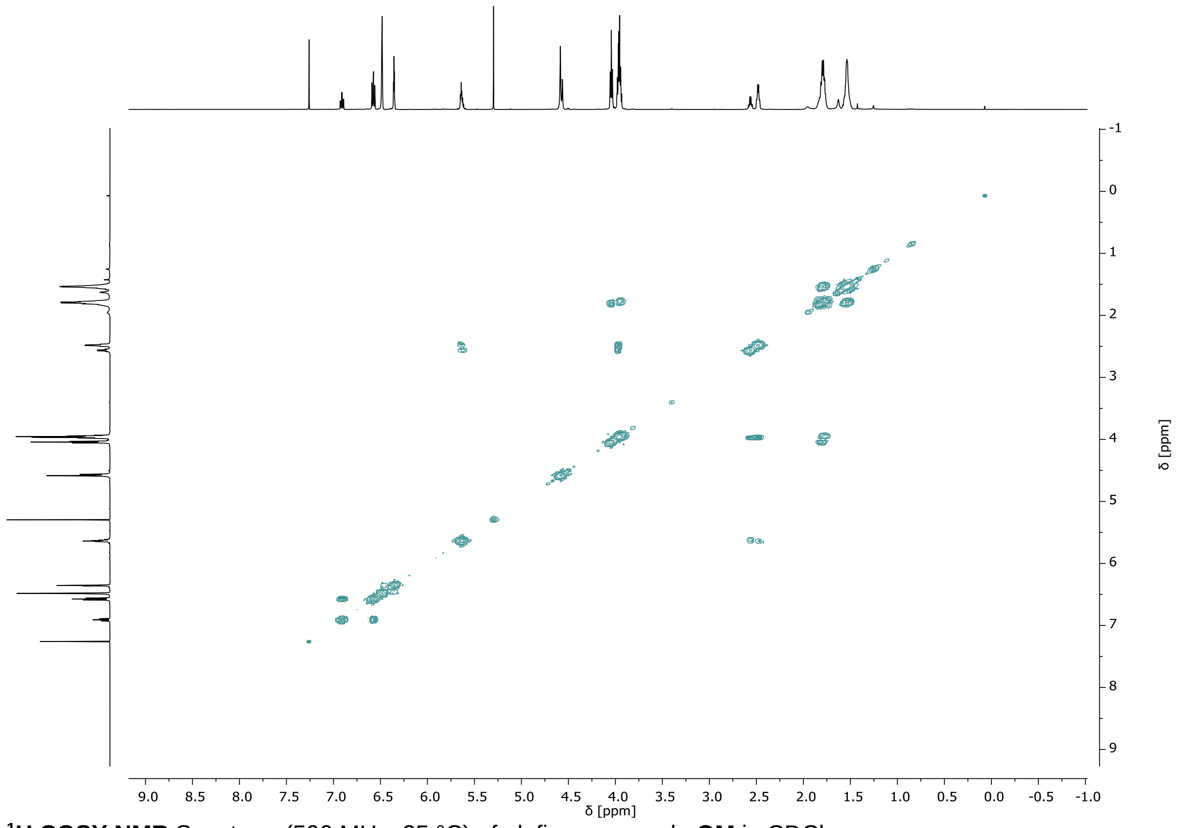

^{1}H COSY NMR Spectrum (500 MHz, 25 °C) of olefin macrocycle **OM** in $CDCl_3$.

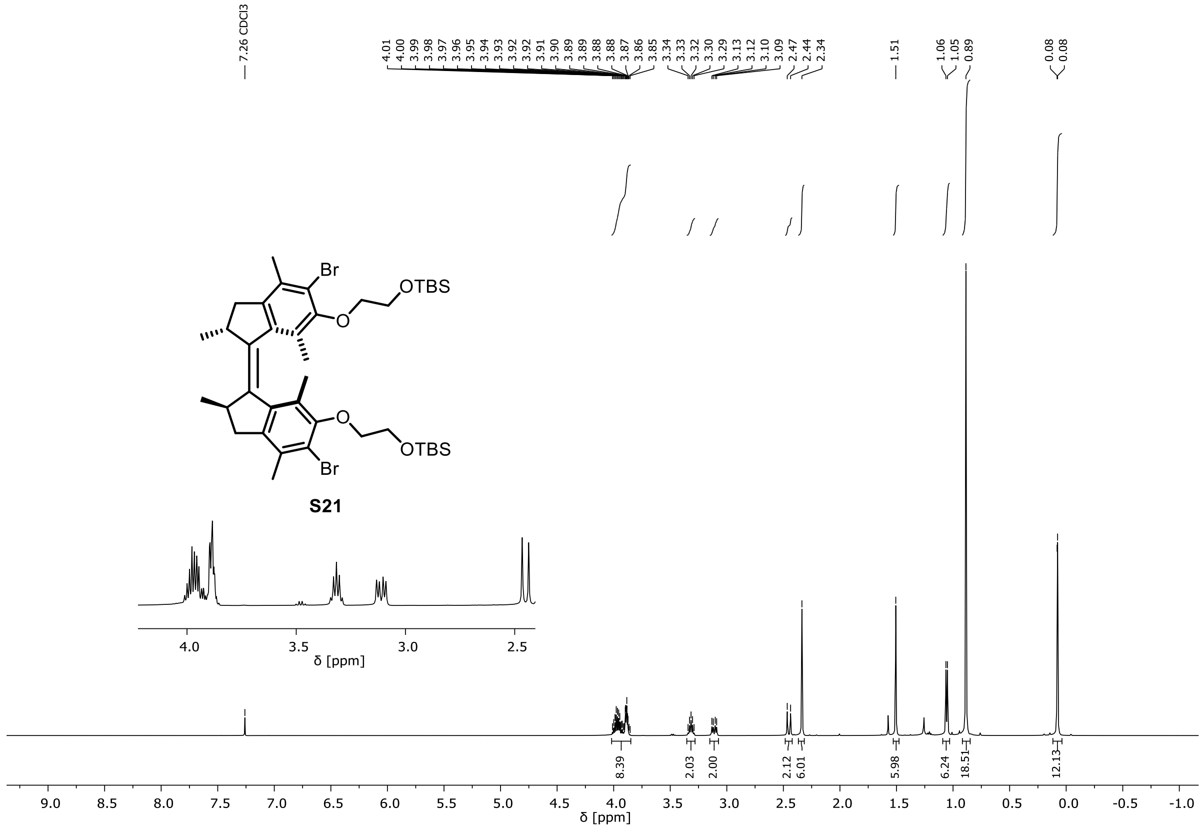

^{1}H NMR Spectrum (500 MHz, 25 °C) of **S21** in $CDCl_3$.

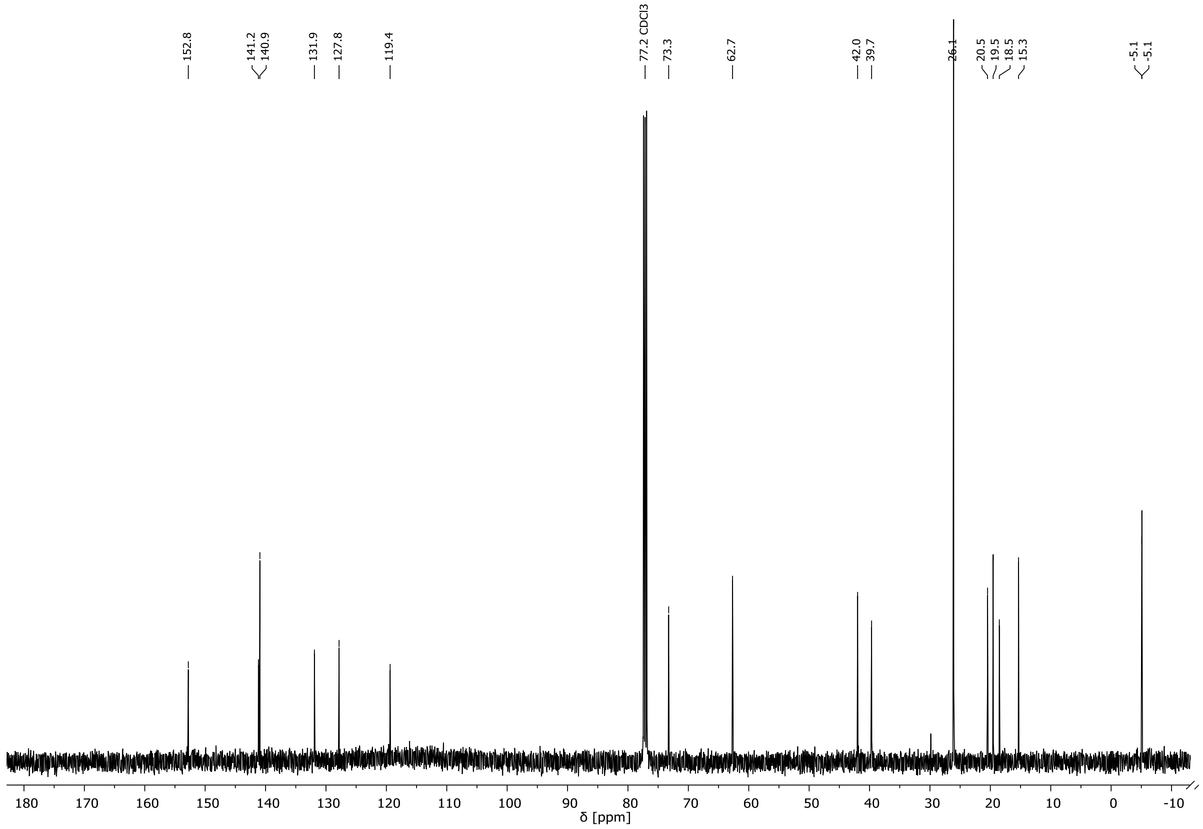

^{13}C NMR Spectrum (126 MHz, 25 °C) of **S21** in $CDCl_3$.

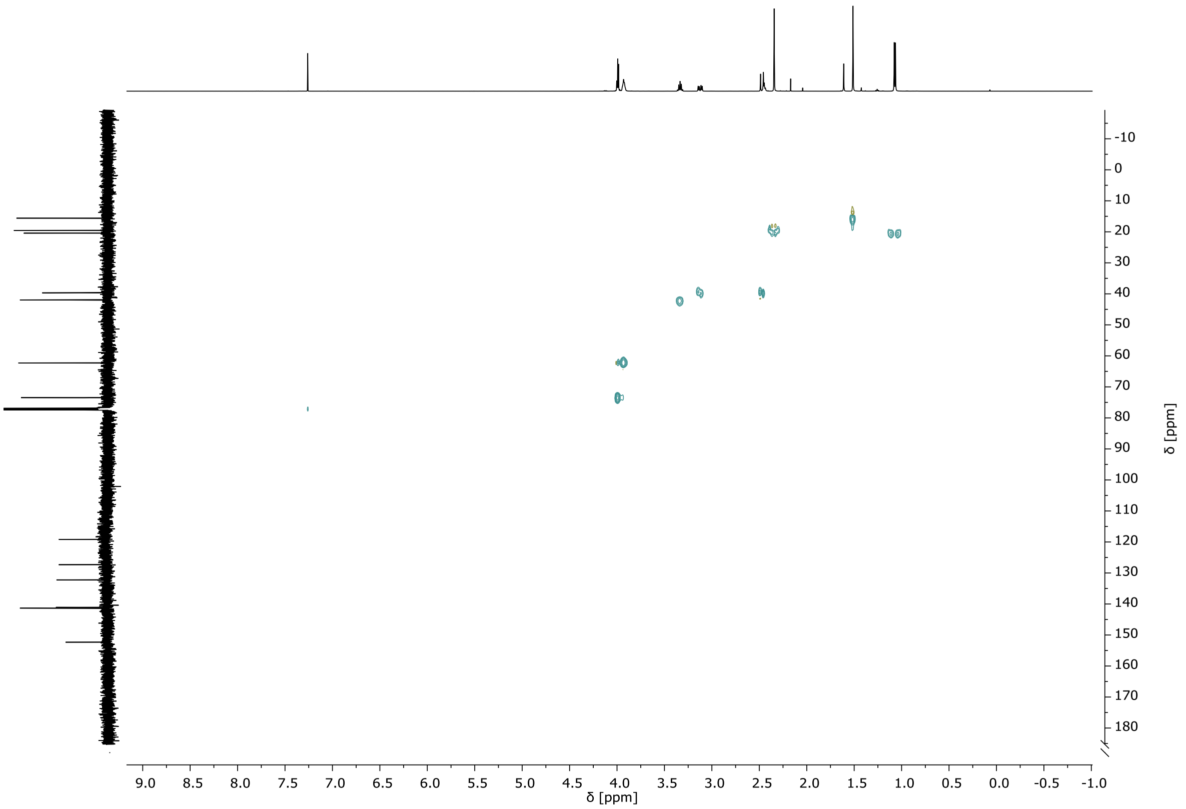

¹H, ¹³C HSQC NMR Spectrum (500 MHz, 25 °C) of **S21** in $CDCl_3$.

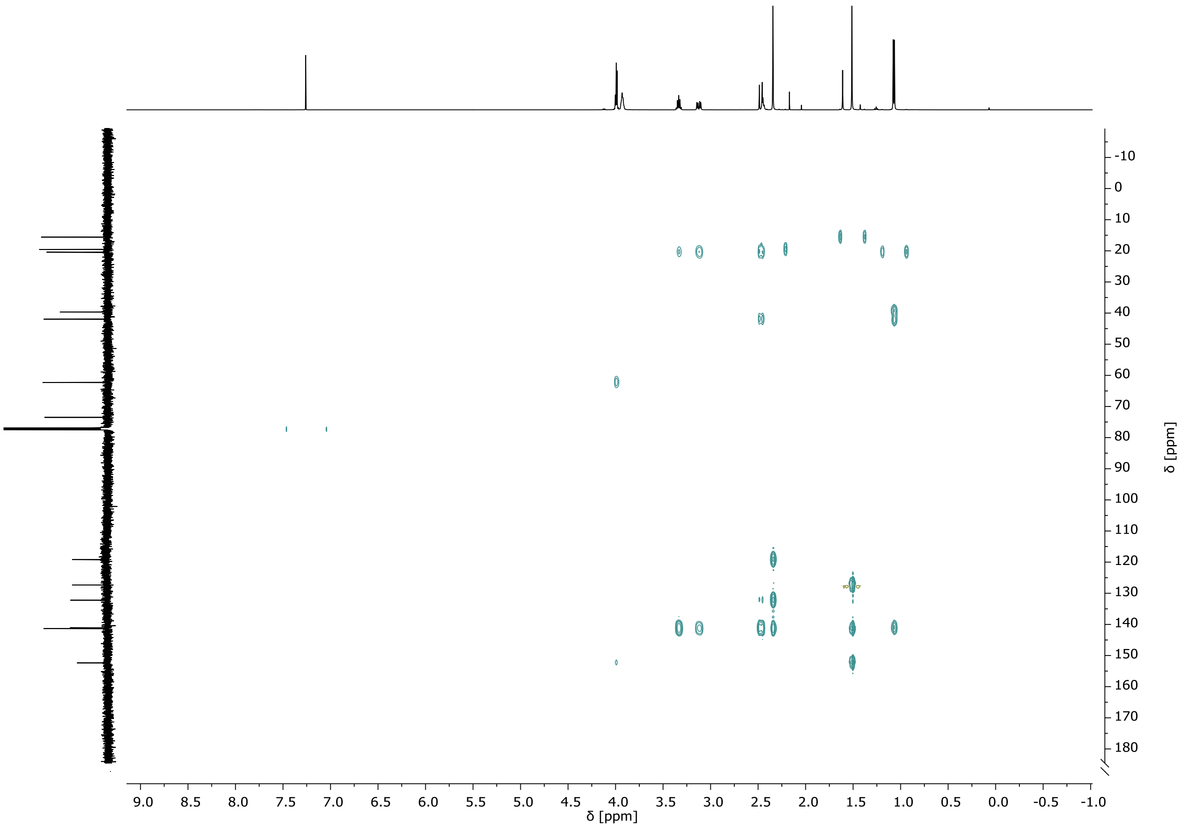

¹H, ¹³C HMBC NMR Spectrum (500 MHz, 25 °C) of **S21** in $CDCl_3$.

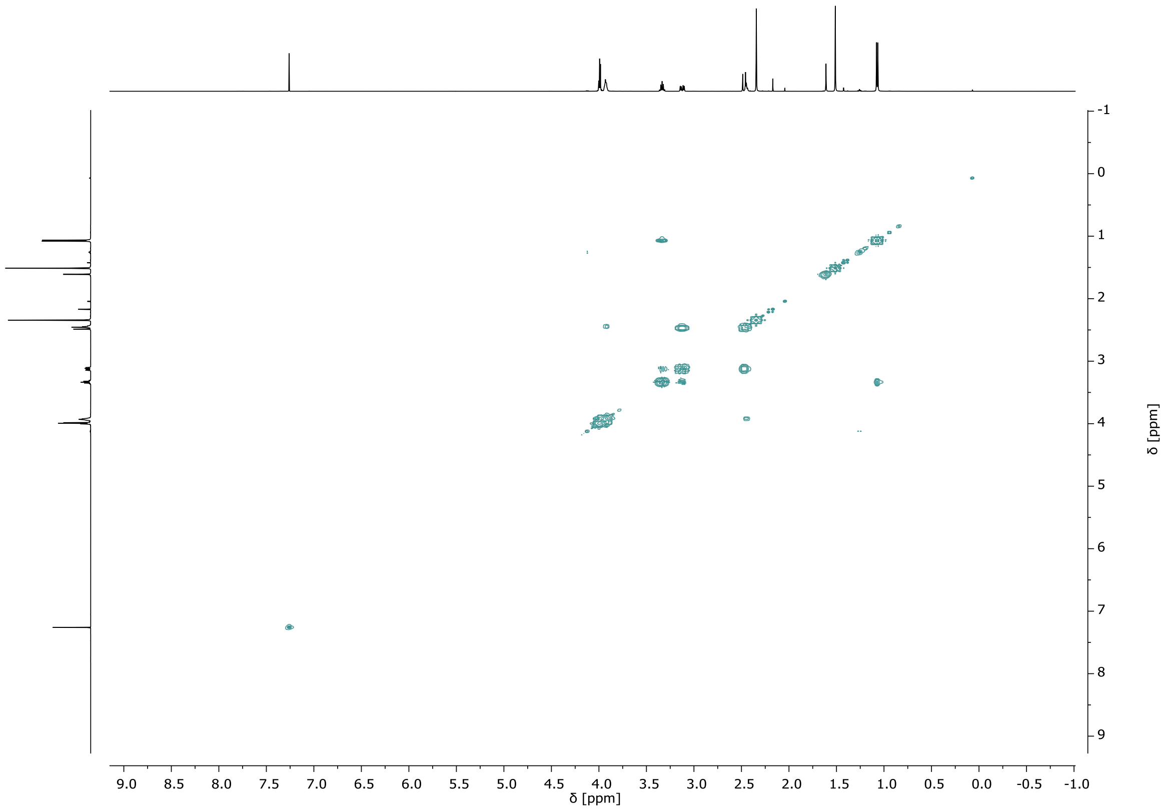

^{1}**H COSY NMR** Spectrum (500 MHz, 25 °C) of **S21** in $CDCl_3$.

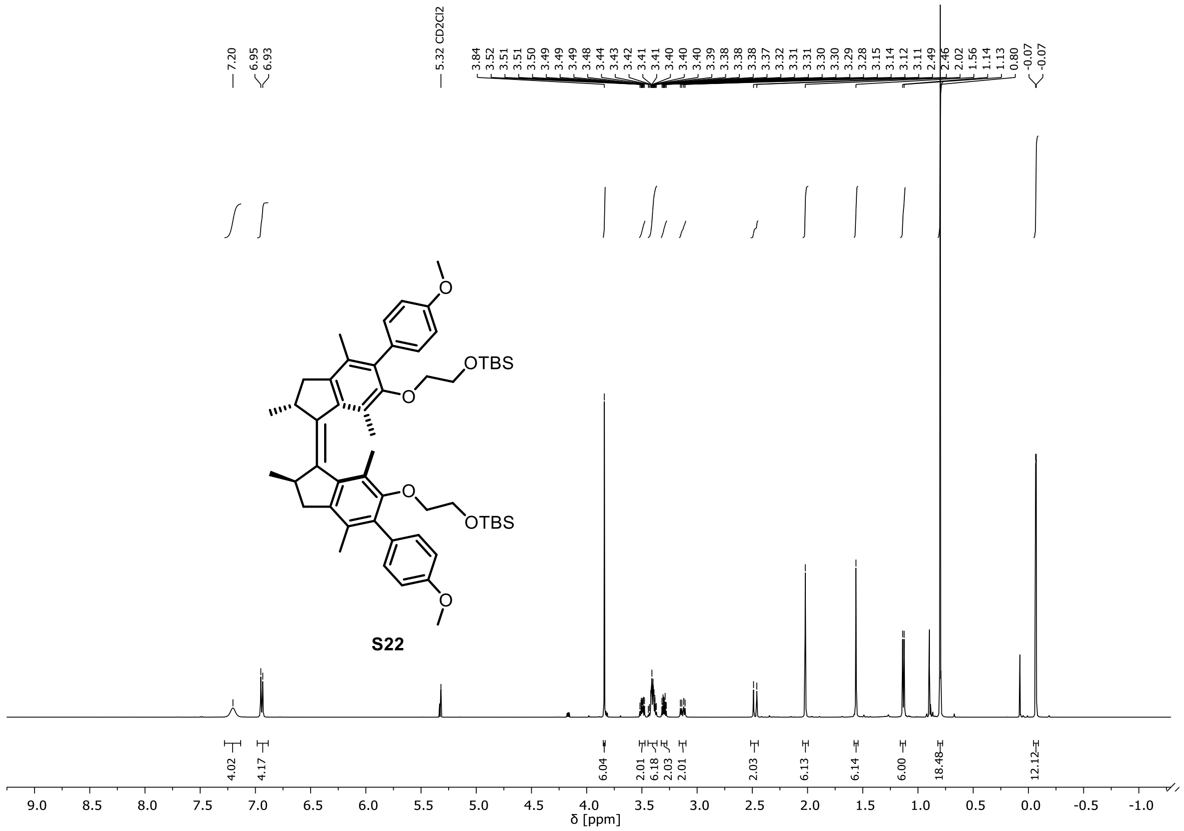

^{1}H NMR Spectrum (500 MHz, 25 °C) of **S22** in $CDCl_3$.

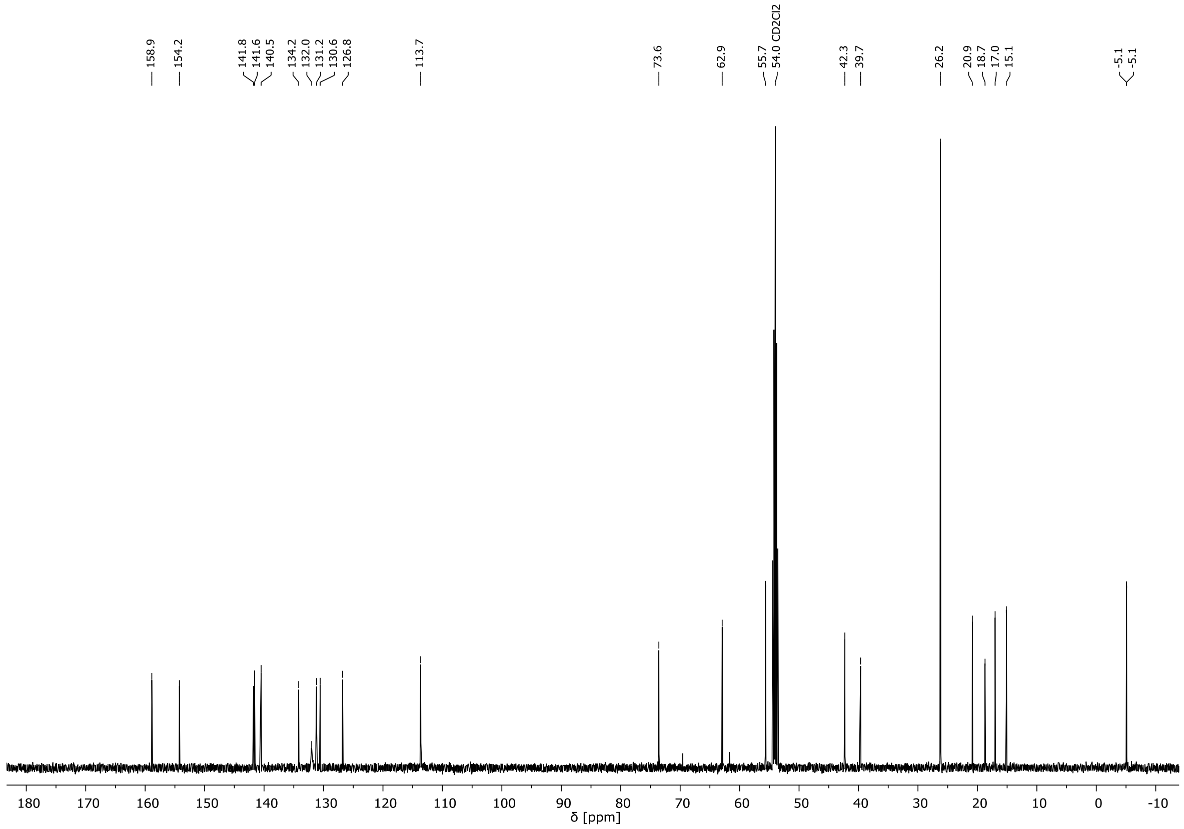

^{13}C NMR Spectrum (126 MHz, 25 °C) of **S22** in $CDCl_3$.

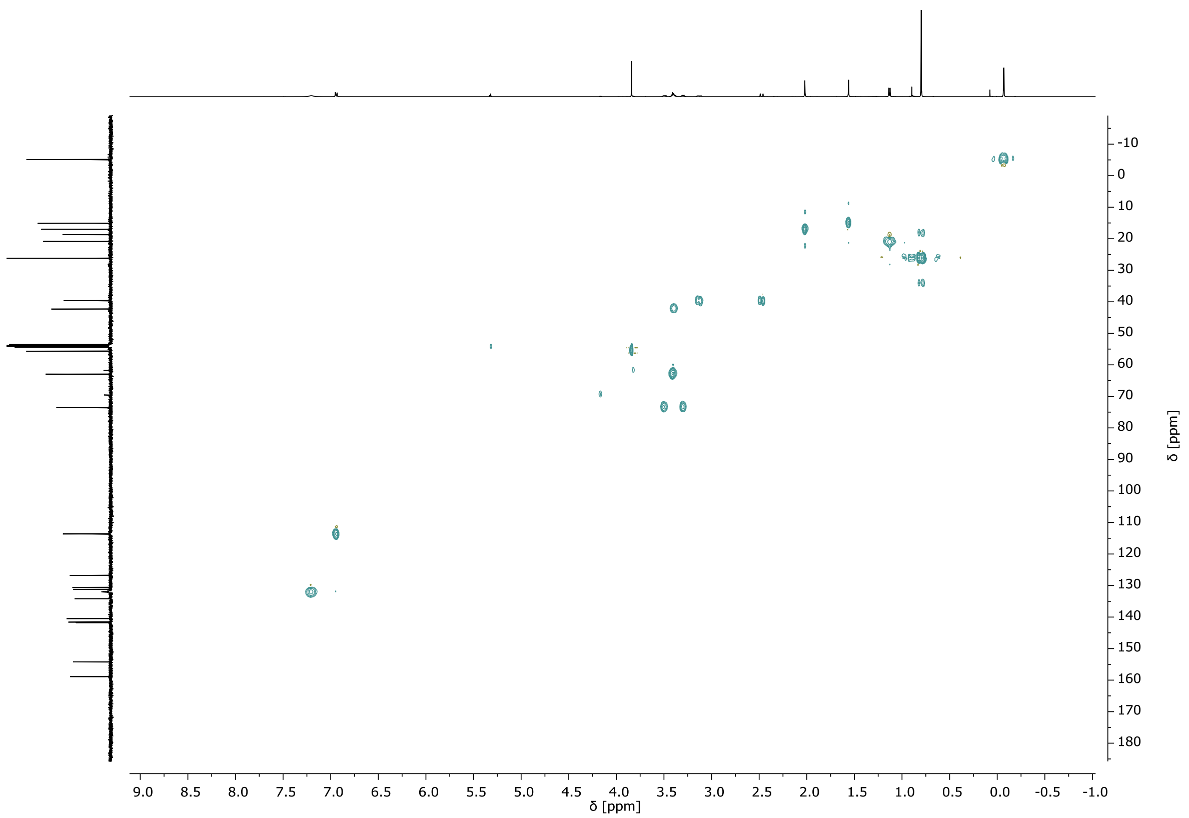

^{1}H, ^{13}C HSQC NMR Spectrum (500 MHz, 25 °C) of **S22** in $CDCl_3$.

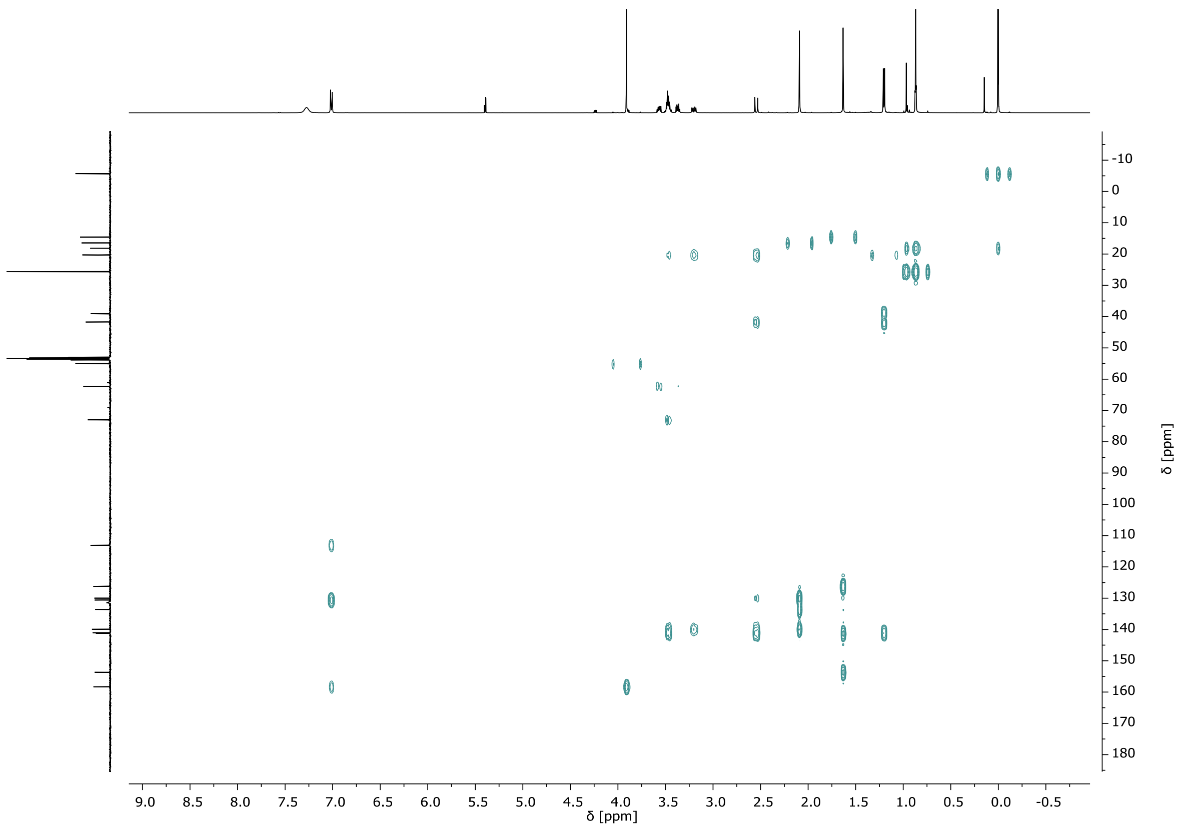

^{1}H, ^{13}C HMBC NMR Spectrum (500 MHz, 25 °C) of **S22** in $CDCl_3$.

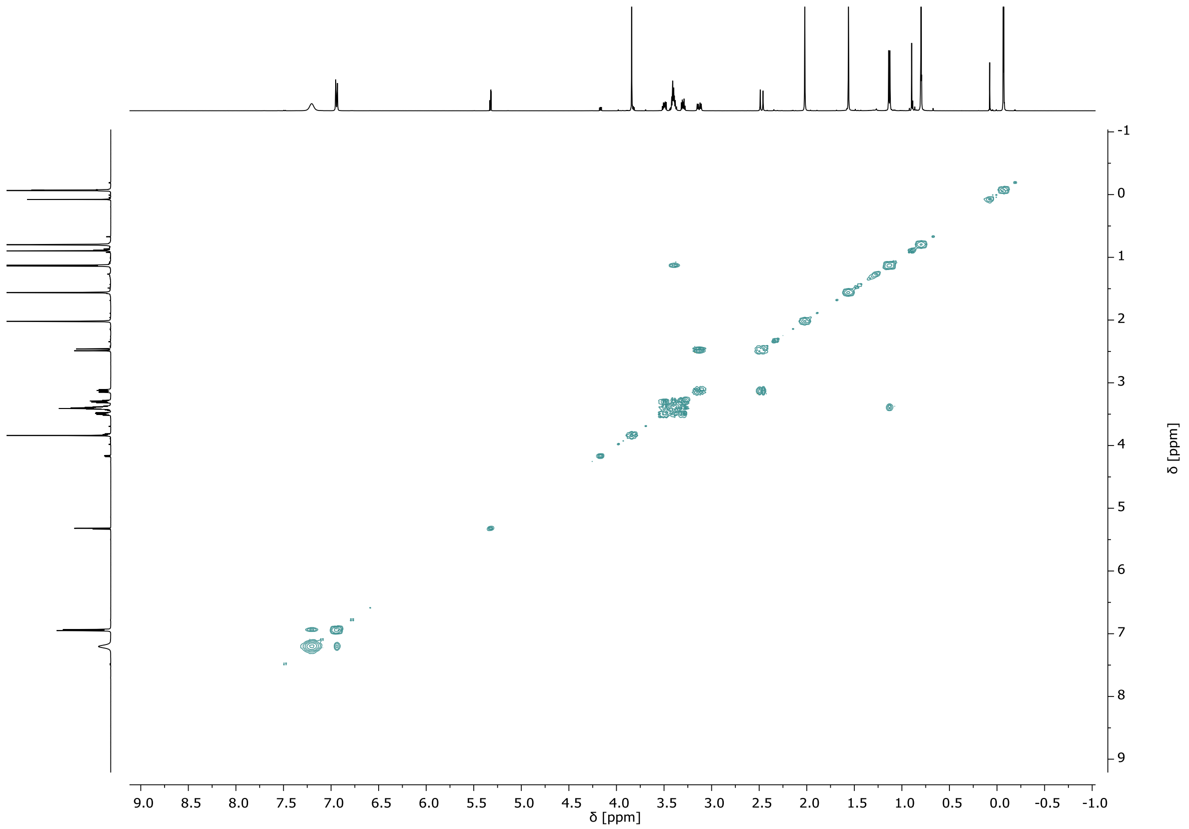

^{1}H COSY NMR Spectrum (500 MHz, 25 °C) of **S22** in $CDCl_3$.

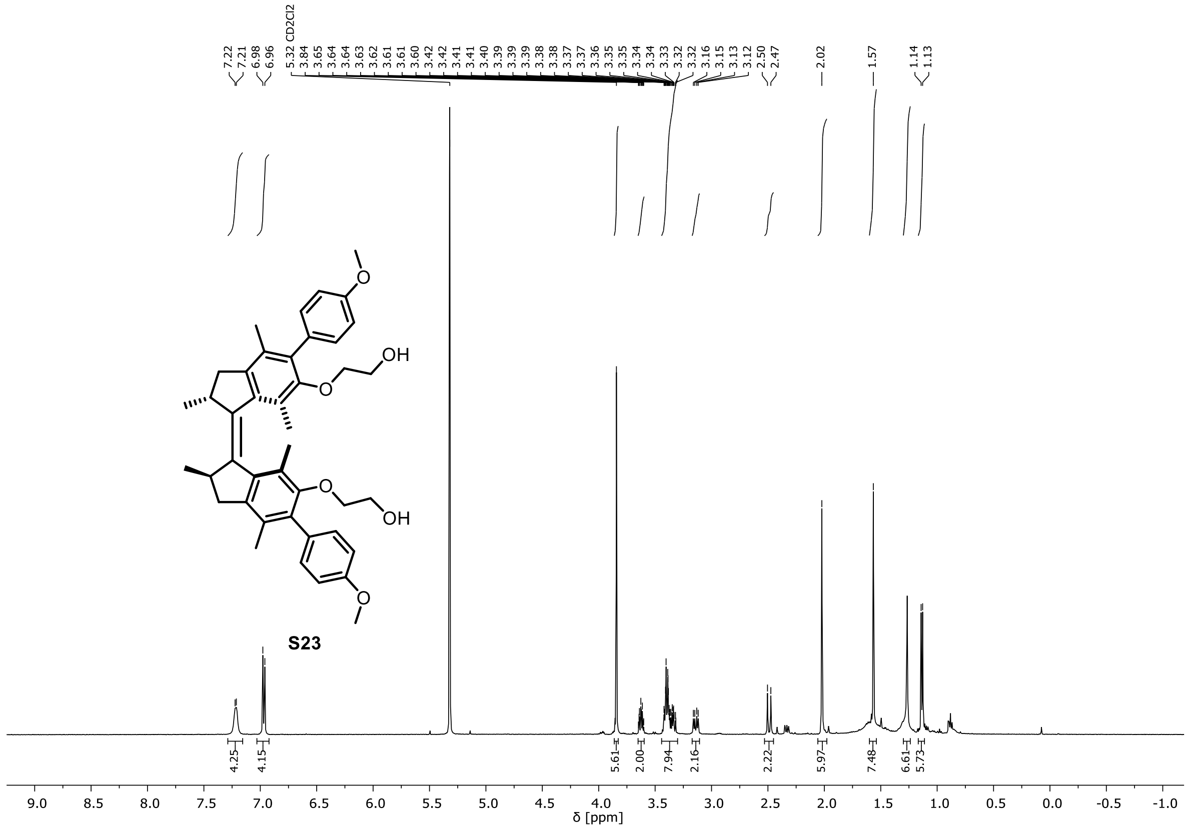

^{1}H NMR Spectrum (500 MHz, 25 °C) of **S23** in $CDCl_3$.

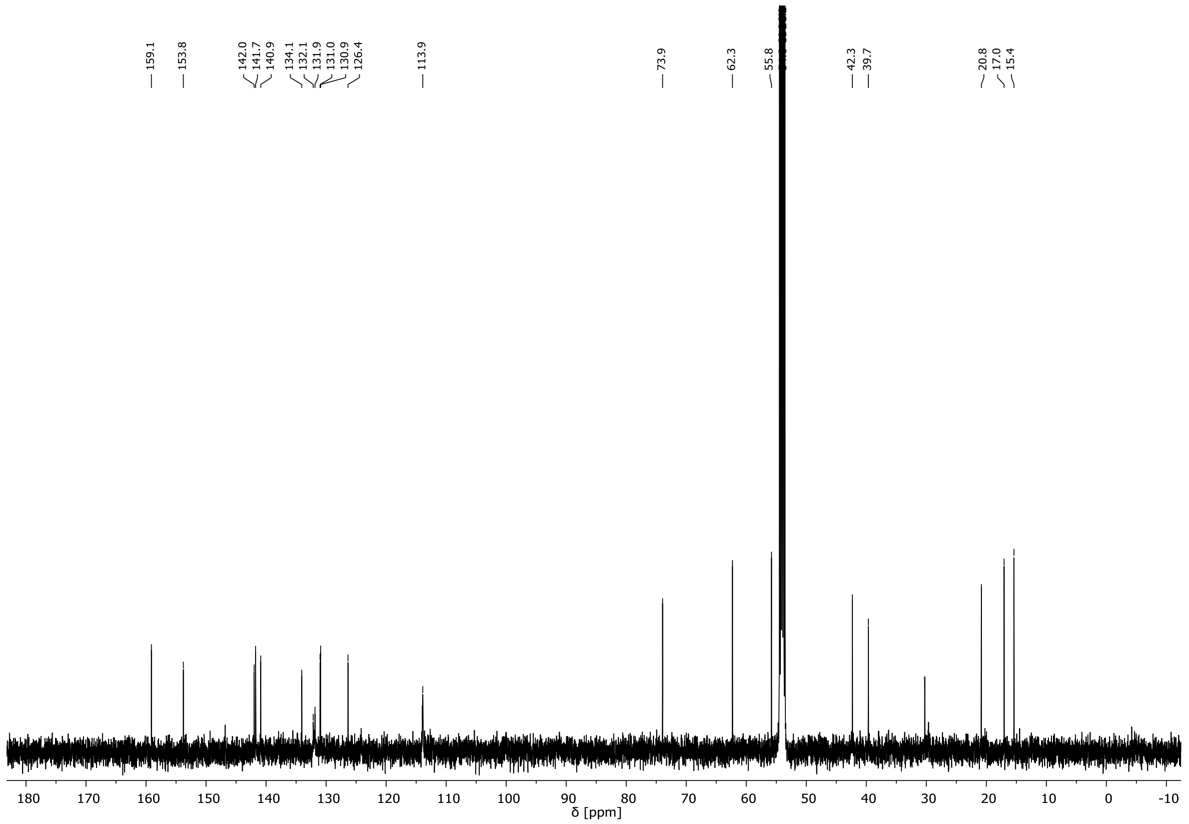

^{13}C NMR Spectrum (126 MHz, 25 °C) of **S23** in $CDCl_3$.

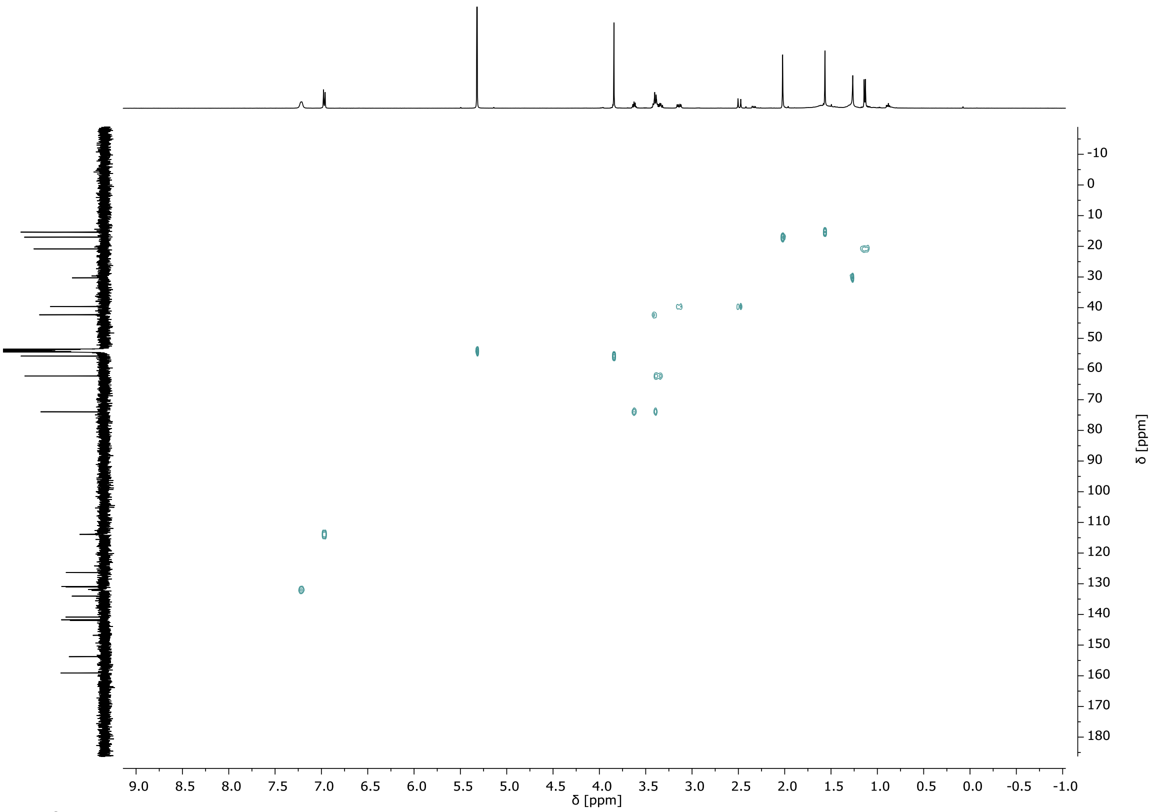

^{1}H, ^{13}C HSQC NMR Spectrum (500 MHz, 25 °C) of **S23** in $CDCl_3$.

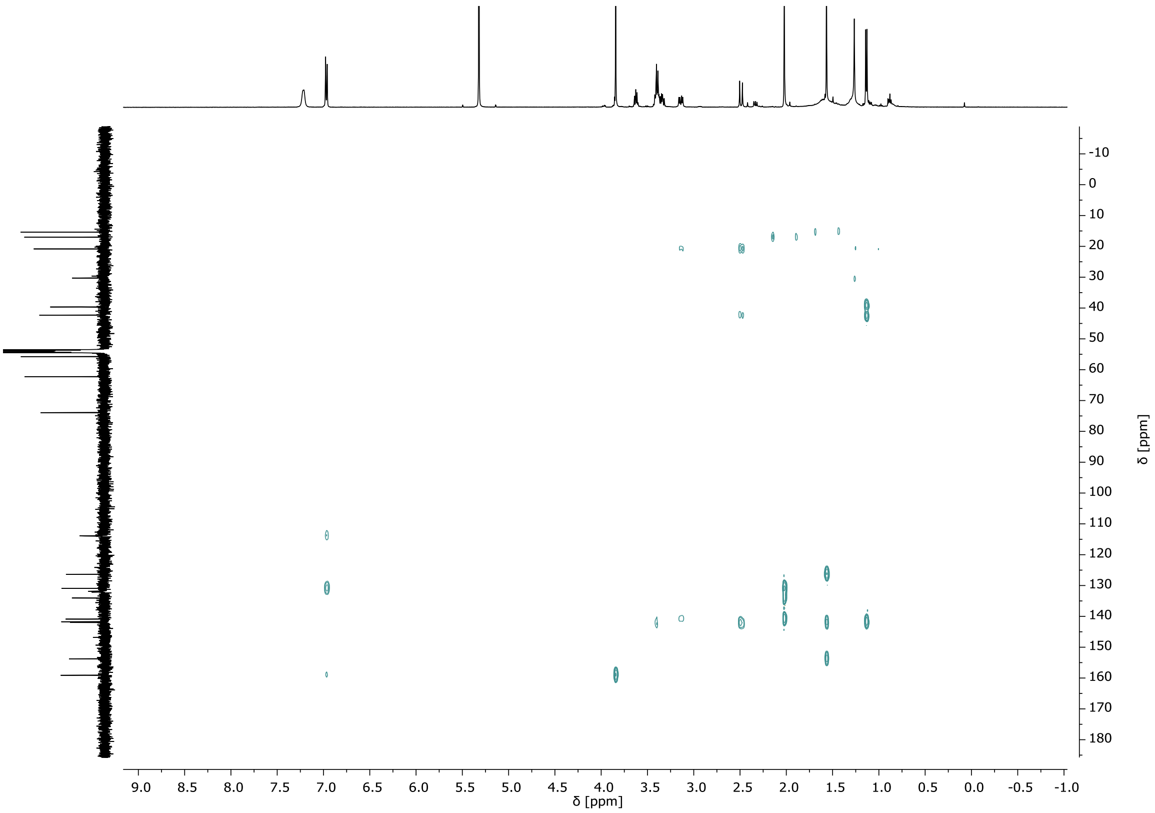

^{1}H, ^{13}C HMBC NMR Spectrum (500 MHz, 25 °C) of **S23** in $CDCl_3$.

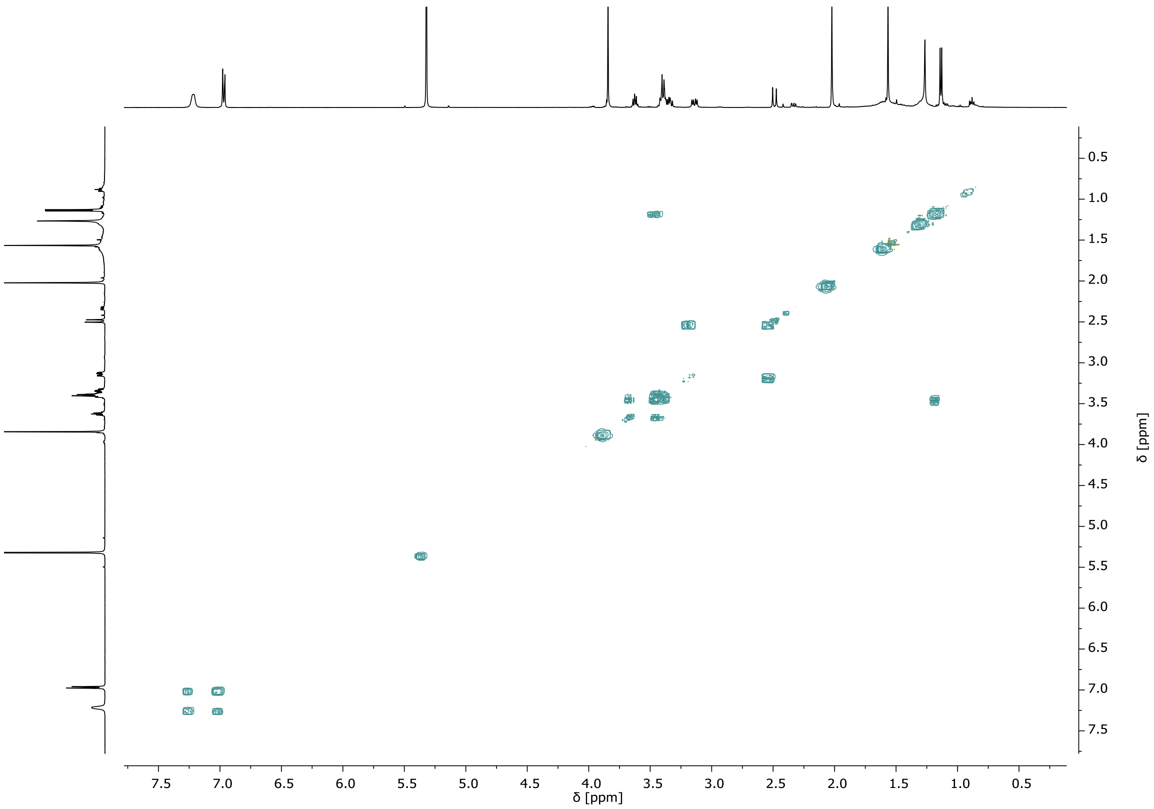

^{1}H COSY NMR Spectrum (500 MHz, 25 °C) of **S23** in $CDCl_3$.

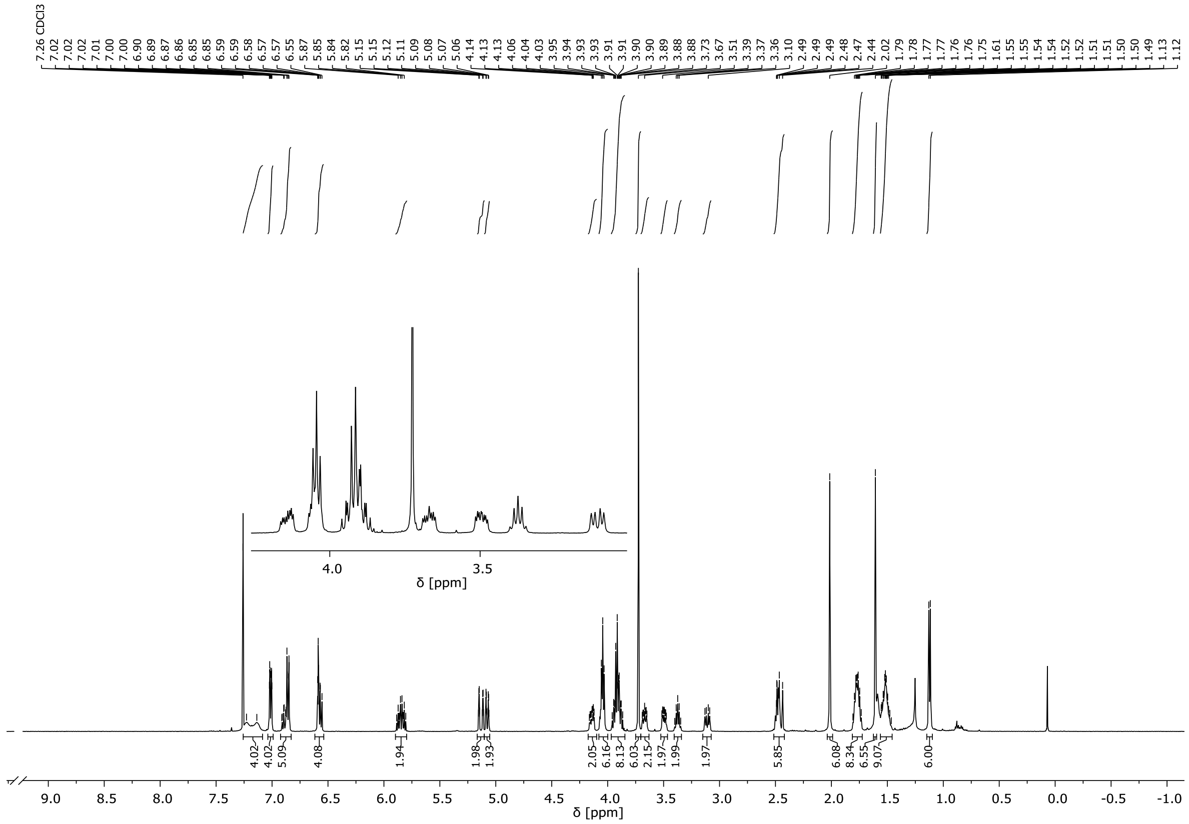

^{1}H NMR Spectrum (500 MHz, 25 °C) of motor macrocycle (*Z*s)-**MM** in $CDCl_3$.

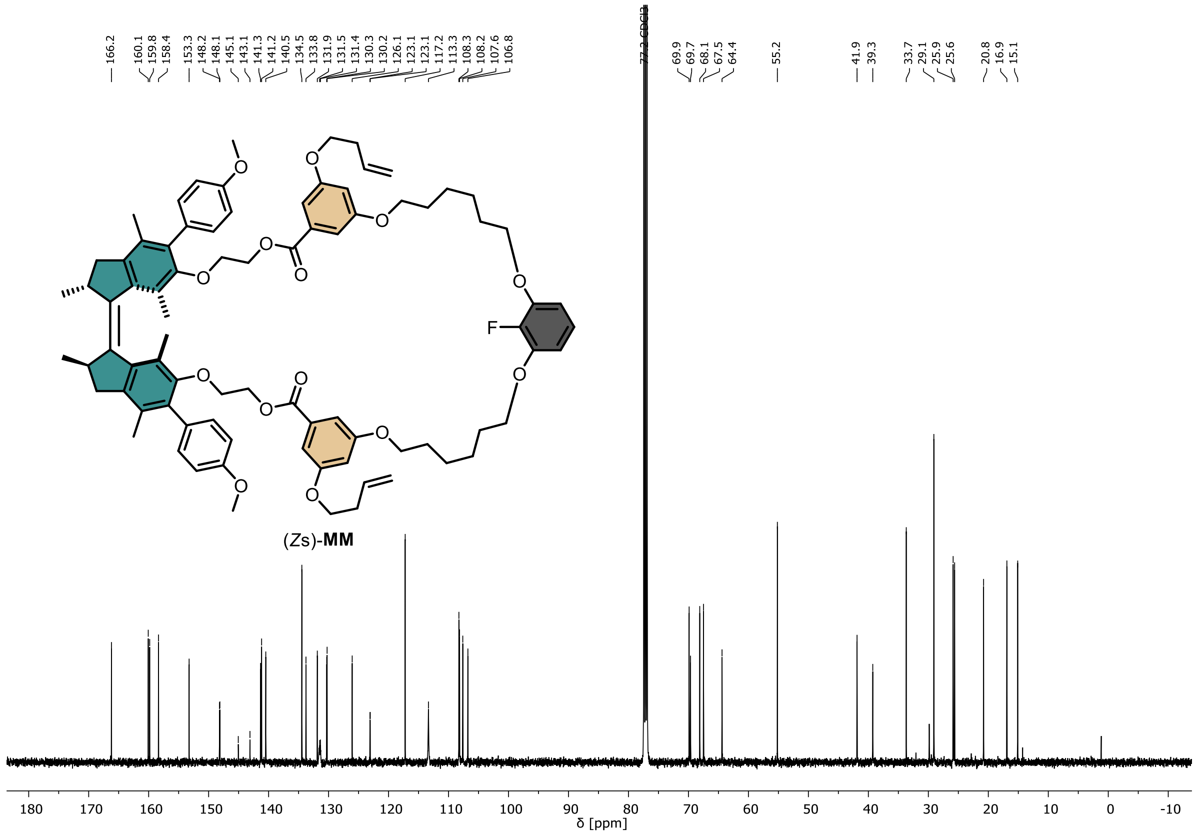

^{13}C NMR Spectrum (126 MHz, 25 °C) of motor macrocycle (*Z*s)-**MM** in $CDCl_3$.

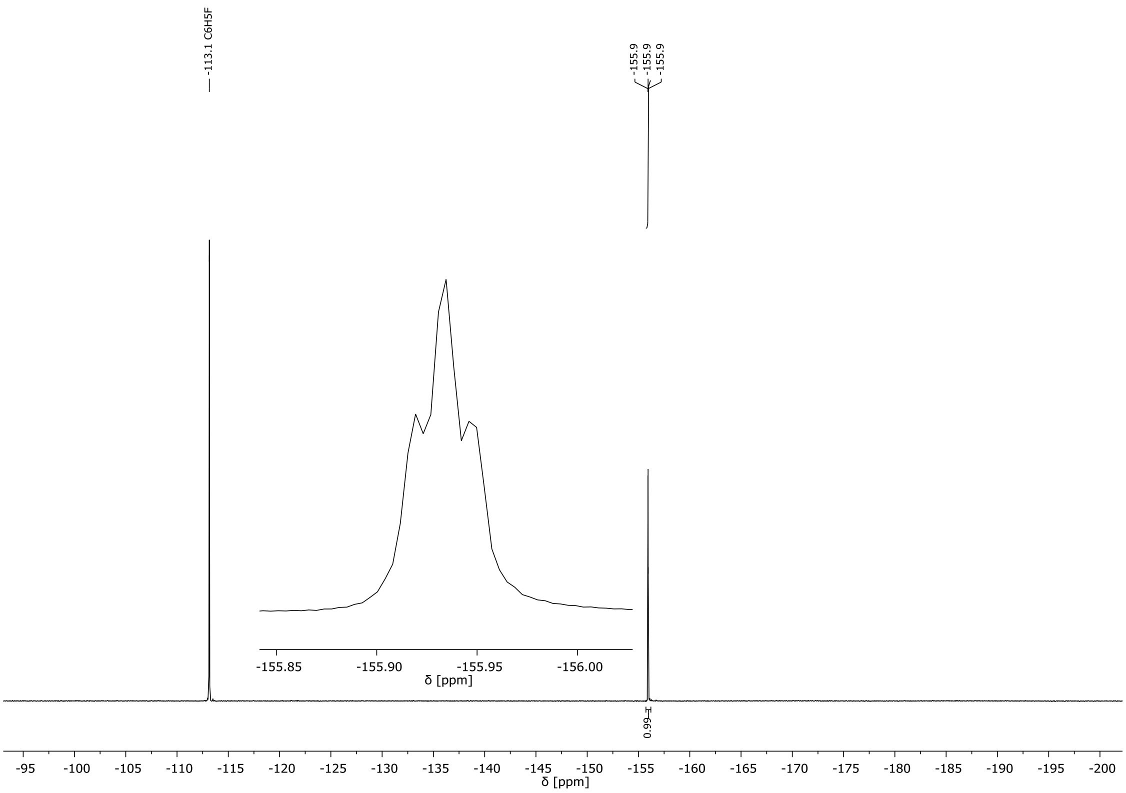

^{19}F NMR Spectrum (471 MHz, 25 °C) of motor macrocycle (*Z*s)-**MM** in $CDCl_3$.

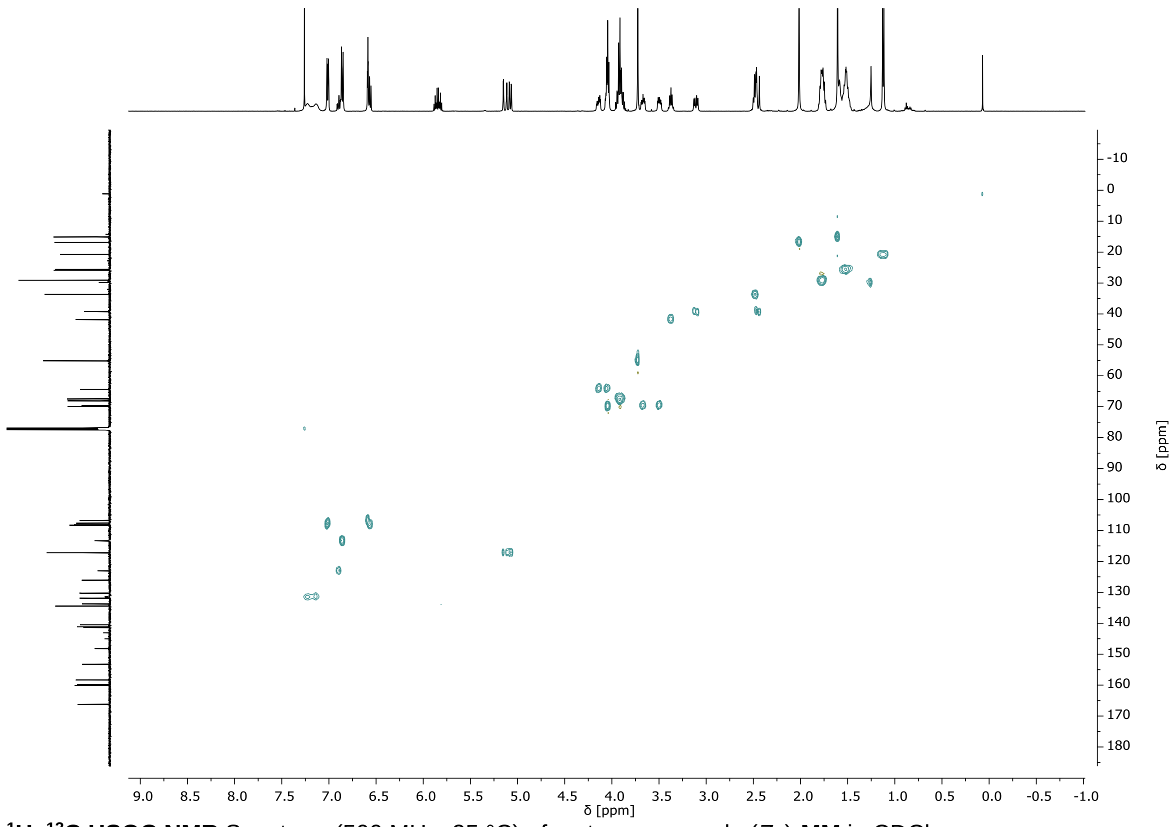

^{1}H, ^{13}C HSQC NMR Spectrum (500 MHz, 25 °C) of motor macrocycle (*Z*s)-**MM** in $CDCl_3$.

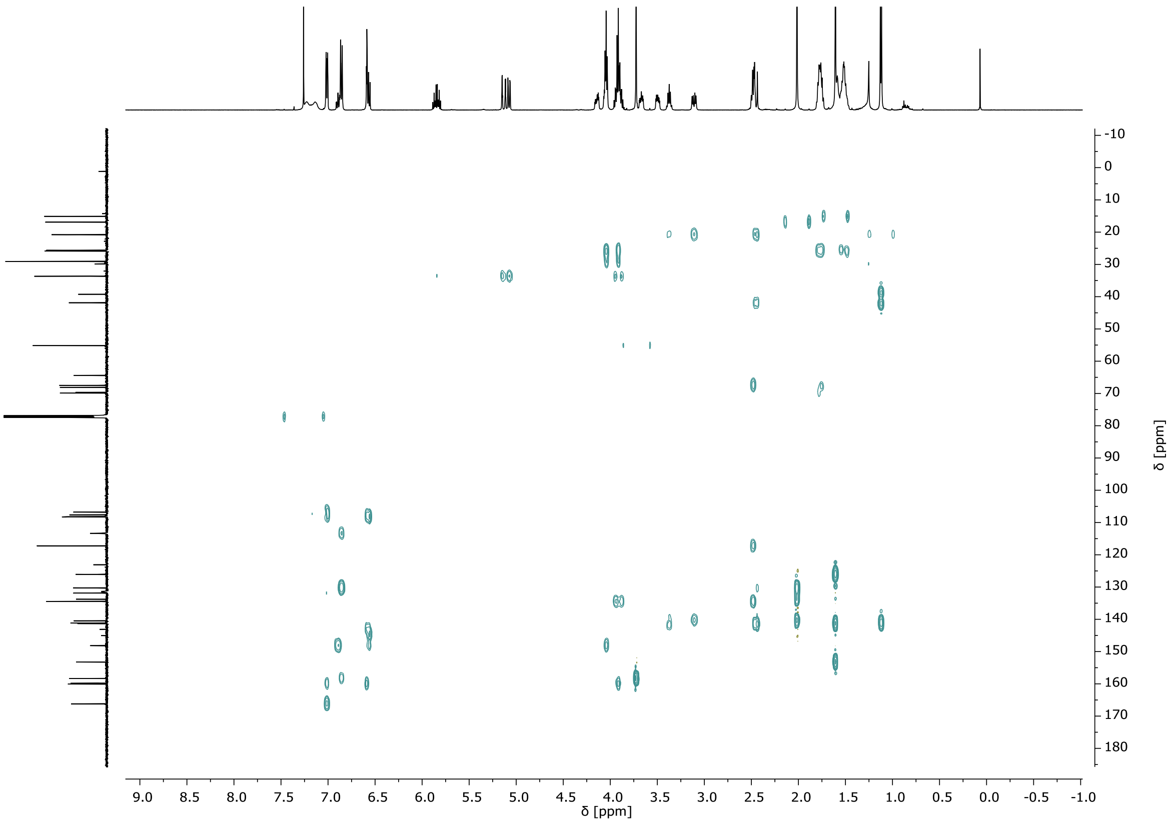

¹H, ¹³C HMBC NMR Spectrum (500 MHz, 25 °C) of motor macrocycle (*Z*s)-**MM** in $CDCl_3$.

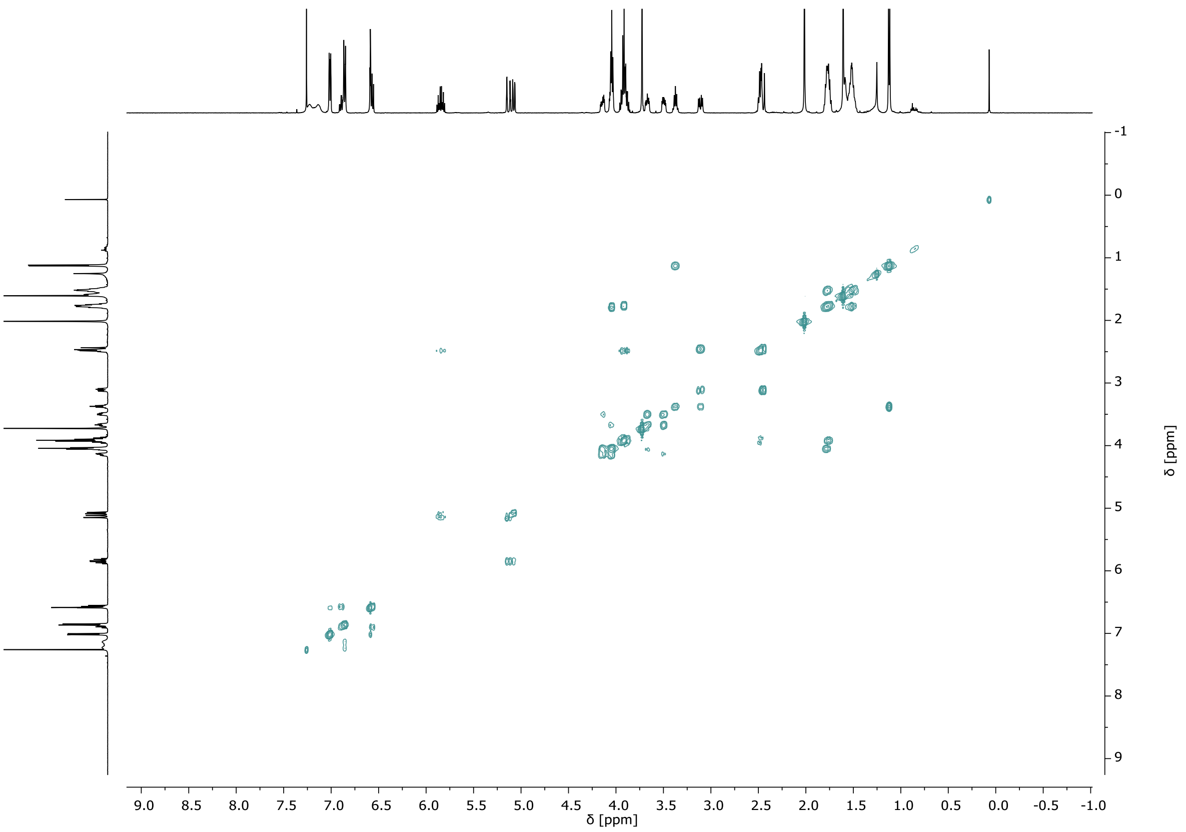

¹H COSY NMR Spectrum (500 MHz, 25 °C) of motor macrocycle (*Z*s)-**MM** in $CDCl_3$.
